# Effect and process evaluation of a kindergarten-based, family-involved intervention with a randomized cluster design on sedentary behaviour in 4- to 6- year old European preschool children: The ToyBox-study

**DOI:** 10.1371/journal.pone.0172730

**Published:** 2017-04-05

**Authors:** Julie Latomme, Greet Cardon, Ilse De Bourdeaudhuij, Violeta Iotova, Berthold Koletzko, Piotr Socha, Luis Moreno, Odysseas Androutsos, Yannis Manios, Marieke De Craemer, On behalf of ToyBox-study group

**Affiliations:** 1 Ghent University, Department of Movement and Sport Sciences, Ghent, Belgium; 2 Medical University Varna, Clinic of Paediatric Endocrinology, Varna, Bulgaria; 3 University of Munich Medical Centre, Dr. von Hauner Children’s Hospital, Munich, Germany; 4 Children’s Memorial Institute, Warsaw, Poland; 5 University of Zaragoza, GENUD (Growth, Exercise, Drinking behaviour and Development), Zaragoza, Spain; 6 Harokopio University, School of Health Science & Education, Department of Nutrition and Dietetics, Athens, Greece; Vanderbilt University, UNITED STATES

## Abstract

**Background:**

The aim of the present study evaluated the effect and process of the ToyBox-intervention on proxy-reported sedentary behaviours in 4- to 6-year-old preschoolers from six European countries.

**Methods:**

In total, 2434 preschoolers’ parents/primary caregivers (mean age: 4.7±0.4 years, 52.2% boys) filled out a questionnaire, assessing preschoolers’ sedentary behaviours (TV/DVD/video viewing, computer/video games use and quiet play) on weekdays and weekend days. Multilevel repeated measures analyses were conducted to measure the intervention effects. Additionally, process evaluation data were included to better understand the intervention effects.

**Results:**

Positive intervention effects were found for computer/video games use. In the total sample, the intervention group showed a smaller increase in computer/video games use on weekdays (ß = -3.40, p = 0.06; intervention: +5.48 min/day, control: +8.89 min/day) and on weekend days (ß = -5.97, p = 0.05; intervention: +9.46 min/day, control: +15.43 min/day) from baseline to follow-up, compared to the control group. Country-specific analyses showed similar effects in Belgium and Bulgaria, while no significant intervention effects were found in the other countries. Process evaluation data showed relatively low teachers’ and low parents’ process evaluation scores for the sedentary behaviour component of the intervention (mean: 15.6/24, range: 2.5–23.5 and mean: 8.7/17, range: 0–17, respectively). Higher parents’ process evaluation scores were related to a larger intervention effect, but higher teachers’ process evaluation scores were not.

**Conclusions:**

The ToyBox-intervention had a small, positive effect on European preschoolers’ computer/video games use on both weekdays and weekend days, but not on TV/DVD/video viewing or quiet play. The lack of larger effects can possibly be due to the fact that parents were only passively involved in the intervention and to the fact that the intervention was too demanding for the teachers. Future interventions targeting preschoolers' behaviours should involve parents more actively in both the development and the implementation of the intervention and, when involving schools, less demanding activities for teachers should be developed.

**Trial registration:**

clinicaltrials.gov NCT02116296

## Background

In early childhood, the prevalence of overweight and obesity has increased and became a worldwide health problem [1]. In 1990, the prevalence of overweight and obesity in children under the age of five was globally estimated to be 4.2%, in 2010 it increased to 6.7%, and it is estimated to be 9.1% in 2020 [1]. The most important cause of overweight and obesity is an energy imbalance, in which the body gains weight because the energy intake exceeds the energy expenditure [2]. An important behaviour that influences the energy balance is sedentary behaviour (SB), which can be defined as any waking behaviour that encompasses sitting or lying as the dominant posture (such as TV viewing or traveling in a car) and requires low levels of energy expenditure (i.e. <1.5 metabolic equivalents of rest) [3, 4]. Typical SBs during preschool years are TV viewing (including DVD and video viewing), computer use (including use of games and consoles) and quiet play (e.g. reading, playing with dolls, making puzzles, etc.) [5, 6].

In many European preschool children under the age of five, high levels of time spent on these SBs can be found and high proportions of preschoolers exceed the guideline of maximum one hour screen time per day (e.g. in Europe, 29% (Germany) to 75% (Bulgaria) exceeds this guideline on weekdays and 48% (Germany) to 91% (Bulgaria) exceeds this guideline on weekend days) [7]. Therefore, SB in preschoolers has become a new focus of interest [8, 9]. However, only a limited number of intervention studies can be found in the literature targeting preschoolers’ SB [8]. In a study of Dennison et al. [10], a preschool-based intervention consisting of a 7-session program on TV viewing (in which 1 session per week was given consisting of a 20-minute interactive, educational component for both the child, parent and caregiver) was developed to reduce TV and video viewing at home in American preschool children under the age of five. The results showed a reduction of 3.1 hours/week in preschoolers’ amount of TV/video viewing. A family-based ‘Active Play’ intervention developed by O’Dwyer et al. [11], consisting of a 10-week active play program involving both the child and the parent in an activity and educational component, showed also a significant reduction in sedentary time (-1.5% on weekdays and -4.3% on weekend days) in UK preschoolers’ under the age of 5. Also a school-based active play intervention of O’Dwyer et al. [12] with a 6-week active play program aimed to decrease preschoolers’ sedentary time, but no significant intervention effects were observed in this study.

Interventions targeting preschoolers’ SB often focus on reducing screen time, because screen time -and TV viewing in particular- is frequently used as a proxy marker of overall SB in this age group [5, 13]. However, quiet play (e.g. tinkering, puzzling, reading, playing with dolls, etc.) is another common SB in preschoolers [14]. Furthermore, it is possible that a decrease in one SB (e.g. TV/DVD/video viewing) is compensated by an increase in another SB (e.g. quiet play or computer/video games use). Until today, no intervention for preschoolers could be detected in the literature targeting several components of SB simultaneously.

The overall aim of the ToyBox-intervention was to prevent overweight and obesity in 4- to 6- year old preschoolers, by promoting four energy balance related behaviours: (1) physical activity (PA), (2) reduction/interruption of SB, (3) water consumption and (4) healthy snacking. Regarding the SB-behaviour, the ToyBox-intervention aimed to target several components of SB simultaneously. The ToyBox-intervention is framed within a socio-ecological perspective [15] [16] because it is important for prevention strategies to focus both on the individual level (i.e. preschoolers) and the environmental level (i.e. teachers and parents/primary caregivers).

The first aim of the present study was to investigate the effect of the ToyBox-intervention on European preschoolers’ SBs (i.e. TV/DVD/video viewing, computer/video games use and quiet play). The effects on the other behaviours have been reported elsewhere [17, 18]. Additionally, we investigated whether the intervention-effects were different in the six European countries.

Besides evaluating the effect of the ToyBox-intervention, it is also important to evaluate the process of the intervention, because the variability in the effectiveness of an intervention can depend on the level of implementation [19, 20]. Combining the effect evaluation data with the process evaluation data will therefore result in a better understanding of the (lack of) effects of a health promotion intervention [21]. Therefore, the second aim of the study was to investigate whether higher teachers’ and parents’ process evaluation scores of the SB-component of the intervention was related to a larger intervention effect.

## Methods

### Ethics approval and consent to participate

We declare that all applicable institutional regulations pertaining to the ethical use of human volunteers were followed during this research. Ethical approval was provided by the Ethical Committees of all participating European countries (i.e. Ethical committee of Ghent University Hospital (Belgium), Committee for the Ethics of the Scientific Studies (KENI) at the Medical University of Varna (Bulgaria), Ethikkommission der Ludwig- Maximilians-Universität München (Germany), the Ethics Committee of Harokopio of Athens (Greece), Ethical Committee of Children’s Memorial Health Institute (Poland), and CEICA (Comité Etico de Investigacion Clinica de Aragon (Spain)).

Participants received an information letter in which they were briefly informed about the purpose of the study. By signing a written informed consent, they gave their consent to participation in the study.

### Study protocol

The kindergarten-based, family-involved ToyBox-intervention had a randomized cluster (pre-test/post-test) design including intervention and control kindergartens across six European countries: Belgium, Bulgaria, Germany, Greece, Poland and Spain [7, 22] (for the supporting CONSORT Checklist, see S1 Table). The pre-test measurements were conducted between May and June 2012, and the post-test measurements were conducted one year later, between May and June 2013.

**Fig 1:**
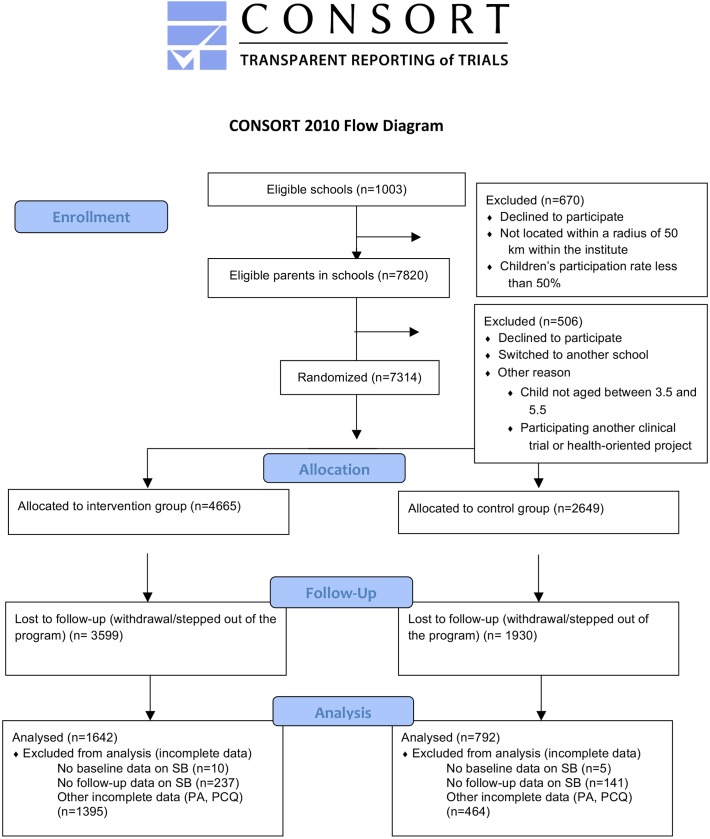
CONSORT Flow Diagram.

Per country, provinces were selected (i.e. West- and East-Flanders in Belgium, Varna in Bulgaria, Bavaria in Germany, Attica in Greece, Warsaw and surroundings in Poland, and Zaragoza in Spain). Within each of these provinces, lists of municipalities were created including information on socio-demographic background variables (i.e. mean years of education for the population of 25–55 years and/or annual income). Then, tertiles were created resulting in three groups of municipalities with different socio-demographic backgrounds, i.e. low socio-economic status (SES), medium SES and high SES municipalities. In each of these three groups, about five municipalities were randomly selected in each country, resulting in an equal distribution of preschoolers’ in each SES group [23]. Next, kindergartens, day-care centers or preschool settings across all countries were randomly selected within these municipalities. To enhance clarity, all these settings are referred to as "kindergartens" in the present paper. To inform the kindergarten staff about the purpose of the ToyBox-study, a visit was performed in each kindergarten. After the pre-test measurements (that took place at the kindergartens), the municipalities were randomly assigned to the intervention or the control group (2:1) (Fig 1). This randomization was done centrally by the coordinating center (i.e. Harokopio University Athens). Kindergartens allocated to the intervention group received the intervention materials to be used during the school year 2012–2013. Kindergartens allocated to the control group were informed that they would receive the intervention material one year later, and that they could continue with the normal kindergarten curriculum. Then, parents/primary caregivers of preschoolers born in 2007 and 2008 of the participating kindergartens received an information letter in which they were briefly informed about the purpose of the ToyBox-study. By signing a written informed consent, they gave their consent to participation in the study. All parents/primary caregivers who agreed to participate were asked to fill out a questionnaire measuring socio-demographic factors, lifestyle behaviours and perinatal factors, once before the intervention (i.e. pre-test) and once after the intervention (i.e. post-test). Power analyses were performed before the start of the intervention study. Based on current literature, a baseline value for children’s body mass index (BMI) = 16.35 kg m^−2^, an expected follow-up value of children’s BMI = 16.17 kg m^−2^, a standard deviation = 1.73, an α-value = 0.05 (two-sided) and a power/MDE = 0.8 were used (statistical testing method: 2-sample t-test), estimating that a sample size of at least 800 participants with complete data at baseline and follow-up is aimed for to detect behavioural changes.

### The ToyBox-study: SB-component

For the structural planning of the ToyBox-intervention, six steps were systematically followed using the Intervention Mapping Protocol as a guide and scientific basis [24]. Information about the systematic development of the SB-component of the ToyBox-intervention can be found elsewhere [25]. The ToyBox-intervention was implemented during one school year, and was conducted at four levels which aimed to improve children’s social and physical environment regarding the targeted EBRBs. At level 1, teachers were asked in the beginning of the school year to conduct environmental changes in the classroom/kindergarten (e.g. create free space to increase children’s free movement), which were retained until the end of the school year. At level 2, teachers were asked to promote the actual EBRBs in the classroom/kindergarten on a regular basis and on a predefined time within each day (e.g. do short movement breaks twice in the morning and twice in the afternoon). At level 3, teachers implemented classroom activities, aiming total class participation, for at least one hour per day. At level 4, parents/caregivers received newsletters, tip-cards and posters which included simple advice on how to implement similar environmental changes at home, act as role models and perform the targeted EBRBs with their children. Regarding level 3, first, each of the four behaviours was subsequently targeted for four weeks (i.e. first focus period). Thereafter, each behaviour was targeted again for two weeks (i.e. repetition period). During week 13 until 16, the intervention focused for the first time on the SB-component. During the last two weeks of the intervention (week 23 and 24), the SB-component was targeted for the second time [23] [25].

The ToyBox-intervention was mainly implemented through kindergarten teachers, who received three training sessions of minimum one hour per session [26]. The first training session was implemented prior to the first focus period, the second training session was implemented immediately after the completion of the first four weeks of the intervention implementation, and the third training session was implemented prior to the repetition period. Before the start of the intervention, the teachers received the “ToyBox”, in which newsletters, tip cards, posters, a hand puppet and five handbooks could be found (i.e. one Teachers’ General Guide and four Classroom Activity Guides, one for each intervention component). The Teachers’ General Guide contained background information about the ToyBox-intervention and an overview of the time plan and procedures to be followed during the intervention period. The Classroom Activity Guide for the SB-component included three themes: (a) how to set environmental changes in the classroom (i.e. how to rearrange the classroom in order to reduce SB, e.g. by providing a standing play corner), which were implemented throughout the whole school year, (b) how to let the child perform the actual behaviour (e.g. implementing short movement breaks), and (c) classroom activities (e.g. stories on limiting SB, longer movement breaks, etc.). Teachers were asked to apply the environmental changes and perform the actual behaviour with the children throughout the school year and to implement several classroom activities of those listed in the Classroom Activity Guide for SB. To involve also parents/primary caregivers, two newsletters, two tip-cards and one poster on SB were taken home by the preschoolers. These materials contained key messages, tips and strategies for the parents/primary caregivers to limit the SB of their child. All materials can be found on the ToyBox-website (www.toybox-study.eu).

### Measures

The Primary Caregivers’ Questionnaire (PCQ) was designed to be filled out at home by the parents/primary caregivers and covers several components (i.e. socio-demographic factors, lifestyle behaviours and perinatal factors). In the present study, relevant socio-demographic measures (i.e. education level of the mother, used as a proxy measure of family SES [27]) and measures of preschoolers’ SB (i.e. TV/DVD/video viewing, computer/video games use and quiet play) will be reported. Education level of the mother was asked on a 5-point Likert-type scale, ranging from “less than 7 years” to “more than 16 years”, and was afterwards dichotomized into lower (14 or fewer years of education) or high (more than 14 years of education) education level, for the ease of interpretation [28]. The amount of time that children spent on TV/DVD/video viewing, computer/video games use and quiet play on weekdays and weekend days was assessed in three questions of the PCQ (one for each SB-subcomponent, i.e. TV-viewing, computer/video games use and quiet play). For example, “How much time does your child spend on TV-viewing (a) on weekdays, and (b) on weekend days?”. Possible answer options varied on a 9-point Likert-type scale, ranging from “never” to “more than 8 hours/day”. Additionally, a 10th answer option was “I don’t know”. Afterwards, these categorical values were recoded into numerical values according to the midpoint method (e.g. 3–4 hours/day was recoded into 3.5 hours/day) [7]. Numerical values were calculated separately for weekdays and weekend days. The PCQ was pre-tested and found to be a valid and reliable tool to assess preschool children’s SB [29]. To objectively estimate PA levels, total daily step counts were derived from motion sensors (i.e. pedometers or accelerometers) [30].

### Process evaluation

To evaluate the process of the intervention, process evaluation tools were developed based on the recommendations of Saunders, Evans & Joshi [31] [32] who described five important key elements for conducting a process evaluation: (1) reach (participation level in the intervention), (2) fidelity (quality of intervention implementation), (3) dose delivered (level in which the intervention was delivered), (4) dose received—exposure (active participation level and level of use of the materials and resources), and (5) dose received–satisfaction (satisfaction level of the implementers) [32]. In the present study, the key element “reach” was not included, as there were no data available on this dimension.

### Teachers

To calculate the process evaluation scores (PES) for the teachers, teachers received monthly logbooks with questions (e.g. use and feedback on the intervention materials, changes made in the environment, preschoolers performing classroom activities, etc.). Two of these logbooks contained specific questions regarding the implementation of the SB-component in the classroom. These specific questions (at least one question per key element), information about the scoring, and descriptive results can be found in Table 1. The scores of all 24 questions (each question with a score of 0 or 1) were summed to calculate a PES for each teacher. A higher teachers’ PES represents a better implementation of the SB-component of the intervention. A PES was calculated for each intervention kindergarten, by calculating the mean PES of all teachers within that kindergarten. Then, based on all kindergartens’ PES, tertiles were created dividing intervention kindergartens into three groups of equal size: kindergartens with a low, medium or high teachers’ PES. The control group was added as a forth group.

**Table 1:** Overview process evaluation questions and scoring, to calculate the PES for teachers (score on 24).

TEACHERS			
**First focus period**			
**Fidelity**	**Dose delivered**	**Dose received–exposure**	**Dose received—satisfaction**
1. *“Did you deliver the first sedentary behaviour* *newsletter* *to the parents*?*”* **▪ 1 = delivered (75.7%)** **▪ 0 = not delivered**	5. *“Was the classroom appropriately arranged for movement breaks every day of the week*?*”* (assessed on a 5-point scale) **▪ 1 = often or always (61.7%)** **▪ 0 = other answers**	8. *“Did you implement the classroom activities as described in the manual for sedentary behaviour*?*”* **▪ 1 = often or always (49.8%)** **▪ 0 = other answers**	Following items were assessed on a 5-point scale **▪ 1 = agree or totally agree** **▪ 0 = other answers** 9. *“It was easy to read and understand the text in the Classroom Activity Guide for sedentary behaviour”* **(1 = 68.0%)** 10. *“The amount of information and activities in the Classroom Activity Guide for sedentary behaviour were appropriate”* **(1 = 53.0%)** 11. *“It was easy to implement the activities described in the Classroom Activity Guide for sedentary behaviour”* **(1 = 60.7%)** 12. *“I enjoyed the activities I delivered this month”* **(1 = 65.2%)** 13. *“The activities I delivered this month were enjoyed by the children”* **(1 = 73.3%)** 14. *“The information presented in the Classroom Activity Guide for sedentary behaviour*, *the content of the material and the way the activities should be delivered are appropriate to achieve the goals”* **(1 = 61.7%)**
2. *“Did you deliver the first sedentary behaviour* *tip card* *to the parents*?*”* **▪ 1 = delivered (75.7%)** **▪ 0 = not delivered**	6. *“Did you devote on average at least one hour per week in the classroom activities as described in the manual for sedentary behaviour*?*”* (assessed on a 5-point scale) **▪ 1 = often or always (60.2%)** **▪ 0 = other answers**		
3. *“Did you deliver the sedentary behaviour* *poster* *to the parents*?*”* **▪ 1 = delivered (72.4%)** **▪ 0 = not delivered**	7. Sum score of 26 items related to implementation of classroom activities for SB (3 kangaroo stories, 8 classroom activities, 11 movement corners and 4 excursions) **▪ 1 = mostly implemented (9.1%)** **▪ 0 = mostly not implemented**		
4. *“All planned activities were performed*.*”* (assessed on a 5-point scale) **▪ 1 = agree or totally agree (47.2%)** **▪ 0 = other answers**			
**Repetition period**			
**Fidelity**	**Dose delivered**	**Dose received–exposure**	**Dose received—satisfaction**
15. *“Did you deliver the second sedentary behaviour* *newsletter* *to the parents*?*”* **▪ 1 = delivered (61.1%)** **▪ 0 = not delivered**	18. *“Was equipment and space appropriately arranged for movement breaks every day of the week*?*”* **▪ 1 = often or always (50.9%)** **▪ 0 = other answers**	21. *“Did you implement the classroom activities as described in the manual for sedentary behaviour*?*”* **▪ 1 = often or always (37.2%)** **▪ 0 = other answers**	Following items were assessed on a 5-point-scale **▪ 1 = agree or totally agree** **▪ 0 = other answers**
			22. *“It was easy to implement the activities described in the Classroom Activity Guide for sedentary behaviour”* **(1 = 48.0%)**
16. *“Did you deliver the second sedentary behaviour* *tip card* *to the parents*?*”* **▪ 1 = delivered (60.9%)** **▪ 0 = not delivered**	19. *“Did you devote on average at least one hour per week to the classroom activities as described in the manual for sedentary behaviour*?*”* **▪ 1 = often or always (46.5%)** **▪ 0 = other answers**		23. *“I enjoyed the activities I delivered this month”* **(1 = 54.8%)**
17. *“All planned activities were performed*.*”* (assessed on a 5-point scale) **▪ 1 = agree or totally agree (34.1%)** **▪ 0 = other answers**	20. Sum score of 26 items related to implementation classroom activities for SB (implementation of 3 kangaroo stories, 8 classroom activities, 11 movement corners and 4 excursions) **▪ 1 = mostly implemented (8.7%)** **▪ 0 = mostly not implemented**		24. *“The activities I delivered this month were enjoyed by the children”* **(1 = 59.8%)**
**Mean score (5.1/7)**	**Mean score (2.8/6)**	**Mean score (1.0/2)**	**Mean score (6.5/9)**
**Range (0–7)**	**Range (0–6)**	**Range (0–2)**	**Range (0–9)**

#### Parents/primary caregivers

After the intervention, also parents/primary caregivers were asked to fill out a questionnaire in which the intervention process was evaluated. These questions (at least one question per key element), information about the scoring, and descriptive results can be found in Table 2. The scores of all 17 questions (each question with a score of 0 or 1) were summed to calculate a PES for each parent/primary caregiver. A higher parents’ PES represents a better implementation of the SB-component of the intervention. Based on all parents’ PES, tertiles were created dividing preschoolers from the intervention group into three groups of equal size: preschoolers with a low, medium or high parents’ PES. The control group was added as a fourth group.

**Table 2:** Overview process evaluation questions and scoring, to calculate the PES for parents (score on 17).

PARENTS		
Dose delivered	Dose received–exposure	Dose received—satisfaction
1–5 *“Did you or your partner* *receive* *the materials regarding sedentary behaviour*?*”* (one score for each component: newsletter 1, newsletter 2, tip card 1, tip card 2, poster) **▪ 1 = yes** - Newsletter 1 (47.4%) - Newsletter 2 (45.7%) - Tip card 1 (42.4%) - Tip card 2 (40%) - Poster (33.8%) **▪ 0 = no or I don’t know**	11. *“Did you implement as a family the suggested activities of the ToyBox newsletters and tip cards*?*”* **▪ 1 = often or always (28.2%)** **▪ 0 = other answers**	12. *“In general*, *how easy was it to understand the text in the ToyBox newsletters and tip cards*?*”* **▪ 1 = easy or very easy (77.4%)** **▪ 0 = other answers** 13. *“In general*, *did you find the information provided in the ToyBox newsletters and tip cards trustful*?*”* **▪ 1 = to some degree or to a large degree (74.8%)** **▪ 0 = other answers** 14. *In general*, *how useful did you find the suggestions and tips for parents in the ToyBox newsletters and tip cards*?*”* **▪ 1 = somewhat useful or very useful (69.0%)** **▪ 0 = other answers** 15. *“Did you*, *your partner and your child enjoy the ToyBox activities conducted with the family*?*”* **▪ 1 = enjoyed it (a lot) (51.1%)** **▪ 0 = other answers** 16. *“In general*, *what did you think about the amount of text in the ToyBox newsletters and tip cards*?*”* **▪ 1 = about right (62.1%)** **▪ 0 = other answers** 17. *“In general*, *what did you think of the design (colours*, *lay out*, *type of letters) of the ToyBox newsletters and tip cards*?*”* **▪ 1 = liked it (a lot) (72.7%)** **▪ 0 = other answers**
6–10 *“Did you or your partner* *read* *the materials regarding sedentary behaviour*?*”* (one score for each component: newsletter 1, newsletter 2, tip card 1, tip card 2, poster) **▪ 1 = yes** - Newsletter 1 (40.6%) - Newsletter 2 (39.2%) - Tip card 1 (36.4%) - Tip card 2 (35.1%) - Poster (27.1%) **▪ 0 = no or I don’t know** ▪		
**Mean score (4.6/10)**	**Mean score (0.3/1)**	**Mean score (4.7/6)**
**Range (0–10)**	**Range (0–1)**	**Range (0–6)**

### Statistical analyses

All preschoolers whose parents/primary caregivers filled out less than 75% of the total PCQ, who had incomplete data on one of the SB outcome variables or who had no PA data were excluded from the dataset (n = 3095). Descriptive statistics were computed to describe the sample characteristics, using SPSS statistics version 22.0 for Windows. To assess the potential effect of the intervention on all the outcome variables, to describe differences between countries, and to control for clustering of preschoolers in classes in kindergartens, multilevel repeated measures analyses were conducted using MLwiN 2.31 (Centre for Multilevel Modelling, University of Bristol, UK) with five levels: time, preschooler, class, kindergarten and country. All analyses were adjusted for age, sex, education level of the mother and PA (step counts). To look for differences between preschool children who were excluded with those who were not excluded, attrition analyses were conducted as a logistic regression with three levels (child, class and kindergarten).

The main effects of Time and the two-way interaction effects of Time x Group were considered for the total sample. Additionally, differences between countries were examined by adding the three-way interaction of Time x Group x Country in the model. When this three-way interaction effect was significant for any of the SB-outcome variables, the multilevel analyses were repeated for each country separately.

As an effect size, the proportion of variance explained by the interaction-variable Time x Group in addition to the other variables was calculated (η_p_^2^). An effect of 0.02 (2% explained variance) or lower is considered as small, an effect between 0.12–0.25 (12–25% explained variance) is considered as medium and an effect of 0.26 (26% explained variance) or higher is considered as large. The effect sizes were calculated using the following formula: total variance of the model without the interaction term minus the total variance of the full model, divided by the total variance of the model without the interaction term (i.e. Model_MinusInteraction_—Model_Full_ / Model_MinusInteraction_). This number was then multiplied by 100 to convert it to a percentage. Effect sizes are only reported in the text (in percentages) and not in the tables.

Finally, to study the effect of the process evaluation scores on preschoolers’ SB, multilevel repeated measures analyses were performed with five levels (time, preschooler, class, kindergarten and country), and Time x Group interaction effects were investigated. For all analyses, statistical significance level was set on p<0.05. All effects with a p-value between 0.05 and 0.10 were seen as borderline significant.

## Results

### Descriptives

In total, 2434 parents/primary caregivers of preschool children (52.2% boys; mean age: 4.7±0.4 years) across six European countries provided valid data at baseline and follow-up (n_intervention group_ = 1642; n_control group_ = 792). Attrition analyses showed no significant differences in age, sex and group of preschoolers who were excluded and those who were not excluded (OR_age_ = 1.04; 95% CI_age_ = 0.93, 1.16; OR_sex_ = 1.03; 95% CI_sex_ = 0.92, 1.14; OR_excluded_ = 0.22; 95% CI_group_ = 0.91, 1.52). Descriptive statistics of the sample can be found in Table 3. The CONSORT flow diagram for participants throughout the study can be found in Fig 1.

**Table 3:** Descriptive statistics of preschool children with complete data across the six intervention countries.

	Total	Belgium	Bulgaria	Germany	Greece	Poland	Spain
*n*	2434	528	73	294	356	723	460
*Mean age (years)*	4.7	4.4	4.9	4.5	4.8	4.8	4.8
*Boys (%)*	52.2	51.9	57.5	52.7	51.1	51.3	53.7
*SES (% lower SES)*	33.7	31.6	30.1	55.8	48.6	18.4	35.0
*PA (mean steps)*							
• *weekday* • *weekend day*	• 10.443 • 9.524	• 10.162 • 8.350	7.727 8.831	• 10.848 • 9.822	• 8.686 • 8.241	• 10.565 • 10.590	• 12.054 9.955

### Intervention effects

#### Total sample

Results for the SB outcomes are shown in Table 4. Compared to the control group, preschoolers from the intervention group showed a smaller increase from baseline to follow-up in computer/video games use on weekdays (ß = -3.40, p = 0.06; control group: +8.89 min/day; intervention group: +5.48 min/day; η_p_^2^ = 0.10) and on weekend days (ß = -5.97, p = 0.05; control group: +15.43 min/day; intervention group: +9.46 min/day; η_p_^2^ = 0.12). However, these effects were only borderline significant. No other significant interaction effects were found in the total sample.

**Table 4:** Time and interaction effects for SB-outcomes for the total sample.

n = 2434 (I = 1642, C = 792)	Pre-test (min/day)	Post-test (min/day)	Time (β)	Time*Group (β)
**TV/DVD/video viewing**				
Weekday				
*Intervention*	78.44	77.34	-1.97	0.87
*Control*	77.49	75.51		
Weekend day				
*Intervention*	124.40	124.40	0.99	-1.00
*Control*	120.85	121.84		
**Computer/video games use**				
Weekday				
*Intervention*	17.05	22.53	8.88***	-3.40*
*Control*	14.57	23.46		
Weekend day				
*Intervention*	33.84	43.30	15.44***	-5.97*
*Control*	31.12	46.55		
**Quiet play**				
Weekday				
*Intervention*	76.92	72.56	-6.65	2.29
*Control*	78.39	71.74		
Weekend day				
*Intervention*	116.65	106.92	-12.24**	2.51
*Control*	118.50	106.26		

*p<0.10

**p<0.05

***p< .001

All analyses were adjusted for age, sex, education level of the mother, and PA on weekdays and weekend days.

#### Effects per country

A significant three-way interaction effect of Time x Group x Country was found for computer/video games use on weekend days, meaning that significant differences in intervention effects between the countries could be observed. Results obtained from the multilevel repeated measures analyses for all SB outcomes in each country are shown in Table 5. For Belgium, results showed that there was a smaller increase in computer/video games use on weekend days in the intervention group, compared to the control group (ß = -7.77, p = 0.02; control group: +18.08 min/day, intervention group: +10.31 min/day; η_p_^2^ = 0.29) from baseline to follow-up. For Bulgaria, results showed that preschoolers from the intervention group decreased in computer/video games use on weekend days from baseline to follow-up, whereas preschoolers from the control group showed an increase in computer/video games use on weekend days from baseline to follow-up (ß = -37.96, p = 0.01; control group: +21.89 min/day, intervention group: -16.07 min/day; η_p_^2^ = 2.99). No significant intervention effects were found for Poland, Spain, Greece and Germany.

**Table 5:** Time and interaction effects for SB-outcomes per country.

	Pre-test (min/day)	Post-test (min/day)	Time (β)	Time*Group (β)
**Belgium (n = 528, I = 330, C = 198)**				
**TV/DVD/video viewing**				
Weekday				
*Intervention*	69.19	68.10	-0.35	-0.74
*Control*	74.57	74.21		
Weekend day				
*Intervention*	113.43	114.08	3.62	-2.97
*Control*	122.33	125.95		
**Computer/video games use**				
Weekday				
*Intervention*	10.51	16.31	10.98***	-5.18
*Control*	11.06	22.04		
Weekend day				
*Intervention*	22.97	33.28	18.09***	-7.77*
*Control*	22.87	40.95		
**Quiet play**				
Weekday				
*Intervention*	73.60	71.16	-6.44	4.00
*Control*	82.74	76.30		
Weekend day				
*Intervention*	155.00	137.78	-17.78	-0.56
*Control*	165.88	148.10		
**Bulgaria (n = 73, I = 35, C = 38)**				
**TV/DVD/video viewing**				
Weekday				
*Intervention*	81.77	82.30	7.70	-7.17
*Control*	91.57	99.27		
Weekend day				
*Intervention*	123.09	128.20	11.80	-6.67
*Control*	138.79	150.59		
**Computer/video games use**				
Weekday				
*Intervention*	38.66	36.05	9.11	-11.72
*Control*	23.46	32.57		
Weekend day				
*Intervention*	79.44	63.37	21.89**	-37.96**
*Control*	39.33	61.22		
**Quiet Play**				
Weekday				
*Intervention*	52.46	44.42	-3.24	-4.79
*Control*	58.84	55.60		
Weekend day				
*Intervention*	93.38	82.67	-5.68	-5.04
*Control*	78.71	73.04		
**Germany (n = 294, I = 242, C = 52)**				
**TV/DVD/video viewing**				
Weekday				
*Intervention*	43.97	43.79	-0.97	0.78
*Control*	35.79	34.82		
Weekend day				
*Intervention*	70.14	72.80	5.37	-2.71
*Control*	60.39	65.76		
**Computer/video games use**				
Weekday				
*Intervention*	7.72	12.41	5.94	-1.25
*Control*	9.98	15.92		
Weekend day				
*Intervention*	13.43	18.69	9.05	-3.78
*Control*	13.99	23.04		
**Quiet Play**				
Weekday				
*Intervention*	82.73	76.40	-5.76	-0.59
*Control*	104.85	99.12		
Weekend day				
*Intervention*	106.86	103.66	-1.77	-1.44
*Control*	111.24	109.48		
**Greece (n = 356, I = 453, C = 270)**				
**TV/DVD/video viewing**				
Weekday				
*Intervention*	92.70	91.09	5.90	-7.51
*Control*	81.07	86.97		
Weekend day				
*Intervention*	137.61	141.62	-0.76	4.76
*Control*	136.07	135.31		
**Computer/video games use**				
Weekday				
*Intervention*	12.11	18.56	9.60**	-3.68
*Control*	16.78	26.39		
Weekend day				
*Intervention*	31.41	39.25	13.06**	-5.22
*Control*	38.40	51.46		
**Quiet Play**				
Weekday				
*Intervention*	73.63	73.72	-8.17	8.27
*Control*	66.59	58.42		
Weekend day				
*Intervention*	99.16	96.45	-16.75	14.04
*Control*	95.16	78.41		
**Poland (n = 723, I = 453, C = 270)**				
**TV/DVD/video viewing**				
Weekday				
*Intervention*	96.28	92.78	-4.71	1.22
*Control*	92.87	88.15		
Weekend day				
*Intervention*	142.08	139.01	-0.53	-2.54
*Control*	133.26	132.73		
**Computer/video games use**				
Weekday				
*Intervention*	27.60	33.85	10.10***	-3.85
*Control*	23.47	33.57		
Weekend day				
*Intervention*	45.62	57.99	15.07***	-2.70
*Control*	41.17	56.24		
**Quiet Play**				
Weekday				
*Intervention*	104.99	97.40	-9.97	2.38
*Control*	104.79	94.82		
Weekend day				
*Intervention*	138.08	127.19	-9.15	-1.73
*Control*	140.67	131.52		
**Spain (n = 460, I = 323, C = 137)**				
**TV/DVD/video viewing**				
Weekday				
*Intervention*	75.35	76.70	-6.19	7.54
*Control*	81.50	75.31		
Weekend day				
*Intervention*	144.41	142.08	-0.93	-1.40
*Control*	131.50	130.57		
**Computer/video games use**				
Weekday				
*Intervention*	12.93	17.53	4.36	0.26
*Control*	8.93	13.30		
Weekend day				
*Intervention*	34.53	45.78	14.81**	-3.57
*Control*	34.18	48.99		
**Quiet Play**				
Weekday				
*Intervention*	58.57	54.87	-1.24	-2.36
*Control*	56.52	55.16		
Weekend day				
*Intervention*	101.69	92.38	-12.97	3.27
*Control*	98.42	85.45		

*p<0.10

**p<0.05

***p< .001

In all analyses, there was adjusted for age, sex, education level of the mother, and PA on weekdays and weekend days.

### Results of the teachers’ PES

Based on all the individual teachers’ PES (n = 388), a mean PES per kindergarten was calculated (mean teachers with a PES per kindergarten: 4.4, range: 0–13). This could be done for 89 of the 96 intervention kindergartens. The mean teachers’ PES for all kindergartens participating in the intervention was 15.58 (±5.53) on a total score of 24. Based on the teachers’ PES, kindergartens were divided into three groups of approximately equal size: (1) kindergartens with a low teachers’ PES (score≤13, mean: 9.25, SD: 3.502, n_kindergartens_ = 29, n_preschoolers_ = 545), (2) kindergartens with a medium teachers’ PES (score 13–19.30, mean: 16.60, SD: 1.90, n_kindergartens_ = 31, n_preschoolers_ = 526), and (3) kindergartens with a high teachers’ PES (score>19.30, mean: 21.26, SD: 1.34, n_kindergartens_ = 29, n_preschoolers_ = 514).

#### TV/DVD/video viewing

For TV/DVD/video viewing on weekdays and weekend day, no significant interaction effects were found.

#### Computer/video games use

**Fig 2:**
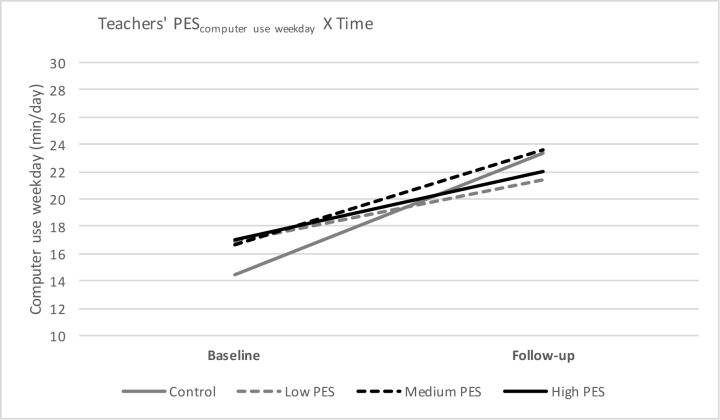
Teachers’ PES for computer/video games use on weekdays from baseline to follow-up.

**Fig 3:**
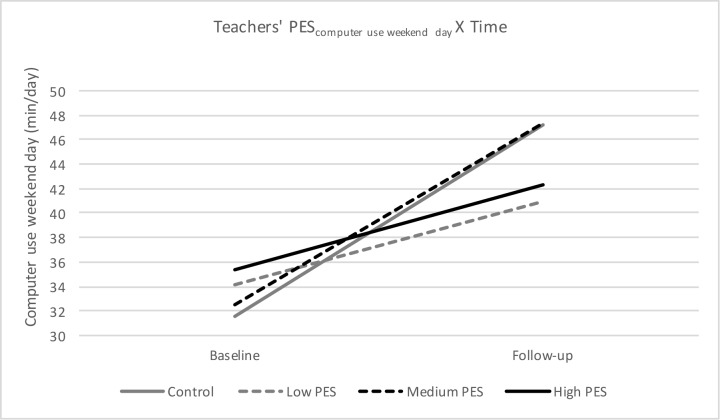
Teachers’ PES for computer/video games use on weekend days from baseline to follow-up.

For both computer/video games use on weekdays and weekend day, a significant interaction effect of PES-group X Time was found, meaning that there is a significant difference in computer/video games use on weekdays and on weekend days from baseline to follow-up depending on the PES-group preschoolers belonged to (Figs 2 and 3). More specific, there was a difference between the control group and preschool children from kindergartens with a low teachers’ PES (β = 4.43, p = 0.007), and between the control group and preschool children from kindergartens with a high teachers’ PES (β = 3.81; p = 0.026). Furthermore, there was also a difference between preschool children from kindergartens with a medium teachers’ PES and preschool children from kindergartens with low teachers’ PES (β = 8.01; p = 0.013) and between preschool children from kindergartens with a medium teachers’ PES and preschool children from kindergartens with a high teachers’ PES (β = 7.74; p = 0.018). Preschoolers from kindergartens with a low teachers’ PES and a high teachers’ PES had a significant smaller increase in computer/video games use on weekdays (low teachers’ PES: +4.47 min/day, p<0.001; high teachers’ PES: +5.09 min/day, p<0.001) and weekend day (low teachers’ PES: +6.74 min/day, p = 0.002; high teachers’ PES: +7.00 min/day, p = 0.004), compared to the control group (weekday: +8.90 min/day, p<0.001; weekend day: +15.66 min/day, p<0.001) and compared to the preschoolers with a medium teachers’ PES (weekend day: +14.75 min/day, p = 0.003) No other significant interaction effects were found.

#### Quiet play

For quiet play on weekdays and weekend day, no significant interaction effects were found.

### Results of the parents’ PES

A parents’ PES could be calculated for 1475 of the 1642 preschoolers. The mean parents’ PES for these preschoolers was 8.67 (±5.50) on a total score of 17. Based on the parents’ PES, preschoolers were divided into three groups of approximately equal size, based on their parents’ PES: (1) preschoolers with a low parents’ PES (score ≤5, mean: 2.52, SD: 2.09, n = 494), (2) preschoolers with a medium parents’ PES (score 6–12, mean: 8.60, SD: 2.14, n = 524), and (3) preschoolers with a high parents’ PES (score >12, mean: 15.39, SD:1.26, n = 457).

### TV/DVD/video viewing

For TV/DVD/video viewing on weekdays and weekend day, no significant interaction effects were found.

### Computer/video games use

**Fig 4:**
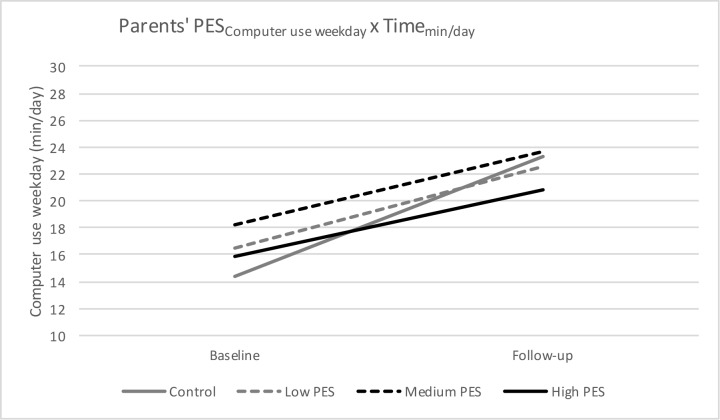
Parents’ PES for computer/video games use on weekdays from baseline to follow-up.

**Fig 5:**
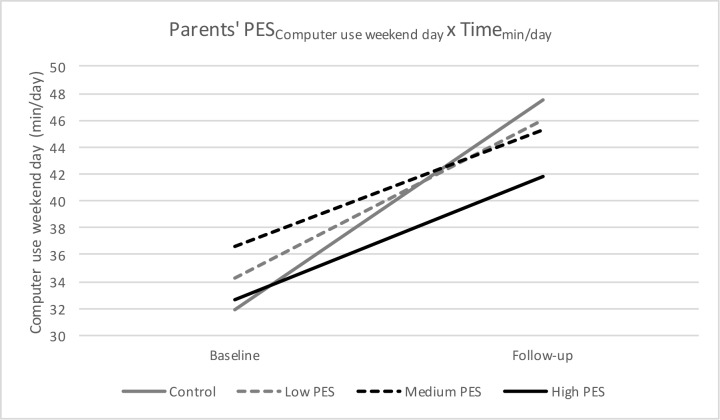
Parents’ PES for computer/video games use on weekend days from baseline to follow-up.

For both computer/video games use on weekdays and weekend day, a significant interaction effect of PES-group X Time was found, meaning that there is a significant difference in computer/video games use on weekdays and on weekend days from baseline to follow-up depending on the PES-group preschoolers belonged to (Figs 4 and 5). More specific, there was a difference between the control group and preschool children with a medium parents’ PES (β = 3.51, p = 0.04), and between the control group and preschool children with a high parents’ PES β = 4.03; p = 0.02). Preschoolers with a medium parents’ PES and a high parents’ PES had a significant smaller increase in computer/video games use on weekdays (medium parents’ PES: +5.39 min/day, p = 0.02; high parents’ PES: +4.87 min/day, p<0.001) and weekend day (medium parents’ PES: +8.57 min/day, p<0.001; high parents’ PES: +9.16 min/day, p<0.001), compared to the control group (weekday: +8.90 min/day, p<0.001; weekend day: +15.66 min/day, p<0.001). No other significant interaction effects were found (all p>0.05).

#### Quiet play

For quiet play on weekdays and weekend day, no significant interaction effects were found.

## Discussion

The aim of the present study was twofold. The first aim was to investigate the effect of the ToyBox-intervention on SB in 4- to 6-year-old preschoolers in six European countries (Belgium, Bulgaria, Germany, Greece, Poland and Spain). The second aim of the study was to investigate whether a better PES was related to a more beneficial intervention effect on preschoolers’ SB. Both teachers and parents/primary caregivers were involved in the intervention, using both kindergarten-based and family-involved components in order to decrease preschoolers’ SB.

In the total sample, a borderline significant intervention effect was found for computer/video games use on week- and weekend days. Both the intervention group and the control group showed an increase in computer/video games use on week- and weekend days over time, but the increase in the intervention group was smaller than the increase in the control group. Similar effects were found in the Belgian sample for computer/video games use on weekend days. For Bulgaria, a decrease was found in computer/video games use on weekend days in the intervention group, and an increase in the control group. Although these intervention effects are statistically significant, the biological relevance should be interpreted with caution because the effect sizes are small. However, taking into account that SB normally increases with age [33], these effects (i.e. the smaller increase in Belgium and the little decrease in Bulgaria) may become biologically more relevant at a later age, if the trend persists.

To our knowledge, the ToyBox-intervention is the first intervention that was able to limit the age-related increase in preschoolers’ computer/video games use on week- and weekend days. In contrast to the family-based interventions of O’Dwyer et al. [11] and Dennison et al. [10], the ToyBox-intervention did not lead to a reduction in TV/DVD/video viewing. This might be attributed to the fact that in these interventions parents were more actively involved. This could also explain why the school-based intervention of O’Dwyer et al. [12] was not effective in reducing sedentary time in preschoolers, as parents were not involved in this intervention. In sum, these findings could mean that school-based interventions in which parents are actively engaged are more effective [11, 34]. However, the positive findings in the studies of O’Dwyer et al. [11] and Dennison et al. [10] can also be due to the fact that in these studies diary reports instead of questionnaires were used to measure SB, which are a more accurate measure to report behaviour as the events are reported on the day they occur, and not retrospectively [35]. Another reason for the fact that no reduction in TV/DVD/video viewing was found, might be the fact that parents still do not realize that TV/DVD/video viewing should be limited, as in a qualitative study of Jordan, Hersey, McDivitt and Heitzler [36] interviews revealed that for many families there is a lack of concern that long periods of TV/DVD/video viewing is a problem for their child. Furthermore, in the focus groups conducted within the ToyBox-study, parents indicated that they consider TV viewing as e good educational tool for their children [37].

Furthermore, a remarkable finding in the present study is that both the intervention group and control group show a stagnation in TV/DVD/video viewing over time. This might be due to the fact that, for TV/DVD/video viewing, the ToyBox-intervention was not strong enough to cause an effect that is larger than the effect of an increased awareness (e.g. through filling out the pre-test questionnaires in which the concept ‘sedentary behaviour’ was mentioned).

A possible reason why the intervention was not able to cause a decrease in preschoolers’ quiet play can be due to the fact that parents and teachers still -despite the fact that this information was provided through the intervention materials- do not fully understand what ‘quiet play’ exactly is, because they did not receive and/or read the intervention materials, or because sitting still is seen as an important aspect in education. Consequently, parents and teachers probably did not focus enough on limiting this behaviour in their preschooler during the intervention, leading to similar effects in the control group and the intervention group. Furthermore, this type of SB may be less important to target as it may be more educational than prolonged screen use.

The process evaluation data of the teachers showed that the quality of the intervention was relatively good, as the score on the dimension “fidelity” was relatively high, meaning that teachers delivered the materials (i.e. newsletters, tip cards and poster) to the parents as requested. An important weakness on this dimension was that not all planned activities were performed by the teachers, in both the first focus period and the repetition period. Also the score on the dimension “dose received-satisfaction” was relatively high, meaning that the teachers were generally satisfied about the SB-component of the intervention, especially in the first focus period. In the repetition period, the level of satisfaction of the teachers decreased, as they indicated that it was no longer easy to implement the activities described in the Classroom Activity Guide for SB and teachers did not longer enjoy the activities they delivered.

Scores on the dimension “dose delivered” and “dose received-exposure” were generally lower. The first was mostly due to the fact that most of the classroom activities for SB (e.g. excursions) were not implemented by the teachers. This finding is also in line with the abovementioned weakness on the dimension “fidelity” (i.e. that not all planned activities were performed by the teachers). As the teachers were generally satisfied about the intervention, these findings could mean that the planned (classroom) activities were too demanding for the teachers. This could also explain why the scores on the other aspects of the “dose delivered” dimension (i.e. appropriately rearranging the classroom for movement breaks and devoting at least one hour per week on classroom activities) are higher, as these aspects are less demanding and easier to implement. These results stress the importance of using activities in an intervention that are easy to implement and not very time consuming for the teachers (e.g. short movement breaks, short stories, etc.). The lower score on the dimension “dose received-exposure”, which was mostly due to lower scores in the repetition period, could mean that teachers lost their interest and/or motivation in the intervention over time which consequently led to a less active participation level in the intervention and a decrease in use and implementation of the activities. Facilitating teachers’ efforts and keeping them motivated throughout the intervention is therefore strongly recommended for future interventions. Important factors influencing the latter are for example support of the school principal, giving structured and corrective feedback to the teachers, teachers’ self-efficacy and teachers’ beliefs [38].

Considering the process evaluation data of the parents, the data showed a relative high score on the dimension “dose received-satisfaction”, meaning that parents were generally satisfied about the (materials used in the) SB-component of the intervention. However, half of the parents indicated that they and their children did not enjoy the ToyBox-activities regarding SB. This could be due to the fact that these activities included a restriction of TV/DVD/video viewing and computer/video games use, which could have been perceived as a ‘punishment’. A possible strategy to cope with this in the future might be to include homeworks which are novel, fun, and involve activities and social contact, as a study of Kipping, Jago and Lawlor [39] has shown that this is an effective method that is enjoyed by both parents and children. Furthermore, it should be remarked that parents’ beliefs and attitudes influence the beliefs and attitudes of their children [40, 41]. Therefore, it is likely that when the parents did not like the ToyBox-activities, the child did neither. Consequently, tailoring the intervention materials so that both the parents and children enjoy it should be taken into account for future interventions. This can be done, for example, by adapting a “co-participatory approach” in which the intervention and intervention materials are developed taking into account stakeholders needs, ideas, reality, etc. Finally, scores on the dimension “dose delivered” and “dose received-exposure” were generally low, as more than half of the parents reported that they did not receive and/or read the materials regarding SB, and they did not implement the suggested activities of the newsletters and tip cards as a family. This shows clearly that parents were not very motivated or had not much time or energy to implement the intervention. This issue can be addressed in the future by providing parents with information about the importance of commitment, by eliciting motivational statements about adhering to the intervention and by helping parents to identify and develop plans for overcoming barriers towards the intervention that may arise during the intervention [42].

Results investigating whether better teachers’ and parents’ PES were related to a more beneficial effect on preschoolers’ SB, showed that preschoolers with a low and a high teachers’ PES and preschoolers with a medium and a high parents’ PES had a smaller increase in computer/video games use on weekdays and weekend days, compared to the control group. This means that a better implementation by the parents is related to a more beneficial effect on preschoolers’ SB, while a better implementation by the teachers is not related to a more beneficial effect. Especially involving the home environment (i.e. parents/primary caregivers) seems therefore important in intervention studies focusing on preschoolers’ SB, which can also be supported by the literature [11, 10, 43–45, 41]. The main reason for this is that SBs in preschoolers (i.e. TV/DVD/video viewing, computer/video games use and quiet play) are behaviours that are mainly performed at home.

A possible reason why an effect on computer/video games use was found even in the group with a low teachers’ PES, can be that computer/video games use is a SB that is easy to limit because it is the least popular SB in preschoolers. This is also confirmed by our data (i.e. preschoolers watch more TV and spend more time in quiet play).

A first possible reason why the ToyBox-intervention did not cause larger effects, can be attributed to the relative low levels of intervention implementation and motivation of the parents and the teachers during the repetition period. A second possible reason may be due to the fact that the ToyBox-intervention is a standardized European intervention. This means that the ToyBox-intervention was generally the same in all countries with room for only small cultural and local adaptations. Although the randomized controlled trial is the golden standard in medical research [46], it might not be the most appropriate in health promotion research [47, 48]. Another possible reason why the ToyBox-intervention did not cause the expected effects can be because the intervention included only a small family component, meaning that parents were only passively involved in the intervention. As the SBs targeted in this intervention (i.e. TV/DVD/video viewing, computer/video games use and quiet play) mainly take place in the afternoon and on weekend days, the involvement of parents in reducing/limiting these SBs is thus very important. Furthermore, improving the intervention materials so that they are more enjoyed by the parents and children, and motivating the parents to actively participate in both the development and implementation of the intervention [11] might be a promising strategy. Results of a study of White, Taylor and Moss [43] indeed show that the involvement of parents in the development of an intervention can induce larger effects.

The lack of significant effects of the intervention in the other countries, i.e. Germany, Greece, Poland and Spain, can be due to the lack of statistical power in these country-specific subgroups. More specifically, it is likely that the absence of effects in these countries can be attributed to a relatively limited sample size.

Strengths of the current study are the large sample (n = 2434) of preschoolers that provided valid data, and the cluster randomized controlled trial with a pre-test post-test design. In addition, also the use of process evaluation questionnaires for both kindergarten teachers and parents/primary caregivers can be seen as a strength. The calculation of the process evaluation scores was theory-based, as key elements from the process evaluation model of Saunders et al. [32] were used. This method was also used elsewhere [49, 50]. However, it should be acknowledged that there is no standardized method to calculate process evaluation scores. This means that there are several ways to calculate these scores, depending on the focus and weight that is given to each of the key elements.

Study limitations include the parental report of the outcome variables (i.e. TV/DVD/video viewing, computer/video games use and quiet play). Parental report is a subjective proxy-measure that possibly can be biased. However, the questionnaire used in the present study was pre-tested and the results showed that this questionnaire is a valid and reliable tool to assess preschool children’s SB [29]. Objective measures of SB, such as accelerometry, may overcome these validity issues in future research. However, this method cannot distinguished between several sub-components of SB (i.e. TV/DVD/video viewing, computer/video games use and quiet play). Ideal is the use of subjective and objective measures of SB in combination. Another limitation is that no data were collected regarding novel technologies such as Smartphones, iPads, etc., which may also influence the amount of SB in preschoolers.

## Conclusions

Overall, the ToyBox-intervention caused significant but small effects on European preschoolers’ sedentary behaviours. More specifically, intervention effects were found for computer/video games use on weekdays and weekend days in the total sample (i.e. a smaller increase over time for computer/video games use in the intervention group compared to the control group). For Belgian preschoolers, a similar intervention effect was found but only for weekend days. For Bulgarian preschoolers, a decrease in computer/video game use on weekend days was found in the intervention group, and an increase in the control group. No effects were found for the other countries. Process evaluation data of the sedentary-behaviour component of the intervention revealed low process evaluation scores for the parents and for the teachers during the second part of the intervention. Therefore, it is recommended for future interventions to search for different implementation strategies. More specifically, parents should be involved more actively in both the development and implementation of the intervention, and teachers should be provided with more practical, easy and less time consuming activities that limit sedentary behaviour. Furthermore, both parents and teachers should be frequently motivated, so that they continue in implementing and delivering the intervention in a qualitative way over time, also at later stages of the intervention, especially because the amount of sedentary behaviour in preschoolers increases with age.

## Supporting information

S1 Dataset (SAV)

S1 Table (DOC)

S1 Text (PDF)

S2 Text (PDF)

## Abbreviations

SB: Sedentary Behaviour
SES: Socio-Economic Status
PCQ: Primary Caregivers’ Questionnaire
PA: Physical Activity
PES: Process Evaluation Scores
