# Supplementary material for: Effect and process evaluation of a kindergarten-based, family-involved intervention with a randomized cluster design on sedentary behaviour in 4- to 6- year old European preschool children: The ToyBox-study
Source: PLoS One. 2017 Apr 5;12(4):e0172730. doi: 10.1371/journal.pone.0172730 (PMC5381762; doi:10.1371/journal.pone.0172730)
Supplement: S1 Text — (PDF) [file pone.0172730.s003.pdf]

ETHISCH COMITE

Universitair Ziekenhuis
De Pintelaan 185
9000 Gent
ethisch.comite@UGent.be
tel. +32 9 332 33 36 - +32 9 332 68 54 - +32 9 332 26 88
fax +32 9 332 49 62

Document A (interventioneel academisch onderzoek)

VERZOEK TOT ADVIES VAN HET ETHISCH COMITE BETREFFENDE EEN ONDERZOEKSPROJECT BIJ DE MENS

EUDRACT NUMMER (INDIEN INTERVENTIONEEL GENEESMIDDELENONDERZOEK):

1. TITEL VAN HET ONDERZOEK

MULTIFACTORIAL EVIDENCE BASED APPROACH USING BEHAVIOURAL MODELS IN UNDERSTANDING AND PROMOTING FUN, HEALTHY FOOD, PLAY AND POLICY FOR THE PREVENTION OF OBESITY IN EARLY CHILDHOOD

2. GEGEVENS VAN DE ONDERZOEKER(S) [de eerste onderzoeker moet een persoon zijn die vast verbonden is aan de dienst (geen ASO) of universiteit] :

- NAAM: ILSE DE BOURDEAUDHUIJ
- FUNCTIE: ZELFSTANDIG ACADEMISCH PERSONEEL
- UZ DIENST :
  OF FACULTEIT/VAKGROEP: GE25 BEWEGINGS- EN SPORTWETENSCHAPPEN
- TELEFOONNUMMER: 09/264.63.11
- FAX: 09/264.64.84
- E-MAIL: ILSE.DEBOURDEAUDHUIJ@UGENT.BE
- NAAM UZ DIENSTHOOFD:
  OF NAAM VAKGROEPVOORZITTER: PROF. DR. DIRK DE CLERCQ

- NAAM: GREET CARDON
- FUNCTIE: ZELFSTANDIG ACADEMISCH PERSONEEL
- UZ DIENST :
  OF FACULTEIT/VAKGROEP: GE25 BEWEGINGS- EN SPORTWETENSCHAPPEN
- TELEFOONNUMMER: 09/264.91.42
- FAX:
- E-MAIL: GREET.CARDON@UGENT.BE
- NAAM UZ DIENSTHOOFD:
  OF NAAM VAKGROEPVOORZITTER: PROF. DR. DIRK DE CLERCQ

- NAAM: ELLEN DE DECKER
- FUNCTIE: DOCTORAATSSTUDENT
- UZ DIENST :
  OF FACULTEIT/VAKGROEP: GE25 BEWEGINGS- EN SPORTWETENSCHAPPEN
- TELEFOONNUMMER: 09/264.86.83
- FAX:
- E-MAIL: ELLEN.DEDECKER@UGENT.BE
- NAAM UZ DIENSTHOOFD:
  OF NAAM VAKGROEPVOORZITTER: PROF. DR. DIRK DE CLERCQ

- NAAM: MARIEKE DE CRAEMER
- FUNCTIE: DOCTORAATSSTUDENT
- UZ DIENST :
  OF FACULTEIT/VAKGROEP: GE25 BEWEGINGS- EN SPORTWETENSCHAPPEN
- TELEFOONNUMMER: 09/264.86.83
- FAX:
- E-MAIL: MARIEKE.DECRAEMER@UGENT.BE

- NAAM UZ DIENSTHOOFD:
  OF NAAM VAKGROEPVOORZITTER: PROF. DR. DIRK DE CLERCQ

3. GEGEVENS VAN DE MEDEWERKER(S) AAN DE STUDIE:

- NAAM:
- FUNCTIE:
- UZ DIENST :
  OF FACULTEIT/VAKGROEP:
- TELEFOONNUMMER:
- FAX:
- E-MAIL:
- NAAM UZ DIENSTHOOFD:
  OF NAAM VAKGROEPVOORZITTER

4. SOORT ONDERZOEK:

☒ INTERVENTIONEEL ONDERZOEK (INTERVENTION)

☐ MET GENEESMIDDEL (ALLE ITEMS VAN TOEPASSING AANDUIDEN)
- ☐ FASE I
- ☐ FASE II
- ☐ FASE III
- ☐ FASE IV
- ☐ PROEF VOOR GENTHERAPIE EN SOMATISCHE CELTHERAPIE
- ☐ PROEF MET GENEESMIDDELEN DIE GENETISCH GEWIJZIGDE ORGANISMEN BEVATTEN
- ☐ PROEF MET CELTHERAPIE MET XENOGENEN

☒ ANDERE SPECIFICEER (VB MEDICAL DEVICE, BLOEDAFNAME, RX,…)
- ☐ MEDICAL DEVICE
- ☐ BLOEDAFNAME, RX,…
- ☒ OTHER: MULTIFACTORIAL PROJECT FOR KINDERGARTENS: EDUCATIONAL MATERIAL (MANUALS), NEWSLETTERS, TIP CARDS, HAND PUPPET

5. IS HET ONDERZOEK

☐ DIAGNOSTISCH
☐ FYSIOLOGISCH
☐ MORFOLOGISCH

☐ THERAPEUTISCH
☐ FYSIOPATHOLOGISCH
☒ EPIDEMIOLOGIC

6. IS HET ONDERZOEK IN BELGIË

☒ MONOCENTRIC

☐ MULTICENTRISCH

☐ HET ETHISCH COMITÉ UZ GENT IS "CENTRAAL" ETHISCH COMITÉ

☐ JA

- NAAM, ADRES, TEL., FAX EN E-MAIL VAN ANDERE ETHISCHE COMITÉ DIE MEEWERKEN AAN HET ONDERZOEK + NAAM VAN DE LOKALE ONDERZOEKER

☐ NEEN

- NAAM, ADRES, TEL, FAX EN E-MAIL VAN HET CENTRAAL ETHISCH COMITÉ

7. WILL THIS PROJECT TAKE PLACE IN OTHER COUNTRIES AS WELL?

☒ IN EUROPE - WHICH ARE THE PARTICIPATING COUNTRIES: BULGARIA, GERMANY, GREECE, POLAND AND SPAIN

☐ IN USA

8.	IS THIS STUDY RECEIVING FINANCIAL SUPPORT?

☒ YES ☐ NEEN

☐ FWO/BOF

☐ FARMACEUTISCHE INDUSTRIE:

☒ OTHER: 7TH FRAMEWORK PROGRAMME (THEME 2)

9.	WIE IS DE OPDRACHTGEVER VAN DE NIET INDUSTRIE GESPONSORDE STUDIE

☐ MEDEWERKER VAN HET UZ (NAAM EN ADRES)

☐ MEDEWERKER VAN DE UNIVERSITEIT GENT (NAAM EN ADRES)

☐ ANDERE, SPECIFIEER (NAAM EN ADRES)

10.	GEEF EEN KORTE SAMENVATTING VAN HET PROTOCOL (MINIMUM 30 ZINNEN/ EEN HALVE PAGINA EN MAXIMUM ÉÉN PAGINA), VERSTAANBAAR VOOR MENSEN NIET GESPECIALISEERD IN DE MATERIE, VERWIJS NIET ALLEEN NAAR EEN BIJGEVOEGD PROTOCOL.

IN FEBRUARY AND MARCH 2012, KINDERGARTENS WILL BE CONTACTED TO ASK FOR THEIR PERMISSION TO PARTICIPATE IN A EUROPEAN PROJECT. WHEN KINDERGARTENS AGREE WITH PARTICIPATION, INFORMATIONAL LETTERS AND INFORMED CONSENTS WILL BE HANDED OVER TO THE PARENTS, SO THAT THE PARENTS CAN GIVE CONSENT FOR THEIR CHILD TO PARTICIPATE IN THE STUDY.
FROM APRIL UNTIL JUNE 2012, BASELINE MEASUREMENTS WILL TAKE PLACE IN 1100 FLEMISH PRESCHOOL CHILDREN BETWEEN 3 AND 4 YEARS OLD. DURING THESE BASELINE MEASUREMENTS, PRESCHOOL CHILDREN'S HEIGHT, WEIGHT AND WAIST CIRCUMFERENCE WILL BE MEASURED. PRESCHOOLERS FROM PARENTS THAT PROVIDED CONSENT, WILL WEAR A MOTION SENSOR (ACCELEROMETER) FOR 5 CONSECUTIVE DAYS (INCLUDING 2 WEEKEND DAYS), WHICH WILL BE MOUNTED AT CHILDREN'S RIGHT HIP. THIS DEVICE MEASURES PRESCHOOL CHILDREN'S ACCELEROMETERS, WHICH ENABLES US TO INVESTIGATE THEIR PHYSICAL ACTIVITY LEVEL ACROSS SEVERAL DAYS. FURTHERMORE, PRESCHOOL CHILDREN WILL HAVE TO PERFORM 2 MOTOR SKILL TESTS: BALANCING ON A BENCH THAT IS UPSIDE DOWN AND JUMP FROM SIDE TO SIDE ACROSS A SMALL BAR FOR 30 SECONDS. TEACHERS WILL FILL IN A QUESTIONNAIRE FOR TEACHERS AND PARENTS THAT PROVIDED CONSENT FOR THEIR CHILD TO WEAR AN ACCELEROMETER, WILL BE ASKED TO FILL IN 2 QUESTIONNAIRES: A PRIMARY CAREGIVERS' QUESTIONNAIRE AND A FOOD FREQUENCY QUESTIONNAIRE (FOR THEIR CHILD).
AFTER BASELINE MEASUREMENTS, KINDERGARTENS WILL BE DIVIDED INTO CONTROL KINDERGARTENS AND INTERVENTION KINDERGARTENS. INTERVENTION KINDERGARTENS WILL RECEIVE THE INTERVENTION MATERIAL WHICH THEY WILL BE ABLE TO USE DURING THE SCHOOL YEAR 2012-2013. CONTROL KINDERGARTENS WILL RECEIVE THE INTERVENTION MATERIAL ONE YEAR LATER (SCHOOL YEAR 2013-2014). THE INTERVENTION MATERIAL IS SUPPLEMENTARY MATERIAL ON TOP OF THE DAILY PROGRAMME, IS USER-FRIENDLY AND DOES NOT IMPLY A LOT OF EXTRA WORK FROM THE KINDERGARTEN TEACHERS.
THE INTERVENTION MATERIAL CONSISTS OF 4 TEACHER MANUALS WHICH CONTAIN FUN ACTIVITIES AND STORIES THAT CAN BE USED TO TEACH CHILDREN SOMETHING MORE ABOUT THE DIFFERENT BEHAVIOURS (MORE PHYSICAL ACTIVITY, LESS SEDENTARY BEHAVIOUR, MORE WATER AND LESS SOFT DRINK CONSUMPTION AND HEALTHY SNACKING). EVERY BEHAVIOUR WILL BE REPEATED FOR TWO PERIODS IN THE CLASSROOM. DURING THE FIRST FOCUS, THERE IS A FOCUS ON EACH BEHAVIOUR FOR 4 WEEKS. THE TEACHER HAS 4 WEEKS' TIME TO LEARN THE CHILDREN SOMETHING ABOUT A CERTAIN BEHAVIOUR. ALSO PARENTS ARE INVOLVED IN THE PROJECT. EVERY PRESCHOOL CHILD RECEIVES A NEWSLETTER DURING THE FIRST WEEK OF EACH BEHAVIOUR. DURING THE THIRD WEEK, EVERY CHILD RECEIVES A TIP-CARD WITH TIPS AND TRICKS FOR THE PARENTS HOW TO DO THE BEHAVIOUR AT HOME. AFTER THE FIRST FOCUS, THERE IS A REPETITION PERIOD LATER DURING THE SCHOOL YEAR. DURING THIS REPETITION PERIOD, EVERY BEHAVIOUR IS REPEATED FOR TWO WEEKS EACH. PRESCHOOL CHILDREN RECEIVE A SECOND NEWSLETTER FOR EVERY SPECIFIC BEHAVIOUR AND ALSO A SECOND TIP CARD.
AT THE END OF THE SCHOOL YEAR 2012-2013, FOLLOW-UP MEASUREMENTS WILL TAKE PLACE. THIS WILL BE FROM MAY UNTIL JUNE 2013. AGAIN, THESE 1100 PRESCHOOL CHILDREN (WHICH ARE NOW ONE YEAR OLDER) WILL BE MEASURED (HEIGHT, WEIGHT, WAIST CIRCUMFERENCE). PRESCHOOL CHILDREN WITH CONSENT FROM THEIR PARENTS WILL WEAR AN ACCELEROMETER FOR 5 CONSECUTIVE DAYS (INCLUDING 2 WEEKEND DAYS). PARENTS AND TEACHERS WILL BE ASKED TO FILL IN THE SAME QUESTIONNAIRES AS THE ONES THE YEAR BEFORE.

11. WELKE ZIJN DE ARGUMENTEN (THEORETISCHE, EXPERIMENTELE OF ANDERE) DIE EEN VOORDEEL LATEN VERWACHTEN VAN DE TE TESTEN NIEUWE METHODE, VAN HET TE TESTEN NIEUWE PREPARAAT, ETC. BOVEN DE GEKENDE EN REEDS GEBRUIKTE ?

PRESCHOOL AGE IS A CRITICAL AGE DURING WHICH PRESCHOOL CHILDREN LEARN BEHAVIOURS WHICH THEY WILL PERFORM FOR THE REST OF THEIR LIVES. THEREFORE, IT IS IMPORTANT TO TEACH THEM HEALTHY BEHAVIOURS LIKE PHYSICAL ACTIVITY, LOW LEVELS OF SEDENTARY BEHAVIOUR AND NUTRITION. PHYSICAL ACTIVITY, SEDENTARY BEHAVIOUR AND NUTRITION HAVE AN INFLUENCE ON THE DEVELOPMENT OF OVERWEIGHT AND OBESITY AT A LATER AGE. WHEN PRESCHOOL CHILDREN ALREADY PERFORM UNHEALTHY BEHAVIOURS AT A YOUNG AGE, THERE IS MORE CHANCE THAT THEY WILL ALSO PERFORM THESE UNHEALTHY BEHAVIOURS AT A LATER AGE. IN ADDITION, DURING THE PRESCHOOL AGE IT IS STILL POSSIBLE TO POSITIVELY CHANGE THESE BEHAVIOURS. IN THIS WAY, WE WILL TRY TO PREVENT OVERWEIGHT AND OBESITY, BOTH IN PRESCHOOL CHILDREN AS WELL AS AT A LATER STAGE.

12. WERD EEN ANALOOG ONDERZOEK REEDS ELDERS UITGEVOERD, HETZIJ IN ZIJN GEHEEL, HETZIJ GEDEELTELIJK ?

ZO JA, WAAR ? WAT WAS HET RESULTAAT ? WAAROM WORDT HET IN DEZE STUDIE HERNOMEN ?

THE EUROPEAN ENERGY-STUDY IS COMPARABLE, BUT WAS CONDUCTED IN 10-12 YEAR OLD CHILDREN. RESULTS ARE NOT YET AVAILABLE, AS THE INTERVENTION JUST FINISHED.
THE TOYBOX-PROJECT IS A COMPARABLE STUDY, BUT IN 4- TO 6-YEAR-OLD CHILDREN. AT THIS AGE, BEHAVIOURS ARE BEING DEVELOPED THAT ARE STILL EXECUTED AT A LATER, ADULT AGE. PRESCHOOL CHILDREN HAVE A CRITIQUE AGE TO DEVELOP OVERWEIGHT AND OBESITY AND TO MAINTAIN THIS WEIGHT STATUS DURING ADOLESCENCE AND DURING ADULTHOOD.

13. ZAL EEN CHEMISCHE SUBSTANTIE TOEGEDIEND WORDEN ?

☐ JA ☒ NEEN

ZO JA:

A. LANGS WELKE WEG ?

B. NAAM EN OORSPRONG VAN DE SUBSTANTIE :

C. AAN WIE WORDT DE RECEPTIE, OPSLAG, VERDELING EN TERUGSTUREN VAN NIET-GEBRUIKTE CHEMISCHE SUBSTANTIES TOEVERTROUWD ?

D. ZULLEN RADIO-ISOTOPEN TOEGEDIEND WORDEN ?

☐ JA ☐ NEEN

WELKE ?

14. INDIEN HET OM EEN NIEUWE SUBSTANTIE GAAT, HEEFT DE ONDERZOEKER KENNIS GENOMEN VAN HET VOLLEDIG TOXICOLOGISCH, DIERFARMACOLOGISCH EN HUMAAN DOSSIER ?

☐ JA ☐ NEEN

ZO NEEN, LEG UIT :

15. KEUZE VAN DE PROEFPERSONEN :

A. GEZONDEN ?

☒ JA ☐ NEEN

PATIËNTEN LIJDEND AAN :

B. ZWANGERE VROUWEN OF VROUWEN DIE TIJDENS HET ONDERZOEK ZWANGER KUNNEN WORDEN ?

☐ JA ☒ NEEN

C. AANTAL PROEFPERSONEN IN HET UZ GENT :

D. AANTAL PROEFPERSONEN EXTERN (IN BELGIË): 1100

E. LEEFTIJD : 4-6 YEARS OLD

F. GESLACHT : BOYS AND GIRLS

G. HOW ARE THEY BEING RECRUITED? GREECE (COORDINATING COUNTRY) HAS A LIST WITH ALL FLEMISH MUNICIPALITIES FROM 3 SOCIO-ECONOMIC BACKGROUNDS (LOW, MEDIUM AND HIGH), FROM WHICH 5 MUNICIPALITIES FROM EACH SOCIO-ECONOMIC BACKGROUND WILL BE RANDOMLY CHOSEN. THE KINDERGARTENS WITHIN THESE SELECTED MUNICIPALITIES ARE LISTED AND WILL BE CONTACTED. AFTER A TALK WITH THE KINDERGARTEN PRINCIPAL, IT WILL BECOME CLEAR WHETHER THE SCHOOL WILL/WILL NOT PARTICIPATE. WHEN THE KINDERGARTEN WANTS TO PARTICIPATE, PRESCHOOLERS RECEIVE AN INFORMATIONAL LETTER AND AN INFORMED CONSENT FOR THE PARENTS. IF PARENTS ARE WILLING TO LET THEIR CHILD WEAR AN ACCELEROMETER FOR 5 CONSECUTIVE DAYS, THEY RETURN A SIGNED INFORMED CONSENT. PARENTS THAT DO NOT WANT THEIR CHILD TO BE MEASURED CAN REPORT THIS TO THE INVESTIGATORS.

16. WANNEER VERWACHT MEN VOORDEEL VOOR DE DEELNEMER

A. HEEFT HET EXPERIMENT EEN DIAGNOSTISCH OF THERAPEUTISCH DOEL DAT ONMIDDELLIJK VOORDEEL AAN DE ONDERZOCHTE ZAL BRENGEN ?

☐ JA ☒ NEEN

B. MAAKT HET EXPERIMENT DEEL UIT VAN EEN DIAGNOSTISCH EN THERAPEUTISCH PLAN WAARVAN MEN MAG VERWACHTEN DAT DE RESULTATEN BINNEN AFZIENBARE TIJD VOOR ANDERE ZIEKEN NUTTIG ZULLEN ZIJN ?

☐ JA ☒ NEEN

C. MAAKT HET EXPERIMENT DEEL UIT VAN EEN GEHEEL VAN ONDERZOEKEN WAARVAN HET DIAGNOSTISCH OF THERAPEUTISCH BELANG NIET ONMIDDELLIJK DUIDELIJK IS, MAAR WAARVAN MAG WORDEN VERWACHT DAT DE RESULTATEN LATER TOT DIAGNOSTISCHE OF THERAPEUTISCHE TOEPASSINGEN OF TOT EEN BETERE KENNIS VAN DE FYSIOPATHOLOGISCHE MECHANISMEN ZULLEN LEIDEN ?

☒ JA ☐ NEEN

17. WELKE INTERVENTIES ZIJN SPECIFIEK VOOR DE STUDIE (NAAST DE STANDAARDBEHANDELINGEN), HOE FREQUENT EN GEDURENDE WELKE TIJD ?

A. ZUIVER KLINISCHE EVALUATIES, OM DE ....

B. FUNCTIETESTS OF DYNAMISCHE PROEVEN

WELKE ....

OM DE ....

C. RADIOGRAFISCHE EN/OF ISOTOPISCHE INVESTIGATIES

WELKE ....

OM DE ....

D. BLOEDAFNAMEN : ....

....

....

E. WEEFSELAFNAME : ....

F. ANDERE : ....

18. REKENING HOUDEND MET DE HUIDIGE GEGEVENS VAN DE WETENSCHAP:

A. MEENT U DAT DEZE STUDIE:

☒ WAARSCHIJNLIJK GEEN ENKEL RISICO INHOUDT

☐ EEN MOGELIJK RISICO INHOUDT.

WELK RISICO EN DE FREQUENTIE :

☐ ZEER WAARSCHIJNLIJK EEN RISICO INHOUDT.

WELK RISICO EN DE FREQUENTIE :

B. WELKE ZIJN DE MEEST VOORKOMENDE BIJWERKINGEN VAN HET PREPARAAT ONDER STUDIE ?
(DE BIJWERKINGEN MOETEN EVENEENS DUIDELIJK VERMELD WORDEN IN HET INFORMATIE- EN TOESTEMMINGSFORMULIER VAN DE DEELNEMER)

19. INFORMATIE EN TOESTEMMING VAN DE PROEFPERSONEN

<u>A. WILSBEKWAME VOLWASSENEN</u>

☐ JA ☐ NEEN

WORDT DE TOESTEMMING VAN DE PROEFPERSONEN BEKOMEN NA EEN KLARE EN OBJECTIEVE UITEENZETTING VAN HET DOEL VAN HET ONDERZOEK ?

SCHRIFTELIJK :

☐ JA ☐ NEEN

MONDELING :

☐ JA ☐ NEEN

ZO NEEN, WAAROM NIET ?

WORDT IN DIT LAATSTE GEVAL DE TOESTEMMING GEGEVEN DOOR ANDEREN DAN DE PROEFPERSONEN ?

☐ JA ☐ NEEN

ZO JA, DOOR WIE ?

ZIJN ER SPECIALE GROEPEN : EIGEN STUDENTEN, EIGEN PERSONEEL ?

<u>B. WILSONBEKWAME VOLWASSENEN</u> (= SOMMIGE PSYCHIATRISCHE PATIENTEN, PERSONEN IN DE ONMOGELIJKHEID HUN WIL TE UITEN, ...)

☐ JA ☐ NEEN

WORDT DE TOESTEMMING GEGEVEN DOOR ANDEREN DAN DE PROEFPERSONEN ?

☐ JA ☐ NEEN

ZO JA, DOOR WIE ?

C. KINDEREN

☒ JA ☐ NEEN

WORDT DE TOESTEMMING GEVRAAGD VAN HUN WETTELIJKE VERANTWOORDELIJKEN ?

☒ JA ☐ NEEN

IS ER EEN INFORMATIE- EN TOESTEMMINGSFORMULIER VOOR KINDEREN VANAF 12 JAAR VOORZIEN?

☐ JA ☒ NEEN

20. IS HET INFORMATIEFORMULIER VOOR DE PROEFPERSONEN IN BIJLAGE GEVOEGD

☒ JA ☐ NEEN

ZO NEEN, WAAROM NIET ?

21. IS HET FORMULIER VOOR SCHRIFTELIJKE TOESTEMMING IN BIJLAGE GEVOEGD ?

☒ JA ☐ NEEN

ZO NEEN, WAAROM NIET ?

22. ZULLEN DE PERSONEN IN DE LOOP VAN DEZE STUDIE VOORTDUREND ONDER MEDISCH TOEZICHT STAAN

☐ JA ☒ NEEN

A. WIE IS DE TOEZICHTHOUDENDE GENEESHEER ?

B. ZAL DIT TOEZICHT, ZO NODIG, VERZEKERD KUNNEN WORDEN TIJDENS DE UREN DIE OP DE STUDIE VOLGEN ?

☐ JA ☐ NEEN

C. ALS DE PERSOON NAAR HUIS TERUGKEERT TIJDENS DE UREN DIE OP HET ONDERZOEK VOLGEN, ZAL IN GEVAL VAN NOOD SNEL CONTACT MET EEN GENEESHEER KUNNEN OPGENOMEN WORDEN ?

☐ JA ☐ NEEN

D. NAAM VAN DEZE GENEESHEER ?

23. IS ER VOOR HET ONDERZOEK EEN VERZEKERING AFGESLOTEN CONFORM DE BELGISCHE WET VAN 7 MEI 2004?

(HET VERZEKERINGSCERTIFICAAT MOET BIJ DE AANVRAAG GEVOEGD WORDEN INDIEN NIET VERZEKERD DOOR UZGENT/UGENT)

☒ JA

DOOR WELKE VERZEKERINGSPOLIS BENT U VERZEKERD ?
(VERWIJZEN NAAR EEN BIJGEVOEGD DOCUMENT VOLSTAAT NIET)

☐ UZ GENT

☒ UGENT: VERZEKERINGSPOLIS VAN DE UGENT-BA

☐ ANDERE + OMVANG VAN DE DEKKING:

☐ NEEN, WAAROM NIET?

24 . FINANCIËLE OVEREENKOMST

(INDIEN EEN DEFINITIEVE FINANCIËLE OVEREENKOMST NOG NIET BESCHIKBAAR IS, DAN KAN EEN BUDGET PROPOSAL DAT TEGENGETEKEND IS DOOR EEN VERTEGENWOORDIGER VAN DE FINANCIERDER + ONDERZOEKER VOLSTAAN) INDIEN HET BEDRAG VAN DE DEFINITIEVE FINANCIËLE OVEREENKOMST HOGER IS DAN HET INGEDIENDE "BUDGET PROPOSAL", MOET DEZE DEFINITIEVE FINANCIËLE OVEREENKOMST ALSNOG TER GOEDKEURING VOORGELEGD WORDEN AAN HET ETHISCH COMITÉ)

☒ NIET VAN TOEPASSING

☐ AANWEZIG MET VOLGENDE ONDERVERDELING:

- ☐ ERELOON:
- ☐ VERGOEDING VOOR TECHNISCHE PRESTATIES:

IK VERKLAAR DE GEHELE VERANTWOORDELIJKHEID VAN HET HIERBOVEN VERMELD PROJECT OP MIJ TE NEMEN EN BEVESTIG DAT VOOR ZOVER DE HUIDIGE KENNIS HET TOELAAT, DE GEGEVEN INLICHTINGEN MET DE WERKELIJKHEID OVEREENSTEMMEN.

DE ONDERZOEKER,	HET U.Z. DIENSTHOOFD OF DE VAKGROEPVOORZITTER (VOOR AKKOORD)
DATUM :<br>NAAM : PROF. DR. ILSE DE BOURDEAUDHUIJ<br>HANDTEKENING :	DATUM :<br>NAAM : PROF. DR. DIRK DE CLERCQ<br>HANDTEKENING :

HET UZ-DIENSTHOOFD OF DE VAKGROEPVOORZITTER VAN EVENTUELE ANDERE BETROKKEN DIENSTEN (VOOR AKKOORD)

DATUM :<br>NAAM :<br>HANDTEKENING :	DATUM :<br>NAAM :<br>HANDTEKENING :
