# Supplementary material for: Effect and process evaluation of a kindergarten-based, family-involved intervention with a randomized cluster design on sedentary behaviour in 4- to 6- year old European preschool children: The ToyBox-study
Source: PLoS One. 2017 Apr 5;12(4):e0172730. doi: 10.1371/journal.pone.0172730 (PMC5381762; doi:10.1371/journal.pone.0172730)
Supplement: S2 Text — (PDF) [file pone.0172730.s004.pdf]

SEVENTH FRAMEWORK PROGRAMME

THEME [KBBE-2009-2-1-03]
[Behavioural models for prevention of obesity, with a particular focus on children]

Grant agreement for: Collaborative project[*]

Annex I - "Description of Work"

Project acronym: ToyBox

Project full title: " Multifactorial evidence based approach using behavioural models in understanding and promoting fun, healthy food, play and policy for the prevention of obesity in early childhood: ToyBox "

Grant agreement no: 245200

Version date: 2013-01-28

Table of Contents

Part A

A.1 Project summary 3
A.2 List of beneficiaries 4
A.3 Overall budget breakdown for the project 5

Workplan Tables

WT1 List of work packages 1
WT2 List of deliverables 2
WT3 Work package descriptions 10
Work package 1........ 10
Work package 2........ 13
Work package 3........ 17
Work package 4........ 20
Work package 5........ 24
Work package 6........ 28
Work package 7........ 32
Work package 8........ 35
Work package 9........ 40
Work package 10........ 43
WT4 List of milestones 46
WT5 Tentative schedule of project reviews 48
WT6 Project effort by beneficiaries and work package 49
WT7 Project effort by activity type per beneficiary 50
WT8 Project efforts and costs 53

A1:
Project summary

Project Number [1]	245200	Project Acronym [2]	ToyBox

One form per project

General information

Project title [3]	Multifactorial evidence based approach using behavioural models in understanding and promoting fun, healthy food, play and policy for the prevention of obesity in early childhood: ToyBox		
Starting date [4]	01/03/2010		
Duration in months [5]	50		
Call (part) identifier [6]	FP7-KBBE-2009-3		
Activity code(s) most relevant to your topic [7]	KBBE-2009-2-1-03: Behavioural models for prevention of obesity, with a particular focus on children		

Abstract [9]

Objective The ToyBox proposal addresses KBBE-2009-2-1-03 - Behavioural models for prevention of obesity, with a particular focus on children. It will primary aim to influence children’s behaviours and prevent obesity in early childhood. Strategy The proposal will identify key behaviours related to early childhood obesity and their determinants and evaluate behavioural models and educational strategies. Based on the obtained insights at a local level, a multidisciplinary team will develop and implement a school based family involved intervention programme that could be applied on a European scale. Process, impact, outcome and cost-effectiveness evaluation will be conducted to support decision making for European Public Health Policy. Methods The combined use of Precede-Proceed Model and Intervention Mapping will provide the framework for the development, implementation and evaluation of the ToyBox intervention. To achieve this, the project will be subdivided into 10 WPs. This carefully planned stepwise approach will include systematic reviews, secondary analyses of existing data sets, focus group research and school policies overview. Consortium The ToyBox project consortium spans the necessary multidisciplinary variety of experts such as public health experts, epidemiologists, nutritionists, physical activity experts, pedagogists, psychologists, behavioural scientists, nutritionists, paediatricians, early childhood psychologists, health economists, totalling 15 partners, from 10 countries. The consortium, consists of 11 universities, 1 research institute, 2 advocacy groups and an SME representing all regions of Europe. The consortium has ample experience in conducting and coordinating multi-centre international research as well as undertaking dissemination activities to all relevant stakeholders.

A2:
List of Beneficiaries

Project Number [1]	245200	Project Acronym [2]	ToyBox

List of Beneficiaries

No	Name	Short name	Country	Project entry month[10]	Project exit month
1	HAROKOPIO UNIVERSITY	HUA	Greece	1	50
2	LUDWIG-MAXIMILIANS-UNIVERSITAET MUENCHEN	LMU-Muenchen	Germany	1	50
3	UNIVERSITEIT GENT	UGent	Belgium	1	50
4	VERENIGING VOOR CHRISTELIJK HOGER ONDERWIJS WETENSCHAPPELIJK ONDERZOEK EN PATIENTENZORG	VUA	Netherlands	1	50
5	UNIVERSIDAD DE ZARAGOZA	UniZar	Spain	1	50
6	HOGSKOLEN I OSLO OG AKERSHUS	HiOA	Norway	18	50
7	UNIVERSITY OF DURHAM	UDUR	United Kingdom	1	50
8	Staatsinstitut fur Fruhpadagogik	IFP	Germany	1	50
9	INSTYTUT POMNIK CENTRUM ZDROWIA DZIECKA	CMHI	Poland	1	50
10	Medical University Of Varna	MUV	Bulgaria	1	50
11	THE INTERNATIONAL ASSOCIATION FOR THE STUDY OF OBESITY	IASO	United Kingdom	1	50
12	NATIONAAL INSTITUUT VOOR GEZONDSHEIDSBEVORDERING EN ZIEKTEPREVENTIE	NIGZ	Netherlands	1	50
13	AOK-Verlag GmbH	AOK-Verlag	Germany	1	50
14	Roehampton University	RoU	United Kingdom	1	50
15	UNIVERSITE DU LUXEMBOURG	ULU	Luxembourg	1	50
16	STICHTING VU-VUMC	VUmc	Netherlands	23	50
17	CBO BV	CBO	Netherlands	30	50

A3:
Budget Breakdown

Project Number [1]	245200	Project Acronym [2]	ToyBox

One Form per Project

Participant number in this project [11]	Participant short name	Fund. % [12]	Ind. costs [13]	Estimated eligible costs (whole duration of the project)					Requested EU contribution
				RTD / Innovation (A)	Demonstration (B)	Management (C)	Other (D)	Total A+B+C+D	
1	HUA	75.0	T	470,400.00	0.00	211,200.00	25,000.00	706,600.00	589,000.00
2	LMU-Muenchen	75.0	T	513,600.00	0.00	0.00	0.00	513,600.00	385,200.00
3	UGent	75.0	T	682,400.00	0.00	0.00	0.00	682,400.00	511,800.00
4	VUA	75.0	T	0.00	0.00	0.00	0.00	0.00	0.00
5	UniZar	75.0	T	397,600.00	0.00	0.00	0.00	397,600.00	298,200.00
6	HiOA	75.0	T	140,000.00	0.00	0.00	0.00	140,000.00	105,000.00
7	UDUR	75.0	T	120,800.00	0.00	0.00	0.00	120,800.00	90,600.00
8	IFP	75.0	T	128,800.00	0.00	0.00	0.00	128,800.00	96,600.00
9	CMHI	75.0	T	198,260.00	0.00	0.00	0.00	198,260.00	148,695.00
10	MUV	75.0	T	198,400.00	0.00	0.00	0.00	198,400.00	148,800.00
11	IASO	75.0	T	185,600.00	0.00	0.00	0.00	185,600.00	139,200.00
12	NIGZ	75.0	T	0.00	0.00	0.00	0.00	0.00	0.00
13	AOK-Verlag	75.0	F	179,680.00	0.00	0.00	0.00	179,680.00	134,760.00
14	RoU	75.0	T	46,800.00	0.00	0.00	0.00	46,800.00	35,100.00
15	ULU	75.0	T	25,200.00	0.00	0.00	0.00	25,200.00	18,900.00
16	VUmc	75.0	T	196,000.00	0.00	0.00	0.00	196,000.00	147,000.00
17	CBO	50.0	A	0.00	0.00	0.00	187,200.00	187,200.00	140,400.00
Total				3,483,540.00	0.00	211,200.00	212,200.00	3,906,940.00	2,989,255.00

Note that the budget mentioned in this table is the total budget requested by the Beneficiary and associated Third Parties.

*** The following funding schemes are distinguished**

Collaborative Project (if a distinction is made in the call please state which type of Collaborative project is referred to: (i) Small of medium-scale focused research project, (ii) Large-scale integrating project, (iii) Project targeted to special groups such as SMEs and other smaller actors), Network of Excellence, Coordination Action, Support Action.

1. Project number

The project number has been assigned by the Commission as the unique identifier for your project, and it cannot be changed. The project number **should appear on each page of the grant agreement preparation documents** to prevent errors during its handling.

2. Project acronym

Use the project acronym as indicated in the submitted proposal. It cannot be changed, unless agreed during the negotiations. The same acronym **should appear on each page of the grant agreement preparation documents** to prevent errors during its handling.

3. Project title

Use the title (preferably no longer than 200 characters) as indicated in the submitted proposal. Minor corrections are possible if agreed during the preparation of the grant agreement.

4. Starting date

Unless a specific (fixed) starting date is duly justified and agreed upon during the preparation of the Grant Agreement, the project will start on the first day of the month following the entry info force of the Grant Agreement (NB : entry into force = signature by the Commission). Please note that if a fixed starting date is used, you will be required to provide a detailed justification on a separate note.

5. Duration

Insert the duration of the project in full months.

6. Call (part) identifier

The Call (part) identifier is the reference number given in the call or part of the call you were addressing, as indicated in the publication of the call in the Official Journal of the European Union. You have to use the identifier given by the Commission in the letter inviting to prepare the grant agreement.

7. Activity code

Select the activity code from the drop-down menu.

8. Free keywords

Use the free keywords from your original proposal; changes and additions are possible.

9. Abstract

10. The month at which the participant joined the consortium, month 1 marking the start date of the project, and all other start dates being relative to this start date.

11. The number allocated by the Consortium to the participant for this project.

12. Include the funding % for RTD/Innovation – either 50% or 75%

13. Indirect cost model
- **A: Actual Costs**
- **S: Actual Costs Simplified Method**
- **T: Transitional Flat rate**
- **F :Flat Rate**

Workplan Tables

Project number

245200

Project title

ToyBox—Multifactorial evidence based approach using behavioural models in understanding and promoting fun, healthy food, play and policy for the prevention of obesity in early childhood: ToyBox

Call (part) identifier

FP7-KBBE-2009-3

Funding scheme

Collaborative project

WT1
List of work packages

Project Number [1]	245200	Project Acronym [2]	ToyBox

LIST OF WORK PACKAGES (WP)

WP Number [53]	WP Title	Type of activity [54]	Lead beneficiary number [55]	Person-months [56]	Start month [57]	End month [58]
WP 1	Coordination and management	MGT	1	26.00	1	50
WP 2	Identification of behaviours associated with overweight and obesity in childhood	RTD	4	27.00	1	32
WP 3	Identification of the determinants of most important energy-balance related behaviours	RTD	3	34.00	5	32
WP 4	Review of behavioural models and educational strategies to promote healthy weight and healthy energy	RTD	7	25.00	1	32
WP 5	Contextual and legislation framework of pre-primary education and school-based health promotion	RTD	12	42.00	2	40
WP 6	Development of school-based family involved intervention programme applicable on a European scale	RTD	2	106.00	6	46
WP 7	Implementation and evaluation of a cluster randomized intervention	RTD	1	144.70	20	50
WP 8	Development, validation and training for the outcome and impact assessment tools	RTD	5	26.80	1	36
WP 9	Evaluation of the cost-effectiveness of the intervention	RTD	3	17.50	20	48
WP 10	Dissemination	OTHER	11	29.00	6	50
			Total	478.00		

WT2:
List of Deliverables

Project Number [1]	245200	Project Acronym [2]	ToyBox

List of Deliverables - to be submitted for review to EC

Delive-rable Number [61]	Deliverable Title	WP number [53]	Lead benefi-ciary number	Estimated indicative person-months	Nature [62]	Dissemi-nation level [63]	Delivery date [64]
D1.1	Information and communication system	1	1	0.15	O	PP	3
D1.2	Website including a restricted area and publiuc-access area	1	1	0.15	O	PP	6
D1.3	Data base including all data from relevant existing studies to be used inthe secondary data analyses	1	1	0.15	O	CO	6
D1.4	Data base including all data and results from WPs 2-9	1	1	0.15	O	RE	50
D1.5	Complete sets of ToyBox intervention materials (electronic and hard-copy format and material...	1	1	0.15	R	PU	50
D2.1	A protocol for the systematic review on the most important energy balance behaviours	2	4	1.00	O	RE	2
D2.2	A protocol for the secondary data analysis of existing data sets in the participating countries	2	4	1.00	O	RE	2
D2.3	A brief report with recommendations for the focus group interviews	2	4	18.00	R	PU	5

Deliverable Number [61]	Deliverable Title	WP number [53]	Lead beneficiary number	Estimated indicative person-months	Nature [62]	Dissemination level [63]	Delivery date [64]
	to be held in WP3						
D2.4	A report with input and recommendations to be used for the development of the intervention	2	4	5.00	R	RE	8
D2.5	A scientific paper submitted on the systematic review	2	4	1.00	O	PU	32
D2.6	A scientific paper submitted on the secondary analyses	2	4	1.00	O	PU	32
D3.1	A protocol for the focus groups	3	3	1.00	O	RE	6
D3.2	A standardised form for data gathering and analyses	3	3	0.50	O	RE	7
D3.3	A report by country on specifically identified parental determinants which need to be addressed and	3	3	14.50	R	RE	12
D3.4	A report from teachers' focus groups, with identified determinants by country (for WP6)	3	3	14.00	R	RE	12
D3.5	A scientific paper submitted in a peer-reviewed journal on the focus group research	3	3	2.00	O	PU	32
D3.6	A scientific paper submitted in a peer-reviewed journal on the	3	3	2.00	O	PU	32

Delive-rable Number [61]	Deliverable Title	WP number [53]	Lead benefi-ciary number	Estimated indicative person-months	Nature [62]	Dissemi-nation level [63]	Delivery date [64]
	systematic review						
D4.1	A report on the reviews on educational strategies and psychological approaches	4	14	5.00	R	PU	6
D4.2	A report on behavioural models used in school-based interventions	4	7	5.00	R	PU	6
D4.3	A report with recommendations to inform WP6	4	7	9.00	R	RE	8
D4.4	A scientific paper submitted on most important educational strategies	4	14	2.00	O	PU	32
D4.5	A scientific paper on the systematic review of behavioural models	4	7	2.00	O	PU	32
D4.6	A scientific paper on the evidence base...	4	7	2.00	O	PU	32
D5.1	A standardized form for data gathering and analysing of policies and legislations	5	12	1.50	O	RE	4
D5.2	A standardized form for data gathering and analysing of health promotion activities	5	12	1.50	O	RE	6
D5.3	Report on existing policies and regulations applying in pre-primary education	5	12	6.00	R	PU	6

Delive-rable Number [61]	Deliverable Title	WP number [53]	Lead benefi-ciary number	Estimated indicative person-months	Nature [62]	Dissemi-nation level [63]	Delivery date [64]
D5.4	Report on health promotion activities policy and legislations	5	12	6.00	R	PU	8
D5.5	Report on ongoing health promotion activities	5	12	6.00	R	PU	8
D5.6	Report on most important contextual factors affecting success	5	3	6.00	R	PU	8
D5.7	Report on good examples of policies and health promotion activities in the school setting	5	12	3.00	R	PU	9
D5.8	Blueprint for strategies and core components for the development of the ToyBox	5	12	1.50	O	RE	9
D5.9	Blueprint on what should be reported in a scientific paper presenting an intervention study	5	12	1.50	O	PU	9
D5.10	A scientific paper submitted on existing health promotion activities	5	12	3.00	O	PU	40
D5.11	A scientific paper submitted on obstacles and success factors for developing and implementing	5	12	3.00	O	PU	40
D5.12	A scientific paper submitted on strategies and recommendations for policy makers	5	12	3.00	O	PU	40

Delive-rable Number [61]	Deliverable Title	WP number [53]	Lead benefi-ciary number	Estimated indicative person-months	Nature [62]	Dissemi-nation level [63]	Delivery date [64]
D6.1	ToyBox Teacher's General Guide and ToyBox Classroom Activities Guide developed in Engllish	6	2	10.00	O	RE	21
D6.2	ToyBox Teacher's General Guide and ToyBox Classroom Activities Guide - Translated and adapted in 6	6	2	18.00	O	RE	23
D6.3	ToyBox Parental Activities Guide-Developed in English	6	2	10.00	O	RE	21
D6.4	ToyBox Parental Activities Guide-Translated and adapted for use in six countries	6	2	15.00	O	RE	21
D6.5	All material printed and CD-ROMs with presentations prepared and ready for use for all six countries	6	2	6.00	O	RE	24
D6.6	Training modules for teachers in participating countries developed	6	2	12.00	O	RE	27
D6.7	The total set of ToyBox printed material and toys delivered in all participating countries	6	2	10.00	O	RE	27
D6.8	Internet material developed and put online to support the	6	2	10.00	O	RE	31

Delive-rable Number [61]	Deliverable Title	WP number [53]	Lead benefi-ciary number	Estimated indicative person-months	Nature [62]	Dissemi-nation level [63]	Delivery date [64]
	programme, in the languages						
D6.9	Final versions of ToyBox material in all languages-material for classroom-report on changes	6	2	15.00	O	PU	46
D7.1	A protocol for the implementation and evaluation of the ToyBox intervention	7	1	8.00	O	RE	25
D7.2	Process evaluation tools developed	7	1	6.00	O	RE	26
D7.3	Report on the process, impact and outcome evaluation at a country and European scale	7	1	124.70	R	PU	48
D7.4	Report on mediators and moderators affecting the magnitude of the intervention	7	1	6.00	R	PU	50
D8.1	A report on the review of the literature on tools assessing food intake and physical (in)activity	8	5	2.00	R	PU	10
D8.2	Protocol(s) for planning, training and data collection	8	5	9.80	O	RE	26
D8.3	A protocol for the collection of study data from the participating centres with an agreed format	8	5	7.00	O	RE	25
D8.4	Databases for data entry and manual	8	5	2.00	O	RE	28

Delive-rable Number [61]	Deliverable Title	WP number [53]	Lead benefi-ciary number	Estimated indicative person-months	Nature [62]	Dissemi-nation level [63]	Delivery date [64]
	for data entry procedures will be developed for all tools						
D8.5	A scientific paper submitted in a peer reviewed journal on the review of the literature on tools	8	5	2.00	O	PU	16
D8.6	Report on the validity and harmonization process of the tools	8	5	2.00	R	PU	36
D8.7	A scientific paper submitted in a peer reviewed journal on the development and validity testing	8	5	2.00	O	PU	36
D9.1	Interim report with the cost of the intervention	9	3	3.50	R	PU	43
D9.2	Projection model in MS Excel	9	3	4.00	O	PU	46
D9.3	A full report containing the health economic model description, data inputs and results	9	3	6.00	R	PU	48
D9.4	A brief report presenting key findings regarding long term costs, effects and cost-effectiveness	9	3	4.00	R	PU	48
D10.1	A listing of the Network of Networks	10	11	2.00	O	PU	6
D10.2	One Network of networks meetings	10	11	12.00	O	PU	50

Delive-rable Number [61]	Deliverable Title	WP number [53]	Lead benefi-ciary number	Estimated indicative person-months	Nature [62]	Dissemi-nation level [63]	Delivery date [64]
D10.3	Matrices of key scientific findings against specific policies and interventions	10	11	3.00	O	PU	50
D10.4	A list with at least twenty (20) items that appeared in independent public media	10	11	2.00	O	RE	50
D10.5	A list with at least six (6) scientific papers submitted in a peer reviewed journals or conference	10	11	2.00	O	CO	50
D10.6	Policy recommendation fact sheets for EU, national and local policy-makers	10	11	8.00	O	PU	50
			Total	452.75			

WT3:
Work package description

Project Number [1]	245200	Project Acronym [2]	ToyBox

One form per Work Package

Work package number [53]	WP1	Type of activity [54]	MGT
Work package title	Coordination and management		
Start month	1		
End month	50		
Lead beneficiary number [55]	1		

Objectives

The aim of this WP is the coordination, management and administration of the project
1.1 Support the overall running, and coordinate the integrated progress of the project, including financial management
1.2 Set up a structure for communication
1.3 Store and manage all data obtained in WP 2-9
1.4 Retrieve and administer all results from WP2-9
1.5 Communicate with and report to the European Commission

Description of work and role of partners

Description of work
This WP will comprise the co-ordination task to promote and ensure integrated and timely progress of the project communication. For this purpose this WP will set up a communication and feedback structure, apply a quality control infrastructure, prepare and organise progress meetings, coordinate timely reporting of content, deliverables and finance, and generally support the steering Committee and WPs of ToyBox. Furthermore, it is of importance that all results from WP2-5 will be stored centrally in databases that can easily be accessed by all WP 6 participants. This will be time efficient and it will guarantee that all results are reported in the same way. Formats for data storage will be decided upon in close collaboration with the relevant WP leaders. It is also of great importance that all data obtained in WP 2-9 are stored and managed centrally after initial data management and analyses within the subsequent WPs. This WP will also acquire the data for the secondary data analyses which will be more efficient since different WPs will use the same datasets.

The work in this WP will consist of five Tasks:

Task 1.1 (month 0- 50) MGT
Support the overall running of the project, including the financial management
1.1.1 Coordinate timely reporting of content, deliverables and finance, and generally support the steering Committee and WPs of the project
1.1.2 Allocate the budget to different partners and activities and distribute payments received from the Commission to the partners.
1.1.3 The coordinator will also manage changes to the membership of the consortium.

Task 1.2 (month 0-2) OTH (Dissemination)
Set up a structure for communication
1.2.1 Develop the programme's website to include a restricted area (for ToyBox partners only), a restricted area for teachers of intervention schools and a public-access area; an information system will be developed for the restricted area to enable communication between partners (e.g. forum) and upload of relevant documents.
1.2.2 Include important documents (i.e. minutes of the meetings, tools, etc) to the ToyBox partners' restricted area of the website;
1.2.3 Include a brief introduction and guide for the intervention, ToyBox material, Frequently Asked Questions & Answers and useful contact details for the local teams to the teachers restricted area of the website;
1.2.4 Update the website with relevant documents and results;
1.2.5 Prepare an overview for plenary meetings and regular telephone conferences.

Task 1.3 (month 0-3) RTD
Acquire existing datasets for secondary data analyses in WP 2.
1.3.1 Contact owners of existing datasets and ask for the data;
1.3.2 Define the data quality;
1.3.3 Develop a data base to store datasets from all relevant existing studies; if additional datasets beyond the already identified ones, then distribute payments received from the Commission to the partners.
1.3.4 Send data to WP2 leader, where the data will be checked and restructured.

Task 1.4 (month 0-50) RTD
Develop and manage data storage and accessibility
1.4.1 Define formats to deliver cleaned data and final datasets for central storage concerning literature reviews (WPs 2, 4 and 5) including information on search strategies, references of the retrieved papers and relevant tables;
1.4.2 Define formats to deliver data concerning focus group interviews (WP 3) for central storage including transcription;
1.4.3 Define formats to deliver cleaned and final datasets from the evaluation of the ToyBox intervention (WPs 7 and 8) including codebooks and recoding, scale formation and other data reduction syntax;
1.4.4 Develop a data base to store datasets from all relevant WPs and ensure easy access for all partners.

Task 1.5 (month 6-50) MGT
Communicate and report to the European Commission
Write and edit annual reports (month 12 and 30) and final reports, including a full description of the school-based intervention programme aiming to prevent childhood obesity that will be ready for implementation across Europe, based on the reports from WP 2-10.

Person-Months per Participant

Participant number [10]	Participant short name [11]	Person-months per participant
1	HUA	26.00
	Total	26.00

List of deliverables

Delive-rable Number [61]	Deliverable Title	Lead benefi-ciary number	Estimated indicative person-months	Nature [62]	Dissemi-nation level [63]	Delivery date [64]
D1.1	Information and communication system	1	0.15	O	PP	3
D1.2	Website including a restricted area and publiuc-access area	1	0.15	O	PP	6
D1.3	Data base including all data from relevant existing studies to be used inthe secondary data analyses	1	0.15	O	CO	6
D1.4	Data base including all data and results from WPs 2-9	1	0.15	O	RE	50
D1.5	Complete sets of ToyBox intervention materials (electronic and hard-copy format and material...	1	0.15	R	PU	50
		Total	0.75			

Description of deliverables

D1.1) Information and communication system: (based on Task 1.2) [month 3]<br>D1.2) Website including a restricted area and publiuc-access area: Full title: Website including a restricted area (for ToyBox partners), a restricted area for teachers of intervention schools and a public-access area (based on Task 1.2) [month 6]<br>D1.3) Data base including all data from relevant existing studies to be used inthe secondary data analyses: (based on Task 1.3) [month 6]<br>D1.4) Data base including all data and results from WPs 2-9: (based on Task 1.4) [month 50]<br>D1.5) Complete sets of ToyBox intervention materials (electronic and hard-copy format and material...: Full title: Complete sets of ToyBox intervention materials (electronic and hard-copy format and material for classroom activities and games) in six languages (part of the final report) The person months are indicative as this is part of the final report. [month 50]

Schedule of relevant Milestones

Milestone number [59]	Milestone name	Lead beneficiary number	Delivery date from Annex I [60]	Comments
MS1	Kick-off Steering Committee meeting	1	2	
MS2	Opening of the web-portal	1	6	

WT3:
Work package description

Project Number [1]	245200	Project Acronym [2]	ToyBox

One form per Work Package

Work package number [53]	WP2	Type of activity [54]	RTD
Work package title	Identification of behaviours associated with overweight and obesity in childhood		
Start month	1		
End month	32		
Lead beneficiary number [55]	4		

Objectives

2.1 Identification of the most important energy balance related behaviours among young children that are associated with overweight and obesity.
2.2 Identification of risk groups (family demographics, socioeconomic indices and parental BMI) with regard to children's energy balance related behaviours, overweight and obesity.
2.3 Translation of findings into practical input for WP3 and WP6.

Description of work and role of partners

In this WP we will focus on three main energy-balance related behaviours i.e. physical activity, sedentary and eating behaviours and examine which specific sub-behaviours are most important regarding children's overweight and obesity. The review and secondary analyses will focus on relevant sub-behaviours of eating (e.g. skipping breakfast, snacking, soft drink consumption, fruit and vegetable consumption, eating in front of TV, etc), physical activity (active transport, leisure time physical activity, sports, school time physical activity, domestic activities, etc) and sedentary behaviours (television viewing, internet use, computer games, reading, etc) and their relationship with overweight and obesity.

To ensure easy flow of the text the term 'behaviour' will be used in all WPs and deliverables to describe behaviours and sub-behaviours.

VUmc will focus on dietary and sedentary behaviours, while UGent will focus on the physical activity behaviours.

The work in this WP will consist of three main Tasks:

Tasks 2.1 (month 1-12) (VUmc, UGent)
Systematic review on energy balance related behaviours in young children and identification of risk groups with regard to these behaviours
Identify the most important energy balance related behaviours of young children and certain risk-groups in the population with regard to these behaviours. This information will be used in WP3 and WP 6.
2.1.1 Develop a protocol for a systematic review of the existing literature, including the development of inclusion criteria (including a list of exposures/behaviours and outcomes of interest) and method of quality assessment. Develop and running a search strategy to identify relevant primary studies. Only primary studies reported from 1990 will be included, since behaviour patterns have been subject to change since then;
2.1.2 Review abstracts of papers identified from the search (Task 2.1.1) for possible inclusion in the review by two independent reviewers;
2.1.3 Retrieve full copies of papers that were identified for possible inclusion and, after reading the papers, make a final decision about which studies should be included in the review;
2.1.4 Extract relevant data from studies that will be included in the review;
2.1.5 Score the methodological quality of the included studies by two independent reviewers;
2.1.6 Conduct data analysis;
2.1.7 Write the report of the systematic review, including an assessment of 1) the most important energy balance-related behaviours of young children, that are associated with overweight and obesity in early childhood, and 2) risk groups with regard to energy balance related behaviours, overweight and obesity (based on family demographics and socioeconomic indices and parental BMI);
2.1.8 Report data and results to WP1 to store and manage the data and the results.

Task 2.2 (months 1-12)
Conduct secondary data analyses
Secondary data analyses will be conducted using existing data sets from participating countries on energy balance related behaviours among young children.
2.2.1 Identify available and appropriate studies in participating countries for the secondary data analysis;
2.2.2 Develop a protocol for accumulating existing datasets from participating countries on overweight and obesity indices and children's energy balance behaviours related to overweight and obesity in early childhood. If additional datasets are needed, then contact coordinator who is responsible for acquiring them (Task 1.3);
2.2.3 Conduct the secondary data analysis in the selected studies (identified by Task 2.2.1). Where possible within the timeframe, data will be transferred following anonymisation of the data to VUmc, and analysed. Where it is more appropriate for the analysis to be carried out by academic colleagues based at sites (other Partner sites or other Institutions) that host the data (e.g. because of ethical issues associated with transfer of data), VUmc will co-ordinate the analysis at these sites;
2.2.4 Write the report of the secondary data analysis, to include an assessment of the most important energy balance related behaviours related to overweight and obesity in early childhood;
2.2.5 Report data and results to WP1 to store and manage the data and the results.

VUmc will conduct Task 2.2 but in close co-operation with WP participants and others based at other sites hosting the data for the primary studies selected for the secondary data analysis.

Task 2.3 (month 4-8)
Translate findings into practical input for WP3 and WP6.
2.3.1 Discuss with WP participants the findings from the systematic review and secondary data analysis;
2.3.2 Formulate draft recommendations regarding the most important energy balance-related behaviours of young children. These will inform the content and formation of the focus groups interviews (WP 3);
2.3.3 Formulate recommendations on risk groups with regard to energy balance related behaviours and/or overweight and obesity that need to be included in the focus group interviews (WP3);
2.3.4 Write the report of the recommendations to inform the development of the intervention material (WP6).

Task 2.3 will be co-ordinated by Partner 4 (VUmc), but all WP participants will be involved in the drafting of and agreement on the recommendations.

Person-Months per Participant

Participant number [10]	Participant short name [11]	Person-months per participant
1	HUA	1.00
2	LMU-Muenchen	1.00
3	UGent	4.00
4	VUA	18.00
5	UniZar	1.00
9	CMHI	1.00
10	MUV	1.00
	Total	27.00

List of deliverables

Delive-rable Number [61]	Deliverable Title	Lead benefi-ciary number	Estimated indicative person-months	Nature [62]	Dissemi-nation level [63]	Delivery date [64]
D2.1	A protocol for the systematic review on the most important energy balance behaviours	4	1.00	O	RE	2
D2.2	A protocol for the secondary data analysis of existing data sets in the participating countries	4	1.00	O	RE	2
D2.3	A brief report with recommendations for the focus group interviews to be held in WP3	4	18.00	R	PU	5
D2.4	A report with input and recommendations to be used for the development of the intervention	4	5.00	R	RE	8
D2.5	A scientific paper submitted on the systematic review	4	1.00	O	PU	32
D2.6	A scientific paper submitted on the secondary analyses	4	1.00	O	PU	32
		Total	27.00			

Description of deliverables

D2.1) A protocol for the systematic review on the most important energy balance behaviours: Full title: A protocol for the systematic review on the most important energy balance behaviours and sub-behaviours and the identification of risk groups (based on family demographics, socioeconomic indices and parental BMI) that are associated with overweight and obesity in young children (based on Task 2.1) [month 2]

D2.2) A protocol for the secondary data analysis of existing data sets in the participating countries: Full title:A protocol for the secondary data analysis of existing data sets in the participating countries on energy balance behaviours associated with overweight and obesity in young children (based on Task 2.2) [month 2]

D2.3) A brief report with recommendations for the focus group interviews to be held in WP3: Full title: A brief report with recommendations for the focus group interviews to be held in WP3, regarding the energy-balance sub-behaviours that have to be discussed and the groups to be recruited (based on Task 2.3.2 and Task 2.3.3) [month 5]

D2.4) A report with input and recommendations to be used for the development of the intervention: (based on Task 2.3) [month 8]

D2.5) A scientific paper submitted on the systematic review: Full title: A scientific paper submitted in a peer reviewed journal on the systematic review on the most important energy balance behaviours that are associated with overweight and obesity in young children (identifying risk groups based on family demographics, socioeconomic indices and parental BMI) (based on Task 2.1) [month 32]

D2.6) A scientific paper submitted on the secondary analyses: Full title: A scientific paper submitted in a peer reviewed journal on the secondary analyses on the most important energy balance behaviours associated with overweight and obesity of young children in European member states (identifying risk groups based on family demographics, socioeconomic indices and parental BMI) (based on Task 2.2) [month 32]

Schedule of relevant Milestones

Milestone number [59]	Milestone name	Lead beneficiary number	Delivery date from Annex I [60]	Comments
MS7	Completion of systematic literature review on identification of energy balance-related behaviours	4	8	
MS8	Completion of secondary data analyses on importance of energy balance behaviours	4	8	

WT3:
Work package description

Project Number [1]	245200	Project Acronym [2]	ToyBox

One form per Work Package

Work package number [53]	WP3	Type of activity [54]	RTD
Work package title	Identification of the determinants of most important energy-balance related behaviours		
Start month	5		
End month	32		
Lead beneficiary number [55]	3		

Objectives

3.1. Carrying out a qualitative study to identify the determinants of the behaviours found in WP2 to be related with overweight and obesity in young children
3.2. Identification of the most important parenting practices and home environmental characteristics that are associated with overweight and obesity in young children
3.3. Provision of practical input, recommendations and guidance for the development of the intervention in WP6

Description of work and role of partners

Description of work
In WP3 qualitative methods will be used to detect critical determinants of the identified specific behaviours in WP2. Focus groups interviews will be executed with parents and teachers. The results of WP3 will provide information of predisposing (e.g. children’s food preferences), reinforcing (e.g. parental and teachers’ behaviours, knowledge, beliefs and attitudes, food promotion and advertising, parental and teachers’ practices) and enabling factors (e.g. time constraints, access to safe playgrounds) determining children’s behaviours, which will be used for the development of the of the intervention material in WP6.
The few studies available on determinants in this young age group can lead us in developing the questioning routes for the qualitative research. Focus group interviews with parents and teachers are chosen for several reasons. The flexible questioning and synergetic effect of group conversations increases the likelihood that data and ideas will be produced that would remain uncovered with other methods (e.g. one on one interviews, self-reported questionnaires. By executing focus groups in the different countries it will be possible to draw conclusions about similarities and differences between countries [131]. These focus groups will furthermore be valuable to generate ideas about possible effective intervention strategies using the intervention mapping protocol [59]. As a consequence these focus groups will provide us with information of predisposing, reinforcing and enabling factors determining children’s behaviours and actual environment taking into account the views of important stakeholders such as the parents and teachers.

We hypothesize that determinants will be partly similar across European countries, partly country-specific and partly family-sociodemographic specific. All determinants identified in WP3 will be included in the intervention material to facilitate in the best possible way the local, cultural and sociodemographic needs within a European scale approach.

Task 3.1 (month 5-6) (UGent, VUmc)
Review on determinants of energy balance related behaviours
3.1.1 A literature search will be performed on determinants of the behaviours found in WP2 to be related to overweight and obesity in young children. The review will include personal (e.g. eating preferences, neophobia), social (e.g. rules about TV viewing and snacking, parental support, parents being physically active together with their children) and environmental (e.g. availability and opportunities for healthy energy balance related behaviours, safety) determinants. The results of this review will inform Task 3.2 on potential determinants that should be discussed during the focus groups interviews.

Task 3.2 (month 6-7)

Develop a protocol for the focus group research
3.2.1. Write a design and protocol for the focus group research based on the literature search. A protocol will be developed for focus groups in parents and a different protocol for focus groups in teachers.
3.2.2. Develop a country specific plan for the focus groups in parents and teachers in each country.
3.2.3. The English version of the protocol will be sent to all WP partners. The protocol will then be translated and culturally adapted for use in the six countries participating in WP6.
3.2.4. Training of the partners in executing the focus group interviews.
3.2.5. Development of a framework for data gathering and analysing the focus group interviews across countries.

Task 3.2 will be co-ordinated by Partner 3 (UGent), but all WP participants will be involved.

Task 3.3 (month 6-9)
Execution of focus groups
3.3.1 Recruitment of parents and teachers in each country: each focus group consists of 6-8 parents or 4-8 teachers, respectively. Special attention will be paid to gender, age and SES heterogeneity.
3.3.2 Conduct focus groups interviews following the protocol. At least 4 groups of parents and 3 groups of teachers will be executed per country.
3.3.3 Each country reports the results of the focus group interviews with parents and teachers using the standardised forms in English.

Task 3.3 will be co-ordinated by Partner 3 (UGent), but all WP participants (except VUmc) will be involved.

Task 3.4 (month 10-12) (UGent)
Analysis of focus groups data
3.4.1 Analyse the data according to the data analysis plan. Write reports on focus group research, for parents and teachers to include important determinants of the behaviours identified in WP2 to be related with overweight and obesity in young children and promising strategies to involve parents and teachers in school-based interventions.
3.4.2 Translate findings into recommendations for the development of the intervention (WP6).

Person-Months per Participant

Participant number [10]	Participant short name [11]	Person-months per participant
1	HUA	3.00
2	LMU-Muenchen	3.00
3	UGent	18.00
4	VUA	1.00
5	UniZar	3.00
9	CMHI	3.00
10	MUV	3.00
	Total	34.00

List of deliverables

Delive-rable Number [61]	Deliverable Title	Lead benefi-ciary number	Estimated indicative person-months	Nature [62]	Dissemi-nation level [63]	Delivery date [64]
D3.1	A protocol for the focus groups	3	1.00	O	RE	6
D3.2	A standardised form for data gathering and analyses	3	0.50	O	RE	7

List of deliverables

Delive-rable Number [61]	Deliverable Title	Lead benefi-ciary number	Estimated indicative person-months	Nature [62]	Dissemi-nation level [63]	Delivery date [64]
D3.3	A report by country on specifically identified parental determinants which need to be addressed and	3	14.50	R	RE	12
D3.4	A report from teachers' focus groups, with identified determinants by country (for WP6)	3	14.00	R	RE	12
D3.5	A scientific paper submitted in a peer-reviewed journal on the focus group research	3	2.00	O	PU	32
D3.6	A scientific paper submitted in a peer-reviewed journal on the systematic review	3	2.00	O	PU	32
		Total	34.00			

Description of deliverables

D3.1) A protocol for the focus groups: (based on Task 3.2) [month 6]

D3.2) A standardised form for data gathering and analyses: (based on Task 3.2) [month 7]

D3.3) A report by country on specifically identified parental determinants which need to be addressed and: Full title: A report by country on specifically identified parental determinants which need to be addressed and tackled in the Parental Activities Guide (WP6). Specific text, phrases on how to be communicated and addressed will be included (based on Task 3.4) [month 12]

D3.4) A report from teachers' focus groups, with identified determinants by country (for WP6): Full title: A report from teachers' focus groups, with identified determinants by country which need to be addressed and tackled in the Teacher's General Guide (WP6). Description: This report will further contain information and guidance for WP6 on issues raised by the teachers regarding their self-efficacy, information material, training and support they might need to be provided to improve their self efficacy and feel confident and motivated to deliver the intervention (based on Task 3.4) [month 12]

D3.5) A scientific paper submitted in a peer-reviewed journal on the focus group research: Full title: A scientific paper submitted in a peer-reviewed journal on the focus group research methods and findings (based on Tasks 3.2-3.4) [month 32]

D3.6) A scientific paper submitted in a peer-reviewed journal on the systematic review: Full title: A scientific paper submitted in a peer-reviewed journal on the systematic review of the most important parenting practices and home environmental characteristics that are associated with overweight obesity in young children (based on Task 2.2) [month 32]

Schedule of relevant Milestones

Milestone number [59]	Milestone name	Lead benefi-ciary number	Delivery date from Annex I [60]	Comments
MS14	Completion of focus group interviews and analyses	3	12	

WT3:
Work package description

Project Number [1]	245200	Project Acronym [2]	ToyBox

One form per Work Package

Work package number [53]	WP4	Type of activity [54]	RTD
Work package title	Review of behavioural models and educational strategies to promote healthy weight and healthy energy		
Start month	1		
End month	32		
Lead beneficiary number [55]	7		

Objectives

4.1. Identification and critically appraisal of educational strategies and psychological approaches explaining young children's acquisition and formation of energy-balance related behaviours, and facilitating their management.
4.2. Systematic review of behavioural models used in school-based interventions in pre-primary and primary education settings for the prevention of obesity
4.3. Translation of findings into practical evidence-based input for WP 6

Description of work and role of partners

WP4 will be concerned with reviewing the evidence for the effectiveness of educational strategies, and behavioural models, in relation to healthy eating, physical activity and energy-balance related behaviours of young children. Behavioural models (e.g. Social Cognitive Theory, the Socio-Ecological Model, the Theory of Planned Behaviour and the Social Marketing) provide the theoretical framework for the design and implementation of school based interventions whereas educational strategies (e.g. number of exposures to food, methods of encouragement, or 'learning to like' procedures) are practical plans of action that parents and teachers can use to help children achieve the desired behaviours.

Knowledge from the findings will then be transferred to WP6, to ensure the best evidence-based design for a school-based family involving intervention. The approach will consist mainly of two tasks:

Task 4.1 will form a critical narrative review of the literature composed of two parts: a) a review of psychological approaches (theoretical explanations and their evidence) on young children's acquisition and formation of energy-balance related behaviours, including social and biological theories of learning and motivation, and understanding individual differences in risk for obesity-related behaviours; b) a review of educational strategies used by teachers, parents and health professionals to alter these learned behaviours toward a healthy energy-balance related outcome.

Task 4.2 will form a systematic review focusing on behavioural models used as theoretical framework for the design and implementation of school based interventions (both at pre-primary and primary education settings) aiming to prevent obesity. The knowledge accumulated from these reviews will then be formulated and transferred to WP6, to ensure the best evidence-based intervention that is practicable in young children (Task 4.3).

Task 4.1 (months 1-6) (RoU, ULU, IFP)
Critical narrative review of educational strategies and psychological approaches explaining young children's acquisition and formation of energy-balance related behaviours, and facilitating their management
4.1.1. Conduct a literature search of evidence underlying psychological approaches on how young children acquire their eating and activity habits, including meal sizes and food likes and dislikes and the implications for the development of individual risk factors for obesity.
4.1.2. Conduct a literature search of evidence for the effectiveness of educational strategies (e.g. number of exposures to food, methods of encouragement) practised by parents, teachers and health professionals in order to manage young children's energy balance related behaviours.
4.1.3. Critically appraise the compatibility or otherwise of the findings from these two review processes.

4.1.4. Write the report of the reviews, including proposals for evidence-based strategies that modify children's behaviour to reduce their risk of obesity.
4.1.5. Report and transfer information, findings and recommendations to WP1.

Task 4.2 (months 1-6) (UDUR)
Systematic review of behavioural models used in school-based interventions in pre-primary and primary education settings for the prevention of obesity.
A systematic review will be conducted in the existing literature focusing on behavioural models used in school-based interventions in pre-primary and primary education settings for the prevention of obesity.

4.2.1. Develop a protocol for a systematic review of the existing literature, including the development of inclusion criteria and method of quality assessment;
4.2.2. Develop and run a search strategy to identify relevant primary studies.
4.2.3. Review abstracts of papers identified from the search (tasks 4.1.2) for possible inclusion in the review by two independent reviewers;
4.2.4. Retrieve full copies of papers that were identified for possible inclusion in the review from Task 4.1.3 and, after reading the papers, make a final decision about which studies should be included in the review;
4.2.5. Extract relevant data from studies that will be included in the review;
4.2.6. Score the methodological quality of the included studies by two independent reviewers;
4.2.7. Make decision if retrieved studies allow a meta-analytic review approach and if so conduct meta-analytic data analysis;
4.2.8. Write the report of the systematic review, including an assessment of quality of the included studies;
4.2.9. Report and transfer data and results to WP1.

Task 4.3 (months 6-8)
Translate findings into practical input for WP 6
4.3.1. Discuss with partners the findings from the systematic review (Task 4.1) and the review in Task 4.2;
4.3.2. Formulate recommendations regarding the most important components of energy balance related behavioural interventions at school setting for young children;
4.3.3. Formulate recommendations regarding the most important educational strategies for the formation of desired energy balance related behaviours in young children;
4.3.4. Make a synthesis of the recommendations formed in Tasks 4.3.2 and 4.3.3. The combined recommendations will inform the development of the intervention in WP 6.

Task 4.3 will be co-ordinated by Partner 7 (UDUR), but all WP participants will be involved in the drafting of and agreement on the recommendations.

Person-Months per Participant

Participant number [10]	Participant short name [11]	Person-months per participant
1	HUA	1.00
2	LMU-Muenchen	1.00
3	UGent	2.00
5	UniZar	1.00
7	UDUR	10.00
8	IFP	3.00
9	CMHI	1.00
10	MUV	1.00
14	RoU	2.50
15	ULU	2.50

Person-Months per Participant

Participant number [10]	Participant short name [11]	Person-months per participant
	Total	25.00

List of deliverables

Deliverable Number [61]	Deliverable Title	Lead beneficiary number	Estimated indicative person-months	Nature [62]	Dissemination level [63]	Delivery date [64]
D4.1	A report on the reviews on educational strategies and psychological approaches	14	5.00	R	PU	6
D4.2	A report on behavioural models used in school-based interventions	7	5.00	R	PU	6
D4.3	A report with recommendations to inform WP6	7	9.00	R	RE	8
D4.4	A scientific paper submitted on most important educational strategies	14	2.00	O	PU	32
D4.5	A scientific paper on the systematic review of behavioural models	7	2.00	O	PU	32
D4.6	A scientific paper on the evidence base...	7	2.00	O	PU	32
		Total	25.00			

Description of deliverables

D4.1) A report on the reviews on educational strategies and psychological approaches: Full title: A report on the reviews on educational strategies and psychological approaches explaining the acquisition and formation of energy-balance related behaviours and facilitating their management (based on Task 4.1) [month 6]

D4.2) A report on behavioural models used in school-based interventions: Full title: A report on behavioural models used in school-based interventions in pre-primary and primary education settings for the prevention of obesity (based on Task 4.2) [month 6]

D4.3) A report with recommendations to inform WP6: Full title: A report with recommendations on most important behavioural models and educational strategies for the formation of desired eating and physical activity behaviours in young children to inform WP6 (based on Task 4.3) [month 8]

D4.4) A scientific paper submitted on most important educational strategies: Full title: A scientific paper submitted in a peer-reviewed journal on most important educational strategies and psychological approaches explaining the acquisition and formation of energy-balance related behaviours and facilitating their management (based on Task 4.1) [month 32]

D4.5) A scientific paper on the systematic review of behavioural models: Full title: A scientific paper on the systematic review of behavioural models used in school-based interventions in pre-primary and primary education settings for the prevention of obesity (based on Task 4.2) [month 32]

D4.6) A scientific paper on the evidence base...: Full title: A scientific paper on the evidence base, available and emerging, to help inform policy makers across Europe develop and implement strategies to prevent childhood obesity (based on Task 4.3) [month 32]

Schedule of relevant Milestones

Milestone number [59]	Milestone name	Lead beneficiary number	Delivery date from Annex I [60]	Comments
MS3	Completion of systematic literature review on behavioural models	7	6	
MS4	Completion of narrative literature review on educational strategies	7	6	

WT3:
Work package description

Project Number [1]	245200	Project Acronym [2]	ToyBox

One form per Work Package

Work package number [53]	WP5	Type of activity [54]	RTD
Work package title	Contextual and legislation framework of pre-primary education and school-based health promotion		
Start month	2		
End month	40		
Lead beneficiary number [55]	12		

Objectives

5.1. Identification of legislation and policies for pre-primary education settings in the participation countries.
5.2. Overview of existing health promotion activities in pre-primary education at a local, regional, national and European level and to identify the role of existing stakeholders
5.3. Identification of obstacles and success factors for developing and implementing health promotion policies and programmes in pre-primary education settings taking into account the cross-cultural aspects of school policies and systems.
5.4. Outline of strategies and recommendations for improving the development and implementation of interventions based at pre-primary education settings

Description of work and role of partners

Schools as well as school-based health promotion activities, food services and personnel function under a given contextual and legislation framework which varies at a local and national level. This contextual framework as well pre-primary education personnel (key stakeholders) are important moderators and mediators of any intervention applied in the pre-primary education setting since they will be the actual facilitators of the intervention.
In WP5 existing legislation and policies with regard to the above will be identified. In addition, existing initiatives and programmes concerning healthy eating and physical activity in pre-primary education settings (for age group 4-6 years) but also obstacles and success factors that influence the development and implementation of these programmes will be overviewed in each country participating in WP6.

Task 5.1 (month 2-6) (NIGZ, HiOA)
Overview of policies and regulations in pre-primary school settings in Europe
5.1.1. Development of a framework for data gathering and analysing of policies and legislations across countries (such as the national school curriculum, number of children per teacher, bans on the sales of sweets and soft drinks in kindergartens, local regulations on banning sweet shops in the vicinity of the kindergarten, creating safe walking and cycling tracks to kindergarten, food promotion and advertising in kindergarten setting, etc);
5.1.2. Cross-cultural aspects of pre-primary policies and regulations will be recorded and analysed on a national, regional and local level. The standardized form developed in 5.1.1 will be used for the collection of the data;
5.1.3. Write a report identifying these policies at a European scale.
5.1.4. Report and transfer data and results to WP1.

Task 5.2 (month 2-8) (UGent, NIGZ)
Overview of health promotion activities at pre-primary school settings in Europe and identification of the contextual factors affecting their success
5.2.1. Development of a framework for data gathering and analysing of policies and practices regarding health promotion activities in pre-primary education (such as criteria for a teacher to implement health promotion activities, material implemented, timeframe of implementation, etc);
5.2.2. Implementation of this standardized form to retrieve data on policies and practices regulating health promotion activities in pre-primary education;
5.2.3. NIGZ will make an overview of existing health promotion activities in pre-primary education at a local, regional, national level, making use of the 'HEPS inventory tool' (available October 2009). The tool is developed as part of the HEPS project (3 year project on developing and implementing healthy eating and

physical activity in pre-primary education settings, period 2008-2011, funded by DG Sanco and led by NIGZ, www.hepseurope.eu) and consists of a set of quality criteria for school based interventions. These criteria will include demonstrated effectiveness, systematic whole school approach, efficiency of the programme, suitability for education and use in the classroom, level of participation and diversity;
5.2.4. Obstacles and success factors will be identified by UGent for the development and implementation of health promotion activities in school setting (as identified in Task 5.2.1) taking into account the cross-cultural aspects of school policies and systems. Additional analysis will be carried out for the target group with specific reference to who approves the implementation of health promotion programmes, who funds them, who implements them, who is monitoring or evaluating them, who are the potential stakeholders, which are the inhibiting and promoting factors, which factors ensure sustainability of the programme and what cross-cultural aspects of school policies and systems can be identified;
5.2.5. A report on ongoing health promotion activities in pre-primary education settings at local, regional and national level (NIGZ);
5.2.6. A report on most important contextual factors affecting success of health promotion activities in pre-primary education settings on a European scale (UGent).
5.2.7. Report and transfer data and results to WP1.

Task 5.3 (month 8-9) (NIGZ, HiOA, UGent)
Outline recommendations for the development and implementation of interventions at school setting
5.3.1. Identify good examples of policies and health promotion activities in the school setting among the ones collected in Tasks 5.1 and 5.2 as well as through the European Network for Nutrition and Physical Activity. These will be presented in a report form.
5.3.2. To outline strategies and recommendations for improving the development and implementation of interventions at pre-primary education settings.
5.3.3. To outline a blueprint on what should be reported in a scientific paper presenting an intervention study.
5.3.4. Development of a report with successful and sustainable interventions for pre-primary education settings, leading to a blueprint for the core components of the ToyBox, including recommendations for national implementation.

Task 5.3 will be co-ordinated by Partner 12 (NIGZ), but all WP participants will be involved.

Person-Months per Participant

Participant number [10]	Participant short name [11]	Person-months per participant
1	HUA	1.00
2	LMU-Muenchen	1.00
3	UGent	9.00
5	UniZar	1.00
6	HiOA	10.00
9	CMHI	1.00
10	MUV	1.00
12	NIGZ	18.00
	Total	42.00

List of deliverables

Delive-rable Number [61]	Deliverable Title	Lead benefi-ciary number	Estimated indicative person-months	Nature [62]	Dissemi-nation level [63]	Delivery date [64]
D5.1	A standardized form for data gathering and analysing of policies and legislations	12	1.50	O	RE	4
D5.2	A standardized form for data gathering and analysing of health promotion activities	12	1.50	O	RE	6
D5.3	Report on existing policies and regulations applying in pre-primary education	12	6.00	R	PU	6
D5.4	Report on health promotion activities policy and legislations	12	6.00	R	PU	8
D5.5	Report on ongoing health promotion activities	12	6.00	R	PU	8
D5.6	Report on most important contextual factors affecting success	3	6.00	R	PU	8
D5.7	Report on good examples of policies and health promotion activities in the school setting	12	3.00	R	PU	9
D5.8	Blueprint for strategies and core components for the development of the ToyBox	12	1.50	O	RE	9
D5.9	Blueprint on what should be reported in a scientific paper presenting an intervention study	12	1.50	O	PU	9
D5.10	A scientific paper submitted on existing health promotion activities	12	3.00	O	PU	40
D5.11	A scientific paper submitted on obstacles and success factors for developing and implementing	12	3.00	O	PU	40
D5.12	A scientific paper submitted on strategies and recommendations for policy makers	12	3.00	O	PU	40
		Total	42.00			

Description of deliverables

D5.1) A standardized form for data gathering and analysing of policies and legislations: Full title: A standardized form for data gathering and analysing of policies and legislations at pre-primary education settings (based on Task 5.1.1) [month 4]

D5.2) A standardized form for data gathering and analysing of health promotion activities: Full title: A standardized form for data gathering and analysing of health promotion activities policy and legislations at pre-primary education settings (based on Task 5.2.1) [month 6]

D5.3) Report on existing policies and regulations applying in pre-primary education: Full title: Report on existing policies and regulations applying in pre-primary education settings at local, regional and national level (based on Task 5.1.3) [month 6]

D5.4) Report on health promotion activities policy and legislations: Full title: Report on health promotion activities policy and legislations in pre-primary education settings at local, regional and national level (based on Task 5.2.2) [month 8]

D5.5) Report on ongoing health promotion activities: Full title: Report on ongoing health promotion activities in pre-primary education settings at local, regional and national level (based on Task 5.2.3) [month 8]

D5.6) Report on most important contextual factors affecting success: Full title: Report on most important contextual factors affecting success of health promotion activities in pre-primary education settings in Europe (based on Task 5.2.4) [month 8]

D5.7) Report on good examples of policies and health promotion activities in the school setting: (based onTask 5.3) [month 9]

D5.8) Blueprint for strategies and core components for the development of the ToyBox: Full title: Blueprint for strategies and core components for the development of the ToyBox intervention material in WP6 (based on Task 5.3) [month 9]

D5.9) Blueprint on what should be reported in a scientific paper presenting an intervention study: (based on Task 5.3) [month 9]

D5.10) A scientific paper submitted on existing health promotion activities: Full title: A scientific paper submitted in a peer reviewed journal on existing health promotion activities in pre-primary education settings at a local, regional, national and European level identifying the role of existing stakeholders (based on Task 5.2.3) [month 40]

D5.11) A scientific paper submitted on obstacles and success factors for developing and implementing: Full title: A scientific paper submitted in a peer reviewed journal on obstacles and success factors for developing and implementing health promotion policies and programmes in pre-primary education settings (based on Task 5.2.4) [month 40]

D5.12) A scientific paper submitted on strategies and recommendations for policy makers: Full title: A scientific paper submitted in a peer reviewed journal on strategies and recommendations for policy makers for improving the development and implementation of school-based health promotion activities for pre-primary education in Europe [month 40]

Schedule of relevant Milestones

Milestone number [59]	Milestone name	Lead beneficiary number	Delivery date from Annex I [60]	Comments
MS5	Report on existing policies and legislations at pre-primary education settings	12	6	
MS9	Report on health promotion activities and legislations at pre-primary education settings	12	8	
MS10	Report on contextual factors at pre-primary education settings	12	8	
MS11	Report on good examples of Policies and health promotion activities at pre-primary education setting	12	9	

Project Number [1]	245200	Project Acronym [2]	ToyBox

One form per Work Package

Work package number [53]	WP6	Type of activity [54]	RTD
Work package title	Development of school-based family involved intervention programme applicable on a European scale		
Start month	6		
End month	46		
Lead beneficiary number [55]	2		

Objectives

6.1. Development of an intervention programme and relevant material for young children to be applied at the school setting in different European countries.
6.2. Development, production and adaptation of the material in the different languages.
6.3. Provision to the research centres, implementing the intervention in WP7, of the developed material, all necessary training and guidance on how to transfer this knowledge and expertise in the local schools and teachers.

Description of work and role of partners

In this WP, a multi-component school-based family-involved intervention will be produced. The experience gained by the three partners (LMU, IFP, AOK-Verlag) who have developed, implemented and evaluated the TigerKids project in Germany as well as some of the TigerKids material will be used as a basis for the ToyBox intervention material. However all this material will be extensively revised and upgraded to incorporate the input and findings from WPs 2-5, aiming to facilitate in the best possible way local, cultural and sociodemographic needs within a European scale approach (WPs 2-3). Furthermore the intervention will be enriched with behavioural models, educational strategies and 'how to implement' procedures based on the best practices identified by the literature reviews, the local policy and contextual framework regarding pre-primary education in the participating countries (WPs 4-5). As soon as the English version has been completed, it will be sent to the participating countries to be translated, culturally adapted and tested for suitability and acceptability. During this process, pre-primary teachers and representatives from national Educational Councils (or other equivalent Entities) will be engaged to ensure acceptability of the ToyBox material and intervention programme and increase their support throughout the implementation period (WP7).

The TigerKids material was chosen to serve as the basis for the ToyBox intervention material. No person-months have been allocated for the translation of the TigerKids material into English since this is a deliverable of an ongoing Grant by RoU.

The ToyBox material will consist of a) Teacher's General Guide, b) Classroom Activities Guide and c) Parental Activities Guide. All printed material as well as a wooden train for the structural exploration of food, food models, board games, a hand-puppet, physical activity equipment, etc. will be included in a box, specifically designed for the project (ToyBox).

Task 6.1 (month 6-27)
Development of the ToyBox Teacher's General Guide
6.1.1. The Teacher's General Guide will provide an overview of the programme and its objectives and some general information and background knowledge regarding the activities implemented in the school setting. The material will be based on the most effective behavioural models and educational strategies (identified in WP4) aiming to address and tackle children's energy balance related behaviours and their determinants as identified in WPs 2 and 3.
6.1.2. The final version of the ToyBox information material for the teacher as prepared in Task 6.2.1 will be translated and culturally adapted for all participating countries (Bulgaria, Belgium, Germany, Greece, Poland, Spain). During local adaptation period, engaging local stakeholders to increase acceptability of the ToyBox material and intervention programme.

6.1.3. Prior to final printings (Task 6.1.4), each country will pilot-test the produced material
6.1.4. Printing of the material and delivery in all participating countries

Task 6.1 will be co-ordinated by Partner 2 (LMU), but all WP participants will be involved.

Task 6.2 (month 6- 27)
Development of the ToyBox Classroom Activities Guide
6.2.1. The ToyBox Classroom Activities Guide based on the input and the results obtained by WPs 2-4. This material is estimated to include two or three books with activities for use in the classroom, a wooden train for the structural exploration of food, food models, board games, a hand-puppet, physical activity equipment, etc. All this will be included in a box, specifically designed for the project (ToyBox).
6.2.2. The final version of the ToyBox material for each country as prepared in Task 6.2.1 will be translated and culturally adapted for all participating countries (Bulgaria, Belgium, Germany, Greece, Poland, Spain). During local adaptation period, engaging local stakeholders to increase acceptability of the ToyBox material and intervention programme.
6.2.3. Printing of the material and delivery of both printed material and ToyBox in all participating countries.

Task 6.2 will be co-ordinated by Partner 2 (LMU), but all WP participants will be involved.

Task 6.3 Development of the ToyBox Parental Activities Guide (month 12-27)
6.3.1. The Parental Activities Guide of the ToyBox intervention will be based on the input and results obtained in WPs 2, 3 and 4. WP2 will provide information on specific sub-behaviours that should be addressed whereas WP4 will inform on educational strategies both to motivate parents to adopt desired behaviours but also how parents that parents could help their children form desired behaviours. The results of WP3 will also be included to address the specific determinants (parental and home environmental) identified at a European scale to affect young children's behaviour. Specific strategies and methods will be provided to the parents to overcome barriers and enhance facilitators of healthy energy-balance related behaviours in young children.
6.3.2. The final version of the ToyBox Parental Activities Guide for each country as prepared in Task 6.4.1 will be translated and culturally adapted for all participating countries (Bulgaria, Belgium, Germany, Greece, Poland, Spain). During local adaptation period, engaging local stakeholders to increase acceptability of the ToyBox material and intervention programme.
6.3.3. Printing of the material and delivery in all participating countries.

Task 6.3 will be co-ordinated by Partner 2 (LMU), but all WP participants will be involved.

Task 6.4 (25-27)
Training of the research teams
6.4.1. Concepts and strategies to how to train and motivate teachers in the different settings will be developed.
6.4.2. Research members from all WP participants (HUA, LMU, UGent, UniZar, CMHI, MUV) will be trained on how to educate teachers on the implementation of the ToyBox programme. The trained researchers will be further responsible for educating the teachers, who will actually implement the intervention in the class and with the parents at a local level in WP7.

Task 6.4 will be co-ordinated by Partner 2 (LMU), but all WP participants will be involved.

Task 6.5. (month 26-31)
Support of the intervention via the website
Internet material will be developed and put online in the languages of the participating countries to support the programme. This will also include a brief guide on the intervention, the actual ToyBox material and Frequently Asked Questions (FAQs). Initially, only teachers participating in the programme will have access to them, so as to avoid contamination of the control group. After the end of the intervention, the material will be accessible to the public and additional activities, attractive to children, will be available.

Task 6.5 will be co-ordinated by Partner 2 (LMU), but all WP participants will be involved.

Task 6.6. (month 30-46)
Revise the ToyBox material based on the input obtained of the intervention during the implementation phase
During the implementation of the ToyBox intervention all input received locally regarding issues not well understood or addressed by the material, missing or confusing information etc will be constantly monitored.

This input along with the process evaluation throughout the implementation of the intervention (WP7) will guide LMU to revise and further improve the material to accommodate additional local input on this joint European attempt. A report on any changes made to the ToyBox material will be made by country.

Task 6.6 will be co-ordinated by Partner 2 (LMU), but all WP participants will be involved.

Person-Months per Participant

Participant number [10]	Participant short name [11]	Person-months per participant
1	HUA	7.00
2	LMU-Muenchen	32.00
3	UGent	9.00
4	VUA	2.00
5	UniZar	7.00
7	UDUR	2.00
8	IFP	12.00
9	CMHI	7.00
10	MUV	7.00
13	AOK-Verlag	19.00
14	RoU	2.00
	Total	106.00

List of deliverables

Delive-rable Number [61]	Deliverable Title	Lead benefi-ciary number	Estimated indicative person-months	Nature [62]	Dissemi-nation level [63]	Delivery date [64]
D6.1	ToyBox Teacher’s General Guide and ToyBox Classroom Activities Guide developed in Engllish	2	10.00	O	RE	21
D6.2	ToyBox Teacher’s General Guide and ToyBox Classroom Activities Guide - Translated and adapted in 6	2	18.00	O	RE	23
D6.3	ToyBox Parental Activities Guide-Developed in English	2	10.00	O	RE	21
D6.4	ToyBox Parental Activities Guide-Translated and adapted for use in six countries	2	15.00	O	RE	21
D6.5	All material printed and CD-ROMs with presentations prepared and ready for use for all six countries	2	6.00	O	RE	24
D6.6	Training modules for teachers in participating countries developed	2	12.00	O	RE	27

List of deliverables

Delive-rable Number [61]	Deliverable Title	Lead benefi-ciary number	Estimated indicative person-months	Nature [62]	Dissemi-nation level [63]	Delivery date [64]
D6.7	The total set of ToyBox printed material and toys delivered in all participating countries	2	10.00	O	RE	27
D6.8	Internet material developed and put online to support the programme, in the languages	2	10.00	O	RE	31
D6.9	Final versions of ToyBox material in all languages-material for classroom-report on changes	2	15.00	O	PU	46
		Total	106.00			

Description of deliverables

D6.1) ToyBox Teacher’s General Guide and ToyBox Classroom Activities Guide developed in Engllish: (based on Tasks 6.1 and 6.2) [month 21]

D6.2) ToyBox Teacher’s General Guide and ToyBox Classroom Activities Guide - Translated and adapted in 6: Full title: ToyBox Teacher’s General Guide and ToyBox Classroom Activities Guide- Translated and adapted for use in six countries (based on Tasks 6.1 and 6.2) [month 23]

D6.3) ToyBox Parental Activities Guide- Developed in English: (based on Task 6.3) [month 21]

D6.4) ToyBox Parental Activities Guide- Translated and adapted for use in six countries: (based on Task 6.3) [month 21]

D6.5) All material printed and CD-ROMs with presentations prepared and ready for use for all six countries: All material refers to Teacher’s General Guide, Classroom Activities Guide and Parental Activities Guide (based on Tasks 6.1, 6.2, 6.3) [month 24]

D6.6) Training modules for teachers in participating countries developed: (based on Task 6.4) [month 27]

D6.7) The total set of ToyBox printed material and toys delivered in all participating countries: (based on Tasks 6.1-6.3) [month 27]

D6.8) Internet material developed and put online to support the programme, in the languages: Full title: Internet material developed and put online to support the programme, in the languages of the participating countries. (based on Task 6.5) [month 31]

D6.9) Final versions of ToyBox material in all languages-material for classroom-report on changes: Full title: Final versions of ToyBox material in all languages (incl. English) and ToyBox with material for classroom activities (Tasks 6.1-6.6) and report on any changes made to the ToyBox material by country (Task 6.6) See Annex I: List of deliverables for the detailed information on level of dissemination [month 46]

Schedule of relevant Milestones

Milestone number [59]	Milestone name	Lead benefi-ciary number	Delivery date from Annex I [60]	Comments
MS16	Intervention materials ready for the intervention study and delivered to participating countries	2	27	

Project Number [1]	245200	Project Acronym [2]	ToyBox

One form per Work Package

Work package number [53]	WP7	Type of activity [54]	RTD
Work package title	Implementation and evaluation of a cluster randomized intervention		
Start month	20		
End month	50		
Lead beneficiary number [55]	1		

Objectives

7.1 Development and implementation of an intervention procedure
7.2 Evaluation of the process of the intervention to ensure that the intervention is implemented as intended
7.3 Evaluation of the effectiveness of the intervention in terms of impact and outcome changes.

Description of work and role of partners

A cluster randomized intervention will be implemented in six different European countries, namely Greece, Spain, Belgium, Germany, Poland, Bulgaria. Comprehensibility, readability, relevance, credibility, attractiveness of the intervention will be tested, so that the intervention components can be adapted if necessary. Furthermore, it will be tested whether the intervention induces changes in important energy balance behaviours and their determinants.
The target of this intervention will be children (4-6 year olds), their parents and the teachers. The intervention will be implemented in the pre-primary education setting by teachers and will be tested in a pre-test post-test design including an intervention and a control group. The pre- and post intervention period is estimated to last two months and in order to avoid seasonality effect, the pre- and post-examination will take place at the same time of the year leaving 9-10 months in between for implementation. Only 4-5 years old (with a parental signed consent form) will be examined at baseline and only these children will be re-examined at the follow up, about a year later.
From each country 20 pre-primary education schools with 2 classes each (20 children per class on average) will participate in the evaluation study, resulting in about 4800 participating children on a European level. Power calculations based on previous studies of school-based interventions indicates that this sample size is sufficient to detect changes in energy balance behaviours and their determinants.
The schools will be assigned randomly as intervention or controls in a 2:1 ratio. The control group will not receive any intervention materials but will continue to follow the standard curriculum. Schools will be the level of randomisation in order to prevent contamination of intervention activities. This WP will also be responsible for the coordination of data entry of pre- and post-intervention evaluation.

Task 7.1 (month 23-28)
Train teachers implementing the intervention
7.1.1. Develop a protocol for the teachers' training.
7.1.2. Coordinate the training of the teachers who will implement the intervention using the researchers trained in WP6.

Task 7.1 will be co-ordinated by Partner 1 (HUA), but all WP participants will be involved.

Task 7.2 (month 22-40)
Conduct the cluster randomized intervention
7.2.1. Write a study protocol for ensuring standardized school and parent recruitment, questionnaire administration, data collection, data entry and data management
7.2.2. Obtain approvals from Ministry or local authorities to contact and enter the schools.
7.2.3. Recruit randomly 20 schools per participating country for inclusion in the evaluation study; explain the purpose of the study and the content of the intervention. Classes will be randomly selected within predefined regions to maximise representativeness of the overall population but also to ensure high risk groups (e.g. families with certain socio-demographic characteristics (as identified in WP2) will be included.

7.2.4. Assign randomly these 20 schools as intervention or controls in a 2:1 ratio.
7.2.5. Implement the intervention at the participating schools assigned to the intervention group in six countries (Greece, Spain, Belgium, Germany, Poland, Bulgaria)

Task 7.2 will be co-ordinated by Partner 1 (HUA), but all WP participants will be involved.

Task 7.3 (month 21-45)
Process evaluation
This will be assessed by questionnaires directed to parents, teachers and school boards in the intervention schools to assess easiness of implementation. It will try to assess whether the intervention was implemented in the way it was intended, if it was adjusted/ changed along the way, what were the experiences/responses of those who were implementing the intervention and how the collaboration functioned. Partnership working between the relevant agencies will be specifically monitored and mapped during the course of this project. The results of WP5 will inform this Task of specific areas that should be addressed during the process evaluation.
7.3.1. Identify appropriate evaluating tools (i.e. questionnaires to assess process measures, i.e. intelligibility, credibility, readability, relevance, attractiveness of the intervention tools) to be used by parents, teachers and school boards
7.3.2. Translate and back-translate all process measures tools in the six corresponding languages.
7.3.3. Request teachers to complete logbooks and questionnaires assessing process measures (i.e. context, reach, dose delivered, dose received, fidelity, implementation and recruitment) during the 9 months that the intervention is implemented at the schools.
7.3.4. Conduct a process evaluation at a country and European level.
7.3.5. Development of databases for data entry.
7.3.6. Coordination of data entry.
7.3.7. Clean all data according to standardised protocols;
7.3.8. Executing the analyses and write report on the findings.
7.3.9. Report and transfer data and results to WP1 for storage and overall data management.

Task 7.3 will be co-ordinated by Partner 1 (HUA), but all WP participants will be involved.

Task 7.4 (month 23-46)
Impact and outcome evaluation
We will evaluate whether the intervention is having the intended impact on the behavioural and environmental factors it is aimed at, and adjust accordingly. The impact evaluation will be based on pre- and post-changes on children's diet, sedentary and physical activity behaviours, predisposing and enabling factors related to home and school social and physical environment, parental eating and physical activity behaviours and parental self reported BMI. The outcome evaluation will be based on pre- and post-changes on children's weight, BMI and waist circumference as well as overweight and obesity prevalence. All the tools for the impact and outcome evaluation will be developed in WP8.
Both impact and outcome evaluation will be used to assess programme’s effectiveness and add to the evidence about what works (and doesn't work) and help in supporting decision making in Public Health Policy.
7.4.1. Obtain informed consent forms from the parents of participating children.
7.4.2. Tools developed in WP8 will be implemented in the pre- and post-intervention evaluation.
7.4.3. Coordination of data entry (databases will be provided by WP8).
7.4.4. Clean all data according to standardised protocols;
7.4.5. Executing the analyses and write report on the findings.
7.4.6. Report and transfer data and results to WP1 for storage and overall data management.

Task 7.4 will be co-ordinated by Partner 1 (HUA), but all WP participants will be involved.

Person-Months per Participant

Participant number [10]	Participant short name [11]	Person-months per participant
1	HUA	36.00
2	LMU-Muenchen	22.00
3	UGent	22.00

Person-Months per Participant

Participant number [10]	Participant short name [11]	Person-months per participant
5	UniZar	22.00
9	CMHI	20.70
10	MUV	22.00
	Total	144.70

List of deliverables

Deliverable Number [61]	Deliverable Title	Lead beneficiary number	Estimated indicative person-months	Nature [62]	Dissemination level [63]	Delivery date [64]
D7.1	A protocol for the implementation and evaluation of the ToyBox intervention	1	8.00	O	RE	25
D7.2	Process evaluation tools developed	1	6.00	O	RE	26
D7.3	Report on the process, impact and outcome evaluation at a country and European scale	1	124.70	R	PU	48
D7.4	Report on mediators and moderators affecting the magnitude of the intervention	1	6.00	R	PU	50
		Total	144.70			

Description of deliverables

D7.1) A protocol for the implementation and evaluation of the ToyBox intervention: (based on Task 7.2) [month 25]

D7.2) Process evaluation tools developed: (based on Task 7.3) [month 26]

D7.3) Report on the process, impact and outcome evaluation at a country and European scale: (based on Task 7.3) [month 48]

D7.4) Report on mediators and moderators affecting the magnitude of the intervention: Full title: Report on mediators and moderators affecting the magnitude of the intervention at a local and European level to support decision making in Public Health Policy (based on Tasks 7.3 and 7.4) [month 50]

Schedule of relevant Milestones

Milestone number [59]	Milestone name	Lead beneficiary number	Delivery date from Annex I [60]	Comments
MS20	Completion of evaluation of the intervention	1	46	

WT3:
Work package description

Project Number [1]	245200	Project Acronym [2]	ToyBox

One form per Work Package

Work package number [53]	WP8	Type of activity [54]	RTD
Work package title	Development, validation and training for the outcome and impact assessment tools		
Start month	1		
End month	36		
Lead beneficiary number [55]	5		

Objectives

8.1 Development of assessment tools and procedures for the outcome and impact evaluation in WP7.
8.2 Testing the validity of developed tools and procedures
8.3 Training of the researchers participating in outcome and impact evaluation in WP7.

Description of work and role of partners

As part of the evaluation framework (WP7), data will be collected on height, weight, waist circumference (end outcomes) and most important energy balance related behaviours (eating, sedentary and physical activity behaviours) and their determinants as identified in WP2 and WP3 respectively. This WP will be responsible for the development, validity testing and training in use of tools to assess all above mentioned variables.
All the partners included in this WP will obtain the data using the tools described below. Translation of the questionnaires into 6 local languages (Spanish, German, Greek, Dutch, Polish and Bulgarian) will be performed by all the partners included in this WP.

Task 8.1. (month 1-8)
Review of the literature on tools assessing food intake and physical (in)activity to assess the impact of childhood obesity prevention strategies in Europe
Conduct a literature search on tools assessing food intake and physical (in)activity in childhood and identify those most appropriate to use in intervention studies, sensitive enough to detect pre- and post-intervention differences.

Task 8.2. (month 8-26)
Children’s food intake and eating behaviours assessment
A short food frequency questionnaire (FFQ) will be used to assess pre- and post-intervention consumption of specific food groups. This FFQ will be based on previously developed questionnaires and will be adapted to include important food groups or food items identified in WP2. Liaison will be created with ongoing FP6 projects, in which dietary assessment in children is an explicit research topic and from which the ToyBox project could benefit- IDEFICS (http://www.ideficsstudy.eu) and EFCOVAL (http://www.efcoval.org). Furthermore, based on the eating behaviours identified in WP2, some additional questions will be added to form the eating behaviours questionnaire. Both the FFQ and the eating behaviours questionnaire will be completed by parents/guardians. A validity test will be executed prior to the implementation in a small cohort in each country.

Task 8.3 will be co-ordinated by Partner 5 (UniZar), but all WP participants will be involved.

Task. 8.3. (month 8-28)
Children’s physical activity and sedentary behaviours assessment
8.3.1. Based on the findings of literature and research experience of the ToyBox consortium, proxy questionnaires (completed by parents) do not provide reliable data on young children’s (<6 year olds) physical activity levels. A good tool to obtain accurate physical activity level estimates at this young age is accelerometry; however high cost, time constraints, need for extensive training of staff and for downloading the data regularly in a pc, does not make this method an easily applicable one, in large scale studies.
For this reason, physical activity will be measured with the use of pedometers, since it has been shown that daily step counts in preschool children give valid information on physical activity levels (compared to accelerometry

data) [132]. Step counts will be assessed using the Omron Walking Style Pro Pedometer which is most technologically advanced and can give information on physical activity during the different day sections
.
Before the pedometers are handed out, they will all be reset to zero and checked for proper fit and function. A pedometer will be fastened to the waistband of each pupil's pants or skirt. Children will be instructed to wear the pedometer on the left hip, in line with the midpoint of the left knee.
A researcher in collaboration with the teacher and parents will ensure that steps are registered twice a day, once when the child leaves the school setting and once before going to bed. The first registry will provide data on physical activity performed in school setting and the second on physical activity performed at home or at leisure time. During weekend days, only one registry will be obtained, before children go to bed. Measurements will be obtained for both weekdays and weekend days for all children. All teachers will be informed of the procedure and proper instrument use. A parents' informational letter will include instructions for proper instrument use. Parents will be asked to have their children wear the instruments for as long as possible during all waking hours, removing them only for water-based activities and sleeping. A diary will be completed by the parents regarding time periods and occasions that the pedometer was removed (e.g. swimming).
8.3.2. Sedentary behaviours (e.g. watching television, using a computer, playing electronic and board games, reading or painting) and organized activities (i.e. extra-curricular physical activity) identified in WP2 will be assessed by using a questionnaire. In the questionnaire the parents will report the physical activity behaviour of their child based on specific behaviours such as: walking or cycling for transportation together with their child, walking or cycling for leisure together with their child (child also walks or cycles him/her self), membership of sports club (swimming, soccer, gymnastics, ...), active play with their child outdoors, ... The outcome will be reported in minutes of activity per day.
A liaison will be created with an ongoing FP6 project, in which sedentary behaviours in pre-pubertal children is an explicit research topic and from which the new proposed project could benefit – IDEFICS (http://www.ideficsstudy.eu). Validity of the sedentary behaviours questionnaire will be checked using a diary that the parents will fill in during one week. Results from the questionnaire will be compared with those obtained with the diary.

Task 8.3 will be co-ordinated by Partner 5 (UniZar), but all WP participants will be involved.

Task 8.4. (month 8-28)
Anthropometric measurements
For anthropometric measurements children will be barefooted and in their underwear or bathing suit. The following measures will be taken: Body weight (kg): using an electronic scale (Seca), precision 100 g, range 0–200 kg. Height (cm): using a precision stadiometer (Seca), precision 0,2 cm, range 70–200 cm. The subjects stands straight in an upright position, the feet together, knees straight, heels, buttocks and back must touch the back part of the stadiometer. The head must be in a position in order the Frankfort line is horizontal. The arms have to hang relaxed on the side of the body, the inner part of the hand faced to the thigh. The mobile, horizontal part of the stadiometer must touch the head of the subject, with a light pressure on the hair. Waist circumference will be measured with an unelastic tape, precision 0.1 cm, range 0–150 cm, the subject in a standing position, the tape is applied horizontally midway between the lowest rib margin and the iliac crest about the level of the umbilicus, at the end of gentle expiration. Prior to the implementation of the first survey, all the measurements will be tested for intra- and inter-observer reliability in a small children's sample.

Task 8.4 will be co-ordinated by Partner 5 (UniZar), but all WP participants will be involved.

Task.8.5. (month 11-26)
Assessment of social and physical environmental determinants
This Task will develop the tools for the assessment of changes in the social and physical environmental determinants (identified in WP3) of the energy balance related behaviours under study (identified in WP2). The tools will be based on previous instruments and on the major theories of behaviour change (Theory of Planned Behaviour, ANGELO model).
Psychosocial correlates may include: knowledge, beliefs, attitudes, perceptions, social variables, practices and rules, self-efficacy, perceived benefits and perceived barriers, among potential variables detected in WP3.
Environmental correlates will include physical environments, economical factors, policies and regulations related to the target behaviours at home, at school and in the neighbourhood of the child, among potential other environmental variables detected in WPs 3.
These questionnaires will be completed by the parents and the teachers.

8.5.1. Develop a questionnaire to be completed by parents. It will include a) psychosocial correlates for themselves, b) psychosocial correlates for their child and c) environmental correlates regarding home and neighbourhood.
8.5.2. Develop a questionnaire to be completed by teachers. It will include a) psychosocial correlates for themselves and b) environmental correlates regarding classroom and school setting.
8.5.3. All tools (Tasks 8.5.1-8.5.2) will be translated and back-translated in the languages of the countries conducting the intervention and will be used in the pre- and post- intervention evaluation measurements in WP7. In a preliminary phase, test-retest reliability for this questionnaire will be analysed.

Task 8.5 will be co-ordinated by Partner 5 (UniZar), but all WP participants will be involved.

Task 8.6. (month 16-26)
Training of staff involved in the measurements
A detailed protocol for planning, training and data collection on children's food intake and eating behaviours, physical activity and sedentary behaviours and anthropometry will be made. Similar procedures will be followed regarding social and physical environmental determinants.
A training workshop will be organized previous to the pre- and post-intervention measurements (WP7) for the training of researchers in all assessment tools and procedures. Field workers will be trained in all the procedures described in and a quality control protocol will be set up in each country.

Task 8.6 will be co-ordinated by Partner 5 (UniZar), but all WP participants will be involved.

Task 8.7. (month 8-28)
Quality control
Centralized supervision of translations of questionnaires, protocols, standard operating procedures will be performed by UniZar. Verification of correctness by specific procedures, like cross-checking, back-translations, etc. will also be performed. A protocol for the collection of study data from the participating centres with an agreed format and ensuring security and confidentiality of the data will be developed. Databases for data entry will be developed for all tools described in Tasks 8.2 to 8.5 and a manual of data entry procedures will be developed.

Task 8.7 will be co-ordinated by Partner 5 (UniZar), but all WP participants will be involved.

Person-Months per Participant

Participant number [10]	Participant short name [11]	Person-months per participant
1	HUA	1.00
2	LMU-Muenchen	1.00
3	UGent	2.00
4	VUA	1.00
5	UniZar	18.00
9	CMHI	2.80
10	MUV	1.00
	Total	26.80

List of deliverables

Delive-rable Number [61]	Deliverable Title	Lead benefi-ciary number	Estimated indicative person-months	Nature [62]	Dissemi-nation level [63]	Delivery date [64]
D8.1	A report on the review of the literature on tools assessing food intake and physical (in)activity	5	2.00	R	PU	10
D8.2	Protocol(s) for planning, training and data collection	5	9.80	O	RE	26
D8.3	A protocol for the collection of study data from the participating centres with an agreed format	5	7.00	O	RE	25
D8.4	Databases for data entry and manual for data entry procedures will be developed for all tools	5	2.00	O	RE	28
D8.5	A scientific paper submitted in a peer reviewed journal on the review of the literature on tools	5	2.00	O	PU	16
D8.6	Report on the validity and harmonization process of the tools	5	2.00	R	PU	36
D8.7	A scientific paper submitted in a peer reviewed journal on the development and validity testing	5	2.00	O	PU	36
		Total	26.80			

Description of deliverables

D8.1) A report on the review of the literature on tools assessing food intake and physical (in)activity: Full title: A report on the review of the literature on tools assessing food intake and physical (in)activity to assess the impact of childhood obesity prevention strategies in Europe (based on Task 8.1) [month 10]

D8.2) Protocol(s) for planning, training and data collection: Full title: Protocol(s) for planning, training and data collection will be made on children's food intake and eating behaviours, physical activity and sedentary behaviours and anthropometry as well as social and physical environmental determinants. (based on Tasks 8.2-8.5) [month 26]

D8.3) A protocol for the collection of study data from the participating centres with an agreed format: Full title: A protocol for the collection of study data from the participating centres with an agreed format and ensuring security and confidentiality of the data will be developed (based on Task 8.6) [month 25]

D8.4) Databases for data entry and manual for data entry procedures will be developed for all tools: Full title: Databases for data entry and manual for data entry procedures will be developed for all tools described in Tasks 8.2 to 8.5 (based on Task 8.6) [month 28]

D8.5) A scientific paper submitted in a peer reviewed journal on the review of the literature on tools: Full title: A scientific paper submitted in a peer reviewed journal on the review of the literature on tools assessing food intake and physical (in)activity to assess the impact of childhood obesity prevention strategies in Europe (based on Task 8.1) [month 16]

D8.6) Report on the validity and harmonization process of the tools: Full title: Report on the validity and harmonization process of the tools regarding food intake and eating behaviours, physical activity and sedentary behaviours and anthropometry (based on Tasks 8.2-8.7) [month 36]

D8.7) A scientific paper submitted in a peer reviewed journal on the development and validity testing: Full title: A scientific paper submitted in a peer reviewed journal on the development and validity testing of the assessment tools (based on Tasks 8.2-8.7) [month 36]

Schedule of relevant Milestones

Milestone number [59]	Milestone name	Lead beneficiary number	Delivery date from Annex I [60]	Comments
MS15	Completion of survey instruments for evaluation study	5	26	

Project Number [1]	245200	Project Acronym [2]	ToyBox

One form per Work Package

Work package number [53]	WP9	Type of activity [54]	RTD
Work package title	Evaluation of the cost-effectiveness of the intervention		
Start month	20		
End month	48		
Lead beneficiary number [55]	3		

Objectives

9.1 Measurement of the costs associated with preparing and implementing the intervention programme as described in WPs 3, 6 and 7;
9.2 Estimation of the long term costs, effects and cost-effectiveness of the intervention programme.

Description of work and role of partners

The investment associated with the programme will be measured by including data collection related to setting up the programme as explained in WP3 and 6. Also, during the programme implementation, data will be collected regarding resource use (e.g. teacher time, parental time …) (WP7). Then, a health economic model will be developed to calculate the long term impact of the programme in terms of costs and health effects. This will result in a cost-effectiveness analysis expressed in cost per QALY or cost per avoided DALY.

Task 9.1 (month 24-25; 36-42)
Cost assessment
Both the preparation and implementation of the intervention requires the use of resources (staff time, material, etc). Two types of questionnaires will be developed for this purpose:
9.1.1. A questionnaire related to the preparation of the programme will be developed, whereby the project leaders and their staff will be the respondents (see WP 6).
9.1.2. A questionnaire will be developed to be integrated in the process evaluation questionnaires (see WP 7). This may include teacher time, parent time, etc.
9.1.3. The health economic researchers will collect data regarding the unit costs for each type of resource in the involved countries (cost of scientific staff time, cost of teacher time, printing material, etc)
9.1.4. The observed resource use is then afterwards multiplied with the unit cost of each type of resource. E.g. the number of hours of teacher time multiplied with the cost per hour. All unit costs are considered from a societal perspective.

Task 9.2 (month 36-48)
Cost- Effectiveness model
Based on the outcomes of the intervention (impact on weight, physical activity, food group intake, etc), as measured in WP7, predictions will be made related to the adult age of the participating children. The predictions are based on published relationships between childhood and adult parameters (e.g. the relation between childhood obesity and adult obesity; the relation between childhood fruit consumption and adulthood fruit consumption) [127,133-137]. These are then in turn related to avoided cardiovascular disease and avoided cancers (colorectal, breast).
The model will be a Markov model, consisting of different health or disease states and predicting the evolution of a hypothetical cohort of children undergoing the programme vs. a control cohort. The model consists of the following states: healthy, heart disease, cerebrovascular disease, diabetes, breast cancer, colorectal cancer, and death (see also [127,138]). Throughout the model, each member of the cohort can undergo transitions between states (e.g. from healthy to heart disease to cerebrovascular disease to death). The time horizon of the model is at least 50 years in order to capture all relevant future costs and outcomes. The perspective of analysis is societal, meaning that the impact of healthier life is not only assessed from a health care payer perspective but also from a societal perspective, i.e. including the economic impact of work productivity. Data inputs

from the model will be obtained partly from task 9.1., partly from validated published models, and published epidemiological and cost of disease data.

Person-Months per Participant

Participant number [10]	Participant short name [11]	Person-months per participant
1	HUA	1.00
2	LMU-Muenchen	1.00
3	UGent	12.00
5	UniZar	1.00
9	CMHI	0.50
10	MUV	1.00
13	AOK-Verlag	1.00
	Total	17.50

List of deliverables

Deliverable Number [61]	Deliverable Title	Lead beneficiary number	Estimated indicative person-months	Nature [62]	Dissemination level [63]	Delivery date [64]
D9.1	Interim report with the cost of the intervention	3	3.50	R	PU	43
D9.2	Projection model in MS Excel	3	4.00	O	PU	46
D9.3	A full report containing the health economic model description, data inputs and results	3	6.00	R	PU	48
D9.4	A brief report presenting key findings regarding long term costs, effects and cost-effectiveness	3	4.00	R	PU	48
		Total	17.50			

Description of deliverables

D9.1) Interim report with the cost of the intervention: [month 43]

D9.2) Projection model in MS Excel: [month 46]

D9.3) A full report containing the health economic model description, data inputs and results: [month 48]

D9.4) A brief report presenting key findings regarding long term costs, effects and cost-effectiveness: Full title: A brief report presenting key findings regarding long term costs, effects and cost-effectiveness of the programme to inform policy makers [month 48]

Schedule of relevant Milestones

Milestone number [59]	Milestone name	Lead beneficiary number	Delivery date from Annex I [60]	Comments
MS23	Full report on health economic model description, data inputs and results	3	48	

WT3:
Work package description

Project Number [1]	245200	Project Acronym [2]	ToyBox

One form per Work Package

Work package number [53]	WP10	Type of activity [54]	OTHER
Work package title	Dissemination		
Start month	6		
End month	50		
Lead beneficiary number [55]	11		

Objectives

10.1. Listing of a network of all research efforts at the European level on obesity and overweight prevention, nutrition and physical activity, and health inequalities in these issues across Europe, including information on the expertise and projects that are currently underway or being developed in these areas.
10.2. Dissemination of the publicly available deliverables and results from the project to the main stake holders: schoolchildren, parents, school staff, policy makers, health professionals and scientists
10.3. Inform and generate interaction between relevant stakeholders and public policy-makers working on the field of obesity, physical activity nutrition and health inequalities.
10.4. Serve as review board for research findings generated and serve as an advisory and consultancy point for the European Commission in relation to evidence-based policies.

Description of work and role of partners

Effective and successful interventions at school setting can only take place when there is sufficient public and stakeholder understanding and support, along with the necessary backing from policy-makers and politicians. WP10 takes the work of the ToyBox project and disseminates this to relevant stakeholders in order to directly inform policy and practice regarding obesity prevention efforts in young children. Because of the direct applicability of the project's results dissemination to the scientific community and to policy makers and health promotion professionals is equally important. To give this task the necessary attention, the ToyBox project has dedicated a specific WP to dissemination.
This WP will use all information available from WP 2-9, and especially information from WP5 (on contextual factors and existing infrastructures in schools) and WP 9 (on cost-effectiveness of the programme), in order to promote the ToyBox intervention and will further promote timely dissemination of the results of the Work Packages during the course of the project.

Task 10.1 (month 6-50) (IASO)
Prepare and maintain a list of programmes on obesity research, with a special focus on child obesity
To make a list and maintain and develop the IASO database of current and up-coming projects and programmes on obesity research, with a special emphasis on child obesity, and to create a network of groups (a network of networks) engaged in this research at European level. This builds on the current work undertaken in the EU-funded HOPE and EURO-PREVOB projects, and the IASO and EASO member associations.

Task 10.2 (month 6-50)
Promote dissemination of all public deliverables of ToyBox during the course of the project
10.2.1. To promote the ToyBox project and results in major international conferences and congresses;
10.2.2. To co-ordinate a series of press releases timed to coincide with ToyBox presentations at conferences and publication of ToyBox reports and scientific papers.
10.2.3. To produce Toy Box electronic newsletters (2-3 per year) .
10.2.4. To organise network–of-networks meetings aimed at reviewing the work in progress and acting as a vehicle for disseminating evidence-based policy recommendations. These will be scheduled between month 24 and month 36 of the project.

Task 10.2 will be co-ordinated by Partner 11 (IASO), but all WP participants will be involved.

Task 10.3 (month 36-50) (IASO, HiOA, UGent, NIGZ, HUA)

Inform relevant stakeholders and public policy-makers
10.3.1. To construct cross-cutting matrices of scientific knowledge in relation to specific policies and interventions proven to be effective for tackling overweight and obesity, especially among children.
10.3.2. Prepare popular summaries of the results of the ToyBox WPs for dissemination to public media;
10.3.3. Present findings of the ToyBox project to the DG Sanco Platform on Diet, Physical Activity and Health and other interested EC bodies;
10.3.4. Publish results of the ToyBox project in the separate sections on the project's website, including scientific summaries for professionals and more popular summaries on the public section of the website in all relevant languages.
10.3.5. To provide syntheses of the evidence generated in this project and relevant to policy-makers at the European, national and local level.
10.3.6. To inform young researchers and field workers active in the field of health promotion and policy making through the existing Young Public Health Nutritionists Network. (Members of UGent and HiOAare involved in this initiative and focus specifically on young and less experienced people)
10.3.7. To inform Educational Councils and Teachers organizations through the existing Schools for Health in Europe (SHE) Network. NIGZ is the coordinator of this Network.
10.3.8. To link with the recently formed World Public Health Nutritionist Association, which will be launched in 2009 at the IUNS conference in Bangkok, to also provide a global link.
10.3.9. To link with existing training programme networks for health care staff, for public health nutritionists and public health workers in Europe.
10.3.10. To link with the Developmental Origins research groups in order to provide a life course perspective and bring this into schools (early information to prospective mothers etc)

Person-Months per Participant

Participant number [10]	Participant short name [11]	Person-months per participant
1	HUA	4.00
3	UGent	4.00
6	HiOA	5.00
11	IASO	12.00
12	NIGZ	4.00
	Total	29.00

List of deliverables

Deliverable Number [61]	Deliverable Title	Lead beneficiary number	Estimated indicative person-months	Nature [62]	Dissemination level [63]	Delivery date [64]
D10.1	A listing of the Network of Networks	11	2.00	O	PU	6
D10.2	One Network of networks meetings	11	12.00	O	PU	50
D10.3	Matrices of key scientific findings against specific policies and interventions	11	3.00	O	PU	50
D10.4	A list with at least twenty (20) items that appeared in independent public media	11	2.00	O	RE	50
D10.5	A list with at least six (6) scientific papers submitted in a peer reviewed journals or conference	11	2.00	O	CO	50

List of deliverables

Delive-rable Number [61]	Deliverable Title	Lead benefi-ciary number	Estimated indicative person-months	Nature [62]	Dissemi-nation level [63]	Delivery date [64]
D10.6	Policy recommendation fact sheets for EU, national and local policy-makers	11	8.00	O	PU	50
		Total	29.00			

Description of deliverables

D10.1) A listing of the Network of Networks: [month 6]

D10.2) One Network of networks meetings: Network meetings will be organized between month 24 and month 36 [month 50]

D10.3) Matrices of key scientific findings against specific policies and interventions: Full title: Matrices of key scientific findings against specific policies and interventions according to their contexts (e.g., education, legislation) [month 50]

D10.4) A list with at least twenty (20) items that appeared in independent public media: [month 50]

D10.5) A list with at least six (6) scientific papers submitted in a peer reviewed journals or conference: Full title: A list with at least six (6) scientific papers submitted in a peer reviewed journals or conference presentations [month 50]

D10.6) Policy recommendation fact sheets for EU, national and local policy-makers: [month 50]

Schedule of relevant Milestones

Milestone number [59]	Milestone name	Lead benefi-ciary number	Delivery date from Annex I [60]	Comments
MS6	Setting up of network	11	6	
MS12	Web-portal network of networks Communication structure	11	10	
MS17	Meeting for dissemination of the results	11	50	
MS18	Final matrices of scientific findings against policies and interventions	11	50	months 24-36
MS21	Synthesis, scenarios and interaction with DG SANCO, EU Platform for Action and successor, policy mak	11	50	months 36-50

Project Number [1]	245200	Project Acronym [2]	ToyBox

List and Schedule of Milestones

Milestone number [59]	Milestone name	WP number [53]	Lead beneficiary number	Delivery date from Annex I [60]	Comments
MS1	Kick-off Steering Committee meeting	WP1	1	2	
MS2	Opening of the web-portal	WP1	1	6	
MS3	Completion of systematic literature review on behavioural models	WP4	7	6	
MS4	Completion of narrative literature review on educational strategies	WP4	7	6	
MS5	Report on existing policies and legislations at pre-primary education settings	WP5	12	6	
MS6	Setting up of network	WP10	11	6	
MS7	Completion of systematic literature review on identification of energy balance-related behaviours	WP2	4	8	
MS8	Completion of secondary data analyses on importance of energy balance behaviours	WP2	4	8	
MS9	Report on health promotion activities and legislations at pre-primary education settings	WP5	12	8	
MS10	Report on contextual factors at pre-primary education settings	WP5	12	8	
MS11	Report on good examples of Policies and health promotion activities	WP5	12	9	

Milestone number [59]	Milestone name	WP number [53]	Lead beneficiary number	Delivery date from Annex I [60]	Comments
	at pre-primary education setting				
MS12	Web-portal network of networks Communication structure	WP10	11	10	
MS14	Completion of focus group interviews and analyses	WP3	3	12	
MS15	Completion of survey instruments for evaluation study	WP8	5	26	
MS16	Intervention materials ready for the intervention study and delivered to participating countries	WP6	2	27	
MS17	Meeting for dissemination of the results	WP10	11	50	
MS18	Final matrices of scientific findings against policies and interventions	WP10	11	50	months 24-36
MS20	Completion of evaluation of the intervention	WP7	1	46	
MS21	Synthesis, scenarios and interaction with DG SANCO, EU Platform for Action and successor, policy mak	WP10	11	50	months 36-50
MS23	Full report on health economic model description, data inputs and results	WP9	3	48	

WT5:
Tentative schedule of Project Reviews

Project Number [1]	245200	Project Acronym [2]	ToyBox

Tentative schedule of Project Reviews

Review number [65]	Tentative timing	Planned venue of review	Comments, if any
RV 1	24	to be discussed	

WT6:
Project Effort by Beneficiary and Work Package

Project Number [1]	245200	Project Acronym [2]	ToyBox

Indicative efforts (man-months) per Beneficiary per Work Package

Beneficiary number and short-name	WP 1	WP 2	WP 3	WP 4	WP 5	WP 6	WP 7	WP 8	WP 9	WP 10	Total per Beneficiary
1 - HUA	26.00	1.00	3.00	1.00	1.00	7.00	36.00	1.00	1.00	4.00	81.00
2 - LMU-Muenchen	0.00	1.00	3.00	1.00	1.00	32.00	22.00	1.00	1.00	0.00	62.00
3 - UGent	0.00	4.00	18.00	2.00	9.00	9.00	22.00	2.00	12.00	4.00	82.00
4 - VUA	0.00	18.00	1.00	0.00	0.00	2.00	0.00	1.00	0.00	0.00	22.00
5 - UniZar	0.00	1.00	3.00	1.00	1.00	7.00	22.00	18.00	1.00	0.00	54.00
6 - HiOA	0.00	0.00	0.00	0.00	10.00	0.00	0.00	0.00	0.00	5.00	15.00
7 - UDUR	0.00	0.00	0.00	10.00	0.00	2.00	0.00	0.00	0.00	0.00	12.00
8 - IFP	0.00	0.00	0.00	3.00	0.00	12.00	0.00	0.00	0.00	0.00	15.00
9 - CMHI	0.00	1.00	3.00	1.00	1.00	7.00	20.70	2.80	0.50	0.00	37.00
10 - MUV	0.00	1.00	3.00	1.00	1.00	7.00	22.00	1.00	1.00	0.00	37.00
11 - IASO	0.00	0.00	0.00	0.00	0.00	0.00	0.00	0.00	0.00	12.00	12.00
12 - NIGZ	0.00	0.00	0.00	0.00	18.00	0.00	0.00	0.00	0.00	4.00	22.00
13 - AOK-Verlag	0.00	0.00	0.00	0.00	0.00	19.00	0.00	0.00	1.00	0.00	20.00
14 - RoU	0.00	0.00	0.00	2.50	0.00	2.00	0.00	0.00	0.00	0.00	4.50
15 - ULU	0.00	0.00	0.00	2.50	0.00	0.00	0.00	0.00	0.00	0.00	2.50
16 - VUmc	0.00	0.00	0.00	0.00	0.00	0.00	0.00	0.00	0.00	0.00	0.00
17 - CBO	0.00	0.00	0.00	0.00	0.00	0.00	0.00	0.00	0.00	0.00	0.00
Total	26.00	27.00	34.00	25.00	42.00	106.00	144.70	26.80	17.50	29.00	478.00

WT7:
Project Effort by Activity type per Beneficiary

Project Number [1]	245200	Project Acronym [2]	ToyBox

Indicative efforts per Activity Type per Beneficiary

Activity type	Part. 1 HUA	Part. 2 LMU-Mue	Part. 3 UGent	Part. 4 VUA	Part. 5 UniZar	Part. 6 HiOA	Part. 7 UDUR	Part. 8 IFP	Part. 9 CMHI	Part. 10 MUV	Part. 11 IASO	Part. 12 NIGZ	Part. 13 AOK-Ver	Part. 14 RoU
1. RTD/Innovation activities														
WP 2	1.00	1.00	4.00	18.00	1.00	0.00	0.00	0.00	1.00	1.00	0.00	0.00	0.00	0.00
WP 3	3.00	3.00	18.00	1.00	3.00	0.00	0.00	0.00	3.00	3.00	0.00	0.00	0.00	0.00
WP 4	1.00	1.00	2.00	0.00	1.00	0.00	10.00	3.00	1.00	1.00	0.00	0.00	0.00	2.50
WP 5	1.00	1.00	9.00	0.00	1.00	10.00	0.00	0.00	1.00	1.00	0.00	18.00	0.00	0.00
WP 6	7.00	32.00	9.00	2.00	7.00	0.00	2.00	12.00	7.00	7.00	0.00	0.00	19.00	2.00
WP 7	36.00	22.00	22.00	0.00	22.00	0.00	0.00	0.00	20.70	22.00	0.00	0.00	0.00	0.00
WP 8	1.00	1.00	2.00	1.00	18.00	0.00	0.00	0.00	2.80	1.00	0.00	0.00	0.00	0.00
WP 9	1.00	1.00	12.00	0.00	1.00	0.00	0.00	0.00	0.50	1.00	0.00	0.00	1.00	0.00
Total Research	51.00	62.00	78.00	22.00	54.00	10.00	12.00	15.00	37.00	37.00	0.00	18.00	20.00	4.50
2. Demonstration activities														
Total Demo	0.00	0.00	0.00	0.00	0.00	0.00	0.00	0.00	0.00	0.00	0.00	0.00	0.00	0.00
3. Consortium Management activities														
WP 1	26.00	0.00	0.00	0.00	0.00	0.00	0.00	0.00	0.00	0.00	0.00	0.00	0.00	0.00
Total Management	26.00	0.00	0.00	0.00	0.00	0.00	0.00	0.00	0.00	0.00	0.00	0.00	0.00	0.00
4. Other activities														
WP 10	4.00	0.00	4.00	0.00	0.00	5.00	0.00	0.00	0.00	0.00	12.00	4.00	0.00	0.00
Total other	4.00	0.00	4.00	0.00	0.00	5.00	0.00	0.00	0.00	0.00	12.00	4.00	0.00	0.00

WT7:
Project Effort by Activity type per Beneficiary

Total	81.00	62.00	82.00	22.00	54.00	15.00	12.00	15.00	37.00	37.00	12.00	22.00	20.00	4.50

WT7:
Project Effort by Activity type per Beneficiary

Activity type	Part. 15<br>ULU	Part. 16<br>VUmc	Part. 17<br>CBO	Total
1. RTD/Innovation activities				
WP 2	0.00	0.00	0.00	27.00
WP 3	0.00	0.00	0.00	34.00
WP 4	2.50	0.00	0.00	25.00
WP 5	0.00	0.00	0.00	42.00
WP 6	0.00	0.00	0.00	106.00
WP 7	0.00	0.00	0.00	144.70
WP 8	0.00	0.00	0.00	26.80
WP 9	0.00	0.00	0.00	17.50
Total Research	2.50	0.00	0.00	423.00
2. Demonstration activities				
Total Demo	0.00	0.00	0.00	0.00
3. Consortium Management activities				
WP 1	0.00	0.00	0.00	26.00
Total Management	0.00	0.00	0.00	26.00
4. Other activities				
WP 10	0.00	0.00	0.00	29.00
Total other	0.00	0.00	0.00	29.00
Total	2.50	0.00	0.00	478.00

WT8:
Project Effort and costs

Project Number [1]	245200	Project Acronym [2]	ToyBox

Project efforts and costs

Beneficiary number	Beneficiary short name	Estimated eligible costs (whole duration of the project)						Requested EU contribution (€)
		Effort (PM)	Personnel costs (€)	Subcontracting (€)	Other Direct costs (€)	Indirect costs OR lump sum, flat-rate or scale-of-unit (€)	Total costs	
1	HUA	81.00	364,500.00	25,000.00	61,500.00	255,600.00	706,600.00	589,000.00
2	LMU-Muench	62.00	310,000.00	0.00	11,000.00	192,600.00	513,600.00	385,200.00
3	UGent	82.00	410,000.00	0.00	16,500.00	255,900.00	682,400.00	511,800.00
4	VUA	22.00	0.00	0.00	0.00	0.00	0.00	0.00
5	UniZar	54.00	238,500.00	0.00	10,000.00	149,100.00	397,600.00	298,200.00
6	HiOA	15.00	82,500.00	0.00	5,000.00	52,500.00	140,000.00	105,000.00
7	UDUR	12.00	66,000.00	0.00	9,500.00	45,300.00	120,800.00	90,600.00
8	IFP	15.00	75,000.00	0.00	5,500.00	48,300.00	128,800.00	96,600.00
9	CMHI	37.00	106,600.00	3,700.00	15,000.00	72,960.00	198,260.00	148,695.00
10	MUV	37.00	111,000.00	0.00	13,000.00	74,400.00	198,400.00	148,800.00
11	IASO	12.00	66,000.00	0.00	50,000.00	69,600.00	185,600.00	139,200.00
12	NIGZ	22.00	0.00	0.00	0.00	0.00	0.00	0.00
13	AOK-Verlag	20.00	100,000.00	51,280.00	7,000.00	21,400.00	179,680.00	134,760.00
14	RoU	4.50	24,750.00	0.00	4,500.00	17,550.00	46,800.00	35,100.00
15	ULU	2.50	13,750.00	0.00	2,000.00	9,450.00	25,200.00	18,900.00
16	VUmc	0.00	115,000.00	0.00	7,500.00	73,500.00	196,000.00	147,000.00
17	CBO	0.00	110,000.00	0.00	7,000.00	70,200.00	187,200.00	140,400.00
Total		478.00	2,193,600.00	79,980.00	225,000.00	1,408,360.00	3,906,940.00	2,989,255.00

1. Project number

The project number has been assigned by the Commission as the unique identifier for your project. It cannot be changed. The project number **should appear on each page of the grant agreement preparation documents (part A and part B)** to prevent errors during its handling.

2. Project acronym

Use the project acronym as given in the submitted proposal. It cannot be changed unless agreed so during the negotiations. The same acronym **should appear on each page of the grant agreement preparation documents (part A and part B)** to prevent errors during its handling.

53. Work Package number

Work package number: WP1, WP2, WP3, ..., WPn

54. Type of activity

For all FP7 projects each work package must relate to one (and only one) of the following possible types of activity (only if applicable for the chosen funding scheme – must correspond to the GPF Form Ax.v):

• **RTD/INNO =** Research and technological development including scientific coordination - applicable for Collaborative Projects and Networks of Excellence

• **DEM =** Demonstration - applicable for collaborative projects and Research for the Benefit of Specific Groups

• **MGT =** Management of the consortium - applicable for all funding schemes

• **OTHER =** Other specific activities, applicable for all funding schemes

• **COORD =** Coordination activities – applicable only for CAs

• **SUPP =** Support activities – applicable only for SAs

55. Lead beneficiary number

Number of the beneficiary leading the work in this work package.

56. Person-months per work package

The total number of person-months allocated to each work package.

57. Start month

Relative start date for the work in the specific work packages, month 1 marking the start date of the project, and all other start dates being relative to this start date.

58. End month

Relative end date, month 1 marking the start date of the project, and all end dates being relative to this start date.

59. Milestone number

Milestone number:MS1, MS2, …, MSn

60. Delivery date for Milestone

Month in which the milestone will be achieved. Month 1 marking the start date of the project, and all delivery dates being relative to this start date.

61. Deliverable number

Deliverable numbers in order of delivery dates: D1 – Dn

62. Nature

Please indicate the nature of the deliverable using one of the following codes

R = Report, **P** = Prototype, **D** = Demonstrator, **O** = Other

63. Dissemination level

Please indicate the dissemination level using one of the following codes:

• **PU =** Public

• **PP =** Restricted to other programme participants (including the Commission Services)

• **RE =** Restricted to a group specified by the consortium (including the Commission Services)

• **CO =** Confidential, only for members of the consortium (including the Commission Services)

• **Restreint UE =** Classified with the classification level "Restreint UE" according to Commission Decision 2001/844 and amendments

• **Confidentiel UE =** Classified with the mention of the classification level "Confidentiel UE" according to Commission Decision 2001/844 and amendments

• **Secret UE =** Classified with the mention of the classification level "Secret UE" according to Commission Decision 2001/844 and amendments

64. Delivery date for Deliverable

Month in which the deliverables will be available. Month 1 marking the start date of the project, and all delivery dates being relative to this start date

65. Review number

Review number: RV1, RV2, ..., RVn

66. Tentative timing of reviews

Month after which the review will take place. Month 1 marking the start date of the project, and all delivery dates being relative to this start date.

67. Person-months per Deliverable

The total number of person-month allocated to each deliverable.

SEVENTH FRAMEWORK PROGRAMME
THEME 2
FOOD, AGRICULTURE AND FISHERIES, AND BIOTECHNOLOGY

Grant agreement for: ***Collaborative project***
Small or medium-scale focused research project

Annex I - "Description of Work"

Project acronym: *ToyBox*
Project full title: *Multifactorial evidence based approach using behavioural models in understanding and promoting fun, healthy food, play and policy for the prevention of obesity in early childhood*
Grant agreement no.: *245200*

Date of preparation of Annex I (latest version): 08/12/2011
Date of approval of Annex I by Commission:

List of Beneficiaries					
**Beneficiary Number**	**Beneficiary name**	**Beneficiary short name**	**Country**	**Date enter project**	**Date exit project**
1 (Coord.)	Harokopio University	HUA	Greece	1	50
2	LUDWIG-MAXIMILIANS UNIVERSITAET MUENCHEN	LMU	Germany	1	50
3	UNIVERSITEIT GENT	UGent	Belgium	1	50
4	VERENIGING VOOR CHRISTELIJK HOGER ONDERWIJS WETENSCHAPPELIJK ONDERZOEK EN PATIENTENZORG	VUmc	Netherlands	1	50
5	UNIVERSIDAD DE ZARAGOZA	UniZar	Spain	1	50
6	Høgskolen i Oslo og Akershus	HiOA	Norway	1	50
7	UNIVERSITY OF DURHAM	UDUR	UK	1	50
8	Staatsinstitut fur Fruhpadagogik	IFP	Germany	1	50
9	INSTITUT POMNIK CENTRUM ZDROWIA DZIECKA	CMHI	Poland	1	50
10	Medical University of Varna	MUV	Bulgaria	1	50
11	THE INTERNATIONAL ASSOCIATION FOR THE STUDY OF OBESITY	IASO	UK	1	50
12	NATIONAAL INSTITUUT VOOR GEZONDHEIDSBEVORDERING EN ZIEKTEPREVENTIE	NIGZ	Netherlands	1	50
13	AOK- Verlag GmbH	AOK-Verlag	Germany	1	50
14	Roehampton University	RoU	UK	1	50
15	UNIVERSITE DU LUXEMBOURG	ULU	Luxembourg	1	50

Contents Page

PART A

A1. Budget breakdown and project summary.....5
A.1 Overall budget breakdown for the project.....5
A.2 Project summary.....5
A.3 List of beneficiaries.....6

PART B

B1. Concept and objectives, progress beyond state-of-the-art, S/T methodology and work plan.....7

B.1.1 Concept and project objective(s).....7
B.1.2 Progress beyond the state of the art.....14
B.1.3 S/T methodology and associated work plan.....23
B.1.3.1 Overall strategy and general description.....23
B.1.3.2 Timing of work packages and their components.....26
B.1.3.3 Work package list /overview.....29
B.1.3.4 Deliverables list.....30
B.1.3.5 Work package descriptions.....33
B.1.3.6 Efforts for the full duration of the project.....60
B.1.3.7 List of milestones and planning of reviews.....62

B2. Implementation.....64

B.2.1 Management structure and procedures.....64
B.2.2 Beneficiaries.....70
B.2.3 Consortium as a whole.....86
Sub-contracting.....89
B.2.4 Resources to be committed.....90

B3.Impact.....92

B.3.1 Strategic impact.....92
B.3.2 Plan for the use and dissemination of foreground.....98

B4. Ethical issues.....101

B5. Consideration of gender aspects.....109

List of abbreviations/ Glossary

BMI: Body Mass Index (in kg/m^2)

DALYs: Disability adjusted life years; for more information, please see page 23.

EBRB: Energy Balanced Related Behaviours

IM: Intervention Mapping; for more information, please see pages 8-11.

P-P Model: Precede- Proceed Model; for more information, please see pages 8-11.

QALYs: Quality-adjusted life years; for more information, please see page 23.

SES: Socioeconomic status

WHO: World Health Organization

PART A Budget breakdown and project summary

A.1 Budget Breakdown

Participant number in this project [a]	Participant short name	Estimated eligible costs (whole duration of the project)					Total receipts	Requested EU contribution
		RTD / Innovation (A)	Demonstration (B)	Management (C)	Other (D)	Total A+B+C+D		
1	HUA	470,400.00	0.00	211,200.00	25,000.00	706,600.00	0.00	589,000.00
2	LMU-Muench	513,600.00	0.00	0.00	0.00	513,600.00	0.00	385,200.00
3	UGent	682,400.00	0.00	0.00	0.00	682,400.00	0.00	511,800.00
4	VUA	196,000.00	0.00	0.00	0.00	196,000.00	0.00	147,000.00
5	UniZar	397,600.00	0.00	0.00	0.00	397,600.00	0.00	298,200.00
6	HIAK	140,000.00	0.00	0.00	0.00	140,000.00	0.00	105,000.00
7	UDUR	120,800.00	0.00	0.00	0.00	120,800.00	0.00	90,600.00
8	IFP	128,800.00	0.00	0.00	0.00	128,800.00	0.00	96,600.00
9	CMHI	198,260.00	0.00	0.00	0.00	198,260.00	0.00	148,695.00
10	MUV	198,400.00	0.00	0.00	0.00	198,400.00	0.00	148,800.00
11	IASO	185,600.00	0.00	0.00	0.00	185,600.00	0.00	139,200.00
12	NIGZ	187,200.00	0.00	0.00	0.00	187,200.00	0.00	140,400.00
13	AOK-Verlag	179,680.00	0.00	0.00	0.00	179,680.00	0.00	134,760.00
14	RoU	46,800.00	0.00	0.00	0.00	46,800.00	0.00	35,100.00
15	ULU	25,200.00	0.00	0.00	0.00	25,200.00	0.00	18,900.00
TOTAL		3,670,740.00	0.00	211,200.00	25,000.00	3,906,940.00	0.00	2,989,255.00

A.2 Project Summary form

Project Number [1]	245200	Project Acronym [2]	ToyBox

One form per project

General information

Project title [3]	Multifactorial evidence based approach using behavioural models in understanding and promoting fun, healthy food, play and policy for the prevention of obesity in early childhood: ToyBox		
Starting date [4]	01/03/2010		
Duration in months [5]	42		
Call (part) identifier [6]	FP7-KBBE-2009-3		
Activity code(s) most relevant to your topic [7]	KBBE-2009-2-1-03: Behavioural models for prevention of obesity, with a particular focus on children		
Free keywords [8]		obesity prevention; young children; school-based intervention; evaluation; cost-effectiveness; policy makers; contextual factors; eating and physical activity behaviours; determinants of behaviours	

Abstract [9] (max. 2000 char.)

Objective The ToyBox proposal addresses KBBE-2009-2-1-03 - Behavioural models for prevention of obesity, with a particular focus on children. It will primary aim to influence children's behaviours and prevent obesity in early childhood. Strategy The proposal will identify key behaviours related to early childhood obesity and their determinants and evaluate behavioural models and educational strategies. Based on the obtained insights at a local level, a multidisciplinary team will develop and implement a school based family involved intervention programme that could be applied on a European scale. Process, impact, outcome and cost-effectiveness evaluation will be conducted to support decision making for European Public Health Policy. Methods The combined use of Precede-Proceed Model and Intervention Mapping will provide the framework for the development, implementation and evaluation of the ToyBox intervention. To achieve this, the project will be subdivided into 10 WPs. This carefully planned stepwise approach will include systematic reviews, secondary analyses of existing data sets, focus group research and school policies overview. Consortium The ToyBox project consortium spans the necessary multidisciplinary variety of experts such as public health experts, epidemiologists, nutritionists, physical activity experts, pedagogists, psychologists, behavioural scientists, nutritionists, paediatricians, early childhood psychologists, health economists, totalling 15 partners, from 10 countries. The consortium, consists of 11 universities, 1 research institute, 2 advocacy groups and an SME representing all regions of Europe. The consortium has ample experience in conducting and coordinating multi-centre international research as well as undertaking dissemination activities to all relevant stakeholders.

A.3 List of beneficiaries

Beneficiary Number	Beneficiary name	Beneficiary short name	Country	Date enter project	Date exit project
1 (Coord.)	Harokopio University	HUA	Greece	1	50
2	LUDWIG-MAXIMILIANS UNIVERSITAET MUENCHEN	LMU	Germany	1	50
3	UNIVERSITEIT GENT	UGent	Belgium	1	50
4	VERENIGING VOOR CHRISTELIJK HOGER ONDERWIJS WETENSCHAPPELIJK ONDERZOEK EN PATIENTENZORG	VUmc	Netherlands	1	50
5	UNIVERSIDAD DE ZARAGOZA	UniZar	Spain	1	50
6	Høgskolen i Oslo og Akershus	HiOA	Norway	1	50
7	UNIVERSITY OF DURHAM	UDUR	UK	1	50
8	Staatsinstitut fur Fruhpadagogik	IFP	Germany	1	50
9	INSTITUT POMNIK CENTRUM ZDROWIA DZIECKA	CMHI	Poland	1	50
10	Medical University of Varna	MUV	Bulgaria	1	50
11	THE INTERNATIONAL ASSOCIATION FOR THE STUDY OF OBESITY	IASO	UK	1	50
12	NATIONAAL INSTITUUT VOOR GEZONDHEIDSBEVORDERING EN ZIEKTEPREVENTIE	NIGZ	Netherlands	1	50
13	AOK- Verlag GmbH	AOK-Verlag	Germany	1	50
14	Roehampton University	RoU	UK	1	50
15	UNIVERSITE DU LUXEMBOURG	ULU	Luxembourg	1	50

PART B

B1. Concept and objectives, progress beyond state-of-the-art, S/T methodology and work plan

B 1.1 Concept and project objective(s)

The present project addresses KBBE-2009-2-1-03 - Behavioural models for prevention of obesity, with a particular focus on children. The project will use a multidisciplinary analysis to identify the **key behaviours** related to obesity in early childhood and conduct new **behavioural research** to explain why **young children eat the foods they eat and why they do (or do not) participate in physical activity** at a **local level.** ToyBox will review and evaluate **behavioural models** and **educational strategies** to identify best practices in changing children's behaviours. Based on the obtained insights, a multidisciplinary team will develop and implement a **new intervention programme,** that could be applied on a **European scale,** primarily aiming to **influence children's behaviours and prevent obesity,** evaluate its **impact**, **outcome** and **cost-effectiveness** and contribute to the activities of the **EU platform on Diet and Physical Activity and Health** and to support decision making for **European Public Health Policy.**

B 1.1.1 The obesity epidemic and obesity in childhood

Obesity has reached epidemic proportions in adult populations showing a steep increase in the past decades with the highest percentages being observed in US. Projected estimates –assuming that the trends remain the same with those of the last four decades- have shown that the prevalence of overweight and obesity will reach 100% of the adult population in US by 2050 whereas certain subgroups (e.g. black women) would reach that state by 2034 [1]. Europe has not remained unaffected by this pandemic whereas the limited trajectory studies have showed that one third of adult population in UK (almost 13 million individuals) will be obese by 2012 and 150 million adults will be obese in the WHO European Region by 2010, if recent trends in adult obesity continue [2-4].

Overweight and obesity can substantially increase the risk of morbidity (from hypertension, dyslipidaemia and sleep apnoea to coronary artery disease, type 2 diabetes and some forms of cancer) and mortality from all causes but is also associated with psychological burden through the discrimination or stigmatization that obese individuals may suffer [5,6]. Subsequently the obesity epidemic also has huge economic and social implications and consequences. Total health-care costs attributable to obesity/overweight would double every decade to $860.7- $956.9 billion by 2030, accounting for 16-18% of total US health-care costs [1]. In the European Union, obesity accounts for up to 7% of health care costs, and it has been associated with a decreased productivity [7]. Overall, obesity has a substantial economic impact in different European countries, with associated costs ranging from 0.09% to 0.61% of national gross domestic income which reflects a maximum of €10 billion–when translated into absolute costs [8,9]. The true costs are undoubtedly much greater as not all obesity-related conditions are included in the calculations.

Many recent reports have indicated that the prevalence of obesity in childhood and adolescence has been also increasing worldwide at an alarming rate [10-14]. Worldwide, 155 million children are currently overweight of which 30 to 45 million are obese; the corresponding European figures are 14 million and 3 million respectively. In percentages, the mean prevalence of child overweight and obesity in Europe is about 20% and 5% respectively [15]. Childhood obesity is linked with premature onset of puberty, insulin resistance, hypertension and dyslipidaemia in children [16] whereas the psychosocial implications of childhood obesity are also very important and may lead in low self-esteem and disturbed body image or even social seclusion [16,17].

B 1.1.2 The need for early prevention

The epidemic of obesity calls for appropriate measures and effective policies to be taken early in life whereas early prevention seems as an emerging and effective approach to decrease obesity and obesity-related chronic diseases later in adulthood [18-22]. Thus a more population based effort might be required, in contrast to interventions focusing only on high-risk (i.e. already obese) individuals. Overweight in early childhood[1] has been shown to increase the likelihood of being obese in later childhood [23,24] but also tracks to adulthood in one-third to one-half of cases [25], where it becomes associated with an increased prevalence of chronic diseases [26-28]. Reasons to emphasize obesity prevention in early childhood include adipocyte physiology, adiposity rebound and the limited potential for reversing metabolic changes associated with obesity in later life [29]. Early in life and mainly during infancy the ability of adipose tissue stromal cells to differentiate into triglyceride-filled adipocytes is facilitated [30]. Furthermore, dysregulation of appetite and satiety regulating hormones or hormonal peptides such as leptin, ghrelin etc are probably responsible for increased susceptibility to adiposity, particularly in a highly calorigenic and sedentary environment [31]. Finally several studies suggest that when adiposity rebound (i.e. the second rise in BMI curve that normally occurs between ages 5 and 7 years) occurs earlier in life there is an increased risk of obesity, glucose intolerance and diabetes in adulthood [17,32]. The early adiposity rebound recorded in most obese subjects and the striking difference in the mean age of occurrence between obese subjects (3 years of age) and non-obese subjects (6 years of age) suggest that factors have operated very early in life [33].

The dominant influence on food preferences in young children is the energy density of foods [34]. This means that without appropriate regulation of eating behaviour, children will 'feed forward' into obesity in the current energy-dense food environment. However, early food- or physical activity-related experiences may influence the acquisition of food repertoire and engagement in physical activity in later life [35-39]. The development and adoption of desired eating and physical activity habits may help prevent those physiological and hormonal dysregulations whereas it seems that their formation is easier early in life; as such early childhood seems to be the ideal age to intervene.

B 1.1.3 Factors influencing young children's behaviours

An essential step in health promotion planning is the identification of the determinants of engagement in target behaviours [40]. Till now most research on behavioural and environmental determinants of physical activity and diet related behaviours is executed in primary schoolchildren, adolescents and adults. However, part of this research might not be applicable to younger children as personal determinants (e.g. knowledge) predominantly rely on social-cognitive procedures suggesting that eating healthy or being physically active is the result of a rational decision-making process [41], which is not relevant for this young age group due to their developmental stage. Thus social-cognitive theories cannot be used for young children themselves, but they can be used to explain parents' and teachers' behaviours towards preschoolers. In this young age group especially socio-environmental and physical environmental factors should be included in a determinant analysis [42-44]. These should include measures of parental diet and child feeding practices [45-47], as well as aspects of the home environment such as meal structure [48] and television watching [49]. Other key determinants of young children's eating behaviour and weight gain are heritable personality features related to eating, such as food neophobia (avoidance of novel foods), enjoyment of food and satiety responsiveness [50,51]. These are important not just because they are associated with adiposity in young children, but also because they are likely to have a strong influence on parental feeding practices [45,48].

B 1.1.4 Behavioural models and educational strategies to prevent obesity in early childhood

Due to the above, any intervention aiming to prevent childhood obesity early in life should primarily aim to support the development of healthy habits, target parental behaviours and develop a

[1] The terms "early childhood" or "young children" refer to children before the age of 6 years.

supportive social (parental and teachers' attitudes, knowledge, beliefs and practices) and physical environment (home, school, neighbourhood).

Behavioural models provide the theoretical framework for the design and implementation of school based interventions whereas **educational strategies** are practical plans of action that parents and teachers can use to help children achieve the desired behaviours. Recent reviews on the use of such models for the prevention of obesity have indicated that some commonly used and promising models are the Social Cognitive Theory, the Socio-Ecological Model, the Theory of Planned Behaviour and the Social Marketing [52,53] while some educational strategies proven to be effective regard the number of exposures to food, methods of encouragement, or 'learning to like' procedures [51,54,55],

The school setting can serve as an ideal environment to host such models or strategies with the additional advantage of being cost-effective since -with a relatively low cost and no further need for investment on facilities and personnel- access to large populations of children and parents can be achieved. However this requires further organizational changes regarding school- based health promotion programmes, regulations and policies.

What seems to be missing is a socio-ecological framework providing a holistic multifactorial and cost-effective approach, within which the most effective aspects of behavioural models and strategies can be combined, but which will also consider existing policies and regulations and engage relevant stakeholders for the design, implementation and evaluation of the health promotion programme.

B 1.1.5 Approach

A conceptual model ideally serving the needs of the current call is the PRECEDE- PROCEED (P-P) Model[23] [58] (see Figure 1, p.10), an educational and ecological approach in health programme planning. The salient features of this model of programme planning and evaluation are found in the phases and procedures that follow a sequence of steps aligned with a generic logic model or systems model of causes and effects. The framework has two components. The first component is referred to as PRECEDE (Predisposing, Reinforcing and Enabling Constructs in Educational/Ecological Diagnosis and Evaluation) and comprises of several planned assessment phases. The information and knowledge obtained during the PRECEDE phases will guide the development of the intervention; thus the intervention scheme will not be an intuitive process but it will rather be the result of a systematic and dynamic procedure addressing in the most effective way the needs of the corresponding population and providing a tailor-made and potentially cost-effective approach. The second component (Phases 5-8) is marked by the strategic implementation of the intervention providing also the framework for the evaluation of process, impact and outcome of the intervention. This second component is named PROCEED (Policy, Regulatory and Organizational Constructs in Educational and Environmental Development).

Although the P-P Model provides a detailed and well-structured approach for the assessment (PRECEDE) and the implementation and evaluation (PROCEED) procedures, it lacks a stepwise practical guide for the development of the intervention. Such guide can lead scientists in extracting and blending together the results of the PRECEDE phases to come up with a detailed intervention plan with specific goals, practical approaches and guidance on *how* to develop and implement the intervention. In the current project, Intervention Mapping (IM) [59] will provide this stepwise guiding plan and will therefore be incorporated between Phases 4 and 5 of the P-P Model. This approach will be used in this project (as well as the WorkPackages constituting it) as the planning framework for the development, implementation and evaluation of an obesity prevention programme in young children aged 4-6 years old which could be transferred and applied at a European scale. It will consist of the following three steps:

---

[2] The complete Precede-Proceed Model was inspired by CDC's Planned Approach to Community Health (PATCH) [56]

[3] A survey of 253 Universities ranked Precede-Proceed Model highest among the 10 planning models on usefulness for research (86%), and usefulness for practice (90.8%) [57].

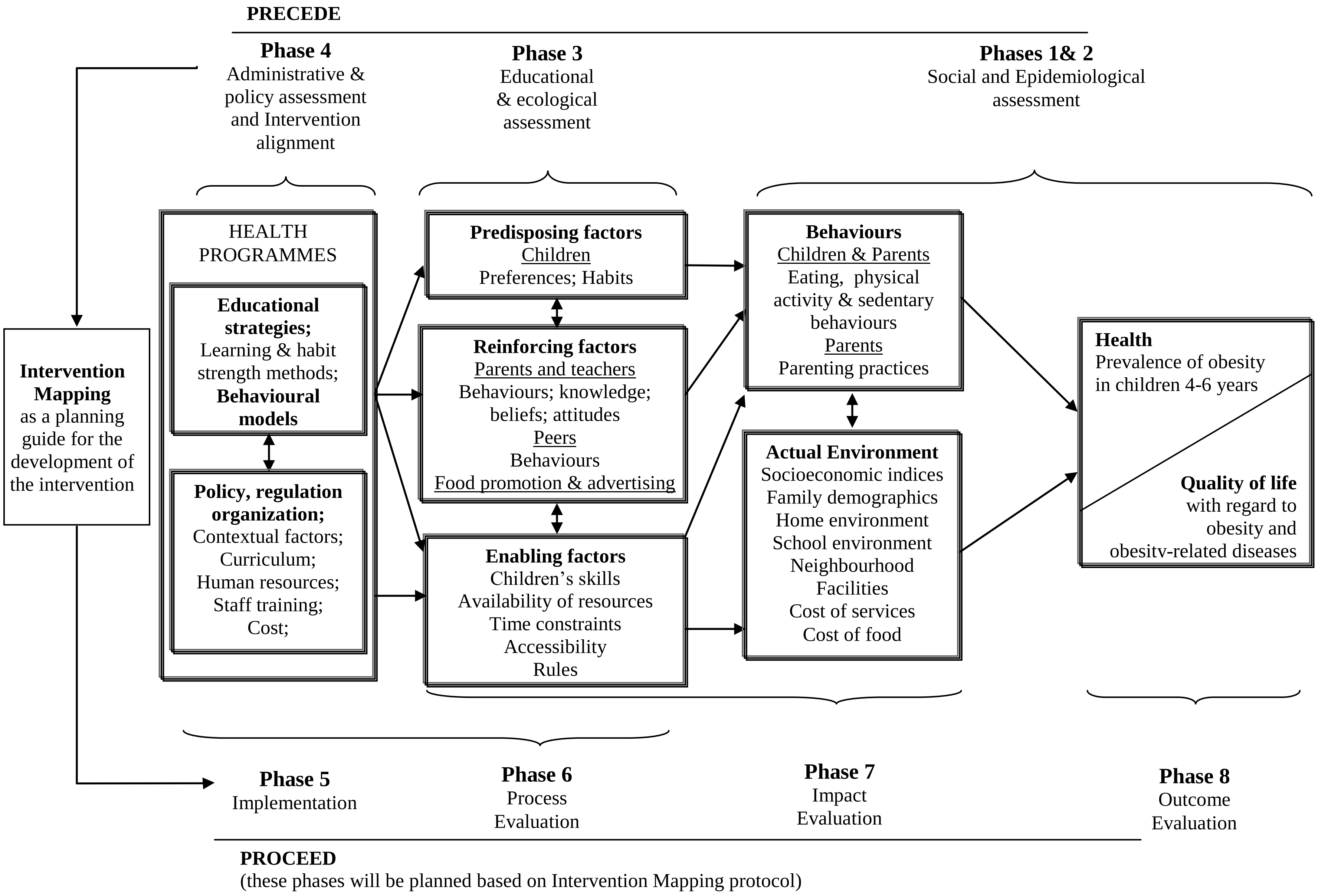

Figure 1: The conceptual model of the ToyBox project, Adapted by: Green and Kreuter [58]

Systematic and planned assessments

The Precede phases aim to unveil *who* has the problem and *why* they have it. In Phases 1 and 2, obesity prevalence in young childhood will be assessed and unique variations (country- and socioeconomic status (SES)- specific differences) will be detected with the use of situational analysis; this will be performed for both **behavioural** (for children aged 4-6 years old and their parents and parenting practices) and **environmental factors** (e.g. physical or socioeconomic). Phase 3 will assess the **causal factors** of behaviours and environment, namely the predisposing factors (children's preferences and habits), the reinforcing factors (parental and teachers' knowledge, beliefs, attitudes, and behaviours, food promotion and advertising) and the enabling factors (skills, resources or barriers that can help or hinder the desired behavioural changes as well as environmental changes), examining the involved stakeholders' views. Finally, having the scope of an intervention programme that can be applied at a European level, Phase 4 will examine the contextual factors and organizational structure (available personnel, curriculum, time, policies and legislations) in different European countries but also seek most appropriate behavioural models and educational strategies applicable in the school setting.

Evidence based development of the ToyBox intervention

The insights gained in the Precede Phases 1-3 will feed the first stages of IM (see Figure 2, p. 12) to establish SMART (Specific, Measurable, Achievable, Relevant, Time-framed) intervention objectives. For each of these objectives the most appropriate **theory and evidence-based methods** (deriving from effective behavioural models) **and educational strategies,** both identified in Precede Phase 4, **will be combined** in a comprehensive intervention programme. Finally, a plan for the implementation and evaluation will be schemed and will be followed in Phases 5-8 of the P-P Model, based on the findings of Precede Phase 4 on Policy, Regulation and Organization factors, to identify most appropriate entry points and alliances in the school setting.

Implementation and evaluation of the ToyBox intervention

Finally, the Proceed phases will follow the IM drafted plan to perform the implementation and the evaluation of the process, impact and outcome of the programme based on measurable objectives. In addition, cost-effectiveness will be explicitly considered as one of the primary outcomes. Cost-effectiveness is defined as the ratio between the (expected) net cost of an intervention and the (expected) health outcomes [60].

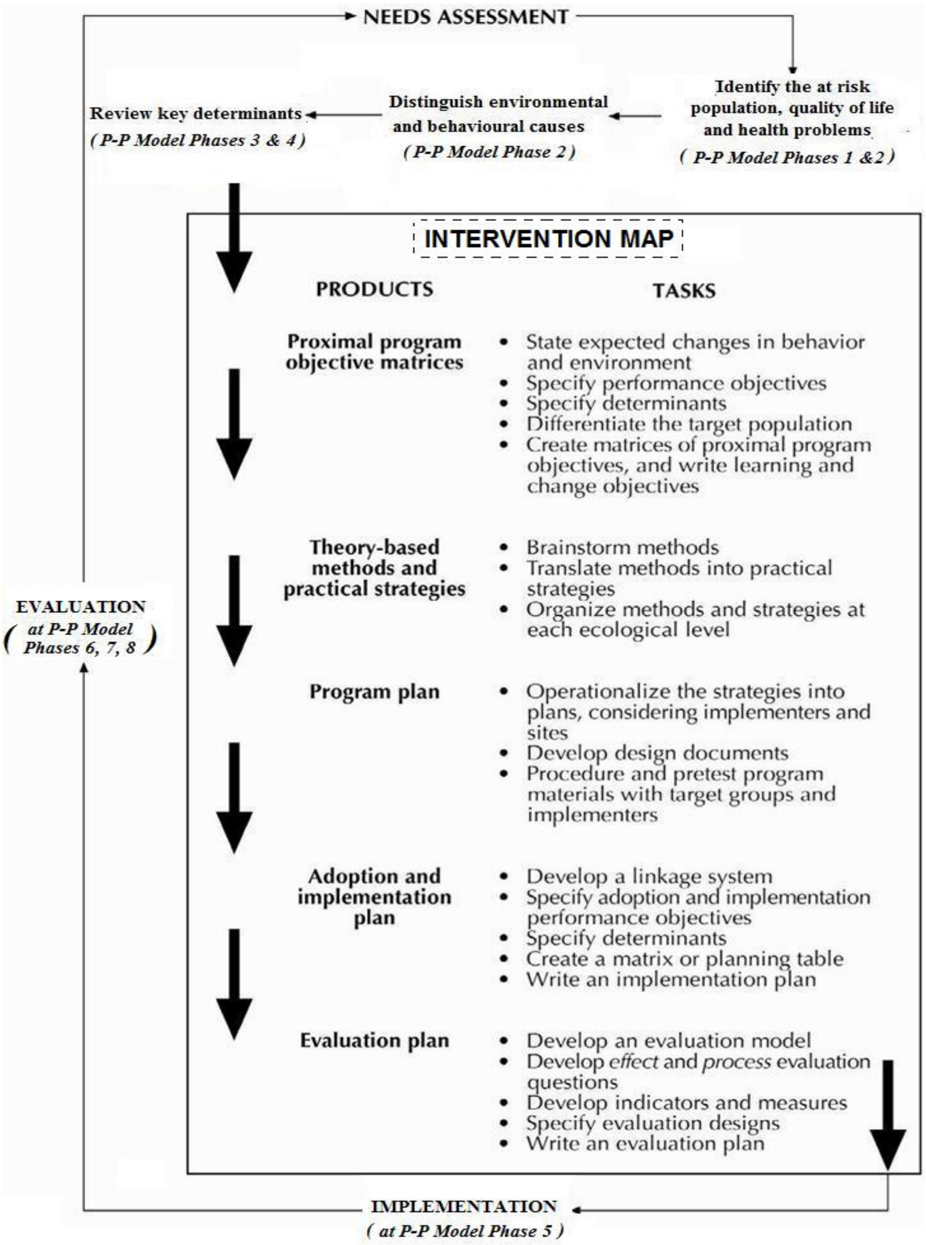

Figure 2: The five steps of the Intervention Mapping will be used for the development of the ToyBox intervention, Source: Bartholomew et al [59].

Thereby, ToyBox will take into account important insights in the field, and go beyond the state-of-the art, as will be extensively addressed below. In short, it will make use of the following important observations:

1) early childhood is a critical period for preventing obesity due to physiological and hormonal adaptations but also to the fact that energy balance related behaviours can more easily be formed and changed;
2) both social (parents, teachers, peers) and physical (home, school, neighbourhood) are important determinants of young children’s energy balance related behaviours;
3) the school setting is an important and potentially cost-effective environment to promote health behaviour among young children as far as any intervention scheme is developed and implemented in alliance with existing policies and regulations with the engagement of stakeholders and making use of available human resources and facilities.

This is to build and evaluate a cost-effective school- based, family involved intervention scheme aiming to prevent obesity in early childhood and which could potentially be applied on a European scale.

B 1.1.6 Objectives of the ToyBox project

1. Conduction of thorough secondary analyses for the identification of children's **lifestyle behaviours** related to obesity in early childhood;
2. **New behavioural research** to understand **at local level** the determinants of these behaviours;
3. Review and evaluation of existing **behavioural models** and **educational strategies** applied in young children aiming to influence energy balance related behaviours;
4. Assessment of the role of **school contextual framework**, legislation and stakeholders acting as mediators and moderators of the effectiveness of any intervention applied in the school setting;
5. Using obtained **insights** from the above mentioned analyses (points 1-3), development of a **multi-component school-based and family involved intervention** applicable on a European scale aiming to influence behaviours in early childhood;
6. Implementation of a **cluster randomized intervention** applying the multi-component programme, evaluate **process, impact and outcome** and estimate its **cost-effectiveness**;
7. Undertaking of **dissemination activities** to contribute to the Activities of the EU Platform on Diet, Physical Activity and Health and **support decision making for European Public Health Policy**.

To reach these objectives a multidisciplinary team of researchers from 9 countries supported by the International Association for the Study of Obesity and the Netherlands Institute for Health Promotion and Disease Prevention (NIGZ-WHO collaborating centre for School Health Promotion), which has been the coordinator for the *Schools for Health in Europe* programme has joined forces. This team has direct access to the most relevant data sets on energy balance related behaviours and ample experience in conducting behavioural research; members of this team have also been leading parts of earlier examples of successful health behaviour change intervention schemes both at a national and European level.

B 1.1.7 Relationship to the call

The present project addresses KBBE-2009-2-1-03 - Behavioural models for prevention of obesity, with a particular focus on children. The project will systematically:

1. Identify **children's eating and physical activity behaviours related to obesity in early childhood;**
2. Conduct new **behavioural research** to explain why **young children eat the foods they eat and why they do (or do not) participate in physical activity** at a **local level;**
3. Review and assess **behavioural models** and **educational strategies** to identify best practices in changing children's behaviours**.**

Integration of expertise **between different fields of science** will **combine** the above **insights** to:

1. Develop and implement a **new intervention programme,** that could be applied on a **European scale,** primarily aiming to **influence children's behaviours** and **prevent obesity**;
2. Evaluate the **impact**, **outcome** and **cost-effectiveness** of this intervention programme;
3. All the above will contribute to the activities of the **EU platform on Diet and Physical Activity and Health** and support decision making for **European Public Health Policy**.

B 1.2 Progress beyond the state of the art

B 1.2.1State-of-the-art

Energy balance related behaviours and their determinants in childhood

Obesity is a chronic outcome of energy imbalance, whereby a long-term excess of energy *intake* over *expenditure* leads to the storage of that excess energy as fat mass. However, both energy intake and energy expenditure are not in itself behaviours and it is less clear which specific health behaviours contribute to the risk of excess weight gain in different populations. This may vary across different groups defined by region, ethnicity, age, gender or sociodemographic characteristics [61].

So, a first important step in health promotion planning is to identify the most relevant energy balance related behaviours and specify these behaviours for risk groups in the population. Some potential target behaviours in this young age within the context of prevention of obesity are increase of fruit & vegetable intake, increase of water consumption, decrease of sugar sweetened beverages, decrease of the consumption of energy dense sugar and fat rich foods, increase of physical activity and decrease of sedentary activities such as TV/video/DVD watching or computer use [62-67]. It has been shown that children at the age of 3-5 years have already established some sedentary lifestyle patterns [68], that overweight preschoolers were less physically active [69], that TV viewing for more than two hours per day was associated with overweight [70], and that high TV viewing was associated with a higher energy intake [49]. For soft-drink consumption, however, results seem contradictory [71]. So far, no systematic review has been conducted to identify the most important sub-behaviours related to overweight or obesity among young children whereas most studies so far, have been conducted in the USA.

The next step, after identifying the specific energy balance related behaviours in early childhood, would be to explain why young children engage in these target behaviours [40]. Till now most research on behavioural and environmental determinants of physical activity and eating behaviours is executed in older primary school children, adolescents and adults. These studies on determinants were mainly based upon social-cognitive theories and models suggesting that eating healthy or being physically active is the result of a rational decision-making process [41]. A literature review on the potential determinants of behaviours of primary school children showed that personal (knowledge, self-efficacy, taste preferences), socio-environmental (parental modelling, peer influences, food promotion and advertising) and physical environmental factors (availability and accessibility) are all related to these behaviours [72-74].

However, when it comes to young children –due to limitations posing from their developmental stage- their behaviours are mostly influenced by family and physical environmental cues without involving conscious decision making [42-44]. It is thus deduced that, since behaviours of preschoolers are not likely to be reasoned and planned, socio-cognitive theories may be insufficient in explaining and changing their behaviours. Only recently, few studies in US focused and investigated the influencing factors of these behaviours in young children by examining parents' and teachers' beliefs or attitudes and assessing the actual school environment using the method of focus groups. Interviews with parents showed that parental perception on the adequacy of activity levels, time, safety, inclement weather and parental lack of knowledge were determining factors of child's engagement in physical activity. Interviews with teachers revealed that they recognized time, space but also equipment as barriers to providing activity in their classroom. Supportive environment was also shown to enhance physical activity [75-77].

Use of behavioural models and educational strategies to promote healthy weight and healthy energy balance related behaviours

The results of a recent Workshop on Childhood Obesity Prevention and Treatment [78] stated that theoretical and conceptual frameworks must be used when designing interventions for paediatric obesity prevention and treatment. **Behavioural models** and conceptual frameworks that have been used in childhood obesity prevention research include the Transtheoretical Model, Theory of Planned Behaviour, Health Belief Model, Social Cognitive Theory, and Socio-ecological Models [67,78]. However, since the developmental immaturity of young children attenuates the power of socio-cognitive theories in explaining their behaviours, it is important to select carefully the theoretical background and define the most appropriate methods and strategies to address young children's unstructured ways of behaving as well as their social and physical environment. For example, to achieve an increase in physical activity, social learning may be more appropriate for girls, whereas structural and environmental interventions enabling physical activity may be more effective for boys. This leads us to the conclusion that a combination of those could be more effective than the use of the one or the other alone [53]. Accordingly, promotion of physical activity is possible with the creation of attractive alternatives to physical play in the inner and outer area of the setting. In addition, what might work in one age group, might not work in others; parental involvement is less effective when children transit to secondary school and this becomes even more evident as they grow older. It is therefore important to use a multifactorial theoretical approach that also considers the impact of system, environment and organizational issues, as well as the need to consider and address individual and group behaviour change [72,79].

Although, behavioural models provide the theoretical framework for the design and implementation of school based interventions, **educational strategies** are practical plans of action that parents and teachers can use to help children achieve the desired behaviours. It should also be noted that combining behavioural models and educational strategies seems like the most prominent way of achieving the desired changes in behaviours. The infant's biological preferences for sweet and aversion for sour and bitter are later modified by socio-cultural learning processes influenced by parents, caretakers and peer groups [80]. Children's tendency to refuse new edibles (food neophobia) is mostly congenital [81], but can be reduced by a process of "learning to like", i.e. by repeated eating of the disliked food in a positive social context [54,55]. Indeed, experiments have shown that children will learn to dislike the flavour of a drink when its consumption is paired with viewing a role model showing negative facial responses to the drink, compared to neutral ones [82]. It is also possible that educating, in children, self-awareness of their own neophobic tendencies, as well as awareness of its lack in appropriate peer role models would encourage trying new foods. This would likely improve the healthiness of children's diets, because fruit, vegetables and good protein sources are particularly avoided by neophobic children [83]. Even so, this aspect of 'fussy eating' needs to be put in the context of evidence that it might be protective against risk of obesity [51], though this is contentious and the resultant diet limited to sweet, fatty and starchy staple foods might promote weight gain as well as poor nutrition [48]. On the other hand, neophobia-induced small meal sizes may enhance sensitivity to internal hunger cues and encourage more adaptive eating. Certainly, consideration of individual differences is likely to be a necessary component of successful interventions [84].

A dominant influence on food preferences in young children is the energy density of foods, even amongst fruit and vegetables, presumably resulting from learned liking due to energy-based reinforcement [34]. This means that without appropriate regulation of eating behaviour (internal or external), children will 'feed forward' into obesity in the current energy-dense food environment. Knowledge of this powerful influence on children's preferences may help caregivers use more effective strategies. For example, combining fruit or vegetables with sensory cues to richer energy density may encourage their consumption. Simply restricting "unhealthy" food and promoting "healthy" food is often counterproductive, i.e. children eat more of the forbidden foods as soon as they are available [85]. However, there is some evidence that an intermediate level of restriction may be effective [86]. Moreover, it is possible that restrictive rules deliberately applied to healthier snacks could actually encourage their consumption [87].

Thus, it is important to increase our knowledge on educational strategies and improve our skills in using them to be able to achieve the desired effects in children's behaviours. Parents and teachers should be familiar with most effective educational strategies that can be used in this age and combine their use appropriately according to children's characteristics. Any intervention (including behavioural models and educational strategies used) should be tailored by age, as well as gender, et cetera. Conclusively, given the complexity underlying young children's behaviours, the combination of effective behavioural models and educational strategies within a socio-ecological framework seems to be the most promising approach for the prevention of obesity [52].

Contextual factors and policy framework for school based interventions

The school setting is an ideal place to implement health promotion interventions, since they provide access to large groups of children and parents without requiring additional investment in terms of personnel, facilities et cetera. Recognition of the importance of multifaceted approaches to health promotion has led to the development of comprehensive school health programmes in the USA and to whole-school approaches to health promotion in Europe [88,89]. These later evolved to define health promoting schools. According to a recent review by the International Union for Health Promotion and Education (IUPHE) [90], a health promoting school has six essential components: 1) healthy school policies: it is clearly defined in documents or accepted practices that promote health and well-being; 2) the school's physical environment: it relates to the buildings, grounds, and equipment in and around the school, drinking water facilities, etc; 3) the school's social environment: it is the combination of the quality of the relationships among and between staff and students, parents and the wider community; 4) health skills and action competencies: it refers to both the formal and informal curriculum and associated activities, where students gain knowledge, understandings, skills and experiences; 5) community links: the connections between the school and local groups and individuals; 6) health services: local and regional school-based or school-linked health services, for child health care and promotion, through the provision of direct services to pupils. This actually means that emphasis is not placed exclusively on teaching and learning processes, but also on the whole school atmosphere, relationships, management structures and physical environment [91]. This holistic approach is central to the health promoting school approach [92]. Thus, identifying the social and environmental factors that influence children's behaviour and taking them into account when designing the intervention programmes could further improve their effectiveness [1,93,94].

However, what is currently lacking in the evidence base for childhood obesity prevention reflects a broader shortage of generalizable, effective, and sustainable interventions in many areas of prevention and health risk behaviours [95-97]. Most of the studies so far have not taken all above mentioned points under consideration when designing and implementing an intervention; possible shortages included insufficient data on specific energy balance related behaviours or their determinants and non use of behavioural models or conceptual frameworks.

In order to be able to transfer and expand a school-based research programme at a national or European level available facilities and human resources, skills and time availability, stakeholders' views and legislation should be taken into account; however such overview is missing from most research programmes. For example, in many cases, the intervention is delivered by staff other than the one existing in the school setting. However the skills and commitment of the person delivering the intervention is highly important in affecting the magnitude of the outcome when delivering school based health promotion programmes. In addition, teachers may often appear highly reluctant for taking extra work with new initiatives and programmes on health promotion [88]. Another factor hindering the transferability of such programmes is the conflict of interest of some stakeholders, such as school canteens or people responsible for health promotion activities at school. On the other hand, over-committed, under-supported teachers may also find it difficult to embrace new programmes [88]. Some of the barriers identified in school-based programmes are lack of time, lack of training, lack of education materials, lack of administrative support and lack of funds; if those are not resolved, adherence to the programme may be risked [98]. If these

parameters are not taken into consideration, then the efficacy, effectiveness and transferability of a school-based research programme at a national or European scale is questionable.

Previous interventions have not being designed neither to ensure a ‘cultural shift’ which is sustainable outside the research project nor to take a holistic view of the whole environment (human resources, facilities, contextual factors and stakeholders’ views and skills) and make attempts to facilitate synergies and provide recommendations for potential policy changes.

School-based intervention programmes to prevent obesity in childhood

The most commonly used public health strategies to combat childhood obesity are targeting infancy (by promoting breastfeeding), childhood or adolescence (by limiting television viewing, encouraging physical activity, increasing fruit and vegetable consumption, controlling portion size and limiting soft drink consumption) [62-67]. The majority of interventions have been conducted in the US and did not focus on either infancy or early childhood but rather aimed at primary school children or adolescents, using the school setting for their implementation. In addition, they comprised studies that focused solely on nutrition or on physical activity, and few combined strategies to address both behaviours [21,72,99]. Nonetheless, experts have recognised recently that a variety of energy balance-related behaviours should be jointly addressed to curb the obesity epidemic, and a carefully planned, evidence and theory-based multidisciplinary approach is necessary to accomplish this [100].

Moreover, a recent review by Sharma on school based interventions for the prevention of obesity [99] found that all interventions documenting parental involvement successfully influenced obesity indices (StEP TWO programme in the UK, the Cretan Health and Nutrition Education Programme, CATCH) [101-104]. In fact parental involvement appears as a key factor in any successful intervention strategy [61]. A recent review by Bluford et al [105] showed that interventions including high parental involvement were very successful in preventing weight gain among preschoolers. Parents are also very important in defining their children’s home environment as they provide the food (quantity & quality) and opportunities for physical activity but also by serving as role models regarding both dietary and physical activity habits. Dwyer et al [106] recently showed that positive parental modelling and preschool programmes facilitated preschool children’s physical activity. Moreover, studies have shown that changing also parental lifestyle habits has led to a significant increase of interventions’ success in children’s energy balance related behaviours [107,108].

To date most efforts to prevent obesity by promoting energy balance-related behaviours have not managed to provide the desired outcomes (reported either as BMI, percent fat content or skin-fold thickness), some studies found improvements on knowledge of health behaviour, but no effect on overweight status whereas nearly all studies included resulted in some improvement in diet or physical activity [72,109,110]. The results of the systematic reviews by Sharma [99] and Summerbell et al [72] revealed that the ineffectiveness of most intervention schemes could be due to the fact that they were not guided by a careful enough systematic evidence- based development process, and therefore they a) have been too general, not well-informed by evidence from earlier research and not rooted in behaviour change theory, b) failed to include intervention strategies tailored to the most important and modifiable determinants of the key health behaviours, c) failed to include strategies aiming to change sustainable school and family environmental factors next to health education strategies, d) did not perform a process evaluation nor e) took into account the stakeholders’ views, the contextual factors and policy framework.

Recent research attempts to explore and prevent childhood obesity in Europe

Some of the above points have been covered by recent and ongoing European research programmes in this field but none of them has systematically combined all of them by using behavioural models and behavioural research to develop insights, taking into account the policy and contextual factors, stakeholders’ role and interests or potential synergies and barriers to develop an intervention for the prevention of obesity which could potentially be transferred at a

national or European scale. Some of these programmes studying either obesity prevention or promotion of healthy energy balance-related behaviours in Europe are:

Pro Children Study: an EU funded cross-European study that focused on promotion of fruit and vegetable intake in 10 to 12 year olds [111]; Results showed positive effects of the intervention in all countries and the initial goal of a 20% increase of total fruit and vegetable intake in the intervention group compared to the control group was achieved [112].

JUMPin: a systematically developed primary-school-based intervention aiming to increase physical activity. It focuses on the use of theory, environmental changes, parental influences and cooperation with multilevel parties. The results show that JUMP-in was effective in influencing physical activity, especially among children from Grade 6 [113].

DO-iT: which aimed at preventing weight gain in three different age groups including teenagers by increasing physical activity. A school based multi component intervention program was developed with Intervention Mapping and evaluated in a cluster randomised controlled trial in the Netherlands. Results showed significant differences in body composition measures in favour of the intervention schools [114].

Kiel Obesity Prevention Study (KOPS), which was a school-based intervention to prevent overweight and obesity in prepubertal children conducted in Germany. One part of KOPS assesses the long-term effect of interventions (1) at school for all children and (2) within ‘overweight families’ for overweight children. The school-based intervention consisted of a 6 h curriculum of nutrition and physical activity. The family-based intervention consisted of a structured sports programme which has also been offered to overweight children. Results showed that the school intervention decreased the incidence and the severity of overweight [115,116];

EPODE/Ville Santé, which is a long running (ongoing) community based approach to promotion of health behaviours and obesity prevention currently involving over 124 cities across France. This EPODE project involves a string focus on children and adolescents, and preliminary results suggest that obesity prevalence is half in intervention communities as compared to control communities (www.villesante.com) [117].

HELENA project, Healthy Lifestyle in Europe by Nutrition in Adolescence (HELENA study): includes cross-sectional, crossover and pilot community intervention multicentre studies, as an integrated approach to better understand the development of non-communicable diseases during adolescence. A cross-sectional study was executed in 10 European countries. An intervention study (computer tailored physical activity advise and diet advice) was executed in 6 centres (5 countries) including 200 adolescents (13-15 year-olds) per country.

IDEFICS project, Identification and prevention of Dietary- and lifestyle-induced health effects. In Children and Infants: ongoing programme focusing on the age group of 2 to 10 years. It is implementing and evaluating prevention modules as part of a community intervention trial in eight European countries.

HOPE project, Health promotion through Obesity Prevention across Europe): The recently started (April 2007) HOPE project is an integrated analysis to support European health policy and to identify social-economic and environmental determinants of energy balance-related behaviours and also effective intervention settings to address them. However, it is not specifically interested in children and although it aims to identify effective intervention settings, it does not actually develop and/or evaluate interventions.

TigerKids: a behavioural intervention originally developed for use in preschool day care settings in in a city setting (Munich) and in a smaller town (Kaufbeuren, Germany), with low-cost, which was proven to be successful and thus highlighted in the EU White Paper on Nutrition, Physical Activity and Health (2007) as one of two European model projects for obesity prevention but the only

school-based one. Based on the positive findings, the programme is now expanding in 5000 day-care settings nationwide in Germany [118].

ENERGY: a multidisciplinary analysis of determinants and intervention schemes on extrinsic and intrinsic factors determining specific nutrition and physical activity behaviours in children and adolescents in different populations and regions. An evidence and theory-based new intervention scheme will be developed to prevent unnecessary weight gain among youth in transition from childhood to adolescence.

Partners of the ToyBox consortium are directly or indirectly involved in each of these projects.

1.2.2 Progress beyond state-of-the-art

Based on current knowledge and ongoing research, it seems that there is an emerging need to move beyond current practices. The logic steps towards this direction would be to a) identify and understand the determinants of energy balance related behaviours to establish the objectives of the intervention, b) combine these insights obtained at a local level with behavioural models and educational strategies for the design and implementation of the intervention, c) engage relevant stakeholders to ensure synergies and low-cost, increase compliance and effectiveness, improve cost-effectiveness and increase institutionalization and sustainability of the intervention at a local or national level and d) use translational research to inform policy makers and support Public Health Policy in creating supportive contexts for childhood obesity preventive actions in Europe.

This is exactly what is proposed in the ToyBox project: existing data will be analyzed and the key **behaviours related to obesity** in young children will be identified and **original behavioural research** will be conducted by executing focus groups interviews with parents and teachers to understand **at local level** the determinants of these behaviours**.** ToyBox will also perform a critical appraisal of the existing **behavioural models** and **educational strategies** applied in young children aiming to influence energy balance related behaviours. On the other hand, ToyBox will make an overview of existing contextual and legislation framework of pre-primary education and health promotion activities. Using all obtained **insights** a **multi-component school-based and family involved intervention** applicable on a European scale will be developed aiming to influence behaviours in early childhood. The new intervention developed will pool experience from previous programmes such as the TigerKids, which will be substantially improved and upgraded to facilitate local, cultural and sociodemographic needs within a European scale approach. A **cluster randomized intervention** applying the multi-component programme will be implemented. **Process, impact, outcome** but also **cost-effectiveness** will be evaluated. This **multidisciplinary approach** of the ToyBox will aim to review procedures and **dissemination activities** to contribute to the Activities of the EU Platform on Diet, Physical Activity and Health and **support decision making for European Public Health Policy**.

While obesity prevention efforts for school-aged children and adolescents are on the rise, little has been done to address the overweight epidemic during early childhood[4] [105,119,120]. Healthful behaviours are fostered by stimulating and encouraging environments in which children live and play. As shown in section B 1.1.2, studies so far have shown the importance of the early years in later food and physical activity behaviours whereas there is also accumulating evidence that certain physiological risk factors are developed in early childhood [35-39]. Currently, an obvious void exists in research programmes addressing both nutrition and physical activity for the prevention of obesity in wide population-based interventions, e.g. in the school setting.

[4] Specifically in ToyBox early childhood or young children will refer to children aged 4-6 years old.

The majority -in some countries up to almost 100%- of all young children (4-6 years old) attend either kindergartens or primary school [121]; however, limited school-based[5] research has focused on preventing obesity in this young age. An exception is the Idefics and the TigerKids programmes. Idefics has targeted a broader age range (2-10 years old) and has developed two modules of intervention, one for children 2-6 and one for 6-10 year olds. In the ToyBox, the very specific goals (energy balance related behaviours) and narrow age range allow more focus and potentially effective approaches compared to the very wide 'developmental wise' age range of 2-6 years old as well as the wide thematic area of the Idefics project, including stress, bone mass and skoliosis. Furthermore, ToyBox will include systematic reviews to identify specific energy balance related behaviours and certain risk groups with regard to them, which will guide the execution of focus groups with parents and teachers; in Idefics no such systematic approach was followed nor focus groups with teachers were executed.

On the other hand, TigerKids targets children at the same age as ToyBox but it was developed for use in Germany and therefore it cannot be considered as appropriate to use at a European level. Furthermore, energy balance related behaviours as well as their determinants or high risk groups were not extensively examined and assessed prior to the development of the intervention to allow tailor-made and most effective approaches. In addition, the contextual and legislation framework of pre-primary education as well as any conflicts or opportunities for synergies with ongoing health promotion activities (that could significantly affect the outcome of the intervention) were not examined in either IDEFICS or TigerKids project.

A recent Eurobarometer report and the White paper on Nutrition, Overweight and Obesity by the European Commission indicate that parents are regarded as the most important influence on obesogenic health behaviours among youth, while the school environment is regarded as an important setting for promotion of change in obesogenic behaviours, by offering opportunities for physical activity, providing more nutritious food and by the provision of obesity related health services [119,122]. But this potentiality can only be achieved if school based interventions take into account the contextual and legislation framework regarding pre-primary education as well as the potential role, engagement, interest and skills of relevant stakeholders.

<u>Advances the ToyBox project will bring about</u>

1. Systematic evidence-based approach combining the Precede-Proceed Model and the Intervention Mapping;
2. Careful systematic review and secondary analyses for the identification of young children's energy balance related behaviours and families' sociodemographic characteristics with higher risk for early childhood obesity;
3. New formative research to record and understand the determinants of children's energy balance related behaviours in early childhood;
4. Use of effective behavioural models and educational strategies to promote healthy weight and healthy energy balance related behaviours for the prevention of obesity;
5. Record existing policy, regulation, organization and contextual factors regarding school and school-based health promotion activities;
6. A multidisciplinary approach by a multidisciplinary team;
7. Developing an evidence-based school-based multi-component intervention addressing local needs within a European scale approach;
8. Implementing a cluster randomized intervention and evaluating process, impact and outcome;

[5] When "school" or "school-setting" is mentioned throughout the ToyBox proposal, it refers to the setting accommodating pre-primary education. In most European Union countries, this setting is kindergarten; however, in certain member states they attend the first grades of primary school (Source: Eyrydice: the information network on education in Europe, http://eacea.ec.europa.eu/portal/page/portal/Eurydice) [121].

9. Making cost estimates and reporting cost-effectiveness to support decision making for Public Health Policy;
10. Involvement of SMEs and international health promotion organisations to promote public private partnerships and ensure exploitation.

Ad 1. Systematic evidence-based approach combining the Precede-Proceed Model and the Intervention Mapping

In health promotion planning, it is important to follow a protocolised, stepwise process of development and evaluation to a) increase the likelihood that the program will reach its target audience and achieve its goals and objectives; b) help ensure the program is implemented and evaluated effectively; c) provide opportunities to involve the community and ensure the program is inclusive (in terms of gender and culture) and addresses social equity issues; and d) maximise the likelihood that the program will become sustainable . To accomplish this, the current project will make a combined use of Precede-Proceed Model and Intervention Mapping protocol; such synergy has been proposed previously by both Green & Kreuter (Precede-Proceed Model) [58] and Bartholomew et al (Intervention Mapping) [59]. The present project will apply this approach for the first time in a European-scale project aiming to promote healthy weight and healthy energy balance related behaviours for the prevention of obesity in early childhood.

Ad 2. Careful systematic review and secondary analyses for the identification of young children's energy balance related behaviours and families' sociodemographic characteristics with higher risk for early childhood obesity

So far, most original research and systematic reviews on the prevention of obesity in childhood have mostly addressed obesity indices and energy balance related behaviours of primary school children. In young children however, research mainly focuses on monitoring obesity prevalence trends while there is limited data and no systematic assessment of specific eating, sedentary and physical activity behaviours related to overweight and obesity. The ToyBox project aims to include secondary analyses of existing data available in the participating European countries to identify most relevant and important energy balance related behaviours in early childhood but also risk groups with regard to sociodemographic characteristics to ensure that all country-, cultural- and SES-specific differences are addressed.

Ad 3. New formative research to record and understand the determinants of children's energy balance related behaviours in early childhood

While young children will be the primary target audience of this project, cognitive immaturity poses a challenge in gathering formative research directly from this audience. Thus, to gain insight into young children's needs, wants and preferences, key informant interviews and focus group discussions will be conducted with secondary influencers—teachers and parents, respectively [76].

The ToyBox will be the first to execute this at a European level for the development of an evidence-based intervention; other programmes in similar age groups have not performed focus groups with teachers to identify the determinants related to the school environment (e.g. Idefics) whereas others have not retrieved such qualitative data neither from parents nor teachers prior to developing their intervention material (e.g. TigerKids).

The determinants identified from the focus groups will lead us to develop the desired and effective intervention components, which will be partly similar across all countries, and partly focusing on the country-specific determinants (cultural adaptation).

Ad 4. Use of effective behavioural models and educational strategies to promote healthy weight and healthy energy balance related behaviours for the prevention of obesity;

Behavioural models identified as effective for the prevention of obesity will provide the theoretical framework of the ToyBox intervention whereas the latest findings of research will bring out the most important educational strategies to achieve effective and sustainable

behavioural changes. The child will be seen as an active, competent co-constructor of knowledge [123] and learning will be a cooperative and communicative activity, where children construct knowledge together with others. Contents will be action-oriented, vivid and multi-sensoric. The behavioural models and educational strategies used in ToyBox will be tailored (by age, gender, etc) and combined within a socio-ecological framework to address young children and effectively result in changes of their energy balance related behaviours. Parents will also be educated on how to use these strategies at home to further support their children in improving their behaviours.

Ad 5. Record existing policy, regulation, organization and contextual factors regarding school and school-based health promotion activities

What is currently lacking in the evidence base for childhood obesity prevention reflects a broader shortage of generalizable, effective, and sustainable interventions in many areas of prevention and health risk behaviours. In order to be able to transfer and expand a research programme at a national or European level available facilities and human resources, skills and time availability, stakeholders' views and legislation, potential barriers and synergies should be taken into account; however such overview is missing from most research programmes. If these parameters are not taken into consideration, then the efficacy, effectiveness and transferability of a programme at a European level is questionable. This is another innovative aspect of the ToyBox, which will take into account also the contextual and policy framework under which schools function for the development of the intervention maximizing its potential for sustainment.

Ad 6. A multidisciplinary approach by a multidisciplinary team

The recent ministerial conference of the WHO European region concluded that the obesity epidemic is a multifactorial determined problem, requiring a multidisciplinary and multisectoral approach. This notion has been adopted in the ToyBox project and this project therefore incorporates the necessary expertise on childhood behaviours, pedagogics, nutrition, physical activity, paediatrics, psychology, health economics, policy and advocacy, school-based interventions. In addition, it incorporates the necessary range of research methodologies (systematic reviews, secondary analysis, qualitative research methods, economic modelling, multilevel and general linear modelling, evaluation of school based interventions).

Ad 7. Developing an evidence-based school-based multi-component intervention addressing local needs within a European scale approach

A multi-component school-based family-involved intervention will be produced. The experience gained by the multidisciplinary group (Academic Institute, Pedagogic Institute and a Publishing Company (SME)), who have developed, implemented and evaluated the TigerKids project in Germany as well as some of the TigerKids material will be used as a basis for the ToyBox intervention material. However all this material will be extensively revised and upgraded, aiming to facilitate in the best possible way local, cultural and sociodemographic needs within a European scale approach. Furthermore the intervention will be enriched with behavioural models, educational strategies and 'how to implement' procedures based on the best practices identified by the literature reviews. The local policy and contextual framework regarding pre-primary education will be assessed aiming to identify potential entry points and increase collaboration and synergies with relevant stakeholders.

Ad 8. Implementing a cluster randomized intervention and evaluating process, impact and outcome

The ToyBox intervention will use a clustered randomized design. Such designs are more appropriate for use at a population level, since they randomise intact social units or clusters of individuals rather than individuals themselves to different intervention groups [124]. They can increase acceptability and reduce stigmatization (everyone gets the same treatment within a cluster) but also maximize generalizability by reducing participant self-selection; thus

they may help overcome some of the problems associated with 'lifestyle intervention' trials in general practice.

In addition, process evaluation will focus on programme implementation, quality control and monitoring that explains study results [125]. Regarding impact and outcome, children's BMI changes (outcome evaluation) will not be the only focus of this intervention programme; it will primarily focus on evaluating the impact of the intervention on behaviours, such as dietary, sedentary or physical activity behaviours and their determinants (e.g. teachers' or parental knowledge, beliefs and attitudes, parental BMI, physical activity, eating and sedentary behaviours, parenting practices, school environment) [72].

Ad 9. Making cost estimates and reporting cost-effectiveness to support decision making for Public Health Policy.

Especially notable is the fact that most studies do not report cost estimates or cost-effectiveness, often a key question asked by public health policy makers who are the ones considering program adoption and diffusion [126]. In ToyBox, health economic modelling will be used to estimate cost-effectiveness of the intervention. If childhood overweight prevalence is reduced and this in turn reduces adulthood obesity, there will be possibly large economic benefits [127]; the resulting net investment (initial investment minus future savings) will be balanced with the predicted health gain (often expressed in QALYs[6] or in avoided DALYs[7]) [60].

ToyBox will use methods to increase the relevance of research findings by enhanced reporting of contextual factors that can lead to improvements in the future translation and adoption of prevention interventions [129,130]. In that sense, ToyBox will support decision making for Public health policy by providing all necessary information for policy makers and programme planners. Based on its reports and outcomes, they could consider the potential of expanding ToyBox as a whole (material and implementation procedure) on a European scale but also use the insights gained to make evidence-based and more-effective decisions on European Public Health Policy (e.g. changes in legislation for food promotion and advertising or changes in the food provided in the school setting).

Ad 10. Involvement of SMEs and international health promotion organisations to promote public private partnerships and ensure exploitation.

ToyBox involves research institutes to provide and gain the necessary scientific knowledge, international public health promotion organisations to guard and ensure the implementability and dissemination of the project results as well as small medium enterprises to promote public-private partnerships.

B 1.3 S/T Methodology and associated work plan

B 1.3.1 Overall strategy and general description

The present project is tailored to KBBE-2009-2-1-03- Behavioural models for prevention of obesity, with a particular focus on children. The ToyBox project will carry out a **multidisciplinary** analysis of existing data to identify **key behaviours** related to obesity in young children and will conduct

[6] QALYs (Quality-adjusted life years) are life years weighted by factors representing the utility or preference of individuals for health outcomes. These preferences are measured on a scale from 0 (death) to 1(perfect health). Conditions such as cardiovascular disease and cancer are not only leading to reduced life expectancy but also decrease those weighting values and therefore lead to a strong reduction in expected QALYs [128].

[7] DALYs (Disability adjusted life years) is a concept put forward by the WHO and adds the time that a person spends in disability and the time lost due to premature death. Disability is expressed as a weight between 0 (full health) and 1(the poorest possible condition) (note the difference with QALYs). The weights are arrived at on the basis of a consensus of experts and are therefore not 'utilities' (in the economic sense). In simple terms, it could be considered as a measurement of the number of healthy years of life that are lost [60].

new behavioural research to understand **why young children eat the foods they eat or why they do (or do not) participate in physical activity**. School setting is an ideal place to apply, in a potential cost-effective way, health promotion activities; however schools as well as schools' food services, health services and personnel function under a given contextual and legislation framework which varies at a local and national level. This framework as well as school personnel (as key stakeholders) are important mediators and moderators of any intervention applied in the school setting since they will be the actual facilitators of the intervention. Based on insights gained at local level, using the results of reviewing and critically appraising existing behavioural models and educational strategies on what works best with young children, but also examining the contextual framework at schools, the ToyBox programme envisions contributing in combating childhood obesity at a European scale.
The ToyBox programme will be a multi component school based and family involved intervention, aiming to facilitate the local needs within a European scale approach. This intervention will be applied in a selected number of European countries and its process, impact, outcome and cost effectiveness evaluation will support decision making for European Public Health Policy.

To achieve this, the specific objectives of ToyBox are:

1. To conduct thorough secondary analyses for the identification of children's **lifestyle behaviours** related to obesity in early childhood and perform **new behavioural research** to understand **at local level** the determinants of these behaviours (Work Packages (WPs) 2 & 3)
2. To review and evaluate existing **behavioural models** and **educational strategies** applied in young children aiming to influence energy balance related behaviours (WP4)
3. Assess the role of **school contextual framework**, legislation and stakeholders acting as mediators and moderators of the effectiveness of interventions applied in school-setting (WP5)
4. Using obtained **insights** from the above mentioned analyses, a **multi-component school-based and family involved intervention** applicable on a European scale will be developed aiming to influence behaviours in early childhood (WP6).
5. To implement a **cluster randomized intervention** applying the multi-component programme, evaluate **process, impact and outcome** and estimate its **cost-effectiveness** (WPs 7, 8 & 9)
6. To undertake dissemination activities to contribute to the Activities of the EU Platform on Diet, Physical Activity and Health and support decision making for European Public Health Policy (WP10).

In order to achieve these objectives the PRECEDE-PROCEED Model will provide the framework for the assessment, implementation and evaluation of the ToyBox intervention while an Intervention Map will provide the planning guide for the development of the intervention. Such a systematically planned, stepwise approach to develop and implement a school-based, family involved intervention to promote healthy energy balance behaviours in young children has not been attempted before on a European scale. In order to achieve these objectives, the project will be subdivided into 10 WPs.

WP1 comprises the co-ordination task to promote and ensure integrated and timely progress of the project. The development of the intervention will be based on the information obtained from WPs2-5 regarding obesity-related behaviours (WP2), their determinants (WP3), best behavioural models and educational strategies to counteract childhood obesity-related behaviours (WP4) as well as local and national legislation, regulation and stakeholders involved in the school setting and act as programme's facilitators (WP5). The outcomes of these WPs constitute the PRECEDE-phases (see Figure 1, p.10) and will be used for the development of the intervention based on the Intervention Mapping (WP6). The ToyBox intervention will be implemented in selected European countries following the PROCEED- Phases and its effectiveness (WPs 7&8) as well as its cost-effectiveness (WP9) will be evaluated and Health Promotion Policy recommendations will be developed and disseminated (WP10).

Detailed Work Plan

The overall aim of the ToyBox project is the design and validation of an evidence and theory based multi component school-based intervention aimed at promoting healthy energy balance-related behaviours in order to prevent overweight and obesity in early childhood at a European scale. The combined use of the Precede-Proceed (P-P) Model (see p. 10) with the Intervention Mapping (see

p.12) will result in a systematically planned and stepwise approach towards this direction; it will be used not only for the development of the intervention but it will also act as a guide to perform planned assessments and to implement and evaluate the intervention.
WP1 comprises the co-ordination task to promote and ensure integrated and timely progress of the project. This WP will be responsible for the overall running and implementation of the project, will carry out the administrative tasks, will be responsible for the financial and organizational management of the project, will communicate and report to the European Commission and will oversee and guard quality-controlled data management and storage. This WP will also be responsible for setting up the structure for communication through regular meetings. In addition, this WP will gather and organise all results achieved in WPs 2-5, in order to have all input for WP 6 available in one research centre and one data base that can easily be accessed by WP 6 and other participants.

The WPs 2 and 3 correspond to P-P Phases 2 and 3, respectively. These WPs will be responsible to identify young children's energy balance related behaviours and their determinants at a local level. **WP2** will perform multidisciplinary analyses of existing data on the most important children's health behaviours contributing to energy balance and execute a systematic review of the literature. It will also identify risk groups for childhood obesity based on sociodemographic characteristics. **WP3** will be responsible for executing focus groups interviews with parents and teachers of young children. This process will lead in the identification of most important predisposing (children's preferences and habits), reinforcing (parental and teachers' beliefs, attitudes, behaviours, food promotion and advertising) and enabling (resources, barriers or skills) factors determining engagement in specific energy balance related behaviours. Results will be synthesized into clear recommendations which will help to define the objectives in WP 6.

WPs 4 and 5 both correspond to P-P Phase 4; the former refers to the Behavioural Models and Educational Strategies that should be included in a health promotion programme and the latter refers to the Policy, Regulation and Organization that usually affects the integration and success of such a programme. **WP4** will explore and critically appraise successful behavioural models by means of a careful systematic literature review and it will additionally review and evaluate educational strategies used for the formation of energy balance related behaviours in early childhood. **WP5** will make an overview of the existing policies and health promotion activities concerning healthy eating and physical activity in (pre)primary school settings (age group 4-6 years). The inventory will also include what is known about obstacles and success factors that influence the development and implementation of these programmes for the target group. Finally in WP5 cross-cultural aspects of school policies will be analysed, for example looking into the national education policies, regulations and legislation on a national, regional and local level (such as the national school curriculum, bans on the sales of sweets and soft drinks in schools, local regulations on banning sweet shops in the vicinity of the school, creating safe walking and cycling tracks to school, etc). This will lead to a set of recommendations for the development and implementation of the ToyBox at a national and European level.

WPs 2-5 are all necessary to provide WP6 with the essential information for designing the new multi-component school-based and family involved intervention scheme. In **WP6**, the development of the intervention will be guided by the five steps of IM. Objectives and determinants will be identified (by WPs 2 and 3), effective methods and strategies (by WP4) will be included and the operationalization of the programme will be explored (by WP5, which will identify implementers, stakeholders and synergies).

WP7 will be devoted to the implementation (P-P Phase 5) and process evaluation (P-P Phase 6) of the newly developed intervention in six Member States, representing different regions in Europe. The intervention will be implemented in 13 schools per country and 7 other schools will serve as controls. The total sample will thus comprise around 4800 children. **WP8** will be devoted to the development and validity testing of assessment tools for eating, sedentary and physical activity behaviours and their determinants to be used for the impact (P-P Phase 7) and outcome (P-P Phase 8) evaluation of the intervention. **WP9** will analyze the cost- effectiveness of the intervention programme as described through previous WPs. A health economic model will be developed to calculate the long term impact of the programme in terms of costs and health effects.

WP10 will undertake dissemination activities for the ToyBox programme and will thereby contribute to the Activities of the EU Platform on Diet, Physical Activity and Health and also support decision making for European Public Health Policy

According to the work plan, and in accordance with the call KBBE-2009-2-1-03, the current project will make use of data from existing studies informing about energy balance-related behaviours. The different studies can be categorised as longitudinal and cross sectional observational studies, suitable for secondary analyses in WP2, where information on energy balance-related behaviours and indices of overweight in early childhood will be needed. For that purpose European data sets have been identified. The ToyBox consortium has access to most data or has already contacted persons responsible for the data and agreed to cooperate in secondary analyses. We accept only data sets that included anonymous information and for which ethical approval was given by relevant authorities. The studies used for secondary data analyses are briefly presented in Table 1.

Table 1 Studies used for secondary analyses in WP2

Study (year of study)	*Coordinator*	*Country*	*Study type*	*Age group*	*Type of variables*
POP-Study (2008-2011)	Lea Maes / Ilse De Bourdeaudhuij	Belgium	Intervention	1.5-6.0	Overweight indices; diet, PA, sedentary; SES
Galcheva S, Iotova V & Petrova K* (2007)	Violeta Iotova	Bulgaria	Cross-sectional	5.5-7.5	Overweight indices; diet, PA ; SES, parental BMI
TigerKids	Berthold Koletzko	Germany	Intervention	3.0-7.0	BMI, diet, PA; SES
GENESIS (2004-2005)	Yannis Manios	Greece	Cross-sectional	1.0-5.0	Overweight indices; diet, PA, sedentary; SES, parental BMI
Charzewska K and Weker H.** (2006)	Institute of Mother and Child Food & Nutrition Institute	Poland	Cross-sectional	4.5	Overweight indices Diet, PA, sedentary;
EnKid (1998-2000)	Lluis Serra-Majem / Javier Aranceta	Spain	Cross-sectional	2.0-20.0	Overweight indices, Diet, PA, sedentary; SES

PA denotes physical activity

*First data submitted for publication in *Pediatria* - Anthropometric and health behavioural patterns among Bulgarian pre-school children [in Bulgarian]

**First data published in *Pediatria Współczesna. Gastroenetrologia, Hepatologia i Żywienie* - Polish study on calcium and vitamin D intake in children aged 4-5 years. 2006, 8: 107-109 [in Polish]

Technological development will be part of WP1, WP6 and WP10 and concern the development of a website suitable as a communication structure between partners and a means to communicate findings to the broader public, e.g. municipal health services.

1. B 1.3.2 Timing of work packages and their components

The chart below shows the timing of the project and the different WPs.

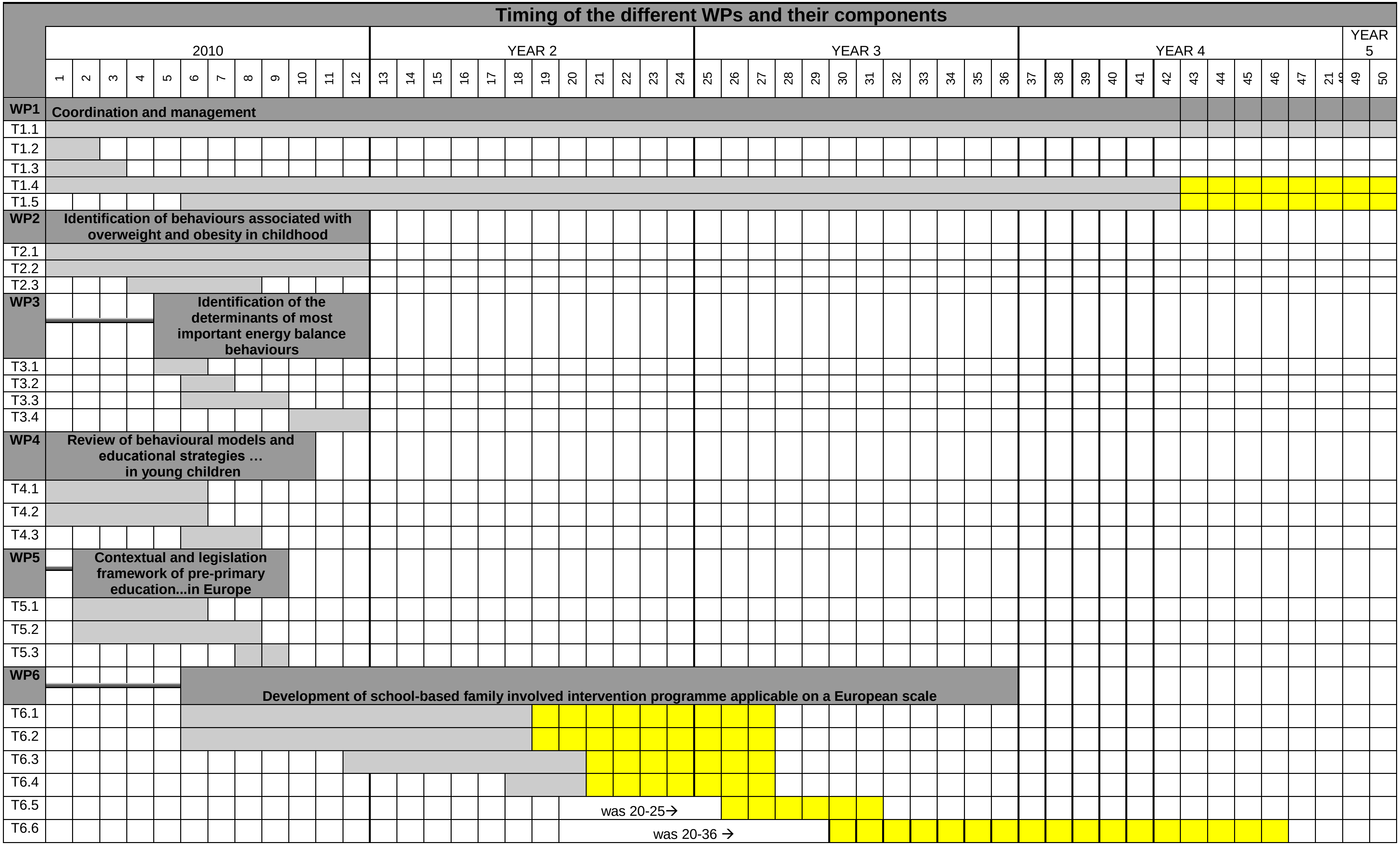
Timing of the different WPs and their components
2010
YEAR 2
YEAR 3
YEAR 4
YEAR 5
1 2 3 4 5 6 7 8 9 10 11 12 13 14 15 16 17 18 19 20 21 22 23 24 25 26 27 28 29 30 31 32 33 34 35 36 37 38 39 40 41 42 43 44 45 46 47 21 49 50
WP1 Coordination and management
T1.1
T1.2
T1.3
T1.4
T1.5
WP2 Identification of behaviours associated with overweight and obesity in childhood
T2.1
T2.2
T2.3
WP3 Identification of the determinants of most important energy balance behaviours
T3.1
T3.2
T3.3
T3.4
WP4 Review of behavioural models and educational strategies … in young children
T4.1
T4.2
T4.3
WP5 Contextual and legislation framework of pre-primary education...in Europe
T5.1
T5.2
T5.3
WP6 Development of school-based family involved intervention programme applicable on a European scale
T6.1
T6.2
T6.3
T6.4
T6.5
was 20-25→
T6.6
was 20-36 →

Timing of the different WPs and their components

	2010										2011												2012												2013												2014			
#	1	2	3	4	5	6	7	8	9	10	11	12	13	14	15	16	17	18	19	20	21	22	23	24	25	26	27	28	29	30	31	32	33	34	35	36	37	38	39	40	41	42	43	44	45	46	47	48	49	50
**WP7**																				Implementation and evaluation of a cluster randomized intervention																														
T7.1																				was 20-22																														
T7.2																				20-32																														
T7.3																				20-34																														
T7.4																																																		
**WP8**	Development, validation and training for the outcome and impact assessment tools																																																	
T8.1																																																		
T8.2																																																		
T8.3																																																		
T8.4																																																		
T8.5																																																		
T8.6																																																		
T8.7																																																		
**WP9**																				Evaluation of the cost-effectiveness of the intervention																														
T9.1																																																		
T9.2																																																		
**WP10**										Dissemination																																								
T10.1																																																		
T10.2																																																		
T10.3																																																		

2. B 1.3.3 Work package list / overview

Table B 1.3.3.a: Work Package List

Work package No	Work package title	Type of activity	Lead beneficiary No	Person-months	Start month	End month
1	Coordination and management	MGT	1	26	0	50
2	Identification of behaviours associated with overweight and obesity in childhood	RTD	4	27	1	12
3	Identification of the determinants of most important energy-balance related behaviours	RTD	3	34	5	12
4	Review of behavioural models and educational strategies to promote healthy weight and healthy energy balance related behaviours in young children	RTD	7	25	1	8
5	Policies and practices for school-based interventions in Europe	RTD	12	42	2	9
6	Development of school-based family involved intervention programme applicable on a European scale	RTD	2	106	6	46
7	Evaluation of a cluster randomized intervention	RTD	1	144,7	20	46
8	Development, validity test and training in use of assessment tools	RTD	5	26,8	1	28
9	Evaluation of the cost-effectiveness of the intervention	RTD	3	17,5	20	48
10	Dissemination	OTHER	11	29	6	50
	**TOTAL**			**478**		

B 1.3.4 Deliverables list

Table B 1.3.4a: Summary of project's main deliverables

Del. no.	Deliverable name	WP no.	Nature	Dissemination level	Delivery date
D2.1 D2.2	Protocols for the systematic review and the secondary data analysis of existing data sets	2	O	RE	2
D1.1	Information and communication system	1	O	PP/PU	3
D5.1	A standardized form for data gathering and analysing of policies and legislations at pre-primary education settings	5	O	RE	4
D2.3	A brief report with recommendations for the focus group interviews to be held in WP3	2	R	PU	5
D1.2	ToyBox Website	1	O	PP/PU	6
D1.3	Data base including all data from relevant existing studies to be used in the secondary data analyses	1	O	CO	6
D10.1	A listing of the Network of Networks	10	O	PU	6
D3.1	A protocol for the focus groups	3	O	RE	6
D4.1 D4.2	Reports on the reviews on educational strategies and psychological approaches and behavioural models used in school-based interventions	4	R	PU	6
D5.2	A standardized form for data gathering and analysing of health promotion activities policy and legislations at pre-primary education settings	5	O	RE	6
D5.3	Report on existing policies and regulations applying in pre-primary education settings	5	R	PU	6
D3.2	A standardised form for data gathering and analyses	3	O	RE	7
D2.4	A report with input and recommendations to be used for the development of the intervention	2	R	RE	8
D4.3	A report with recommendations on most important behavioural models and educational strategies to inform WP6	4	R	RE	8
D5.4 D5.5 D5.6	Reports on a) health promotion activities policy and legislations, b) ongoing health promotion activities and c) most important contextual factors affecting their success in pre-primary education settings	5	R	PU	8
D5.7	Report on good examples of policies and health promotion activities in the school setting	5	R	PU	9
D5.8	Blueprint for strategies and core components for the development of the ToyBox intervention material in WP6	5	O	RE	9
D5.9	Blueprint on what should be reported in a scientific paper presenting an intervention study	5	O	PU	9
D8.1	A report on the review of the literature on tools assessing food intake and physical (in)activity	8	R	PU	10
D3.3 D3.4	Reports on specifically identified parental determinants and teachers' focus groups	3	R	RE	12
D6.1	ToyBox Teacher's General Guide and Classroom Activities Guide: developed in English	6	O	RE	21
D6.3	ToyBox Parental Activities Guide: Developed in English	6	O	RE	21
D6.2	ToyBox Teacher's General Guide and Classroom	6	O	RE	23

	Activities Guide: Translated and adapted for use in six countries				
D8.2	Protocol(s) for planning, training and data collection	8	O	RE	26
D6.4	ToyBox Parental Activities Guide: Translated and adapted for use in six countries	6	O	RE	21
D6.5	All material prepared and ready for use for all six countries	6	O	RE	24
D6.6	Training modules for teachers in participating countries developed	6	O	RE	27
D6.7	The total set of ToyBox printed material and toys delivered in all participating countries	6	O	RE	27
D6.8	Internet material developed and put online to support the programme	6	O	RE	31
D8.3	A protocol for the collection of study data from the participating centres with an agreed format and ensuring security and confidentiality of the data will be developed	8	O	RE	25
D8.4	Databases for data entry and manual for data entry procedures will be developed for all tools described in Tasks 8.2 to 8.5	8	O	RE	28
D7.1	A protocol for the implementation and evaluation of the ToyBox intervention	7	O	RE	25
D7.2	Process evaluation tools developed	7	O	RE	26
D10.2	One Network of networks meetings	10	O	PU	24-36
D2.5<br>D2.6<br>D3.5<br>D3.6<br>D4.4<br>D4.5<br>D4.6<br>D5.10<br>D5.11<br>D5.12	Scientific papers submitted in peer reviewed journals on:<br>▪ the systematic review of EBRB<br>▪ the secondary analyses on EBRB<br>▪ the focus group research methods and findings<br>▪ the systematic review of the most important parenting practices and home environmental characteristics<br>▪ most important educational strategies and psychological approaches<br>▪ the systematic review of behavioural models used in school-based interventions<br>▪ the evidence base, available and emerging, to help inform policy makers across Europe develop and implement strategies to prevent childhood obesity<br>▪ existing health promotion activities<br>▪ obstacles and success factors for developing and implementing health promotion policies and programmes<br>▪ strategies and recommendations for policy makers	2,3,4,5	O	PU	32-40
D6.9	Report on any changes made to the ToyBox material by country	6	R	RE	46
D6.9	Final versions of ToyBox material in all languages (incl. English) and ToyBox with material for classroom activities	6	O	PU	36
D8.5<br>D8.7	Scientific papers submitted in peer reviewed journals on:<br>▪ the review of the literature on tools assessing food intake and physical (in)activity<br>▪ the development and validity testing of the assessment tools	8	O	PU	16<br>36
D8.6	Report on the validity and harmonization process of the tools	8	R	PU	36

D9.1	Interim report with the cost of the intervention	9	R	PU	43
D7.3	Report on the process, impact and outcome evaluation at a country and European scale	7	R	PU	48
D1.4	Data base including all data and results from WPs 2-9	1	O	RE	50
D1.5	Complete sets of ToyBox intervention materials in six languages	1	R	PU	50
D10.3	Matrices of key scientific findings against specific policies and interventions according to their contexts	10	O	PU	50
D10.4	A list with at least twenty (20) items that appeared in independent public media	10	O	RE	50
D10.5	A list with at least six (6) scientific papers submitted in a peer reviewed journals or conference presentations	10	O	RE	50
D10.6	Policy recommendation fact sheets for EU, national and local policy-makers	10	O	PU	50
D7.4	Report on mediators and moderators of the intervention at local and EU level	7	R	PU	50
D9.2	Projection model in MS Excel	9	O	PU	46
D9.3	A full report containing the health economic model description, data inputs and results	9	R	PU	48
D9.4	A brief report presenting key findings regarding long term costs, effects and cost-effectiveness of the programme to inform policy makers	9	R	PU	48

B 1.3.5 Work package descriptions

**Work package number**	1	**Start date or starting event:**				Month 1
**Work package title**	Coordination and management					
**Activity Type**	MGT+RTD+OTH					
**Participant number**	**1**					
**Person-months per beneficiary:**	26					

Objectives

The aim of this WP is the coordination, management and administration of the project

1.1 Support the overall running, and coordinate the integrated progress of the project, including financial management

1.2 Set up a structure for communication

1.3 Store and manage all data obtained in WP 2-9

1.4 Retrieve and administer all results from WP2-9

1.5 Communicate with and report to the European Commission

Description of work

This WP will comprise the co-ordination task to promote and ensure integrated and timely progress of the project communication. For this purpose this WP will set up a communication and feedback structure, apply a quality control infrastructure, prepare and organise progress meetings, coordinate timely reporting of content, deliverables and finance, and generally support the steering Committee and WPs of ToyBox. Furthermore, it is of importance that all results from WP2-5 will be stored centrally in databases that can easily be accessed by all WP 6 participants. This will be time efficient and it will guarantee that all results are reported in the same way. Formats for data storage will be decided upon in close collaboration with the relevant WP leaders. It is also of great importance that all data obtained in WP 2-9 are stored and managed centrally after initial data management and analyses within the subsequent WPs. This WP will also acquire the data for the secondary data analyses which will be more efficient since different WPs will use the same datasets.

The work in this WP will consist of five Tasks:

Task 1.1 (month 0- 50) MGT
Support the overall running of the project, including the financial management

1.1.1 Coordinate timely reporting of content, deliverables and finance, and generally support the steering Committee and WPs of the project

1.1.2 Allocate the budget to different partners and activities and distribute payments received from the Commission to the partners.

1.1.3 The coordinator will also manage changes to the membership of the consortium.

Task 1.2 (month 0-2) OTH (Dissemination)
Set up a structure for communication

1.2.1 Develop the programme's website to include a restricted area (for ToyBox partners only), a restricted area for teachers of intervention schools and a public-access area; an information system will be developed for the restricted area to enable communication between partners (e.g. forum) and upload of relevant documents.

1.2.2 Include important documents (i.e. minutes of the meetings, tools, etc) to the ToyBox partners' restricted area of the website;

1.2.3 Include a brief introduction and guide for the intervention, ToyBox material, Frequently Asked Questions & Answers and useful contact details for the local teams to the teachers restricted area of the website;

1.2.4 Update the website with relevant documents and results;

1.2.5 Prepare an overview for plenary meetings and regular telephone conferences.

Task 1.3 (month 0-3) RTD
Acquire existing datasets for secondary data analyses in WP 2.

1.3.1 Contact owners of existing datasets and ask for the data;
1.3.2 Define the data quality;
1.3.3 Develop a data base to store datasets from all relevant existing studies; if additional datasets beyond the already identified ones, then distribute payments received from the Commission to the partners.
1.3.4 Send data to WP2 leader, where the data will be checked and restructured.

Task 1.4 (month 0-50) RTD
Develop and manage data storage and accessibility

1.4.1 Define formats to deliver cleaned data and final datasets for central storage concerning literature reviews (WPs 2, 4 and 5) including information on search strategies, references of the retrieved papers and relevant tables;
1.4.2 Define formats to deliver data concerning focus group interviews (WP 3) for central storage including transcription;
1.4.3 Define formats to deliver cleaned and final datasets from the evaluation of the ToyBox intervention (WPs 7 and 8) including codebooks and recoding, scale formation and other data reduction syntax;
1.4.4 Develop a data base to store datasets from all relevant WPs and ensure easy access for all partners.

Task 1.5 (month 6-50) MGT
Communicate and report to the European Commission

Write and edit annual reports (month 12 and 30) and final reports, including a full description of the school-based intervention programme aiming to prevent childhood obesity that will be ready for implementation across Europe, based on the reports from WP 2-10.

Deliverables

D1.1. Information and communication system (month 3)
D1.2. Website including a restricted area (for ToyBox partners), a restricted area for teachers of intervention schools and a public-access area (month 6)
D1.3. Data base including all data from relevant existing studies to be used in the secondary data analyses (month 6)
D1.4. Data base including all data and results from WPs 2-9 (month 50)
D1.5. Complete sets of ToyBox intervention materials (electronic and hard-copy format and material for classroom activities and games) in six languages (month 50)

**Work package number**	2		**Start date or starting event:**			Month 1
**Work package title**	Identification of behaviours associated with overweight and obesity in childhood					
**Activity Type**	RTD					
**Participant number**	**4**	3	1	2	5	9	10
**Person-months per beneficiary:**	**18**	4	1	1	1	1	1

Objectives

2.1 Identification of the most important energy balance related behaviours among young children that are associated with overweight and obesity.
2.2 Identification of risk groups (family demographics, socioeconomic indices and parental BMI) with regard to children's energy balance related behaviours, overweight and obesity.
2.3 Translation of findings into practical input for WP3 and WP6.

Description of work

In this WP we will focus on three main energy-balance related behaviours i.e. physical activity, sedentary and eating behaviours and examine which specific sub-behaviours are most important regarding children's overweight and obesity. The review and secondary analyses will focus on relevant sub-behaviours of eating (e.g. skipping breakfast, snacking, soft drink consumption, fruit and vegetable consumption, eating in front of TV, etc), physical activity (active transport, leisure time physical activity, sports, school time physical activity, domestic activities, etc) and sedentary behaviours (television viewing, internet use, computer games, reading, etc) and their relationship with overweight and obesity.

To ensure easy flow of the text the term 'behaviour' will be used in all WPs and deliverables to describe behaviours and sub-behaviours.

VUmc will focus on dietary and sedentary behaviours, while UGent will focus on the physical activity behaviours.

The work in this WP will consist of three main Tasks:

Tasks 2.1 (month 1-12) (VUmc, UGent)
Systematic review on energy balance related behaviours in young children and identification of risk groups with regard to these behaviours
Identify the most important energy balance related behaviours of young children and certain risk-groups in the population with regard to these behaviours. This information will be used in WP3 and WP 6.

2.1.1 Develop a protocol for a systematic review of the existing literature, including the development of inclusion criteria (including a list of exposures/behaviours and outcomes of interest) and method of quality assessment. Develop and running a search strategy to identify relevant primary studies. Only primary studies reported from 1990 will be included, since behaviour patterns have been subject to change since then;
2.1.2 Review abstracts of papers identified from the search (Task 2.1.1) for possible inclusion in the review by two independent reviewers;
2.1.3 Retrieve full copies of papers that were identified for possible inclusion and, after reading the papers, make a final decision about which studies should be included in the review;
2.1.4 Extract relevant data from studies that will be included in the review;
2.1.5 Score the methodological quality of the included studies by two independent reviewers;
2.1.6 Conduct data analysis;

2.1.7 Write the report of the systematic review, including an assessment of 1) the most important energy balance-related behaviours of young children, that are associated with overweight and obesity in early childhood, and 2) risk groups with regard to energy balance related behaviours, overweight and obesity (based on family demographics and socioeconomic indices and parental BMI);

2.1.8 Report data and results to WP1 to store and manage the data and the results.

Task 2.2 (months 1-12)
Conduct secondary data analyses

Secondary data analyses will be conducted using existing data sets from participating countries on energy balance related behaviours among young children.

2.2.1 Identify available and appropriate studies in participating countries for the secondary data analysis;

2.2.2 Develop a protocol for accumulating existing datasets from participating countries on overweight and obesity indices and children's energy balance behaviours related to overweight and obesity in early childhood. If additional datasets are needed, then contact coordinator who is responsible for acquiring them (Task 1.3);

2.2.3 Conduct the secondary data analysis in the selected studies (identified by Task 2.2.1). Where possible within the timeframe, data will be transferred following anonymisation of the data to VUmc, and analysed. Where it is more appropriate for the analysis to be carried out by academic colleagues based at sites (other Partner sites or other Institutions) that host the data (e.g. because of ethical issues associated with transfer of data), VUmc will co-ordinate the analysis at these sites;

2.2.4 Write the report of the secondary data analysis, to include an assessment of the most important energy balance related behaviours related to overweight and obesity in early childhood;

2.2.5 Report data and results to WP1 to store and manage the data and the results.

VUmc will conduct Task 2.2 but in close co-operation with WP participants and others based at other sites hosting the data for the primary studies selected for the secondary data analysis.

Task 2.3 (month 4-8)
Translate findings into practical input for WP3 and WP6.

2.3.1 Discuss with WP participants the findings from the systematic review and secondary data analysis;

2.3.2 Formulate draft recommendations regarding the most important energy balance-related behaviours of young children. These will inform the content and formation of the focus groups interviews (WP 3);

2.3.3 Formulate recommendations on risk groups with regard to energy balance related behaviours and/or overweight and obesity that need to be included in the focus group interviews (WP3);

2.3.4 Write the report of the recommendations to inform the development of the intervention material (WP6).

Task 2.3 will be co-ordinated by Partner 4 (VUmc), but all WP participants will be involved in the drafting of and agreement on the recommendations.

Deliverables

D2.1. A protocol for the systematic review on the most important energy balance behaviours and sub-behaviours and the identification of risk groups (based on family demographics, socioeconomic indices and parental BMI) that are associated with overweight and obesity

	in young children (Task 2.1) (month 2)
D2.2.	A protocol for the secondary data analysis of existing data sets in the participating countries on energy balance behaviours associated with overweight and obesity in young children (Task 2.2) (month 2)
D2.3.	A brief report with recommendations for the focus group interviews to be held in WP3, regarding the energy-balance sub-behaviours that have to be discussed and the groups to be recruited (Task 2.3.2 and Task 2.3.3) (month 5)
D2.4.	A report with input and recommendations to be used for the development of the intervention (Task 2.3) (month 8)
D2.5.	A scientific paper submitted in a peer reviewed journal on the systematic review on the most important energy balance behaviours that are associated with overweight and obesity in young children (identifying risk groups based on family demographics, socioeconomic indices and parental BMI) (Task 2.1) (month 32)
D2.6.	A scientific paper submitted in a peer reviewed journal on the secondary analyses on the most important energy balance behaviours associated with overweight and obesity of young children in European member states (identifying risk groups based on family demographics, socioeconomic indices and parental BMI) (Task 2.2) (month 32)

Work package number	3	Start date or starting event:		5		
Work package title	Identification of the determinants of most important energy-balance related behaviours					
Activity Type	RTD					
Participant number	**3**	1	2	5	9	10	4
Person-months per beneficiary:	18	3	3	3	3	3	1

Objectives

3.1. Carrying out a qualitative study to identify the determinants of the behaviours found in WP2 to be related with overweight and obesity in young children

3.2. Identification of the most important parenting practices and home environmental characteristics that are associated with overweight and obesity in young children

3.3. Provision of practical input, recommendations and guidance for the development of the intervention in WP6

Description of work

In WP3 qualitative methods will be used to detect critical determinants of the identified specific behaviours in WP2. Focus groups interviews will be executed with parents and teachers. The results of WP3 will provide information of predisposing (e.g. children's food preferences), reinforcing (e.g. parental and teachers' behaviours, knowledge, beliefs and attitudes, food promotion and advertising, parental and teachers' practices) and enabling factors (e.g. time constraints, access to safe playgrounds) determining children's behaviours, which will be used for the development of the of the intervention material in WP6.
The few studies available on determinants in this young age group can lead us in developing the questioning routes for the qualitative research. Focus group interviews with parents and teachers are chosen for several reasons. The flexible questioning and synergetic effect of group conversations increases the likelihood that data and ideas will be produced that would remain uncovered with other methods (e.g. one on one interviews, self-reported questionnaires. By executing focus groups in the different countries it will be possible to draw conclusions about similarities and differences between countries [131]. These focus groups will furthermore be valuable to generate ideas about possible effective intervention strategies using the intervention mapping protocol [59]. As a consequence these focus groups will provide us with information of predisposing, reinforcing and enabling factors determining children's behaviours and actual environment taking into account the views of important stakeholders such as the parents and teachers.

We hypothesize that determinants will be partly similar across European countries, partly country-specific and partly family-sociodemographic specific. All determinants identified in WP3 will be included in the intervention material to facilitate in the best possible way the local, cultural and sociodemographic needs within a European scale approach.

Task 3.1 (month 5-6) (UGent, VUmc)
Review on determinants of energy balance related behaviours

3.1.1 A literature search will be performed on determinants of the behaviours found in WP2 to be related to overweight and obesity in young children. The review will include personal (e.g. eating preferences, neophobia), social (e.g. rules about TV viewing and snacking, parental support, parents being physically active together with their children) and environmental (e.g. availability and opportunities for healthy energy balance related behaviours, safety) determinants. The results of this review will inform Task 3.2 on potential determinants that should be discussed during the focus groups interviews.

Task 3.2 (month 6-7)
Develop a protocol for the focus group research

3.2.1. Write a design and protocol for the focus group research based on the literature search. A protocol will be developed for focus groups in parents and a different protocol for focus groups in teachers.
3.2.2. Develop a country specific plan for the focus groups in parents and teachers in each country.
3.2.3. The English version of the protocol will be sent to all WP partners. The protocol will then be translated and culturally adapted for use in the six countries participating in WP6.
3.2.4. Training of the partners in executing the focus group interviews.
3.2.5. Development of a framework for data gathering and analysing the focus group interviews across countries.

Task 3.2 will be co-ordinated by Partner 3 (UGent), but all WP participants will be involved.

Task 3.3 (month 6-9)
Execution of focus groups

3.3.1 Recruitment of parents and teachers in each country: each focus group consists of 6-8 parents or 4-8 teachers, respectively. Special attention will be paid to gender, age and SES heterogeneity.
3.3.2 Conduct focus groups interviews following the protocol. At least 4 groups of parents and 3 groups of teachers will be executed per country.
3.3.3 Each country reports the results of the focus group interviews with parents and teachers using the standardised forms in English.

Task 3.3 will be co-ordinated by Partner 3 (UGent), but all WP participants (except VUmc) will be involved.

Task 3.4 (month 10-12) (UGent)
Analysis of focus groups data

3.4.1 Analyse the data according to the data analysis plan. Write reports on focus group research, for parents and teachers to include important determinants of the behaviours identified in WP2 to be related with overweight and obesity in young children and promising strategies to involve parents and teachers in school-based interventions.
3.4.2 Translate findings into recommendations for the development of the intervention (WP6).

Deliverables

D3.1. A protocol for the focus groups (Task 3.2) (month 6)
D3.2. A standardised form for data gathering and analyses (month 7)
D3.3. A report by country on specifically identified parental determinants which need to be addressed and tackled in the Parental Activities Guide (WP6). Specific text, phrases on how to be communicated and addressed will be included (Task 3.4) (month 12)
D3.4. A report from teachers' focus groups, with identified determinants by country which need to be addressed and tackled in the Teacher's General Guide (WP6). This report will further contain information and guidance for WP6 on issues raised by the teachers regarding their self-efficacy, information material, training and support they might need to be provided to improve their self-efficacy and feel confident and motivated to deliver the intervention (Task 3.4) (month 12)
D3.5. A scientific paper submitted in a peer-reviewed journal on the focus group research methods and findings (Tasks 3.2-3.4) (month 32)
D3.6. A scientific paper submitted in a peer-reviewed journal on the systematic review of the most important parenting practices and home environmental characteristics that are associated with overweight obesity in young children (Task 2.2) (month 32)

**Work package number**	4			**Start date or starting event:**			1		
**Work package title**	Review of behavioural models and educational strategies to promote healthy weight and healthy energy balance related behaviours in young children								
**Activity Type**	RTD								

**Participant number**	**7**	14	15	8	3	1	2	5	9	10
**Person-months per beneficiary:**	**10**	2.5	2.5	3	2	1	1	1	1	1

Objectives

4.1. Identification and critically appraisal of educational strategies and psychological approaches explaining young children's acquisition and formation of energy-balance related behaviours, and facilitating their management.

4.2. Systematic review of behavioural models used in school-based interventions in pre-primary and primary education settings for the prevention of obesity

4.3. Translation of findings into practical evidence-based input for WP 6

Description of work

WP4 will be concerned with reviewing the evidence for the effectiveness of educational strategies, and behavioural models, in relation to healthy eating, physical activity and energy-balance related behaviours of young children. ***Behavioural models*** (e.g. Social Cognitive Theory, the Socio-Ecological Model, the Theory of Planned Behaviour and the Social Marketing) provide the theoretical framework for the design and implementation of school based interventions whereas ***educational strategies*** (e.g. number of exposures to food, methods of encouragement, or 'learning to like' procedures) are practical plans of action that parents and teachers can use to help children achieve the desired behaviours.

Knowledge from the findings will then be transferred to WP6, to ensure the best evidence-based design for a school-based family involving intervention. The approach will consist mainly of two tasks:

Task 4.1 will form a critical narrative review of the literature composed of two parts: a) a review of psychological approaches (theoretical explanations and their evidence) on young children's acquisition and formation of energy-balance related behaviours, including social and biological theories of learning and motivation, and understanding individual differences in risk for obesity-related behaviours; b) a review of ***educational strategies*** used by teachers, parents and health professionals to alter these learned behaviours toward a healthy energy-balance related outcome.

Task 4.2 will form a systematic review focusing on ***behavioural models*** used as theoretical framework for the design and implementation of school based interventions (both at pre-primary and primary education settings) aiming to prevent obesity. The knowledge accumulated from these reviews will then be formulated and transferred to WP6, to ensure the best evidence-based intervention that is practicable in young children (Task 4.3).

Task 4.1 (months 1-6) (RoU, ULU, IFP)

Critical narrative review of educational strategies and psychological approaches explaining young children's acquisition and formation of energy-balance related behaviours, and facilitating their management

4.1.1. Conduct a literature search of evidence underlying psychological approaches on how young children acquire their eating and activity habits, including meal sizes and food likes and dislikes and the implications for the development of individual risk factors for obesity.

4.1.2. Conduct a literature search of evidence for the effectiveness of educational strategies (e.g.

number of exposures to food, methods of encouragement) practised by parents, teachers and health professionals in order to manage young children's energy balance related behaviours.

4.1.3. Critically appraise the compatibility or otherwise of the findings from these two review processes.

4.1.4. Write the report of the reviews, including proposals for evidence-based strategies that modify children's behaviour to reduce their risk of obesity.

4.1.5. Report and transfer information, findings and recommendations to WP1.

Task 4.2 (months 1-6) (UDUR)
Systematic review of behavioural models used in school-based interventions in pre-primary and primary education settings for the prevention of obesity.
A systematic review will be conducted in the existing literature focusing on behavioural models used in school-based interventions in pre-primary and primary education settings for the prevention of obesity.

4.2.1. Develop a protocol for a systematic review of the existing literature, including the development of inclusion criteria and method of quality assessment;
4.2.2. Develop and run a search strategy to identify relevant primary studies.
4.2.3. Review abstracts of papers identified from the search (tasks 4.1.2) for possible inclusion in the review by two independent reviewers;
4.2.4. Retrieve full copies of papers that were identified for possible inclusion in the review from Task 4.1.3 and, after reading the papers, make a final decision about which studies should be included in the review;
4.2.5. Extract relevant data from studies that will be included in the review;
4.2.6. Score the methodological quality of the included studies by two independent reviewers;
4.2.7. Make decision if retrieved studies allow a meta-analytic review approach and if so conduct meta-analytic data analysis;
4.2.8. Write the report of the systematic review, including an assessment of quality of the included studies;
4.2.9. Report and transfer data and results to WP1.

Task 4.3 (months 6-8)
Translate findings into practical input for WP 6
4.3.1. Discuss with partners the findings from the systematic review (Task 4.1) and the review in Task 4.2;
4.3.2. Formulate recommendations regarding the most important components of energy balance related behavioural interventions at school setting for young children;
4.3.3. Formulate recommendations regarding the most important educational strategies for the formation of desired energy balance related behaviours in young children;
4.3.4. Make a synthesis of the recommendations formed in Tasks 4.3.2 and 4.3.3. The combined recommendations will inform the development of the intervention in WP 6.

Task 4.3 will be co-ordinated by Partner 7 (UDUR), but all WP participants will be involved in the drafting of and agreement on the recommendations.

Deliverables
D4.1. A report on the reviews on educational strategies and psychological approaches explaining the acquisition and formation of energy-balance related behaviours and facilitating their management (Task 4.1) (month 6);
D4.2. A report on behavioural models used in school-based interventions in pre-primary and primary education settings for the prevention of obesity (Task 4.2) (month 6);

D4.3.	A report with recommendations on most important behavioural models and educational strategies for the formation of desired eating and physical activity behaviours in young children to inform WP6 (Task 4.3) (month 8).
D4.4.	A scientific paper submitted in a peer-reviewed journal on most important educational strategies and psychological approaches explaining the acquisition and formation of energy-balance related behaviours and facilitating their management (Task 4.1) (month 32)
D4.5.	A scientific paper on the systematic review of behavioural models used in school-based interventions in pre-primary and primary education settings for the prevention of obesity (Task 4.2) (month 32)
D4.6.	A scientific paper on the evidence base, available and emerging, to help inform policy makers across Europe develop and implement strategies to prevent childhood obesity (Task 4.3) (month 32)

**Work package number**	5	**Start date or starting event:**					2	
**Work package title**	Contextual and legislation framework of pre-primary education and school-based health promotion activity in Europe							
**Activity Type**	RTD							
**Participant number**	**12**	6	3	1	2	5	9	10
**Person-months / beneficiary**	**18**	10	9	1	1	1	1	1

Objectives

5.1. Identification of legislation and policies for pre-primary education settings in the participation countries.
5.2. Overview of existing health promotion activities in pre-primary education at a local, regional, national and European level and to identify the role of existing stakeholders
5.3. Identification of obstacles and success factors for developing and implementing health promotion policies and programmes in pre-primary education settings taking into account the cross-cultural aspects of school policies and systems.
5.4. Outline of strategies and recommendations for improving the development and implementation of interventions based at pre-primary education settings

Description of work

Schools as well as school-based health promotion activities, food services and personnel function under a given contextual and legislation framework which varies at a local and national level. This contextual framework as well pre-primary education personnel (key stakeholders) are important moderators and mediators of any intervention applied in the pre-primary education setting since they will be the actual facilitators of the intervention.

In WP5 existing legislation and policies with regard to the above will be identified. In addition, existing initiatives and programmes concerning healthy eating and physical activity in pre-primary education settings (for age group 4-6 years) but also obstacles and success factors that influence the development and implementation of these programmes will be overviewed in each country participating in WP6.

Task 5.1 (month 2-6) (NIGZ, HiOA)
Overview of policies and regulations in pre-primary school settings in Europe

5.1.1. Development of a framework for data gathering and analysing of policies and legislations across countries (such as the national school curriculum, number of children per teacher, bans on the sales of sweets and soft drinks in kindergartens, local regulations on banning sweet shops in the vicinity of the kindergarten, creating safe walking and cycling tracks to kindergarten, food promotion and advertising in kindergarten setting, etc);
5.1.2. Cross-cultural aspects of pre-primary policies and regulations will be recorded and analysed on a national, regional and local level. The standardized form developed in 5.1.1 will be used for the collection of the data;
5.1.3. Write a report identifying these policies at a European scale.
5.1.4. Report and transfer data and results to WP1.

Task 5.2 (month 2-8) (UGent, NIGZ)
Overview of health promotion activities at pre-primary school settings in Europe and identification of the contextual factors affecting their success

5.2.1. Development of a framework for data gathering and analysing of policies and practices regarding health promotion activities in pre-primary education (such as criteria for a teacher to implement health promotion activities, material implemented, timeframe of implementation, etc);
5.2.2. Implementation of this standardized form to retrieve data on policies and practices regulating health promotion activities in pre-primary education;

5.2.3. NIGZ will make an overview of existing health promotion activities in pre-primary education at a local, regional, national level, making use of the 'HEPS inventory tool' (available October 2009). The tool is developed as part of the HEPS project (3 year project on developing and implementing healthy eating and physical activity in pre-primary education settings, period 2008-2011, funded by DG Sanco and led by NIGZ, www.hepseurope.eu) and consists of a set of quality criteria for school based interventions. These criteria will include demonstrated effectiveness, systematic whole school approach, efficiency of the programme, suitability for education and use in the classroom, level of participation and diversity;

5.2.4. Obstacles and success factors will be identified by UGent for the development and implementation of health promotion activities in school setting (as identified in Task 5.2.1) taking into account the cross-cultural aspects of school policies and systems. Additional analysis will be carried out for the target group with specific reference to who approves the implementation of health promotion programmes, who funds them, who implements them, who is monitoring or evaluating them, who are the potential stakeholders, which are the inhibiting and promoting factors, which factors ensure sustainability of the programme and what cross-cultural aspects of school policies and systems can be identified;

5.2.5. A report on ongoing health promotion activities in pre-primary education settings at local, regional and national level (NIGZ);

5.2.6. A report on most important contextual factors affecting success of health promotion activities in pre-primary education settings on a European scale (UGent).

5.2.7. Report and transfer data and results to WP1.

Task 5.3 (month 8-9) (NIGZ, HiOA, UGent)
Outline recommendations for the development and implementation of interventions at school setting

5.3.1. Identify good examples of policies and health promotion activities in the school setting among the ones collected in Tasks 5.1 and 5.2 as well as through the European Network for Nutrition and Physical Activity. These will be presented in a report form.

5.3.2. To outline strategies and recommendations for improving the development and implementation of interventions at pre-primary education settings.

5.3.3. To outline a blueprint on what should be reported in a scientific paper presenting an intervention study.

5.3.4. Development of a report with successful and sustainable interventions for pre-primary education settings, leading to a blueprint for the core components of the ToyBox, including recommendations for national implementation.

Task 5.3 will be co-ordinated by Partner 12 (NIGZ), but all WP participants will be involved.

Deliverables

D5.1. A standardized form for data gathering and analysing of policies and legislations at pre-primary education settings (Task 5.1.1) (month 4)

D5.2. A standardized form for data gathering and analysing of health promotion activities policy and legislations at pre-primary education settings (Task 5.2.1) (month 6)

D5.3. Report on existing policies and regulations applying in pre-primary education settings at local, regional and national level (Task 5.1.3) (month 6)

D5.4. Report on health promotion activities policy and legislations in pre-primary education settings at local, regional and national level (Task 5.2.2) (month 8)

D5.5. Report on ongoing health promotion activities in pre-primary education settings at local, regional and national level (Task 5.2.3) (month 8)

D5.6. Report on most important contextual factors affecting success of health promotion activities in pre-primary education settings in Europe (Task 5.2.4) (month 8)

D5.7. Report on good examples of policies and health promotion activities in the school setting

	(Task 5.3) (month 9)
D5.8.	Blueprint for strategies and core components for the development of the ToyBox intervention material in WP6 (Task 5.3) (month 9)
D5.9.	Blueprint on what should be reported in a scientific paper presenting an intervention study (Task 5.3) (month 9)
D5.10.	A scientific paper submitted in a peer reviewed journal on existing health promotion activities in pre-primary education settings at a local, regional, national and European level identifying the role of existing stakeholders (Task 5.2.3) (month 40)
D5.11.	A scientific paper submitted in a peer reviewed journal on obstacles and success factors for developing and implementing health promotion policies and programmes in pre-primary education settings (Task 5.2.4) (month 40)
D5.12.	A scientific paper submitted in a peer reviewed journal on strategies and recommendations for policy makers for improving the development and implementation of school-based health promotion activities for pre-primary education in Europe (month 40)

**Work package number**	6		**Start date or starting event:**				6				
**Work package title**	Development of school-based family involved intervention programme applicable on a European scale										
**Activity Type**	RTD										
**Participant number**	**2**	13	8	3	4	1	5	9	10	7	14
**Person-months per beneficiary**	**32**	19	12	9	2	7	7	7	7	2	2

Objectives

6.1. Development of an intervention programme and relevant material for young children to be applied at the school setting in different European countries.
6.2. Development, production and adaptation of the material in the different languages.
6.3. Provision to the research centres, implementing the intervention in WP7, of the developed material, all necessary training and guidance on how to transfer this knowledge and expertise in the local schools and teachers.

Description of work

In this WP, a multi-component school-based family-involved intervention will be produced. The experience gained by the three partners (LMU, IFP, AOK-Verlag) who have developed, implemented and evaluated the TigerKids project in Germany as well as some of the TigerKids material will be used as a basis for the ToyBox intervention material. However all this material will be extensively revised and upgraded to incorporate the input and findings from WPs 2-5, aiming to facilitate in the best possible way local, cultural and sociodemographic needs within a European scale approach (WPs 2-3). Furthermore the intervention will be enriched with behavioural models, educational strategies and 'how to implement' procedures based on the best practices identified by the literature reviews, the local policy and contextual framework regarding pre-primary education in the participating countries (WPs 4-5). As soon as the English version has been completed, it will be sent to the participating countries to be translated, culturally adapted and tested for suitability and acceptability. During this process, pre-primary teachers and representatives from national Educational Councils (or other equivalent Entities) will be engaged to ensure acceptability of the ToyBox material and intervention programme and increase their support throughout the implementation period (WP7).

The TigerKids material was chosen to serve as the basis for the ToyBox intervention material. No person-months have been allocated for the translation of the TigerKids material into English since this is a deliverable of an ongoing Grant by RoU.

The ToyBox material will consist of a) Teacher's General Guide, b) Classroom Activities Guide and c) Parental Activities Guide. All printed material as well as a wooden train for the structural exploration of food, food models, board games, a hand-puppet, physical activity equipment, etc. will be included in a box, specifically designed for the project (ToyBox).

Task 6.1 (month 6-27)
Development of the ToyBox Teacher's General Guide

6.1.1. The Teacher's General Guide will provide an overview of the programme and its objectives and some general information and background knowledge regarding the activities implemented in the school setting. The material will be based on the most effective behavioural models and educational strategies (identified in WP4) aiming to address and tackle children's energy balance related behaviours and their determinants as identified in WPs 2 and 3.
6.1.2. The final version of the ToyBox information material for the teacher as prepared in Task 6.2.1 will be translated and culturally adapted for all participating countries (Bulgaria, Belgium, Germany, Greece, Poland, Spain). During local adaptation period, engaging local stakeholders to increase acceptability of the ToyBox material and intervention programme.

6.1.3. Prior to final printings (Task 6.1.4), each country will pilot-test the produced material
6.1.4. Printing of the material and delivery in all participating countries

Task 6.1 will be co-ordinated by Partner 2 (LMU), but all WP participants will be involved.

Task 6.2 (month 6-27)
Development of the ToyBox Classroom Activities Guide

6.2.1. The ToyBox Classroom Activities Guide based on the input and the results obtained by WPs 2-4. This material is estimated to include two or three books with activities for use in the classroom, a wooden train for the structural exploration of food, food models, board games, a hand-puppet, physical activity equipment, etc. All this will be included in a box, specifically designed for the project (ToyBox).
6.2.2. The final version of the ToyBox material for each country as prepared in Task 6.2.1 will be translated and culturally adapted for all participating countries (Bulgaria, Belgium, Germany, Greece, Poland, Spain). During local adaptation period, engaging local stakeholders to increase acceptability of the ToyBox material and intervention programme.
6.2.3. Printing of the material and delivery of both printed material and ToyBox in all participating countries.

Task 6.2 will be co-ordinated by Partner 2 (LMU), but all WP participants will be involved.

Task 6.3 Development of the ToyBox Parental Activities Guide (month 12-27)

6.3.1. The Parental Activities Guide of the ToyBox intervention will be based on the input and results obtained in WPs 2, 3 and 4. WP2 will provide information on specific sub-behaviours that should be addressed whereas WP4 will inform on educational strategies both to motivate parents to adopt desired behaviours but also how parents that parents could help their children form desired behaviours. The results of WP3 will also be included to address the specific determinants (parental and home environmental) identified at a European scale to affect young children’s behaviour. Specific strategies and methods will be provided to the parents to overcome barriers and enhance facilitators of healthy energy-balance related behaviours in young children.
6.3.2. The final version of the ToyBox Parental Activities Guide for each country as prepared in Task 6.4.1 will be translated and culturally adapted for all participating countries (Bulgaria, Belgium, Germany, Greece, Poland, Spain). During local adaptation period, engaging local stakeholders to increase acceptability of the ToyBox material and intervention programme.
6.3.3. Printing of the material and delivery in all participating countries.

Task 6.3 will be co-ordinated by Partner 2 (LMU), but all WP participants will be involved.

Task 6.4 (month 25-27)
Training of the research teams

6.4.1. Concepts and strategies to how to train and motivate teachers in the different settings will be developed.
6.4.2. Research members from all WP participants (HUA, LMU, UGent, UniZar, CMHI, MUV) will be trained on how to educate teachers on the implementation of the ToyBox programme. The trained researchers will be further responsible for educating the teachers, who will actually implement the intervention in the class and with the parents at a local level in WP7.

Task 6.4 will be co-ordinated by Partner 2 (LMU), but all WP participants will be involved.

Task 6.5. (month 26-31)
Support of the intervention via the website

Internet material will be developed and put online in the languages of the participating countries to support the programme. This will also include a brief guide on the intervention, the actual ToyBox material and Frequently Asked Questions (FAQs). Initially, only teachers participating in the

programme will have access to them, so as to avoid contamination of the control group. After the end of the intervention, the material will be accessible to the public and additional activities, attractive to children, will be available.

Task 6.5 will be co-ordinated by Partner 2 (LMU), but all WP participants will be involved.

Task 6.6. (month 30-46)
Revise the ToyBox material based on the input obtained of the intervention during the implementation phase
During the implementation of the ToyBox intervention all input received locally regarding issues not well understood or addressed by the material, missing or confusing information etc will be constantly monitored.
This input along with the process evaluation throughout the implementation of the intervention (WP7) will guide LMU to revise and further improve the material to accommodate additional local input on this joint European attempt. A report on any changes made to the ToyBox material will be made by country.

Task 6.6 will be co-ordinated by Partner 2 (LMU), but all WP participants will be involved.

Deliverables

<u>ToyBox Teacher's General Guide and ToyBox Classroom Activities Guide</u>
D6.1. Developed in English (Tasks 6.1 and 6.2) (month 21)
D6.2. Translated and adapted for use in six countries (Tasks 6.1 and 6.2) (month 23)

<u>ToyBox Parental Activities Guide</u>
D6.3. Developed in English (Task 6.3) (month 21)
D6.4. Translated and adapted for use in six countries (Task 6.3) (month 21)

D6.5. All material (Teacher's General Guide, Classroom Activities Guide and Parental Activities Guide) printed and CD-ROMs with presentations prepared and ready for use for all six countries (Tasks 6.1, 6.2, 6.3) (month 24)
D6.6. Training modules for teachers in participating countries developed (Task 6.4) (month 27)
D6.7. The total set of ToyBox printed material and toys delivered in all participating countries (Tasks 6.1-6.3) (month 27)
D6.8. Internet material developed and put online to support the programme, in the languages of the participating countries. (Task 6.5) (month 31)
D6.9. Final versions of ToyBox material in all languages (incl. English) and ToyBox with material for classroom activities (Tasks 6.1-6.6) and report on any changes made to the ToyBox material by country (Task 6.6) (month 46)

**Work package number**	7		**Start date or starting event:**		20	
**Work package title**	Implementation and evaluation of a cluster randomized intervention					
**Activity Type**	RTD					
**Participant number**	**1**	2	3	5	9	10
**Person-months per beneficiary:**	**36**	22	22	22	20,7	22

Objectives

7.1 Development and implementation of an intervention procedure

7.2 Evaluation of the process of the intervention to ensure that the intervention is implemented as intended

7.3 Evaluation of the effectiveness of the intervention in terms of impact and outcome changes.

Description of work

A cluster randomized intervention will be implemented in six different European countries, namely Greece, Spain, Belgium, Germany, Poland, Bulgaria. Comprehensibility, readability, relevance, credibility, attractiveness of the intervention will be tested, so that the intervention components can be adapted if necessary. Furthermore, it will be tested whether the intervention induces changes in important energy balance behaviours and their determinants.

The target of this intervention will be children (4-6 year olds), their parents and the teachers. The intervention will be implemented in the pre-primary education setting by teachers and will be tested in a pre-test post-test design including an intervention and a control group. The pre- and post intervention period is estimated to last two months and in order to avoid seasonality effect, the pre- and post-examination will take place at the same time of the year leaving 9-10 months in between for implementation. Only 4-5 years old (with a parental signed consent form) will be examined at baseline and only these children will be re-examined at the follow up, about a year later.

From each country 20 pre-primary education schools with 2 classes each (20 children per class on average) will participate in the evaluation study, resulting in about 4800 participating children on a European level. Power calculations based on previous studies of school-based interventions indicates that this sample size is sufficient to detect changes in energy balance behaviours and their determinants.

The schools will be assigned randomly as intervention or controls in a 2:1 ratio. The control group will not receive any intervention materials but will continue to follow the standard curriculum. Schools will be the level of randomisation in order to prevent contamination of intervention activities. This WP will also be responsible for the coordination of data entry of pre- and post-intervention evaluation.

Task 7.1 (month 23-28)

Train teachers implementing the intervention

7.1.1. Develop a protocol for the teachers' training.

7.1.2. Coordinate the training of the teachers who will implement the intervention using the researchers trained in WP6.

Task 7.1 will be co-ordinated by Partner 1 (HUA), but all WP participants will be involved.

Task 7.2 (month 22-40)

Conduct the cluster randomized intervention

7.2.1. Write a study protocol for ensuring standardized school and parent recruitment, questionnaire administration, data collection, data entry and data management

7.2.2. Obtain approvals from Ministry or local authorities to contact and enter the schools.

7.2.3. Recruit randomly 20 schools per participating country for inclusion in the evaluation study; explain the purpose of the study and the content of the intervention. Classes will be randomly selected within predefined regions to maximise representativeness of the overall

population but also to ensure high risk groups (e.g. families with certain socio-demographic characteristics (as identified in WP2) will be included.

7.2.4. Assign randomly these 20 schools as intervention or controls in a 2:1 ratio.

7.2.5. Implement the intervention at the participating schools assigned to the intervention group in six countries (Greece, Spain, Belgium, Germany, Poland, Bulgaria)

Task 7.2 will be co-ordinated by Partner 1 (HUA), but all WP participants will be involved.

Task 7.3 (month 21-45)
Process evaluation

This will be assessed by questionnaires directed to parents, teachers and school boards in the intervention schools to assess easiness of implementation. It will try to assess whether the intervention was implemented in the way it was intended, if it was adjusted/ changed along the way, what were the experiences/responses of those who were implementing the intervention and how the collaboration functioned. Partnership working between the relevant agencies will be specifically monitored and mapped during the course of this project. The results of WP5 will inform this Task of specific areas that should be addressed during the process evaluation.

7.3.1. Identify appropriate evaluating tools (i.e. questionnaires to assess process measures, i.e. intelligibility, credibility, readability, relevance, attractiveness of the intervention tools) to be used by parents, teachers and school boards

7.3.2. Translate and back-translate all process measures tools in the six corresponding languages.

7.3.3. Request teachers to complete logbooks and questionnaires assessing process measures (i.e. context, reach, dose delivered, dose received, fidelity, implementation and recruitment) during the 9 months that the intervention is implemented at the schools.

7.3.4. Conduct a process evaluation at a country and European level.

7.3.5. Development of databases for data entry.

7.3.6. Coordination of data entry.

7.3.7. Clean all data according to standardised protocols;

7.3.8. Executing the analyses and write report on the findings.

7.3.9. Report and transfer data and results to WP1 for storage and overall data management.

Task 7.3 will be co-ordinated by Partner 1 (HUA), but all WP participants will be involved.

Task 7.4 (month 23-46)
Impact and outcome evaluation

We will evaluate whether the intervention is having the intended impact on the behavioural and environmental factors it is aimed at, and adjust accordingly. The impact evaluation will be based on pre- and post-changes on children's diet, sedentary and physical activity behaviours, predisposing and enabling factors related to home and school social and physical environment, parental eating and physical activity behaviours and parental self reported BMI. The outcome evaluation will be based on pre- and post-changes on children's weight, BMI and waist circumference as well as overweight and obesity prevalence. All the tools for the impact and outcome evaluation will be developed in WP8.

Both impact and outcome evaluation will be used to assess programme’s effectiveness and add to the evidence about what works (and doesn't work) and help in supporting decision making in Public Health Policy.

7.4.1. Obtain informed consent forms from the parents of participating children.

7.4.2. Tools developed in WP8 will be implemented in the pre- and post-intervention evaluation.

7.4.3. Coordination of data entry (databases will be provided by WP8).

7.4.4. Clean all data according to standardised protocols;

7.4.5. Executing the analyses and write report on the findings.

7.4.6. Report and transfer data and results to WP1 for storage and overall data management.

Task 7.4 will be co-ordinated by Partner 1 (HUA), but all WP participants will be involved.

**Deliverables**	
D7.1.	A protocol for the implementation and evaluation of the ToyBox intervention (Task 7.2) (month 25
D7.2.	Process evaluation tools developed (Task 7.3) (month 26)
D7.3.	Report on the process, impact and outcome evaluation at a country and European scale (Task 7.3) (month 48)
D7.4.	Report on mediators and moderators affecting the magnitude of the intervention at a local and European level to support decision making in Public Health Policy (Tasks 7.3 and 7.4) (month 50)

**Work package number**	8	**Start date or starting event:**			1		
**Work package title**	Development, validation and training for the outcome and impact assessment tools						
**Activity Type**	RTD						
**Participant number**	**5**	1	2	4	3	9	10
**Person-months per beneficiary:**	**18**	1	1	1	2	2,8	1

Objectives

8.1 Development of assessment tools and procedures for the outcome and impact evaluation in WP7.

8.2 Testing the validity of developed tools and procedures

8.3 Training of the researchers participating in outcome and impact evaluation in WP7.

Description of work

As part of the evaluation framework (WP7), data will be collected on height, weight, waist circumference (end outcomes) and most important energy balance related behaviours (eating, sedentary and physical activity behaviours) and their determinants as identified in WP2 and WP3 respectively. This WP will be responsible for the development, validity testing and training in use of tools to assess all above mentioned variables.

All the partners included in this WP will obtain the data using the tools described below. Translation of the questionnaires into 6 local languages (Spanish, German, Greek, Dutch, Polish and Bulgarian) will be performed by all the partners included in this WP.

Task 8.1. (month 1-8)

Review of the literature on tools assessing food intake and physical (in)activity to assess the impact of childhood obesity prevention strategies in Europe

Conduct a literature search on tools assessing food intake and physical (in)activity in childhood and identify those most appropriate to use in intervention studies, sensitive enough to detect pre- and post-intervention differences.

Task 8.2. (month 8-26)

Children's food intake and eating behaviours assessment

A short food frequency questionnaire (FFQ) will be used to assess pre- and post-intervention consumption of specific food groups. This FFQ will be based on previously developed questionnaires and will be adapted to include important food groups or food items identified in WP2. Liaison will be created with ongoing FP6 projects, in which dietary assessment in children is an explicit research topic and from which the ToyBox project could benefit- IDEFICS (http://www.ideficsstudy.eu) and EFCOVAL (http://www.efcoval.org). Furthermore, based on the eating behaviours identified in WP2, some additional questions will be added to form the eating behaviours questionnaire. Both the FFQ and the eating behaviours questionnaire will be completed by parents/guardians. A validity test will be executed prior to the implementation in a small cohort in each country.

Task 8.3 will be co-ordinated by Partner 5 (UniZar), but all WP participants will be involved.

Task. 8.3. (month 8-28)

Children's physical activity and sedentary behaviours assessment

8.3.1. Based on the findings of literature and research experience of the ToyBox consortium, proxy questionnaires (completed by parents) do not provide reliable data on young children's (<6 year olds) physical activity levels. A good tool to obtain accurate physical activity level estimates at this young age is accelerometry; however high cost, time constraints, need for extensive training of staff and for downloading the data regularly in a pc, does not make this method an easily applicable one, in large scale studies.

For this reason, physical activity will be measured with the use of pedometers, since it has been shown that daily step counts in preschool children give valid information on physical activity levels (compared to accelerometry data) [132]. Step counts will be assessed using the Omron Walking Style Pro Pedometer which is most technologically advanced and can give information on physical activity during the different day sections
.
Before the pedometers are handed out, they will all be reset to zero and checked for proper fit and function. A pedometer will be fastened to the waistband of each pupil's pants or skirt. Children will be instructed to wear the pedometer on the left hip, in line with the midpoint of the left knee.
A researcher in collaboration with the teacher and parents will ensure that steps are registered twice a day, once when the child leaves the school setting and once before going to bed. The first registry will provide data on physical activity performed in school setting and the second on physical activity performed at home or at leisure time. During weekend days, only one registry will be obtained, before children go to bed. Measurements will be obtained for both weekdays and weekend days for all children. All teachers will be informed of the procedure and proper instrument use. A parents' informational letter will include instructions for proper instrument use. Parents will be asked to have their children wear the instruments for as long as possible during all waking hours, removing them only for water-based activities and sleeping. A diary will be completed by the parents regarding time periods and occasions that the pedometer was removed (e.g. swimming).

8.3.2. Sedentary behaviours (e.g. watching television, using a computer, playing electronic and board games, reading or painting) and organized activities (i.e. extra-curricular physical activity) identified in WP2 will be assessed by using a questionnaire. In the questionnaire the parents will report the physical activity behaviour of their child based on specific behaviours such as: walking or cycling for transportation together with their child, walking or cycling for leisure together with their child (child also walks or cycles him/her self), membership of sports club (swimming, soccer, gymnastics, ...), active play with their child outdoors, ... The outcome will be reported in minutes of activity per day.
A liaison will be created with an ongoing FP6 project, in which sedentary behaviours in pre-pubertal children is an explicit research topic and from which the new proposed project could benefit – IDEFICS (http://www.ideficsstudy.eu). Validity of the sedentary behaviours questionnaire will be checked using a diary that the parents will fill in during one week. Results from the questionnaire will be compared with those obtained with the diary.

Task 8.3 will be co-ordinated by Partner 5 (UniZar), but all WP participants will be involved.

Task 8.4. (month 8-28)
Anthropometric measurements

For anthropometric measurements children will be barefooted and in their underwear or bathing suit. The following measures will be taken: Body weight (kg): using an electronic scale (Seca), precision 100 g, range 0–200 kg. Height (cm): using a precision stadiometer (Seca), precision 0,2 cm, range 70–200 cm. The subjects stands straight in an upright position, the feet together, knees straight, heels, buttocks and back must touch the back part of the stadiometer. The head must be in a position in order the Frankfort line is horizontal. The arms have to hang relaxed on the side of the body, the inner part of the hand faced to the thigh. The mobile, horizontal part of the stadiometer must touch the head of the subject, with a light pressure on the hair. Waist circumference will be measured with an unelastic tape, precision 0.1 cm, range 0–150 cm, the subject in a standing position, the tape is applied horizontally midway between the lowest rib margin and the iliac crest about the level of the umbilicus, at the end of gentle expiration. Prior to the implementation of the first survey, all the measurements will be tested for intra- and inter-observer reliability in a small children's sample.

Task 8.4 will be co-ordinated by Partner 5 (UniZar), but all WP participants will be involved.

Task.8.5. (month 11-26)
Assessment of social and physical environmental determinants
This Task will develop the tools for the assessment of changes in the social and physical environmental determinants (identified in WP3) of the energy balance related behaviours under study (identified in WP2). The tools will be based on previous instruments and on the major theories of behaviour change (Theory of Planned Behaviour, ANGELO model).
Psychosocial correlates may include: knowledge, beliefs, attitudes, perceptions, social variables, practices and rules, self-efficacy, perceived benefits and perceived barriers, among potential variables detected in WP3. Environmental correlates will include physical environments, economical factors, policies and regulations related to the target behaviours at home, at school and in the neighbourhood of the child, among potential other environmental variables detected in WPs 3.
These questionnaires will be completed by the parents and the teachers.

8.5.1. Develop a questionnaire to be completed by parents. It will include a) psychosocial correlates for themselves, b) psychosocial correlates for their child and c) environmental correlates regarding home and neighbourhood.
8.5.2. Develop a questionnaire to be completed by teachers. It will include a) psychosocial correlates for themselves and b) environmental correlates regarding classroom and school setting.
8.5.3. All tools (Tasks 8.5.1-8.5.2) will be translated and back-translated in the languages of the countries conducting the intervention and will be used in the pre- and post- intervention evaluation measurements in WP7. In a preliminary phase, test-retest reliability for this questionnaire will be analysed.

Task 8.5 will be co-ordinated by Partner 5 (UniZar), but all WP participants will be involved.

Task 8.6. (month 16-26)
Training of staff involved in the measurements
A detailed protocol for planning, training and data collection on children's food intake and eating behaviours, physical activity and sedentary behaviours and anthropometry will be made. Similar procedures will be followed regarding social and physical environmental determinants.
A training workshop will be organized previous to the pre- and post-intervention measurements (WP7) for the training of researchers in all assessment tools and procedures. Field workers will be trained in all the procedures described in and a quality control protocol will be set up in each country.

Task 8.6 will be co-ordinated by Partner 5 (UniZar), but all WP participants will be involved.

Task 8.7. (month 8-28)
Quality control
Centralized supervision of translations of questionnaires, protocols, standard operating procedures will be performed by UniZar. Verification of correctness by specific procedures, like cross-checking, back-translations, etc. will also be performed. A protocol for the collection of study data from the participating centres with an agreed format and ensuring security and confidentiality of the data will be developed. Databases for data entry will be developed for all tools described in Tasks 8.2 to 8.5 and a manual of data entry procedures will be developed.

Task 8.7 will be co-ordinated by Partner 5 (UniZar), but all WP participants will be involved.

Deliverables

D8.1	A report on the review of the literature on tools assessing food intake and physical (in)activity to assess the impact of childhood obesity prevention strategies in Europe (Task 8.1) (month 10)
D8.2	Protocol(s) for planning, training and data collection will be made on children's food intake and eating behaviours, physical activity and sedentary behaviours and anthropometry as well as social and physical environmental determinants. (Tasks 8.2-8.5) (month 26
D8.3	A protocol for the collection of study data from the participating centres with an agreed format and ensuring security and confidentiality of the data will be developed (Task 8.6) (month 25)
D8.4	Databases for data entry and manual for data entry procedures will be developed for all tools described in Tasks 8.2 to 8.5 (Task 8.6) (month 28)
D8.5	A scientific paper submitted in a peer reviewed journal on the review of the literature on tools assessing food intake and physical (in)activity to assess the impact of childhood obesity prevention strategies in Europe (Task 8.1) (month 16)
D8.6	Report on the validity and harmonization process of the tools regarding food intake and eating behaviours, physical activity and sedentary behaviours and anthropometry (Tasks 8.2-8.7) (month 36)
D8.7	A scientific paper submitted in a peer reviewed journal on the development and validity testing of the assessment tools (Tasks 8.2-8.7) (month 36)

Work package number	9	Start date or starting event:	20			
Work package title	Evaluation of the cost-effectiveness of the intervention					
Activity Type	RTD					
Participant number	**3**	1	2	5	9	10	13
Person-months per beneficiary:	**12**	1	1	1	0,5	1	1

Objectives

9.1 Measurement of the costs associated with preparing and implementing the intervention programme as described in WPs 3, 6 and 7;

9.2 Estimation of the long term costs, effects and cost-effectiveness of the intervention programme.

Description of work

The investment associated with the programme will be measured by including data collection related to setting up the programme as explained in WP3 and 6. Also, during the programme implementation, data will be collected regarding resource use (e.g. teacher time, parental time …) (WP7). Then, a health economic model will be developed to calculate the long term impact of the programme in terms of costs and health effects. This will result in a cost-effectiveness analysis expressed in cost per QALY or cost per avoided DALY.

Task 9.1 (month 24-25; 36-42)
Cost assessment
Both the preparation and implementation of the intervention requires the use of resources (staff time, material, etc). Two types of questionnaires will be developed for this purpose:

9.1.1. A questionnaire related to the preparation of the programme will be developed, whereby the project leaders and their staff will be the respondents (see WP 6).

9.1.2. A questionnaire will be developed to be integrated in the process evaluation questionnaires (see WP 7). This may include teacher time, parent time, etc.

9.1.3. The health economic researchers will collect data regarding the unit costs for each type of resource in the involved countries (cost of scientific staff time, cost of teacher time, printing material, etc)

9.1.4. The observed resource use is then afterwards multiplied with the unit cost of each type of resource. E.g. the number of hours of teacher time multiplied with the cost per hour. All unit costs are considered from a societal perspective.

Task 9.2 (**month 36-48)**
Cost- Effectiveness model
Based on the outcomes of the intervention (impact on weight, physical activity, food group intake, etc), as measured in WP7, predictions will be made related to the adult age of the participating children. The predictions are based on published relationships between childhood and adult parameters (e.g. the relation between childhood obesity and adult obesity; the relation between childhood fruit consumption and adulthood fruit consumption) [127,133-137]. These are then in turn related to avoided cardiovascular disease and avoided cancers (colorectal, breast).
The model will be a Markov model, consisting of different health or disease states and predicting the evolution of a hypothetical cohort of children undergoing the programme vs. a control cohort. The model consists of the following states: healthy, heart disease, cerebrovascular disease, diabetes, breast cancer, colorectal cancer, and death (see also [127,138]). Throughout the model, each member of the cohort can undergo transitions between states (e.g. from healthy to heart disease to cerebrovascular disease to death). The time horizon of the model is at least 50 years in order to capture all relevant future costs and outcomes. The perspective of analysis is societal, meaning that the impact of healthier life is not only assessed from a health care payer perspective but also from a societal perspective, i.e. including the economic impact of work productivity. Data

inputs from the model will be obtained partly from task 9.1., partly from validated published models, and published epidemiological and cost of disease data.

Deliverables

D9.1. Interim report with the cost of the intervention (month 43)

D9.2. Projection model in MS Excel (month 46)

D9.3. A full report containing the health economic model description, data inputs and results (month 48)

D9.4. A brief report presenting key findings regarding long term costs, effects and cost-effectiveness of the programme to inform policy makers (month 48)

**Work package number**	10		**Start date or starting event:**		6	
**Work package title**	Dissemination					
**Activity Type**	OTHER					
**Participant number**	11	1	3	6	12		
**Person-months per beneficiary:**	12	4	4	5	4		

Objectives

10.1. Listing of a network of all research efforts at the European level on obesity and overweight prevention, nutrition and physical activity, and health inequalities in these issues across Europe, including information on the expertise and projects that are currently underway or being developed in these areas.

10.2. Dissemination of the publicly available deliverables and results from the project to the main stake holders: schoolchildren, parents, school staff, policy makers, health professionals and scientists

10.3. Inform and generate interaction between relevant stakeholders and public policy-makers working on the field of obesity, physical activity nutrition and health inequalities.

10.4. Serve as review board for research findings generated and serve as an advisory and consultancy point for the European Commission in relation to evidence-based policies.

Description of work

Effective and successful interventions at school setting can only take place when there is sufficient public and stakeholder understanding and support, along with the necessary backing from policy-makers and politicians. WP10 takes the work of the ToyBox project and disseminates this to relevant stakeholders in order to directly inform policy and practice regarding obesity prevention efforts in young children. Because of the direct applicability of the project's results dissemination to the scientific community and to policy makers and health promotion professionals is equally important. To give this task the necessary attention, the ToyBox project has dedicated a specific WP to dissemination.

This WP will use all information available from WP 2-9, and especially information from WP5 (on contextual factors and existing infrastructures in schools) and WP 9 (on cost-effectiveness of the programme), in order to promote the ToyBox intervention and will further promote timely dissemination of the results of the Work Packages during the course of the project.

Task 10.1 (month 6-50) (IASO)

Prepare and maintain a list of programmes on obesity research, with a special focus on child obesity

To make a list and maintain and develop the IASO database of current and up-coming projects and programmes on obesity research, with a special emphasis on child obesity, and to create a network of groups (a network of networks) engaged in this research at European level. This builds on the current work undertaken in the EU-funded HOPE and EURO-PREVOB projects, and the IASO and EASO member associations.

Task 10.2 (month 6-50)

Promote dissemination of all public deliverables of ToyBox during the course of the project

10.2.1. To promote the ToyBox project and results in major international conferences and congresses;

10.2.2. To co-ordinate a series of press releases timed to coincide with ToyBox presentations at conferences and publication of ToyBox reports and scientific papers.

10.2.3. To produce Toy Box electronic newsletters (2-3 per year) .

10.2.4. To organise network–of-networks meetings aimed at reviewing the work in progress and acting as a vehicle for disseminating evidence-based policy recommendations. These will

be scheduled between month 24 and month 36 of the project.

Task 10.2 will be co-ordinated by Partner 11 (IASO), but all WP participants will be involved.

Task 10.3 (month 36-50) (IASO, HiOA, UGent, NIGZ, HUA)
Inform relevant stakeholders and public policy-makers

10.3.1. To construct cross-cutting matrices of scientific knowledge in relation to specific policies and interventions proven to be effective for tackling overweight and obesity, especially among children.
10.3.2. Prepare popular summaries of the results of the ToyBox WPs for dissemination to public media;
10.3.3. Present findings of the ToyBox project to the DG Sanco Platform on Diet, Physical Activity and Health and other interested EC bodies;
10.3.4. Publish results of the ToyBox project in the separate sections on the project's website, including scientific summaries for professionals and more popular summaries on the public section of the website in all relevant languages.
10.3.5. To provide syntheses of the evidence generated in this project and relevant to policy-makers at the European, national and local level.
10.3.6. To inform young researchers and field workers active in the field of health promotion and policy making through the existing Young Public Health Nutritionists Network. (Members of UGent and **HiOA**are involved in this initiative and focus specifically on young and less experienced people)
10.3.7. To inform Educational Councils and Teachers organizations through the existing Schools for Health in Europe (SHE) Network. NIGZ is the coordinator of this Network.
10.3.8. To link with the recently formed World Public Health Nutritionist Association, which will be launched in 2009 at the IUNS conference in Bangkok, to also provide a global link.
10.3.9. To link with existing training programme networks for health care staff, for public health nutritionists and public health workers in Europe.
10.3.10. To link with the Developmental Origins research groups in order to provide a life course perspective and bring this into schools (early information to prospective mothers etc)

Deliverables

D10.1. A listing of the Network of Networks (month 6);
D10.2. One Network of networks meetings (month 30-36, month 42-50);
D10.3. Matrices of key scientific findings against specific policies and interventions according to their contexts (e.g., education, legislation) (month 50);
D10.4. A list with at least twenty (20) items that appeared in independent public media (prior to month 50);
D10.5. A list with at least six (6) scientific papers submitted in a peer reviewed journals or conference presentations (prior to month 50);
D10.6. Policy recommendation fact sheets for EU, national and local policy-makers (month 50).

B 1.3.6 Efforts for the full duration of the project

Include here two Project Effort Forms which show the person-months per beneficiary associated with each activity for the full duration of the project. Templates are given in Appendix 6.

The first form to be used here is the same as the one used in the proposal; the second form is an extended version with a breakdown to 'activity type'- level per participant. This is required for the correct calculation of the requested EC contribution, as different reimbursement rates apply for the different activity types.

Project Effort Form 1 - Indicative efforts per beneficiary per WP

Participant no./short name	WP1	WP2	WP3	WP4	WP5	WP6	WP7	WP8	WP9	WP10	Total person months
1/HUA	**26**	1	3	1	1	7	**36**	1	1	4	81
2/LMUM		1	3	1	1	**32**	22	1	1		62
3/UGent		4	**18**	2	9	9	22	2	**12**	4	82
4/VUmc		**18**	1	1		2		1			23
5/UniZar		1	3	1	1	7	22	**18**	1		53
6/**HiOA**					10					5	15
7/UDUR				**10**		2					12
8/IFP				3		12					15
9/CMHI		1	3	1	1	7	20.7	2.8	0.5		37
10/MUV		1	3	1	1	7	22	1	1		37
11/IASO										**12**	12
12/NIGZ					**18**					4	22
13/AOK- Verlag						19			1		20
14/RoU				2.5		2					4.5
15/ULU				2.5							2.5
Total	26	27	34	25	42	106	144.7	26.8	17.5	29	**478**

Project Effort Form 2 - indicative efforts per activity type per beneficiary

Activity Type	HUA	LMU	UGent	VUmc	UniZar	**HiOA**	UDUR	IFP	CMHI	MUV	IASO	NIGZ	AOK-Verlag	RoU	ULU	TOTAL ACTIVITIES
RTD/Innovation activities																
WP2	1	1	4	18	1				1	1						27
WP3	3	3	18	1	3				3	3						34
WP4	1	1	2	1	1		10	3	1	1				2.5	2.5	25
WP5	1	1	9		1	10			1	1		18				42
WP6	7	32	9	2	7		2	12	7	7			19	2		106
WP7	36	22	22		22				20.7	22						146
WP8	1	1	2	1	18				2.8	1						25
WP9	1	1	12		1				0.5	1			1			18
Total 'research'	51	62	78	23	53	10	12	15	37	37	0	18	20	7		423
Demonstration activities																
Total 'demonstration'																
Consortium management activities																
wp1	26															26
Total ' management'	26															26
Other activities																
WP10	4		4			5					12	4				29
Total 'other'	4		4			5					12	4				29
TOTAL BENEFICIARIES	81	62	82	23	53	15	12	15	37	37	12	22	20	4.5	2.5	478

Note: This is a new table, with a breakdown of efforts per beneficiary to activity type level, which was not requested in the proposal

B 1.3.7 List of milestones and planning of reviews

List and schedule of milestones					
**Milestone no**	**Milestone name**	**WP no's**	**Lead beneficiary**	**Delivery date from Annex I**	**Comments/ Means of verification**
M1	Kick-off Steering Committee meeting	1	1	2	Minutes of the meeting
M2	Opening of the web-portal	1	1	6	Functioning website
M3	Completion of systematic literature review on behavioural models	4	7	6	Report available
M4	Completion of narrative literature review on educational strategies	4	7	6	Report available
M5	Report on existing policies and legislations at pre-primary education settings	5	12	6	Report available
M6	Setting up of network	10	11	6	Network available
M7	Completion of systematic literature review on identification of energy balance-related behaviours	2	4	8	Report available
M8	Completion of secondary data analyses on importance of energy balance behaviours	2	4	8	Report available
M9	Report on health promotion activities and legislations at pre-primary education settings	5	12	8	Report available
M10	Report on contextual factors at pre-primary education settings	5	12	8	Report available
M11	Report on good examples of Policies and health promotion activities at pre-primary education settings	5	12	9	Report available
M12	Web-portal network of networks Communication structure	10	11	10	Functioning web-portal
M14	Completion of focus group interviews and analyses	3	3	12	Recordings and report
M15	Completion of survey instruments for evaluation study	8	5	26	Survey instruments combined and pretested
M16	Intervention materials ready for the intervention study and delivered to participating countries	6	2	27	Materials and handbook available
M17	Meeting for dissemination of the results	10	11	30-50	Presentations and minutes

Milestone no	Milestone name	WP no's	Lead beneficiary	Delivery date from Annex I	Comments/ Means of verification
M18	Final matrices of scientific findings against policies and interventions	10	11	50	Report available
M20	Completion of evaluation of the intervention	7	1	46	Data cleaned and available
M21	Synthesis, scenarios and interaction with DG SANCO, EU Platform for Action and successor, policy makers and stakeholders	10	11	36-50	Report available
M23	Full report on health economic model description, data inputs and results	9	3	48	Report available

**Tentative schedule of project reviews**			
**Review no.**	**Tentative timing, i.e. after month X = end of a reporting period**	***planned venue of review***	***Comments , if any***
**1**	After project month: 24	**To be discussed**	

B2. Implementation

B 2.1 Management structure and procedures

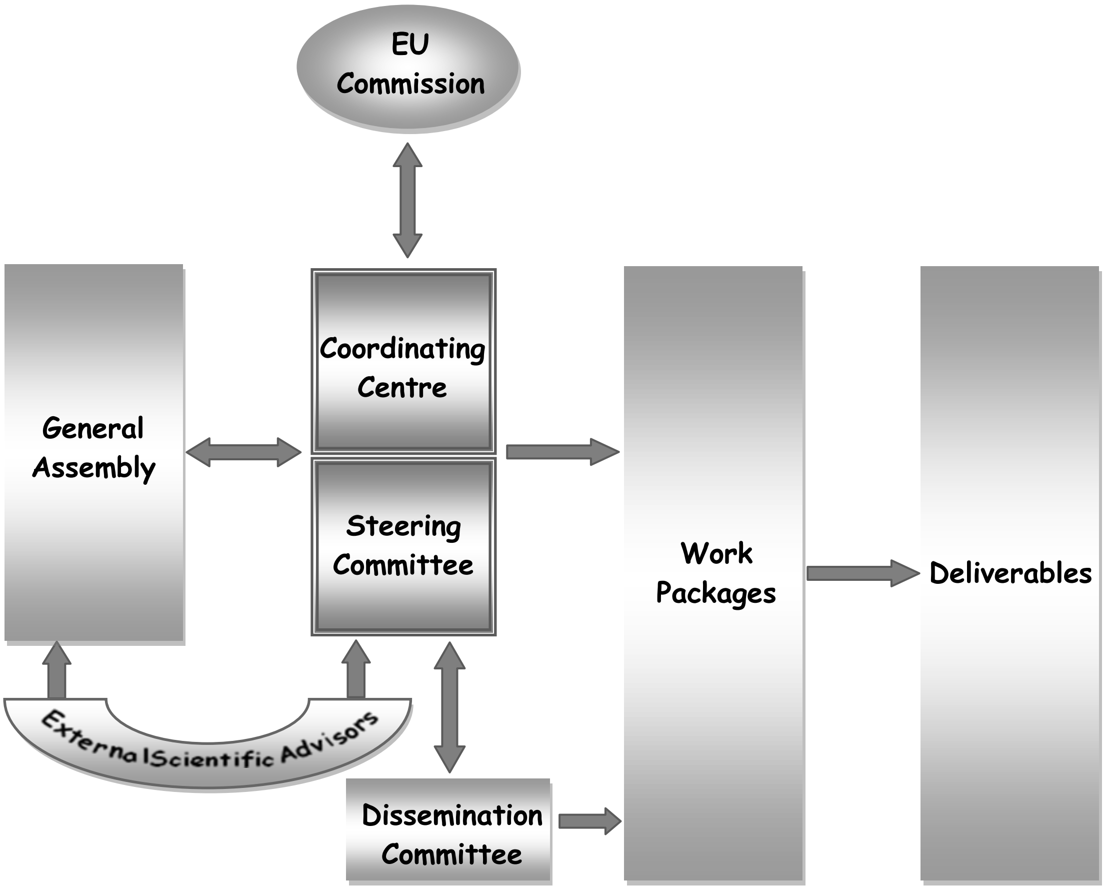

B 2.1.1Project management structure

A centralized management structure will be set up, whereby the Harokopio University (HUA) in Greece as coordinating centre and provider of the chair of the Steering Committee will be responsible for the overall and financial management of the project (see project management chart above). Therefore WPs will report directly to the HUA, which will be responsible for dealing with administrative and management tasks envisaged in project implementation. HUA will also be the central body responsible for communication with the European Commission.

Communication among participants and WP leaders will be strongly supported by periodic meetings, a specially developed project web-portal and web based conference calls. Furthermore, dissemination of the findings of the project among a wide array of stakeholders, (e.g. national hear and obesity associations, schools, policymakers) and the general public will be further facilitated through scientific conferences as well as public presentations.

B 2.1.2General Assembly

The General Assembly will be composed of one duly authorised representative of each partner and will be chaired by the Coordinator. The agenda of the meetings will be proposed by the coordinator, and agree upon with the Steering committee; the coordinator will be responsible for preparing the minutes of each assembly meeting.

The General Assembly will be the decision making body on issues that have to do with matters not described in this project but of equal importance for the optimal execution of work and the scientific impact of this work. Such matters have to do with potential PhD Theses, publications beyond those already committed as deliverables. This will ensure equal access and share of voice for all ToyBox partners on such academic opportunities that ToyBox aims to provide to new scientists and researchers from all participating countries.

B 2.1.3Steering committee

The steering committee (SC) will be the main decision making body of the ToyBox project. Yannis Manios of the project coordinating centre HUA will act as chair of the SC and as such will be responsible for central and financial management. Yannis Manios has executed several multicentre and multi-national studies as a coordinator mainly in East Mediterranean region and Middle East. He has also participated as principal investigator in the FP-5 funded Phytohealth project (Improving health through dietary phytoestrogens: A Pan-European Network on consumers' Issues and opportunities for producers, contract number QLRT-2001-02453), the FP-6 funded HELENA project (Healthy Lifestyle in Europe by Nutrition in Adolescence, contract number FOOD-CT-007034); he has also participated as co-principal investigator in Zincage (Nutritional Zinc, Oxidative stress and Immunosenescence: Biochemical, Genetic and lifestyle Implications for Healthy Ageing, contract number STREP-506850). As such he has ample experience with European Commission (EC)-funded projects. The SC will comprise all work package (WP) leaders. All WP leaders and most partners are principal investigators, or otherwise involved in different EC-funded projects and thus have experience with management of projects and work packages such as those proposed here. Furthermore, the SC includes expertise in all necessary competences for successful management of the project: project coordination and financial management (Yannis Manios), systematic reviews (Summerbell, Brug, Lobstein, Chin A Paw, te Velde), data-management and analyses of multilevel, international data sets (Brug, Buijs, Moreno, De Bourdeaudhuij, Chin A Paw), school-based intervention studies (Manios, Brug, Chin A Paw, De Bourdeaudhuij, Maes, Moreno, Iotova) translation of research results into policy recommendations and dissemination towards key stakeholders, including policy makers and consumers (Lobstein, Buijs, De Henauw, Yngve). In addition, one of the members of the SC will chair a dissemination committee, with representatives of the International Association for the Study of Obesity (IASO), the NIGZ- WHO collaborating centre for School Health Promotion and Høgskolen i Oslo og Akershus (HiOA) ensures that the project management will not only focus on timely realization of all deliverables, but also on anticipation of implementation and dissemination of these deliverables.

The central mission of the SC will be to guide the overall activities and progress of the project. The main tasks of the SC will be:

(1) Agree on specific actions to be taken in order to ensure project success in terms of timely deliverables and milestones this may include:
  a. Adaptation of the planning if necessary
  b. Reallocation of the budget if necessary;
(2) Ensure full collaboration and coordination between WPs and put the interdependency between the WPs to its full use;
(3) Provide additional contact points for information on the overall aims and results of the project.
(4) Discuss and advise the co-ordinator on any legal aspects (intellectual property, regulations, safety & ethical considerations) that may arise.
(5) Monitoring the quality of each WP and thus ensure that project results are of the highest standards;
(6) Provide guidance and monitoring for timely preparation of progress reports for the European Commission;
(7) Develop mechanisms to establish dialog and regular contacts with relevant Commission services, including meetings, briefings and informal contacts between the consortium and relevant Commission services.

B 2.1.4Coordinating centre

The HUA will be responsible for the daily management of the project, including financial management. The coordination centre will consist of a management team and a management support team. The management team will include the project coordinator (Yannis Manios) and the project manager (to be appointed). The management support team consists of people from the secretary and financial department. The central responsibility of the coordination centre will involve the following tasks:

(1) monitor and evaluate overall progress and timely delivery of the deliverables;
(2) set up a structure for communication
    a. arrange monthly conference calls
    b. arrange and facilitate live meetings with WP leaders and invited WP participants in collaboration with partners
    c. develop a functional website, suitable for storing and exchanging documents;
(3) store all data and results retrieved from all WPs;
(4) the financial management of the entire project;
(5) communicate and report to the European Commission;
(6) follow-up the progress of each work package and objectives of the project as a whole.

HUA will thus be responsible for the day-to-day management and will play a central role in quality control and project reporting. These specific responsibilities will include: progress monitoring, financial management, control and reporting, organizing plenary meetings, coordination of preparation of annual and final reports and liaising to the European Commission. Financial and administrative support staff will assist the co-ordinating team at HUA. Besides regular bilateral contacts and the regular meetings, the HUA chair will hold periodically conferences calls with WP leaders and other project members. The HUA will also have special responsibility for the early development and maintenance of the ToyBox website, which will play a role in the project with respect to, communication within the consortium and to external stakeholders, data management, dissemination of project results and publications.

B 2.1.5Dissemination committee

This committee consists of partners from IASO, NIGZ (the Netherlands Institute for Health Promotion and Disease Prevention) and **HiOA**, who all participate in WP10 devoted to the dissemination of the findings of the project. IASO is the main independent coordinating and advocacy organisation for obesity prevention research and action, and is an important expertise and advice partner for WHO and other international health organisations. NIGZ has been actively involved in the European Network for Health Promoting Schools since 1992 coordinated by the WHO Euro in Copenhagen. Since 2007 NIGZ runs the secretariat of the network which now has a new name: Schools for Health in Europe network, or SHE network. NIGZ runs the annual Go for health campaign for primary schools as an agenda-setting activity. Since 2008 NIGZ acts as a WHO collaborating centre for school health promotion. **HiOA**is represented by Agneta Yngve, who is Editor in Chief for the journal Public Health Nutrition and who has wide experience in advising policy makers and making public health recommendations and as such is well-suited to disseminate the ToyBox results to European policy makers responsible for obesity prevention in the general public.
Apart from actually disseminating the findings the committee members have a good overview of ongoing research in Europe and will ensure that the current project is complementary to other research projects. Their main responsibilities will be the dissemination of the findings to the scientific community, important stakeholders and to the general public.

B 2.1.6External scientific advisors

The consortium will also include external scientific advisors, who are leading experts in the field and not included in the consortium. These advisors will support the consortium with scientific advice and thus ensuring the high scientific standard of the project.
The role and duties of the *External Scientific Advisors* are:

(1) In case of a scientific problem, give expert advice.
(2) Transmit the results and tendencies related to the different aspects of ToyBox study not only at personal but also at institutional level.
(3) Participate in three meetings of ToyBox: at start, intermediate (24 months), and final meeting. The external scientific advisors will participate in coordination actions in these meetings

The following experts have agreed to act as Scientific Advisors for the ToyBox project:

- *Dianne Ward (US)*
  Professor and Director, Intervention and Policy Division, Department of Nutrition, University of North Carolina. Her research focuses on the prevention of obesity through multi-component school and community interventions that promote physical activity and healthy eating. Director of an NIH research project, Linking Child Care to Home and was recently funded by RWJ to develop a measure of the diet environment at child care. She led the team that developed a policy and environmental intervention for child care. NAP SACC, the Nutrition and Physical Activity Self-Assessment for Child Care, is a highly regarded program within the public health community with many states using some aspect of the program. In association with NAP SACC, a number of new measures to assess the child care healthy weight environment and what young children eat were developed in association with this work. Her recent research involves an internet-delivered obesity prevention effort with families of children ages 5-8 years.
- *John J. Reilly (UK)*
  Personal Professor in Paediatric Energy Metabolism, University of Glasgow
  His research is currently focused on: Childhood obesity interventions (randomised controlled trials aimed at prevention and treatment); aetiology of childhood obesity; methodology for measurement of physical activity in childhood; systematic reviews in childhood nutrition.
- *Boyd A. Swinburn (Australia)*
  Professor of Population Health, and Director, WHO Collaborating Center for Obesity Prevention and Related Research and Training, Deakin University
  His major research interest at Deakin University is now centred on obesity prevention particularly in children and adolescents. He has developed and supported a number of community-based demonstration projects in the Barwon-South West region of Victoria and these are linked to similar projects in Melbourne, Auckland, Fiji, and Tonga.

B 2.1.7 Work package leaders and participants

Each WP will be conducted by a task force, led by a senior researcher, experienced in multicenter research (WP-leader) with specific expertise on the work to be conducted in the WP. The WP leader will be responsible for the quality and the progress of the work carried out in the WP, and for the progress of reports.
Each of the WPs needs input from different consortium partners and exchange with other WPs, illustrating the integrated character of the ToyBox project. In case of the WPs 1, 6, 7, 8, 9 and 10, input from almost all partners will be requested, i.e. to ensure a coordinated conduction of the project, to realise the development of the intervention scheme, the implementation and evaluation of the intervention and to encourage regionally-tailored dissemination plans. WP-leaders of the

different WPs will be responsible for the timely supply of the deliverables. WP-leaders will report directly to the coordinator, and information on progress will be made available to other consortium members.
WP-leaders but also WP participants also play an important role in making existing data sets available and accessible. Since several of the partners have access to national or international data on child overweight and obesity and energy balance related behaviours, they will provide their input for the secondary analyses that will be conducted in WPs 2.

WP-participants are qualified researchers who will support each task force by e.g. preparing data specifications, performing statistical analyses and collecting and reviewing literature, supervised by the WP leaders.

Each *Work Package Leader* will manage the day-to-day co-ordination of their work packages (s) as follows:

(1) Ensuring appropriate collaboration between members of the consortium.
(2) Providing sufficient and appropriate information on progress to all other partners.
(3) Co-operating with the project coordinator to ensure that key milestones are achieved and material for reports for dissemination activities is supplied to deadline.
(4) Submission of a named person and a deputy who will undertake these responsibilities for the duration of the project.
(5) The structure and function for each study centre will be designed to facilitate the development of the assigned tasks to each partner.

General responsibility of *WP participants*
Depending on their expertise, consortium members and advisors will be responsible for:

(1) Providing access to national or international observational or intervention studies/data sets on overweight, energy balance-related behaviours, determinants of these behaviours;
(2) Likewise, they can act as intermediate to bring WP leaders in contact with leaders of relevant studies and bring together input from existing projects and interventions on prevention of childhood overweight and promoting favourable energy balance-related behaviours;
(3) Those participating in WPs 2-5 will be responsible for providing input for the development of all the modules of the school based family involved intervention (WP6);
(4) Those participating in WPs 2-5 will also need to provide data and input for the development and adaptation of the assessment tools (WP8) to be used in the impact and outcome evaluation;
(5) Those partners participating in WP10 are responsible for translating outcomes from the evaluation study conducted in WPs 7 and 9, taking into account the findings of WP5 in concrete adaptations in order to create an intervention that is ready to be implemented;
(6) Ensure project progress according the plans with timely completion of deliverables;
(7) Liaison between the project and relevant stakeholders and policy-makers at the national and regional level who can benefit from the project outcomes.

B 2.1.8Communication between consortium members

Communication will be based on three important channels:
(1) <u>Internet</u>
Internet tools will be crucial to enable ongoing, continuous communication and exchange. A project website will be developed with functionalities for document exchange, consortium and WP-specific email lists, web-based conferencing, and data exchange. The ToyBox project website will consist of three parts. The first part will be 'open access' and will be used for informing and communication with stakeholders outside the consortium, including policy makers, health promotion professionals, researchers, educational platforms and the general public. The second part will be username and password restricted to teachers of the schools participating in the intervention; teachers will be able to download and print material and activities from the site and have support during the implementation. The third part will be username and password restricted to the consortium members. This area will include relevant documents, such as administrative EU documents,

descriptions of all WPs, documentation of draft program progress, a contact list, meeting agendas and minutes, and web conferencing facilitation. This area will also be used for the management, storage and exchange of the project's research data. Experience with such project websites have and are being obtained through earlier/other EC funded projects where ToyBox consortium members participate in, such as the Pro Children project (www.prochildren.org), the HOPE project (www.hopeproject.eu), the Eurocadet project (www.EUROCADET.org), the HELENA study (www.helenastudy.eu), the Idefics study (www.ideficsstudy.eu), the ENERGY project (www.projectenergy.eu).

(2) Monthly conference (web) calls

These calls will be held to ensure structured progress monitoring and exchange. The main participants will be the WP-leaders/ SC members, but other project participants will be invited when relevant. At each of these monthly meetings progress of all WPs in the previous time period and work to be conducted in the next steps will be discussed and agree upon. However, if any matters occur that need to be addressed by the consortium, additional conference calls will be organised and held to ensure work flow;

(3) Seven periodic (every 6 months) 'live' meetings

These meetings will be held to intensively review past progress and agree on details of upcoming activities. Three of the meetings will be restricted to WP-leaders (Steering Committee) and the main support staff. During these meetings all WP representers will formally present their progress in PowerPoint presentations, results will be discussed and implications for work in progress within other WPs, policy recommendations and dissemination will be agreed upon. These meetings will also be used to prepare progress reports for the Commission. To the other 'live' meetings a broader group of participants will be invited for broader validation and feedback of interim results, and the final meeting will have a specific dissemination purpose. In these meetings, coordination actions will be undertaken, which will cover the exchange and dissemination of good practices, performance of studies and setting up expert groups. For these meetings, the External Scientific Advisors will be invited to participate and their costs (estimated to be about 1000-1500€ per person per meeting) will be covered by WP1 (category: other direct costs).

B 2.2 Beneficiaries

1. The **Harokopio University in Athens, Greece, (HUA)** consists of four departments; one of these departments is the ***Department of Nutrition and Dietetics***, which offers high standards graduate and postgraduate education covering all aspects of human nutrition and clinical nutrition science. Within this department, Dr Yannis Manios represents the research group of ***"Nutritional Assessment, Health Promotion and Counselling"***. This group has ample experience in studying the prevalence and interactions among behavioural, demographic and physiological indices leading to the development of obesity and chronic disease risk factors in children and adolescents; developing and implementing intervention programmes aiming to prevent these major health issues. The group also offers its services and expertise on consulting governmental and non-governmental agencies on issues related to the development and implementation of nutrition policies.

Main tasks in the Toy Box project

The HUA will be responsible for the general management (WP1), lead the cluster randomized intervention (WP7), participate in literature review and conducting secondary data analyses (WP2 and WP4), conducting focus groups in Greece (WP3), adapting the developed intervention scheme (WP6), participating in the development of the assessment tools (WP8) and disseminate the results of the project (WP10). They will also provide information at local level for WPs 5 and 9.

Previous experience relevant to those tasks

The group has been involved in various multicentre studies in infants and children funded by either the European Union or other entities. Some of the national studies are the "Cretan Health and Nutrition Education Programme", the GENESIS-study and the CHILDREN-study whereas they have also been involved as partners in the FP5-programme PHYTOHEALTH, FP6-programme HELENA. They are also involved in the FP6-programme HOPE, providing data for the secondary analyses. In addition, the group has been appointed by the Greek Ministry of Education to develop the educational material for "Dietary habits and Nutrition" and "Physical Activity and Health" which is currently used as the basis of the Health Education and Health Promotion activities in all primary schools in Greece.

Short profile of team members involved in the project

Yannis Manios (MS, MPhil, PhD); Assistant Professor in Nutritional Assessment, Health Promotion and Counselling. He will be the project coordinator. He has ample experience in coordinating and conducting epidemiological surveys and intervention programmes in infants, children and adults.

Demosthenes B. Panagiotakos (PhD,FESC);Associate Professor in Biostatistics and Epidemiology with large experience in coordinating and executing large scale studies.

Vassiliki Costarelli (MS, PhD); Lecturer in Human Nutrition and Dietetics. Her research interest lies in eating and physical activity behaviours in different age groups.

Meropi Kontogianni (PhD); Lecturer in Clinical Nutrition, experienced in dietary patterns.

Evangelia Grammatikaki (MS, PhD candidate); dietician- public health nutritionist. Experienced in dietary, PA and fitness assessment, anthropometry and behavioural interventions..

Katerina Kondaki (MS, PhD candidate); exercise physiologist- public health nutritionist. Experienced in dietary, PA and fitness assessment in children and adolescents.

Georgia Kourlaba (MS, PhD); biostatistician, experienced in nutritional epidemiology.

Odysseas Androutsos (MS, PhD candidate); dietician-nutritionist. Experienced in anthropometry and implementation of nutrition interventions. He will be the project manager (WP1)

To be appointed: two research assistants who will be involved in WP2-8 for data collection and data analyses.

Recent publications relevant to the project

Manios Y, Kondaki K, Kourlaba G, Grammatikaki E, Birbilis M, Ioannou E (2008) Television viewing and food habits in toddlers and preschoolers in Greece; The GENESIS study. *Eur J Pediatr* October 3 Epub ahead of print

Manios Y, Costarelli V, Kolotourou M, Kondaki K, Tzavara C, Moschonis G (2007) Prevalence of obesity in preschool Greek children, in relation to parental characteristics and region of residence: the GENESIS Study. *BMC Public Health* 7(1):178

Manios Y, Kafatos I, Kafatos A. (2006) Ten Year Follow-Up Of The Cretan Health And Nutrition Education Program On Children's Physical Activity Levels. *Prev Med* 43(6):442-6

2. The **LUDWIG MAXIMILIANS UNIVERSITAET MUENCHEN, Germany (LMU)** is a leading academic centre in Germany, ranked again No. 1 in research performance nationwide by the German Research Council in 2008. The Department of Paediatrics has the highest research grant income of all non-clinical and clinical departments of the Medical Faculty of LMU. The department has one of the strongest paediatric nutrition programs in Europe, with respect to research, training, and impact on policy and practice.

Main tasks in the Toy Box project

The LMU will be lead the development of the ToyBox multi-component family involved intervention (WP6) and will also provide data for the secondary data analyses (WP2), conduct focus groups in Germany (WP3), implement and evaluate the cluster randomized intervention (WP7) and participate in the development of assessment tools for the evaluation of the intervention (WP8). It will also provide information at local level for WPs 5 and 9.

Previous experience relevant to those tasks

The Dr von Hauner Children´s Hospital of LMU (represented by Prof. Berthold Koletzko) has been or is involved as a partner in the EU projects FUFOSE, NUHEAL, PASSCLAIM, PERILIP, EURRECA and NUTRIMENTHE, and as coordinators in the FP5-programmes CHOP and PIANO and in the FP6-programme EARNEST, and as steering committee member in the DG SANCO project Breastfeeding Promotion in Europe.

The representative of this group, Prof. Berthold Koletzko has participated in national and international advisory groups on nutrition and health (e.g. EU Scientific Committee on Food, Pan-European collaboration on harmonization of European nutrient recommendations and of methodology of nutrient intake assessment for children and adolescents, WHO/FAO Codex Alimentarius Committee on Nutrition and Foods for Special Dietary Uses, expert panel on nutrition and consumer protection of the European Parliament, United Nations University/WHO/FAO expert consultation on global harmonization of nutrient intake standards, ESPGHAN Committee on Nutrition). The expertise in the field of nutrition and metabolism is documented by 219 original papers, 236 reviews, 150 book chapters and 20 books. Prof. Koletzko has initiated and implemented behavioural weight reduction programmes for children (PowerKids, more than 60.000 participating children and their families) and behavioural obesity prevention programmes for pre-school children (TigerKids, more than 120.000 participating children and their families).

Short profile of team members involved in the project

Prof. Berthold Koletzko (MD, PhD); Professor of Paediatrics

Hans Demmelmair (PhD); He has participated in numerous clinical trials and has been involved already in other EU projects.

Martina Scheer; Scientist. She has ample expertise in the effective management and execution of collaborative EU research from her active involvement in the management of the FP6 EARNEST project

Brigitte Anton Biologist. She has ample expertise in the effective management and execution of collaborative EU research from her active involvement in the management of the FP6 EARNEST project.

Recent publications relevant to the project

Demmelmair H, von Rosen J & Koletzko B (2006) Long-term consequences of early nutrition. Early Hum Dev 82, 567-574.

Koletzko B, Broekaert I, Demmelmair H, Franke J, Hannibal I, Oberle D, Schiess S, Baumann BT & Verwied-Jorky S (2005) Protein intake in the first year of life: a risk factor for later obesity? The E.U. childhood obesity project. Adv Exp Med Biol 569, 69-79.

Koletzko B, von Kries R, Closa Monasterolo R, Escribano Subías J, Scaglioni S, Giovannini M, et al. Infant Feeding and Later Obesity Risk. In: Koletzko B, Decsi T, Molnar D, de la Hunty A, editors. Early Nutrition Programming and Health Outcomes in Later Life. Springer Science; 2009. p. 15-29.

3a. The research program of the **Department of Movement and Sport Sciences (DMSS)**, research group Physical Activity, Fitness and Health group at the **UNIVERSITEIT GENT** (**Ghent University), Belgium** (**UGent**) has its main focus on health promotion and behavioural change with special attention to nutrition and physical activity. Based on the planning model of health promotion and behavioural change, studies are executed on (1) monitoring, measurement of nutrition and physical activity, including validity and reliability, (2) psycho-social and environmental determinants of both behaviours, (3) development of interventions for children, adolescents and adults, (4) effect and process evaluation studies and (5) dissemination activities.

Main tasks in the Toy Box project

The UGent-DMSS will lead WP3 (determinants and focus groups) and contribute to other WPs conducting literature reviews (WP2, WP4), collaborate in the development of the intervention (WP6) and contribute to WP7 and 8 (implementing and evaluating the intervention).

Previous experience relevant to those tasks

UGent-DMSS has expertise in accelerometer measurement in preschool children (4 year olds), in nutrition measurement as well as in the measurement of body composition in preschool children. UGent-DMSS also has a large expertise in intervention development and evaluation based on behavioural models and strategies.

Short profile of team members involved in the project

Prof dr Ilse De Bourdeaudhuij is Professor at the Department of Movement and Sport Sciences. She is a clinical psychologist and behaviour therapist and obtained a PhD in Health Psychology. Her research topics are the promotion of healthy eating and physical activity, including research on determinants and on developing and evaluating interventions to promote both health behaviours. She is currently president of the International Society of Behavioural Nutrition and Physical Activity. She is currently involved in 6 European projects (HELENA, IDEFICS, HOPE, TEENAGE, ALPHA, ENERGY).

Prof Dr. Greet Cardon is assistant professor in the field of physical activity and health. Her main research topics are the assessment of PA and fitness in children and adolescents designing questionnaires and objectively measuring PA, and the development and evaluation of interventions to promote physical activity in young children and adolescents.

Prof. Dr Benedicte Deforche is assistant professor in the field of physical activity, fitness, health and overweight. Her main research topics are PA in youngsters with overweight and obesity.

Dr Leen Haerens is post-doc in the field of nutrition and physical activity, her main research topics are development, evaluation and implementation of a school-based interventions promoting PA and healthy eating. She also has a special competence in sport pedagogics and physical education.

To be appointed: a research assistant who will support in reviewing the literature (WP2, 3 and 4), organising and conducting the focus group interviews (WP3), and support intervention development (WP6), evaluation of the intervention (WP7) and development of assessment tools (WP8).

Recent publications relevant to the project

De Bourdeaudhuij, I., Lefevre, J., Deforche B., Wijndaele, K., Matton, L., Philippaerts, R. Physical activity and psychosocial correlates in a community sample of normal weight and overweight 11-19 year olds. Obesity Research, 2005;. 13: 1097-1105.

Haerens, L., Deforche, B., Maes, L., Cardon, G., Stevens, V., De Bourdeaudhuij, I. Evaluation of a two-year physical activity and healthy eating intervention in middle school children. Health Education Research,2006; 21, 911-921.

Cardon, G. & De Bourdeaudhuij, I. (2008) Are preschool children active enough? Objectively measured physical activity levels. Research quarterly for exercise and sport, 79 (3) 326-332.

Cardon, G., Van Cauwenberghe, E., Labarque, V., Haerens, L., De Bourdeaudhuij, I. (2008). The contribution of preschool playground factors in explaining children's physical activity during recess. International Journal of Behavioral Nutrition and Physical Activity**,** 5 (Nr 11), Feb 26.

Cardon, G., De Bourdeaudhuij, I. (2007). Comparison of pedometer and accelerometer measures of physical activity in pre-school children. Pediatric Exercise Science, 19(2), 205-214.

3b. The **Department of Public Health (DPH)** in **UNIVERSITEIT GENT** (**Ghent University), Belgium (UGent)** has a long-standing experience in descriptive, analytical and experimental epidemiological studies and in health promotional intervention studies. The scientific interest area of the department is extremely wide and very sensible for permanent social and public evolutions. Three research units of the department will participate in the consortium. The Unit of "Health Information, Education and Health Promotion" is concentrating on the survey of health behaviour of young people, the development of programmes for information and education and their appropriateness. The Unit of "Public Health Nutrition" gained experience in the broad research field of human nutrition and public health related to the growing challenge of obesity. The third Unit "Health Economics" is a new but experienced unit and focuses on health economic evaluations in various preventive and therapeutic areas.

Main tasks in the Toy Box project

The UGent-DPH will lead WP9 (cost-effectiveness) and contribute largely to WP5 (policy related to (pre)school-based intervention programs). It will also contribute to other WPs conducting literature reviews (WPs 2-4), collaborate in the development of the intervention (WP6) and contribute to WPs 7-8 (implementing and evaluating the intervention). The different units will contribute to the dissemination task (WP10) as well.

Previous experience relevant to those tasks

UGent-DPH has already built up substantial experience in the field of human nutrition and public health and has as such been involved in several national and international projects in the field of public health nutrition (HELENA, Idefics, EURONUT, SENECA, DAFNE, EURODIET and EFCOSUM, EUROFI). At national level, they serve as scientific advisory bodies of the national food consumption surveys for Belgium. It is partner of the Flemish Health and Environment Centre.

Staff involved in the project:

Prof. Dr Lea Maes (PhD) is professor at the department of public health. She is a social scientist with a PhD in medical sciences. Her research expertise is in the field of nutrition, monitoring nutrition behaviour in adolescents, developing and evaluating interventions.

Prof. Dr. Stefaan De Henauw is professor at the Department of Public Health. He is a medical doctor with a PhD in Public Health and Master in Human Nutrition. He is coordinator of a Public Health Nutrition research unit. His research topics are cardiovascular and nutritional epidemiology. Recent interests are community-based interventions in the context of obesity.

Prof. Dr. Lieven Annemans is professor of Health Economics at Ghent University and Brussels University. He is Past-President of the Intern. Society for PharmacoEconomics and Outcomes Research, and chairman of the Flemish Health Council. He has experience in health economic evaluations in various preventive and therapeutic areas. His educational record includes Master's degrees in Physical Education and in Management, a Degree of Commercial Engineer, a Degree in Health Economics and a PhD in Economics. His main current interests are health economic evaluation of prevention, personalized medicine, and payment systems.

Dr Inge Huybrechts, Dr Christophe Matthys, Dr Isabelle Sioen are post-doctoral researchers in the field of public health nutrition.

Delphine De Smedt is a research assistant with a Master degree in Biomedical Science and a Master degree in Economics. She has gained experience in the health economic evaluation of nutrition and physical exercise programs related to the health objectives initiative of the Flemish Ministry of health.

To be appointed: a research assistant who will execute the cost-effectiveness study (WP9) and the research on policy related to (pre)school-based interventions in the different countries (WP5).

Recent Publications:

Lachat C, Van Camp J, De Henauw S, Matthys C, Larondelle Y, Remaut – De Winter AM, Kolsteren P. A concise overview of national nutrition action plans in the European Member States. *Public Health Nutr.* 2005;8(3):266-74.

Maes, L., Vereecken CA, Gedrich K, …, Matthys, C, on behalf of the HELENA Study Group. A feasibility study of using a diet optimization approach in a web based computer tailoring intervention for adolescents. International Journal of Obesity *2008;* **32**, S76 - S81.

Annemans L, Lamotte M, Clarys P, Van den AE. Health economic evaluation of controlled and maintained physical exercise in the prevention of cardiovascular and other prosperity diseases. Eur J Cardiovasc Prev Rehabil 2007 December;14(6):815-24.

4. The **EMGO Institute** is - with approximately 200 FTE research personnel and an annual output of over 500 research publications - the largest research institute of the **VERENIGING VOOR CHRISTELIJK HOGER ONDERWIJS WETENSCHAPPELIJK ONDERZOEK EN PATIENTENZORG** (**VU University Medical Center)** (**VUmc**) with a main focus on public health research. EMGO is accredited by the Dutch Royal Academy of Arts and Sciences (KNAW) and in 2004 EMGO's research was rated as excellent on all counts, according to criteria of the KNAW. One of EMGO's main focus is on overweight and obesity-related research. The EMGO institute researchers include all disciplines necessary to study the determinants and possible solutions for prevention of overweight and obesity in children and has ample experience in research on determinants and interventions related to obesity prevention, including school-based studies.

Main tasks in the Toy Box project

The VUmc will lead the identification of the most important behaviours related to overweight and excessive weight gain among 4-6 year-olds and to further identify risk groups in which overweight and unhealthy energy balance related behaviours are highly prevalent (WP2). In addition, in this work package the most important parental determinants will be identified. They will further participate in other WPs, by providing feedback for energy balance related behaviours that will be explored in focus groups (WP3), addressed and reviewed for effective methods and educational strategies (WP4), included in the developed intervention material (WP6), for which assessment tools should be developed (WP8).

Previous experience relevant to those tasks

The group members are currently involved in several EU funded studies: the FP6- funded Eurocadet project, FP5-funded Pro Children project, ALPHA project and EUNAAPA (European Network for Action on Ageing and Physical Activity). The group was also coordinating the recently FP7-funded ENERGY project and the FP6-funded Hope project.

Short profile of team members involved in the project

Prof Johannes Brug (PhD) will be the contact person and work package leader of WP2. He is the director of the EMGO institute and professor of epidemiology. His research is mainly focussed on predicting, explaining and promoting lifestyle nutrition and PA behaviours.
Dr Mai JM Chin A Paw (PhD). She is an associate professor with a background in human movement science and epidemiology. She is experienced in several research methodologies including Intervention Mapping, systematic reviews, and analyses of determinants, mediators and moderators. She is project leader of several projects promoting healthy behaviour among children, of which several school-based interventions including children aged 4-12 years.
Dr Saskia J te Velde (PhD) is a post-doctoral researcher with a background is in human movement science and epidemiology. She is strong in methodology and statistics and will support the review and analyses to be conducted in WP2. She is currently involved in an intervention project aimed at 0-4 year olds and their parents that visit Youth Health Care centres in the Netherlands. Moreover, she is project leader of several projects that investigate determinants of energy balance-related behaviours or that evaluate interventions promoting healthy behaviour among school children.
To be appointed: a postdoctoral researcher for secondary data analyses (WP2).

Recent publications relevant to the project

Chin A Paw M, Singh AS, Brug J, Van Mechelen W. Why did soft drink consumption decrease but screen time not? Mediating mechanisms in a school-based obesity prevention program. Int J Behav Nutr Phys Act 2008;5:41.
van der Horst K, Chin A Paw MJM, Twisk JWR, van Mechelen W. A brief review on correlates of physical activity and sedentariness in youth. Med Sci Sports Exerc 2007;39(8):1241-50.
Hume C, Singh A, Brug J, van Mechelen W, Chinapaw M. Dose-response associations between screen time and overweight among youth. Int J Pediatr Obes. 2008 Jun 26:1-4.

5. The group at the **UNIVERSIDAD DE ZARAGOZA** (**University of Zaragoza), Spain (UniZar**) has a multidisciplinary background, implementing a multidisciplinary-based intervention combining nutritional-physical activity-psychological support approach. This research programme is supported by the Spanish Ministry of Health (FIS PI052451, www.estudioevasyon.com/). All physicians are experts in running clinical and public health investigations in conjunction with nurses, dieticians and physical activity trainers, in performing body composition techniques and indirect calorimetry. The main research activity of the group concerns the study of nutritional status and body composition in children and adolescents.

Main tasks in the Toy Box project

The UniZar will lead the development of assessment tools for the evaluation of the intervention (WP8). It will further provide data for the secondary data analyses (WP2), conduct focus groups in Spain (WP3), participate in adapting the developed intervention scheme (WP6), in implementing and evaluating the cluster randomized intervention (WP7) and also provide information at local level for WPs 5 and 9.

Previous experience relevant to those tasks

The group at the University of Zaragoza (UNIZAR, Spain) is currently involved in three EU funded studies. UNIZAR is co-ordinating HELENA (Healthy Lifestyle in Europe by Nutrition in Adolescence, contract number FOOD-CT-007034), dealing with adolescent nutrition and lifestyle (www.helenastudy.com). UNIZAR is also a member of the consortium involved in the Integrated Project called IDEFICS (Identification and prevention of Dietary- and lifestyle-induced health Effects In Children and infantS, contract number 016181-2), dealing with the primary prevention of obesity in infants and children (www.idefics.eu). UNIZAR is also participating in a Network of Excellence called EURRECA (EURopean RECommendations Aligned, harmonising nutrient recommendations across Europe with special focus on vulnerable groups and consumer understanding, contract number 036196-2); in this network, UNIZAR is the responsible of the research activity on recommendations for children and adolescents (www.eurreca.org). Prof. Luis Moreno, leader of the group has been Scientific Evaluator during the 5th and 6th Framework Programme of the European Union. He has coordinated several epidemiological surveys in the city of Zaragoza and the region of Aragón. He is a member of the European Childhood Obesity Task Force of the European Association for the Study of Obesity, and member of the European Society for Paediatric Gastroenterology, Hepatology and Nutrition (ESPGHAN) Committee of Nutrition.

Short profile of team members involved in the project

Prof. Luis A. Moreno (MD); Professor of Public Health at the University of Zaragoza; leading the group at UniZar.

Jesús Mª Garagorri (MD); Paediatric Endocrinologist -Professor of Paediatrics. He has experience in the management of obese patients and conduction of multidisciplinary-based interventions combining a nutritional-physical activity-psychological support approach.

Gloria Bueno (MD, PhD); Paediatric Endocrinologist -Professor of Paediatrics. She has experience in the management of obese patients and in running clinical studies in conjunction with dieticians and psychologists

Gerardo Rodríguez; Professor of Paediatrics. He has experience in the assessment of the nutritional status and body composition of children and adolescents.

Germán Vicente-Rodriguez; Associate Professor, in the field of Sports Sciences. He has experience in the assessment of physical activity and fitness in children and adolescents.

María Isabel Mesana; researcher in the field of Nutrition. She has experience in the assessment of dietary intake in children and adolescents.

Recent publications relevant to the project

1) Labayen I, Moreno LA, Blay MG, Blay VA, Mesana MI, González-Gross M, Bueno G, Sarría A, Bueno M. Early programming of body composition and fat distribution in adolescents. J Nutr 2006; 136: 147-152.

2) Labayen I, Moreno LA, Ruiz JR, González-Gross M, Wärnberg J, Breidenassel C, Ortega FB, Marcos A, Bueno M, The AVENA Study Group. Small birth weight and later body composition and fat distribution in adolescents: The AVENA Study. Obesity (Silver Spring) 2008; 16: 1680-1686.

3) Ortega FB, Tresaco B, Ruiz JR, Moreno LA, Martín-Matillas M, Mesa JL, Wärnberg J, Bueno M, Tercedor P, Gutiérrez A, Castillo MJ. Cardiorespiratory fitness and sedentary activities are associated with adiposity in adolescents. Obesity (Silver Spring) 2007; 15: 1589-1599.

6. The Unit for Health Communication is located within the department of health, nutrition and management at Høgskolen i Oslo og Akershus, **Lilleström, Norway (HiOA)**. The unit is predominantly dealing with issues concerning children's health and nutrition and health communication. Among previous activities, the leader of the group has been instrumental to the development of the European Master Programme in public health nutrition, and has been active in collecting information on breastfeeding prevalence as well as breastfeeding promotion in several European projects. The new unit consists of Professor Agneta Yngve, PhD student Susanna Kugelberg (Master of political science), PhD student Christel Lynch (nutritionist and manager of the project Pro Greens, part time in Oslo) and university lecturers Jenny Rossen (10%) and Bettina Ehrenblad (10%). The department as such holds ten employees in the nutrition area, including two professors and several senior lecturers. The Norwegian unit is very much linked to the Unit for Public Health Nutrition at Karolinska Institutet, and where most of the staff mentioned above is also attached.

Main tasks in the Toy Box project

The **HiOA**will overview policies and practices for school based interventions in Europe (WP5) and participate in the dissemination of the ToyBox findings to inform relevant stakeholders and public policy makers (WP10).

Previous experience relevant to those tasks

Prof Agneta Yngve and her team have previous experience in projects addressing the following issues: Assessing breastfeeding prevalence, weaning food quality and growth performance in Iranian infants; assessing growth variables of Swedish 7-9 year old children as a part of the WHO European childhood growth surveillance initiative; promoting fruit and vegetable intake in children in schools in ten European countries in the PROGREENS project, supported by the European Commission DG Health and Consumers; identifying effective policies to prevent obesity in the HOPE project, supported by the European Commission DG Research; identifying components in breast milk important for growth and health development; promoting physical activity in a worksite health promotion project; preventing obesity in a clinical trial of overweight patients in a primary care setting. The team has recently been instrumental in collecting information on prevalence of overweight and obesity in the 53 countries of WHO Europe for the WHO report published in 2007.

Short profile of team members involved in the project

Agneta Yngve (MSc, MPH, PhD); public health nutritionist, Professor and Head of Unit. She is also the Editor in Chief for the journal Public Health Nutrition.

Susanna Kugelberg, M Pol Sci, PhD student, specialized in qualitative studies of competencies of nutrition staff and analysis of policy documents;

Christel Lynch, MSc, PhD student, manager of the ProGreens project and with a background in nutrition policy analysis, school based interventions and behaviour change models.

Jenny Rossen, MSc, nutritionist and PE teacher, university lecturer in public health nutrition

Bettina Ehrenblad, MSc, nutritionist, with experience in developing self-assessment tools for schools in nutrition and physical activity and instrumental in the intervention within the ProGreens project. She has also been involved in the collection of data on growth and infant feeding.

Recent publications relevant to the project

Yngve A, De Bourdeaudhuij I, Wolf A, Grjibovski A, Brug J, Due P, Ehrenblad B, Elmadfa I, Franchini B, Perez Rodrigo C, Poortvliet E, Rasmussen M, Thorsdottir I, Klepp KI. Differences in prevalence of overweight, obesity, underweight and stunting in 11-year olds across Europe: The Pro Children Cross-sectional Study. European J Public Health 2008 Apr;18(2):126-30.

Grjibovski A, Ehrenblad B, Yngve A. Breastfeeding in a group of Swedish children: socio-demographic determinants and associations with adiposity in childhood and adolescence. Int J Breastfeeding, 2008 Sep 16;3:23

Villa I, Yngve A, Poortvliet E, Grjibovski A, Liiv K, Sjöström M, Harro M. Dietary intake among under-, normal and overweight 9- and 15-year old Estonian and Swedish schoolchildren. Public Health Nutrition 2007, 10(3):311-22.

7. The Food, Physical Activity and Obesity Research Group in **UNIVERSITY OF DURHAM, UK (UDUR)** focuses on 1) the collation of the evidence base for the prevention of obesity in both children and adults, and dissemination and translation of this evidence into policy and practice 2) the development of community-based initiatives and interventions to prevent obesity in children and adults 3) the development of tools to assess dietary intake and physical activity levels in population level studies. The need to assess and reduce health inequalities underpins this programme of work.

Main tasks in the Toy Box project

The UDUR will be responsible for conducting a systematic review on effective behavioural methods providing the theoretical framework for school based interventions promoting healthy weight and desired energy balance related behaviours in children (WP4). It will also participate in WPs 6 and 8 providing feedback for the development of the intervention and the development of the assessment tools

Previous experience relevant to those tasks

The group's leader Prof. Carolyn Summerbell has significant experience in managing research projects and staff as part of her current and previous roles, and can provide to appropriate networks and key stakeholders as part of her existing portfolio of activity. She has a strong track record of attracting research grants from a variety of funding sources, has co-authored over 80 peer-reviewed research papers (including Cochrane reviews), and is regularly invited to give key note addresses at international conferences. She will be a member of the NIHR Public Health Research programme Research Funding Board starting May 2009. Professor Summerbell is a member of the Editorial Board of the *International Journal of Obesity* and the *British Journal of Nutrition*, and is a regular temporary advisor to WHO. She is Honorary Secretary of the Nutrition Society and Chair of the Association for the study of Obesity. She is currently leading the designation of a new *WHO Collaborating Centre for Nutrition* which is expected to be approved in February 2009, which will have a specific remit for co-ordinating the WHO led (but multi-agency) Nutrition Friendly Schools Initiative.

Short profile of team members involved in the project

Professor Carolyn Summerbell (PhD); Professor in Principal and Professor in Nutrition in the School of Medicine and Health

Dr Helen Moore (PhD); Research Fellow who is an accomplished information scientist. She has project led a number of systematic review, including Cochrane reviews.

Ms Tamara Brown (PhD); Research Fellow (expected to complete her PhD in Spring 2009) and has undertaken three large-scale systematic reviews for the NHS Health Technology Assessment programme, including a systematic review of the long-term effects and economic consequences of treatments for obesity and implications for health improvement.

To be appointed: one research assistant to help in WP4

Recent publications relevant to the project

1. Brown T, Kelly S, Summerbell C. Prevention of obesity: a review of interventions. *Obesity Reviews* (2007) 8 (Suppl 1) 127-130 .

2. Summerbell CD, Waters E, Edmunds LD, Kelly S, Brown T, Campbell KJ. Interventions for preventing obesity in children. *The Cochrane Database of Systematic Reviews*; Issue 3, 2005.

3. Moore H, Summerbell C, Hooper L, Cruickshank K, Vyas A, Johnstone P, Ashton V, Kopelman P. Dietary advice for treatment of type 2 diabetes mellitus in adults. *The Cochrane Database of Systematic Reviews*; Issue 2, 2004.

8. The Bavarian **Staatsinstitut für Frühpädagogik** (**State Institute of Early Childhood Research), Germany (IFP**) is a research centre focusing on innovative projects in early childhood education, research into early learning, and knowledge transfer for practitioners and policy-makers. The IFP has strong links with other research institutes and specialist institutions at the national, European and international level.
The main goals of the IFP are:

- To conduct basic and applied research in the fields of developmental psychology and pedagogy, with particular reference to centre-based early childhood education and care,
- To develop, evaluate and disseminate innovatory approaches in early childhood education and for work with children with special needs,
- To develop strategies for promoting co-operation between kindergartens, families, schools and other facilities,
- To develop and evaluate strategies for improving the initial education/training and continuing professional development of early years personnel.

Main tasks in the Toy Box project

The IFP will participate in reviewing the literature on educational strategies for the formation of desired energy balance related behaviours (WP4) and also participate in developing the intervention material in WP6.

Previous experience relevant to those tasks

The IFP was responsible for developing the Bavarian Early Education Curriculum which is a mandatory framework for all kindergartens in Bavaria. Healthy behaviour and healthy food choices as well as physical activity are central aims of the curriculum.
The IFP was involved in the development of behavioural obesity prevention programmes for pre-school children (TigerKids, more than 120,000 participating children and their families).

Short profile of team members involved in the project

Susanne Kreichauf (MPH) is psychologist and epidemiologist with a focus on the prevention of overweight in infancy and childhood.
Heinz Krombholz (PhD) is psychologist and sports scientist with a focus on the promotion of physical activity in kindergarten.

Recent publications relevant to the project

Krombholz, H. (2007). Körperliche und motorische Entwicklung im Säuglings- und Kleinkindalter. In M. Dartsch (Hrsg.), Musikalische Bildung von Anfang an. S. 53-58. Bonn: VdM Verlag.

Krombholz, H. (2006). Physical performance in relation to age, sex, birth order, social class, and sports activities of preschool children. Perceptual and Motor Skills, 102, 477-484.

Krombholz, H. (2005). Bewegungsförderung im Kindergarten – Ein Modellversuch. Schorndorf: Hoffmann.

9. **The INSTITUT POMNIK CENTRUM ZDROWIA DZIECKA (Children's Memorial Health Institute), Warsaw, Poland (CMHI)** is a big referral hospital in Poland. It covers all pediatric specialities, there are 550 beds for children in the in-patient clinics. It is also a leading pediatric research center. Research covers not only problems of serious and chronic diseases but also public health issues. The institute offers services and expertise on consulting governmental and non-governmental agencies on issues related to the development and implementation of public health policies. It provides scientific support to the food industry, nutrition counselling and health promotion services to the community.

Main tasks in the Toy Box project

The CMHI will provide data for the secondary data analyses (WP2), conduct focus groups in Poland (WP3), and further participate in developing and adapting the developed intervention scheme (WP6), in implementing and evaluating the cluster randomized intervention (WP7) and in contributing to the development of assessment tools for the evaluation of the intervention (WP8). It will also provide information at local level for WPs 5 and 9.

Previous experience relevant to those tasks

Researchers from the institute are involved in various multicentre studies in infants and children funded by national grants. The groups from CMHI have also been involved as partners in the FP5-programme CHOP, FP6-programme EARNEST and FP7-programme NUTRIMENTHE.

Short profile of team members involved in the project

Dr Piotr Socha (MD, PhD); Professor in Pediatrics and Gastroenterology. He will be the project coordinator for CMHI. He has experience in coordinating and conducting studies concerning obesity, nutrition, liver and gastrointestinal problems. He participated in numerous national research projects (he led 3 national projects) and he was involved in 4 EU projects.

Dr Zbigniew Kulaga (MD). He will be responsible for logistics of the project. He is the head of the PUBLIC HEALTH Division. He took overall responsibility (design, protocol writing, EC approval, investigators selection and training, sub-contractors selection, logistics, subject sampling, data management supervision, medical statistics) for a study on Polish children blood pressure reference ranges.

Dr Anna Jakubowska (PhD); a psychologist. The professional experience: 20 years of professional involvement in psychology of patients with chronic illness. She organized workshops/lectures for students of psychology of the University of Warsaw, of the College of Social Psychology and of the Warsaw Medical University as well as courses and trainings in psychology for medical staff, nurses, medical students and graduated physicians.

Joanna Neuhof-Murawska (MS), a dietician. She has experience in epidemiological and clinical surveys in children as well as in dietary assessment.

Dr Mieczyslaw Litwin (MD, PhD), Professor in Pediatrics and Nephrology. He has been involved in numerous epidemiological studies concerning obesity and arterial hypertension. His expertise will be used for project planning, evaluation and interpretation of results.

Dr Dariusz Gruszfeld (MD, PhD). He was actively involved in 3 EU projects (CHOP, EARNEST, NUTRIMENTHE) at each step of the projects. His expertise in methodology, logistics will be used for project planning, evaluation and interpretation of results.

Recent publications relevant to the project

Litwin M, Socha P. Obesity, insulin resistance, metabolic syndrome and graft function in children after renal transplantation - What does really matter? Pediatr Transplant. 2008 Nov 10

Teisseyre M, Wierzbicka A, Socha P, Pawłowska J, Jankowska I, Skorupa E, Socha J. Risk factors of atherosclerosis in children after liver transplant. Pediatr.Transplant. 2007, 11 suppl. 1: 74

Socha P, Koletzko B, Demmelmair H,Jankowska I, Stajniak A, Bednarska-Makaruk M, Socha J. Short-term effects of parenteral nutrition of cholestatic infants with lipid emulsions based on medium-chain and long-chain triacylglycerols. Nutrition 2007, 23: 121-126

10. The **Medical University of Varna, Bulgaria (MUV)** is a fundamental, educational, scientific and medical centre in Northeastern part of Bulgaria and the Black Sea region. Its structure includes Faculties of Medicine, Public Health and Dental Medicine. It is supplied with modern equipment at a high level of information provision. Within MUV, the Faculty of Public Health is a major division in the Medical University-Varna and incorporates 8 departments. The main activities of the Faculty of Public Health and the Dept. of Social Medicine are devoted to public health education, research, training and consultancy. The topic of the Project fully corresponds to department's research interests and its staff members had worked in the field of childhood obesity from the public health perspective. The Department has substantial experience in international (education and research) projects participation and coordination and very successful collaboration with international partners. The FPH has close links of cooperation with national and regional governments and local health and social services that would be essential for fulfilling the project objectives.
The Dept. of Pediatrics and Medical Genetics also established within MUV is the centre of research, clinical excellence and methodological help in North-Eastern Bulgaria. Its main topics are chronic/rare diseases. The Paediatric Endocrinology team created 17 years ago has a long standing history of research in the fields of childhood diabetes epidemiology with a Register established in 1982 and participation in DiaMond and EURODIAB projects; postnatal growth and puberty in children born small for gestational age; rare syndromes and diseases. Clinical and research fellowships, grants for high score of presented work and successful collaborations were fulfilled throughout the years. It is a member of IASO, EASO, ESPE, ISPAD, IAA among others.

Main tasks in the Toy Box project

The MUV will provide data for the secondary data analyses (WP2), conduct focus groups in Bulgaria (WP3), and further participate in developing and adapting the developed intervention scheme (WP6), in implementing and evaluating the cluster randomized intervention (WP7) and in contributing in the development of assessment tools for the evaluation of the intervention (WP8). It will also provide information at local level for WPs 5 and 9.

Previous experience relevant to those tasks

The MUV has long standing expertise in anthropometric studies, longitudinal observations, cross-sectional studies and the necessary statistical back up. Previous and ongoing projects concern TV food advertising to children, metabolic syndrome, GPs attitude towards childhood obesity issues, waist circumference references for 6-18 years old Bulgarian children. Recently they participate in "InForm" Campaign against obesity in children and adolescents, (funded by the European Executive Agency of Public Health) comprising eleven partners from 8 countries.

Short profile of team members involved in the project

Prof. Iotova is a Paediatric Endocrinologist. She is a member of the Educational and Training Committee of ESPE (European Society of Pediatric Endocrinology) and invited member of ESPE Clinical Fellowship Committee.
Dr. Sonya Galcheva (PhD) is interested in obesity in childhood and adolescence and is the recipient of the EASO Young Investigator Award in 2008 (May 2008, Geneva).
Krassimira Stoeva- Aleksandrova (Msc, MPH, PhD) She has 10 years of experience in epidemiologic studies on health determinants of cardio-vascular diseases, incl. behavioural, demographic and socio-economic factors. She is initiator and principal investigator of the Salt and Health Study (2003-2005), conducting a randomised population intervention trial for salt reduction, based on the behaviour change models (Health Belief Model, TTM, Social learning theory).

Recent publications relevant to the project

Galcheva S., V. Iotova, V. Stratev. Television food advertising directed to Bulgarian children.. Arch Dis Child. 2008 May 2. [Epub ahead of print]
Galcheva S, V Iotova, V Tzaneva, V Stratev, K Petrova, M Georgieva. Risk factors for overweight and obesity among Bulgarian children and adolescents, aged 6-18 years. 47th Annual Meeting of European Society for Paediatric Endocrinology (ESPE), Istanbul, Turkey, September 20-23, 2008. Horm Res 2008; 70 (suppl 1). P2-d1-441.
Stoeva K, Feschieva N, Popova S, Dokova K, Perceived barriers to healthy eating among Bulgarian adults - demographic and socio-economic variations, 4th Annual Conference of the International Society of Behavioral Nutrition and Physical Activity (ISBNPA), June 16-18, 2005, Amsterdam, Abs. Book, p.184;

11. The **INTERNATIONAL ASSOCIATION FOR THE STUDY OF OBESITY, London, UK (IASO)** and its policy section, the International Obesity TaskForce (together known as IASO-IOTF) lead the world in promoting issues concerning obesity and obesity policies. IASO-IOTF is a 10,000-member scientific society and research dissemination body with over 50 national and regional associations around the globe.
IASO-IOTF has wide experience in advising policy makers and as such is well-suited to disseminate the results of the proposed programme of work to local policy makers responsible for obesity prevention in the general public. It' s member organization, the European Association for the Study of Obesity, organizes the annual European Congress on Obesity and consists of a scientific network of national associations for the study of obesity.

Main tasks in the Toy Box project
The IASO will lead WP10, where using its wide network of people in the field and policymakers, it will be responsible for disseminating the findings to the people in the field, the policymakers and other stakeholders.
Previous experience relevant to those tasks
IASO-IOTF is the main independent coordinating and advocacy organisation for obesity prevention research and action, and is an important network of expertise and advice, providing assistance to the World Health Organisation and aiding the launch of the DG Sanco European Platform on Diet, Physical Activity and Health. It was responsible for assisting the European regional office of WHO in the preparation and organisation of the ministerial conference on obesity in Istanbul 2006 and for the organisation of follow-up on the charter on curbing the obesity epidemic that was signed there.
IASO-IOTF is currently a partner in the EC-funded HOPE project on obesity, and a partner in the EAHC-funded DYNAMO-HIA project, is an adviser to the EC-funded EURO-PREVOB project, and is the Principal Investigator on the EAHC-funded POLMARK project.
IASO has been involved in several European Commission-funded research projects, including the HOPE (Health Promotion through Obesity Prevention in Europe) project, the DYNAMO-HIA (Dynamic Modelling for Health Impact Assessment), the EAHC-funded project assessing national policies for the regulation of marketing of food to children (POL-MARK). Tim Lobstein, Director of the IOTF-IASO Child Obesity Programme, is also a member of the advisory board for the FP6-funded EURO-PREVOB project on obesity policy analysis, and was an adviser on the DG Sanco-funded project on child obesity prevention policies (EHN-CHOB), and on the steering committee of the EURODIET project. He has previously been a consultant on food policy and public health nutrition for the World Health Organization, the Association of Schools of Public Health in the European Region, the European Science and Technology Observatory, the European Public Health Alliance and the European Commission, for example drafting the Status report on the European Commission's work in the field of nutrition (2002) for DG Sanco.

Short profile of team members involved in the project (1 male)
Tim Lobstein (PhD); senior staff member and Director of the IOTF-IASO Child Obesity Programme. He has been a specialist consultant on food policy for several decades. He is also a visiting research fellow at the Science and Technology Policy Research Unit at the University of Sussex. He has ample experience in disseminating research finding to policy makers and other stakeholders and will therefore lead WP10.
To be appointed: junior staff member, who will support Dr Lobstein and contribute in all dissemination tasks (WP10).
Recent publications relevant to the project
Gonzalez-Zapata LI, Alvarez-Dardet C, Ortiz-Moncada R, Clemente V, Millstone E, Holdsworth M, Sarri K, Tarlao G, Horvath Z, Lobstein T, Savva S. Policy options for obesity in Europe: a comparison of public health specialists with other stakeholders Public Health Nutr. 2008, 29:1-13.
Lobstein T, Baur LA. Policies to prevent childhood obesity in the European Union. Eur J Public Health. 2005 15(6):576-9.
Lobstein T, Baur L, Uauy R; IASO International Obesity TaskForce. Obesity in children and young people: a crisis in public health. Obes Rev. 2004;5 Suppl 1:4-104.

12. The **NATIONAAL INSTITUUT VOOR GEZONDHEIDSBEVORDERING EN ZIEKTEPREVENTIE** (**Netherlands Institute for Health Promotion and Disease Prevention), the Netherlands (NIGZ)** has been actively involved in the European Network for Health Promoting Schools since 1992 coordinated by the WHO Euro in Copenhagen. Since 2007 NIGZ runs the secretariat of the network which now has a new name: Schools for Health in Europe network, or SHE network. NIGZ has a central position in the Netherlands in developing and implementing programmes in support of healthy behaviour and healthy environments. NIGZ motivates, supports and facilitates people in choosing and living a healthy lifestyle. As a national operating institute it supports the local public health policy and their professionals and has structural, national programmes for various target groups, including youth and adolescents, the elderly and deprived socio-economic groups. NIGZ works for settings: school, work, community, care.

Main tasks in the Toy Box project

The NIGZ will lead WP5 making an overview of policies on school and school based health promotion activities in Europe and will develop tools for the assessment of these policies. It will also participate in the dissemination of the ToyBox findings to inform relevant stakeholders and public health policy makers (WP10).

Previous experience relevant to those tasks

NIGZ has been actively involved in the European Network for Health Promoting Schools since 1992 coordinated by the WHO Euro in Copenhagen. Since 2007 NIGZ runs the secretariat of the network which now has a new name: Schools for Health in Europe network, or SHE network.

NIGZ has an excellent reputation in project management in health promotion, quality assessment (Preffi) and innovation. It plays a central role in the national development and implementation of school health promotion in the Netherlands. NIGZ has developed the national Health Promoting School method, in collaboration with 9 national health-theme institutes. NIGZ runs the annual Go for health campaign for primary schools as an agenda-setting activity. Since 2008 NIGZ acts as a WHO collaborating centre for school health promotion.

Short profile of team members involved in the project

G.J.Buijs, (MSc) Manager SHE network, national and international expert on school health promotion, employed as senior adviser at the Netherlands Institute for Health Promotion and Disease Prevention NIGZ. He is project coordinator of the HEPS project, funded by EC, DG Sanco.

S. de Ruiter, (MSc) Project officer SHE network, expert in school health promotion, employed as project officer at the Netherlands Institute for Health Promotion and Disease Prevention (NIGZ). She is project officer for the HEPS project.

Z. Dafesh, (MSc) Project officer HEPS project, employed as senior adviser at the Netherlands Institute for Health Promotion and Disease Prevention NIGZ. She is project coordinator of PRIMA, the Dutch anti-bullying method for primary schools.

Recent publications relevant to the project

Barnekow, V., Buijs, G. et al (2007). Health-promoting schools: a resource for developing indicators. WHO Euro, Copenhagen.

Bessems, K., Ruiter, S. de, and Buijs, G (2006). *Toolkit Overgewicht: preventie van overgewicht binnen de setting school. [Toolkit for overweight prevention in schools]* : Woerden: NIGZ.

Dafesh, Z. et al (2006). *Regioscan GGD en gezondheid op scholen. Onderzoek naar de stand van zaken rond de ondersteuning op scholen door GGD-en op het terrein van gezondheid [regional scan on school health promotion and the role of the regional health services in the Netherlands].* NIGZ, Woerden.

13. The **AOK- Verlag GmbH, Germany (AOK-Verlag)** has been founded 1914 by the “Allgemeine Ortskrankenkasse”, an important player in the compulsary german health system. The company is still owned by this organisation but has to earn its own money by publishing information and prevention products and providing fulfillment services to AOK and its partners in various public health related projects. The company is solidly financed and is currently without any bank loans or other liabilities.

Main tasks in the Toy Box project
The AOK-Verlag will participate in developing the ToyBox multi-component family involved intervention scheme (WP6) and provide cost information for the cost-effectiveness evaluation (WP9).

Previous experience relevant to those tasks
The near Bonn based company has been a partner to Ludwig-Maximilians–University Muenchen since the early beginnings of the German Tigerkids project. They have contributed the classical publishing house works, illustrations and DTP, managed the production and distribution to the Kindergartens. Successful projects are “Sanftes Rückentraining” (spine training), Go!Kids (moving activities) both in collaboration with the University of Bayreuth, Relax Kids, LegaKids and PowerKids in collaboration with LMU.

Short profile of team members involved in the project (1 male, 1 female)
Dipl.kfm. Otto Gmeiner has been the Managing Director of AOK-Verlag for more than 10 years. His professional background is the publishing business which includes employments for Reader´s Digest, EMAP plc and IMP AB, Sweden. He has been closely involved in developing Tigerkids and the successful implementation in Germany with more than 120.000 participating children and their families up until now.

Dip.oec.troph Jutta Retterath is the responsible Marketing Manager of the Tigerkids Project. Her expertise in nutrition sciences but also her professional experience as a practical nutrition adviser has given valuable input to many of our projects and certainly to Tigerkids.

Recent work relevant to the project
Sanftes Rückentraining: spine training in collaboration with the University of Bayreuth

Go!Kids: moving activities for children in collaboration with the University of Bayreuth

Relax Kids

LegaKids computer based activities to help young children read and write http://www.lega-kids.net

PowerKids http://www.powerkids.de/ One of the most complex tasks was the production of the powerkids box. Here they had to find a balance between costs (we wanted a relatively low sales price for families) but robust and attractive, uncommon materials (like wheels, scratch-off dots, lift-off points). They managed the task by using various specialised printers and putting the materials together in their own assembly line.

TigerKids http://www.tigerkids.de/ They had to find ways how to assemble materials which have to function for a three years period of time. Here they used again various specialised printers.

AOK Verlags daily business is the production of all kind of advertising, promotion and display flyers and brochures including mailings. We place a couple of hundred orders each year.

14. **Roehampton University, UK (RoU)** dates back to the 1840s, as an educational institute in London, and became a university in its own right, with four colleges, in 2004. Roehampton University employs 1200 staff, and currently has around 8000 students registered on its programmes at all levels. The research team implicated in Toy Box is formed from members of the Clinical and Health Psychology Research Centre (CHPRC) within the School of Human and Life Sciences of Roehampton University, which focuses on the identification of risk factors for major chronic diseases such as cardiovascular disorders, obesity, and disordered eating to develop strategies for better intervention and prevention. The centre's research activities take place in custom-built laboratories newly equipped for psychophysiological (ECG, EDA, continuous blood pressure, etc.), nutritional (including hidden-balance eating monitors), developmental (including eye-tracking), neuropsychological and cognitive neuroscience (including state-of-the art EEG systems and shared fMRI facilities) methods. Members of the centre also share facilities provided by the School's bioscience and sports physiology laboratories. For field research, the centre has a range of ambulatory testing equipment, and a comprehensive psychological test library.

Main tasks in the Toy Box project

The RoU will participate in reviewing the literature on educational strategies for the formation of desired energy balance related behaviours (WP4) and also provide feedback and guidance for the development of the intervention material in WP6.

Previous experience relevant to those tasks

The team has many years' experience of research into obesity and eating behaviour, including experimental and intervention studies in young children. Relevant expertise includes research into, and treatment of, eating disorders, obesity, binge eating, acquisition of food preferences, psychology of food choice and normal eating behaviour, and physiology of appetite regulation. The team has recently been involved in creating an English version of Tiger Kids, an intervention to promote healthy eating and physical activity in preschool children.

The team members are also experienced researchers in the broad area of health, which includes expertise in the development, maintenance and changing of habitual health behaviours, such as eating, drinking, smoking and physical activity, in both adults and children, as well as understanding the impact of stress and its psychological, emotional and physiological consequences. The team members have been active researchers in these areas for 25 years.

Short profile of team members involved in the project

Leigh Gibson (PhD) is Reader in Biopsychology, Director of the CHPRC, a chartered psychologist at Roehampton University and honorary associate staff member in the Department of Epidemiology and Public Health at UCL. His current research is concerned with influences on appetite and food choice, and their interaction with obesity, stress, health, and cognitive and emotional well-being.

Recent publications relevant to the project

Dovey, T. M., Staples, P. A., Gibson, E. L. & Halford, J. C. G. (2008) Food neophobia and 'picky/fussy' eating in children: A review. Appetite, 50, 181-193.

Brown, K. A., Ogden, J., Vögele, C., & Gibson, E. L. (2008) The role of parental control practices in explaining children's diet and BMI. Appetite, 50, 252-259.

Cooke, L. J., Wardle, J., Gibson, E. L., Sapochnik, M., Sheiham, A., & Lawson, M. (2004) Demographic, familial and trait predictors of fruit and vegetable consumption by pre-school children. Public Health Nutrition, 7, 295-302.

Cooke, L., Wardle, J., & Gibson, E. L. (2003) Relationship between parental report of food neophobia and everyday food consumption in 2-6-year-old children. Appetite, 41, 205-206.

Gibson, E. L. & Wardle, J. (2003) Energy density predicts preferences for fruit and vegetables in 4-year-old children. Appetite, 41, 97-98.

Wardle, J., Sanderson, S., Gibson, E. L., & Rapoport, L. (2001) Factor-analytic structure of food preferences in four-year-old children in the UK. Appetite, 37, 217-223.

Wardle, J., Herrera, M. L., Cooke, L., & Gibson, E. L. (2003) Modifying children's food preferences: the effects of exposure and reward on acceptance of an unfamiliar vegetable. European Journal of Clinical Nutrition, 57, 341-348.

Wardle, J., Cooke, L. J., Gibson, E. L., Sapochnik, M., Sheiham, A., & Lawson, M. (2003) Increasing children's acceptance of vegetables; a randomized trial of parent-led exposure. Appetite, 40, 155-162.

15. The **University of Luxembourg, Luxembourg (ULU):** founded in 2003, the University of Luxembourg is one of the youngest universities in Europe and offers a range of high-quality programmes at all levels. It is multilingual and research-led, with a strong focus on systems biomedicine and related areas from the natural and social sciences. The research team implicated in Toy Box is formed from members of the group in Clinical and Health Psychology (leader: Prof. Claus Vögele, PhD) within the INSIDE Research Centre of the University of Luxemburg, which focuses on the identification of risk factors for major chronic diseases such as cardiovascular disorders, obesity, and disordered eating to develop strategies for better intervention and prevention. The group's research activities take place in custom-built laboratories newly equipped for psychophysiological (ECG, EDA, continuous blood pressure, impedance cardiography etc.), developmental (including eye-tracking), neuropsychological and cognitive neuroscience (including state-of-the art EEG systems) methods. Members of the group also share facilities provided by the University's Neurophysiology laboratories. For field research, the group has a range of ambulatory testing equipment, and a comprehensive psychological test library.

Main tasks in the Toy Box project

The **ULU** will participate in reviewing the literature on psychological approaches explaining young children's acquisition and formation of energy-balance related behaviours, and facilitating their management (WP4) and also provide feedback and guidance for the development of the intervention material in WP6.

Previous experience relevant to those tasks

The team has many years' experience of research into obesity and eating behaviour, including experimental and intervention studies in young children. Relevant expertise includes research into, and treatment of, eating disorders, obesity, binge eating, psychology of food choice and normal eating behaviour, and factors affecting acquisition of physical activity behaviour in children. The team members are also experienced researchers in the broad area of health, which includes expertise in the development, maintenance and changing of habitual health behaviours, such as eating, drinking, smoking and physical activity, in both adults and children, as well as understanding the impact of stress and its psychological, emotional and physiological consequences. The team members have been active researchers in these areas for 25 years.

Short profile of team members involved in the project

Claus Vögele (PhD) Professor of Health Psychology at Luxemburg University and group leader of the team in Clinical and Health Psychology, which focuses on the identification of risk factors for major chronic diseases. He has co-authored several interventions for children to acquire healthy eating and physical activity habits (e.g. Power Kids) and has recently received a grant to create an English version of Tiger Kids.

Recent publications relevant to the project

Azaïs-Braesco, V., Brighenti, F., Paoletti, R., Peracino, A., Scarborough, P., Visioli, F. & Vögele, C. (2009). Healthy food and healthy choices: a new European profile approach. Atherosclerosis Supplements, 10, 1-11.

Brown, K. A., Ogden, J., Vögele, C., & Gibson, E. L. (2008) The role of parental control practices in explaining children's diet and BMI. Appetite, 50, 252-259.

Coppinger, T., Jeanes, Y.M., Dabinett, J., Vögele, C. & Reeves, S. (2010). Physical activity and dietary intake of children aged 9-11 years and the influence of peers on these behaviours: a one-year follow-up. European Journal of Clinical Nutrition, advance online publication 19 May 2010; doi: 10.1038/ejcn.2010.63.

Finnerty, T., Reeves, S., Dabinett, J., Jeanes, Y.M. & Vögele, C. (2010). Effects of peer influence on dietary intake and physical activity in school children. Public Health Nutrition, 13, 376-383.

Hasenböhler, K., Munsch, S., Meyer, A., Käppler, C. & Vögele, C. (2009). Family structure, body mass index and eating behaviour. International Journal of Eating Disorders, 42, 332-338.

Kubiak, T., Vögele, C., Siering, M., Schiel, R. & Weber, H. (2008). Daily hassles and emotional eating in obese adolescents under restricted dietary conditions – the role of ruminative thinking. Appetite, 51, 206-209.

Vögele, C. (2009). The psychology of childhood obesity. Agro Food Industry Hi Tech, 20(5), 34-37.

Vögele, C. & Ellrott, T. (2008). Understanding the decision making process in food choices: reason, drive or learned behaviour? Journal of Clinical Lipidology, 2(55), S36-S37. (Abstract)

B 2.3 Consortium as a whole

Building upon proposed strategies stated in the White paper on Nutrition, Overweight and Obesity related health issues by the European Commission and on the European Charter combating obesity by WHO Europe, the ToyBox project consortium spans the necessary multidisciplinary variety of experts such as public health experts, epidemiologists, nutritionists, physical activity experts, pedagogists, psychologists, behavioural scientists, nutritionists, paediatricians, early childhood psychologists, health economists, totalling 15 partners, from 10 countries. The consortium, consists of 11 universities, 1 research institute, 2 advocacy groups and an SME, and will be coordinated by the HUA, which has experience in coordinating multicenter and multinational research projects and ample experience in EU funded research projects.
Expertise on the various components of eating, physical activity and sedentary behaviour will be provided by Harokopio University in Athens (Greece), VUmc (Netherlands), Ghent University (Belgium), University of Durham (UK), and University of Zaragoza (Spain). Moreover, these partners have ample experience in conducting literature reviews and secondary data analyses.

Moreover, Harokopio University of Athens, Ludwig-Maximilians University of Munich, Ghent University, VUmc, University of Zaragoza and Høgskolen i Oslo og Akershus (HiOA).
are all involved in other EU funded projects on related topics: EARNEST (the early nutrition programming project, contract number FOOD-CT-2005-007036), dealing with the long-term consequences of early nutrition by metabolic programming (www.metabolic-programming.org); HELENA (Healthy Lifestyle in Europe by Nutrition in Adolescence, contract number FOOD-CT-007034), dealing with adolescent nutrition and lifestyle (www.helenastudy.com), IDEFICS (Identification and prevention of Dietary- and lifestyle-induced health Effects In Children and infantS, contract number 016181-2), dealing with the primary prevention of obesity in infants and children (www.idefics.eu), TEENAGE (grant agreement number 2006323), focusing on identification of socioeconomic disparities in health behaviours across Europe, the Pro Children Project (specific RTD programme 'Quality of Life and Management of Living Resources', QLK1-2001-00547), aimed at explaining and promoting fruit and vegetable intake among children (http://www.prochildren.org), HOPE (SP5-CT-2006-044128) creating a network-of-networks to bring European research on obesity prevention together (http://www.hopeproject.eu) and the PROGREENS Project (funded by EC Programme of Community Action in the Field of Public Health 2003-2008) (http://www.progreens.org). All partners are also involved in national longitudinal studies or intervention studies assessing and/or promoting healthy nutrition and physical activity aiming at the prevention of overweight, providing the ToyBox consortium the opportunity to use national level data across Europe as well. This gives the ToyBox consortium access to existing data sets and existing intervention schemes for the different European regions and across Europe.

All partners involved in the various research activities of the ToyBox project have wide experience in the kind of analyses they are responsible for. Every partner has his own specific expertise and discipline. Moreover, partners from the various regions of Europe (South, Eastern, Central and North) are represented, which will lead to unique information and a unique interplay between the different partners and European regions.

Within the Department of Nutrition and Dietetics, Harokopio University in Athens (**HUA**), the research group of "Nutritional Assessment, Health Promotion and Counselling" (led by Dr. Yannis Manios) has been implicated in various multicentre studies funded either by the European Union or other Entities. They have a strong background in conducting epidemiological (GENESIS Study, HELENA, ATTICA Study) and intervention studies in the school setting (Cretan Health and Nutrition Education Programme, HELENA, CHILDREN Study), with a particular focus on child obesity and obesity-related behaviours. Yannis Manios, leading the group was one of the principal investigators for the "Cretan Health and Nutrition Education Programme", which has been acknowledged by international scientific literature and Public Health Authorities as best practice school-based intervention programme. In addition, the group has been awarded with a grant by the Greek Ministry of Education and the National Institute for Youth to develop the educational material

for “Dietary habits and Nutrition” and “Physical Activity and Health” which is currently used as part of the Health Education and Health Promotion activities in all primary schools in Greece. For the past 18 years the group conducts epidemiological and intervention studies at school setting (day-care settings, kindergartens, primary and secondary schools) and has gained important insights on factors affecting the success of school-based interventions; they will therefore lead WP7, being in charge of the implementation and process evaluation of the ToyBox intervention.

The Ludwig- Maximilians University of Munich (**LMU**) has extensive experience working with young children via its coordinating several EU projects such as CHOP, PIANO and EARNEST and as steering committee members in the DG SANCO project Breastfeeding Promotion in Europe. LMU has a strong background in developing intervention programmes for children (PowerKids, more than 60.000 participating children and their families) and behavioural obesity prevention programmes for pre-school children (TigerKids, more than 120.000 participating children and their families). TigerKids has been identified as one of the two model programmes for obesity prevention by the *EU White Paper on A Strategy for Europe on Nutrition, Overweight and Obesity related health issues 2007*[122]. Thus LMU will lead WP6, developing the ToyBox multi-component family-involved intervention programme based on the valuable experience gained during the development, implementation and evaluation of TigerKids programme in Germany. Two other ToyBox partners were involved in the TigerKids project (IFP, AOK-Verlag). IFP is an institute of early childhood education while **AOK-Verlag,** is a SME experienced in producing material for projects addressing children’s behaviours and child obesity. Successful projects in which AOK-Verlag has produced material are “Sanftes Rückentraining” (spine training), Go!Kids (moving activities) both in collaboration with the university of Bayreuth as well as Relax Kids, LegaKids, PowerKids in collaboration with LMU.

The VU University medical centre (**VUmc)** has a strong background in human movement science, nutrition, sports medicine, epidemiological data analysis including systematic reviews and secondary analyses, and in health promotion and have specific experience in developing and evaluating interventions promoting physical activity and/or healthy nutrition in school settings. Therefore, they will lead the WP on identification of important energy balance related behaviours in young children and high risk groups in the population (WP2).

Ghent University (**UGent)** has backgrounds in psychology, sociology, and public health. The Department of Movement of Human Sciences has a special expertise in the field of personal and family related determinants of health behaviours. Prof. Ilse de Bourdeaudhuij will lead the work package on determinants of specific health behaviours with a special focus on family and school environmental factors (WP3). Her ample experience in this field (www.helenastudy.com, www.idefics.eu, www.prochildren.org) and the outcome of WP3 will guide the development of teachers and parents component for the ToyBox material.

The University of Durham (UDUR) have special experience conducting systematic reviews and will lead WP4 including systematic reviews to identify most successful behavioural models (and subsequent methods) used at school setting for promotion of healthy weight and desired energy balance-related behaviours. Roehampton University, UK (RoU) and the University of Luxemburg, Luxemburg (ULU) will also contribute significantly in WP4 with their expertise in experimental and intervention studies in young children. They have a strong background in obesity and eating behaviour, including experimental and intervention studies in young children and acquisition of food preferences, psychology of food choice and normal eating behaviour, physiology of appetite regulation and development, maintenance and changing of habitual health behaviours, such as eating, drinking, and physical activity in both adults and children. Thus they will lead the task on identification of educational strategies used for the formation of desired energy balance related behaviours (WP4) and will also provide feedback and guidance for the development of the ToyBox material for teachers and parents (WP6).

The Unit for Health communication at Høgskolen i Oslo og Akershus **(HiOA)** is new, but the recently appointed Professor Agneta Yngve has built extensive networks on child obesity and health

promotion programmes at a European level from her previous platform Karolinska Institutet, Sweden, where she is still part time employed. She was instrumental in collecting information on prevalence of overweight and obesity in the 53 countries of WHO Europe for the WHO report published in 2007 and she coordinated the Centre for Monitoring of Public Health Nutrition in Europe (EU DG SANCO) and EUNUTNET – The European network for public health nutrition and still coordinates the ProGreens project, fruit and vegetable promotion in ten European countries. She is the editor-in-chief of the journal for Public Health Nutrition, one of the leading journals in the area. The new unit is very much building on the previous work and is building a very strong collaboration connection with the unit at Karolinska Institutet. The PhD students who have previously been involved in policy analysis will also be involved in this project. The new unit therefore has a huge potential as an important and active partner in WP5 and WP10 to make an overview of existing policies at school level an also disseminate the ToyBox findings. Similar will be the role of the Unit of Public Health Nutrition and Food Safety (co-ordinated by Prof. Stefaan De Henauw) within the Department of Public Health in University of Ghent (**UGent**); this unit has already built up substantial experience in the field of human nutrition and public health (cf. www.publichealthnutrition.ugent.be) and has as such been involved in several national and international projects in the field of public health nutrition. At national level, they serve as scientific advisory bodies of the national food consumption surveys for Belgium. The unit is also one of the partners of the Flemish Health and Environment Centre.

UniZar has extensive experience in conducting large scale surveys as part of national or European projects. UNIZAR is co-ordinating HELENA (dealing with adolescent nutrition and lifestyle) (www.helenastudy.com) and participating in IDEFICS (dealing with the primary prevention of obesity in infants and children) (www.idefics.eu) but also they also participate in a Network of Excellence called EURRECA (EURopean RECommendations Aligned, harmonising nutrient recommendations across Europe with special focus on vulnerable groups and consumer understanding, contract number 036196-2), where UNIZAR is the responsible of the research activity on recommendations for children and adolescents (www.eurreca.org). Due to their strong background in the field, UniZar will lead WP8 developing the assessment tools to be used in the evaluation study.

The Eastern European region will be represented by the Children's Memorial Health Institute, Poland (**CMHI**) and Medical University of Varna, Bulgaria (**MUV**). Both partners will also implement and evaluate the ToyBox intervention in their country but will also provide valuable input throughout the development of the intervention, the adaptation of the assessment tools and the execution of the focus groups. Both partners are involved in other European studies (CHOP, EARNEST, NUTRIMENTHE, In-Form) and have experience in conducting surveys and applying qualitative research methods. Within the **UGent** Department of Public Health, Prof. Professor Lieven Annemans is an expert with regard to the use of health economic modelling, and will therefore lead WP9 and make cost estimates and estimates for cost-effectiveness of the ToyBox intervention.

The ToyBox consortium not only consists of University related institutes but also includes other organisations, IASO, NIGZ and IFP. **IASO** is the main independent coordinating and advocacy organisation for obesity prevention research and action, and is an important network of expertise and advice, providing assistance to the World Health Organisation and aiding the launch of the DG Sanco European Platform on Diet, Physical Activity and Health. Its member organization, the European Association for the Study of Obesity, organizes the annual European Congress on Obesity and consists of a scientific network of national associations for the study of obesity. This network is crucial for advising the ToyBox project research activities and for disseminating the project's results to research association throughout Europe. **NIGZ** has been actively involved in the European Network for Health Promoting Schools since 1992 coordinated by the WHO Europe in Copenhagen. Since 2007 NIGZ runs the secretariat of the network which now has a new name: *Schools for Health in Europe network*, or SHE network. NIGZ runs the annual Go for health campaign for primary schools as an agenda-setting activity. Since 2008 NIGZ acts as a *WHO collaborating centre for school health promotion*. The **IFP** is the Bavarian State Institute of Early

Childhood Research and was responsible for developing the Bavarian Early Education Curriculum which is a mandatory framework for all kindergartens in Bavaria. Healthy behaviour and healthy food choices as well as physical activity are central aims of this curriculum. The IFP was involved in the development of the TigerKids behavioural obesity prevention programmes for pre-school children (more than 120,000 participating children and their families) which has now expanded throughout Germany. Using their wide network of national NGOs and policymakers, they will be very valuable for the project regarding the dissemination activities. In addition, the **IASO** and **NIGZ** have a good overview of ongoing European projects and can ensure that the current project is complementary to other research projects already taking place and importantly, other projects that will arise during the course of this project.

Major advantages of this consortium as a whole
The consortium aims to systematically develop and evaluate a school based intervention including a strong family component to promote healthy weight and increase healthy behaviours in young children. This consortium is extremely suited to bring this project to a successful end because:
1) the consortium consists of the most important experts in the field of overweight, energy balance-related behaviours, determinant analyses, developing and evaluating interventions. Firstly, this ensures accesses to data sets for secondary data analyses, and expert development and conduction of survey and intervention research. Moreover, as demonstrated above, the tasks are equally divided between the partners, and they will lead the WP in their specific expertise field. Therefore, the ToyBox consortium will be an optimal consortium to develop an evidence-based intervention based on knowledge from different disciplines.
2) The different institutes and organisations within the ToyBox consortium represent all regions across Europe and organisations that have an inherent international focus. This not only gives the opportunity to discuss and integrate different perspectives, and to create output that is tested and can be implemented and disseminated across Europe.
3) There is an excellent balance between research groups and partners that contribute to dissemination. Dissemination is a critical factor for successful future implementation of the research results on a large scale. The consortium therefore includes organisations with a wide network of national and international health organizations, obesity associations, school network but also of policy makers. These organisations will contribute to a successful dissemination of results. Since the ToyBox consortium includes all relevant multidisciplinary expertise in the field, we ensure that the project is unique and of the highest standards.

B 2.3.1 Sub-contracting

For several expert tasks the ToyBox consortium will subcontract SMEs to ensure good quality of the work. The tasks and SMEs will be described in this section. The sub-contractors will be chosen according to the legal procedures of the partner who will sub-contract the relevant task.

SME for the development of the website
HUA will sub-contract an SME for the development of the multifunctional website. The website will be developed with functionalities for document exchange, consortium and WP-specific email lists, web-based conferencing and data exchange. It will consist of two parts: a) Public internet access for general public, journalists, scientific experts and b) Username and password restricted to the consortium members. The Public internet access will contain all information related to the project: context, partners, information of coming events, areas dedicated for parents and children. The Restricted area shall also contain: a) Forum in which users can discuss project related issues and upload documents (on their own). b) an area for the uploading of meeting meetings and related project information. This will be updated only by the Coordinator. The main page will be in English but there will be translated versions (in six other languages). Main graphics and colour combos for the website will be decided along with the partner who is in charge of the graphics of the Intervention material and the coordinator (HUA). A first version of the website should be working in June-July 2010. The available budget for this sub-contracting is 25.000€

SMEs for the production and delivery of the intervention materials
AOK Verlag will subcontract an SME to produce the hand puppet, ToyBox and wooden train for exploration of food for 6 countries, 20 pre-primary schools per country and 2 classes per school and another SME to deliver all intervention material from Germany to the rest of the participating countries. The available budget for this sub-contracting is 51.280€.

SME for the translation, adaptation, elaboration and printing of the intervention material and questionnaires from the English version into Polish.
CMHI will subcontract a SME to translate and adapt the intervention material and questionnaires from English into Polish, but also for the elaboration of the English versions of the texts and adaptation to Polish, incorporation of the corrections suggested by the other partners involved, analysis of specific requirements in Polish kindergartens and regulations that should be considered in the Polish versions of intervention materials and finally for printing the questionnaires and other intervention material. The available budget for this sub-contracting is 3.700€.

B 2.4 Resources to be committed

The main objective of the ToyBox project is to develop and evaluate a new intervention promoting healthy weight and energy balance related behaviours in young children. The budget is mainly devoted to the required personnel costs necessary to run the coordination, research activities and dissemination of the findings. Other costs are devoted to execution of focus groups, evaluation studies, validation studies, dissemination and communication. Table 2.4.a gives an overview of the total costs by WP by partner, separately for direct costs (personnel), other direct costs (consumables, equipment, travel) and indirect costs (overhead).
Coordinating such a project requires a good infrastructure and communication system to exchange knowledge and findings between the partners. These costs are mainly covered in WP 1 and WP10 by developing a website. In addition, 6 meetings for WP leaders plus invited WP participants will be organised by different WP leaders at the time relevant for their tasks (travel costs) while 2 workshops will be organized to train researchers in methodologies and procedures to be used in ToyBox. Costs for acquiring the rights for and access to the existing data sets (including cleaning these data, potential travel to the research centre owning the data or subcontracting the owner) are included in WP1. Though these costs are mentioned in WP1 for clarity, they have been calculated as a research activity (75% contribution EU), as strictly this activity is part of the research activities of WP2, but was included here for economy of scale. Estimated cost for materials and equipment vary widely across the different research activities as can be seen in Table 2.4.b. Reviewing the literature and conducting secondary data analyses. Conducting the focus groups and developing the assessment tools mainly requires personnel costs and travel expenses for training workshops. Another major component of the proposed project is the actual development of intervention materials. The production of materials will be subcontracted (WP6). Since implementation of the intervention will be done by teachers, the major cost drivers for the evaluation of the study will include personnel costs for the pre- and post- intervention measurements and resources for the equipment (i.e. pedometers, scales, stadiometer) and printing questionnaires. In WP10, a final dissemination meeting inviting representatives of NGOs and important stakeholders will be organised. Also costs related to preparation of the final intervention package are included, such as printing the materials, training the teachers, organize activities. And finally, costs are estimated for the dissemination activities in WP10.

	GR	GR	D	B	NL	E	S	UK
	HUA MGT	HUA	LMU	Ugent	Vumc	UniZar	**HiOA**	UDUR
Person Months	26	55	62	82	23	53	15	12
Personnel Cost	117.000	247.500	310.000	410.000	115.000	238.500	82.500	66.000
Consumables & equipment & travel	15.000	46.500	11.000	16.500	7.500	10.000	5.000	9.500
Cost system	STFR	STFR	STFR	STFR	STFR	STFR	STFR	STFR
Indirect Costs	79.200	176.400	192.600	255.900	73.500	149.100	52.500	45.300
Subcontracting	25.000							
**Total Budget**	236.200	470.400	513.600	682.400	196.000	397.600	140.000	120.800
EU Contribution	100%	75%	75%	75%	75%	75%	75%	75%
**Requested Budget**	236.200	352.800	385.200	511.800	147.000	298.200	105.000	90.600

	D	PL	BG	UK	NL	D	UK	LU
	IFP	CMHI	MUV	IASO	NIGZ	AOK-Verlag	RoU	ULU
Person Months	15	37	37	12	22	20	4.5	2.5
Personnel Cost	75.000	106.600	111.000	66.000	110.000	151.280	24.750	13.750
Consumables & equipment & travel	5.500	15.000	13.000	50.000	7.000	7.000	4.500	2.000
Cost system	STFR	STFR	STFR	STFR	STFR	SFR	STFR	STFR
Indirect Costs	48.300	72.960	74.400	69.600	70.200	21.400	17.550	9.450
Subcontracting		3.700				51.280		
**Total Budget**	128.800	198.260	198.400	185.600	187.200	179.680	46.800	25.200
EU Contribution	75%	75%	75%	75%	75%	75%	75%	75%
**Requested Budget**	96600	148695	148800	139200	140400	134760	35100	18900

**Total project FP7 ToyBox**	
Personnel	2.273.580
Materials	225.000
Indirect cost	1.408.360
Subcontracting	76.280
**Total Project**	**3.906.940**
Total 75% EU Contribution	2.753.055
Total 100% EU Contribution	236.200
**EU Contribution**	**2.989.255**

B3. Impact

B 3.1 Strategic impact

B 3.1.1Expected impact for the overall AREA 2.2.1 Consumers

The overall aim of this thematic area is to increase our knowledge and understanding on consumers' food choices and eating habits which has become a major area of competitiveness for the food industry. As an outcome, food production, advertising and marketing as well as policies, legislation and regulations regarding these issues at a local, national and European level are becoming important influencing factors regarding consumers' food choices and eating habits.

This in turn seems to have a significant role in population's health indices since most of the leading causes of morbidity and mortality such as obesity and obesity-related diseases in Europe are partially related to food intake and food habits. However this trend does not seem to influence the overall population in the same way but certain subgroups appear to be more vulnerable. For these reasons, recording food intake as well as its determinants is of primary importance; by further identifying the most vulnerable groups in the population and understanding the reasons underlying their food choices on a European scale level is an important first step in developing Public Health Policies. This could effectively tackle this issue minimising social inequality and promoting health and well being for the overall population.

However prevention is the best treatment and thus early preventive measures should be the primary aim of Public Health Policy. This is particularly true for obesity and obesity related degenerative diseases. As these are related to the establishment of unfavourable eating habits, preventive measures early in life seem to be a reasonable counteractive measure to prevent these habits before they are already formed and established.

The first part of the ToyBox project is in line with the above issues raised by the overall thematic area. The first WPs are aiming to record food intake and eating habits related to obesity in early childhood as well as identifying the high risk groups on a European scale. Eating habits are beyond just food intake- they include the wider contexts of eating such as parallel activities (e.g. TV watching), social eating (e.g. party food), number and energy content of meals.

In early childhood, both food intake and eating habits are determined by children's social (parents, teachers, peers) and physical environment (e.g. available food at home, food services at school, food promotion and advertising). As young children have not developed a cognitive way of eating, their behaviours regarding eating are affected by some congenital factors such as neophobia but are most heavily determined by social and environmental cues, such as role modelling, peer influences, availability and accessibility to food. Therefore, any attempt for early prevention should target not only children but should primarily focus on affecting their social and physical environment to become more supportive in adopting healthy eating behaviours. Some potential parameters that could be addressed regarding -both social and physical- home environment are existing parental eating habits, parental perceptions, knowledge, beliefs and attitudes regarding food and health but also availability of healthy food options at home. Similarly, at school setting, obesity prevention attempts should take into account that school staff perceptions, beliefs and attitudes (social environment) but also school setting facilities and food services or food promotion at school (physical environment) are important influencing factors for children's behaviours.

The ToyBox will proceed with extensive reviews of the literature and thorough secondary analyses on energy balance related behaviours **(WP2)** and new behavioural research to identify the determinants of these behaviours **(WP3).** Through further systematic reviews on effective behavioural models and intervention strategies promoting healthy energy balance but also

educational strategies for the formation of healthy energy balance behaviours **(WP4),** the ToyBox consortium will gain insights on the above parameters and develop effective counter measures to tackle the childhood obesity epidemic (**WP6**). Important school-related contextual, regulatory and policy factors (e.g. time, space, marketing and advertising of food, food services and food promotion at the school setting) will also be taken into account for the development of the ToyBox intervention programme to increase its potential for sustainment **(WP5).**

However since achieving energy balance requires two “players” (i.e. energy intake and energy expenditure), food intake should not be the only target when aiming to promote healthy weight; any attempt failing to address both eating and physical activity-related behaviours would have minimal odds for being successful.

This is very clearly stated in the call **KBBE-2009-2-1-03 - Behavioural models for prevention of obesity, with a particular focus on children** where the need to understand why people eat the foods they eat or why they do (or do not) participate in physical activity is highlighted. The ToyBox intervention will attempt an evidence-based holistic overview of the early childhood obesity problem and will aim to promote healthy weight by addressing eating, sedentary and physical activity behaviours in young children.

B 3.1.2 Expected impact for the call KBBE-2009-2-1-03 -Behavioural models for prevention of obesity, with a particular focus on children

In line with what is stated in the relevant section of the call, the expected impacts of the ToyBox project are the following:

<u>1st Expected Impact</u>
Based on pooled experience at a local level, new behavioural models and intervention strategies that could be applied on a European scale will be developed to prevent childhood obesity;

<u>2nd Expected Impact</u>
These new behavioural models and intervention strategies should be implemented and evaluated at a European level with the aim of influencing behaviour, particularly of children and preventing childhood obesity. This attempt should be achieved by increased collaboration between different fields of science.

<u>3rd Expected Impact</u>
Contribute to the activities of the EU Platform on Diet, Physical Activity and Health and support Public Health Policy in preventing obesity

<u>The **ToyBox** will achieve the **1st Expected Impact** in the following ways:</u>

1a. Advance the state-of-art on obesity in early childhood by developing insights on important energy-balance related behaviours and their determinants. The geographical as well as socioeconomic distribution of obesity and childhood obesity highlight the need to take into consideration these two important parameters. Existing cohorts from the south of Europe with high prevalence of childhood obesity (Greece and Spain) as well as Eastern Europe (Bulgaria and Poland) and Central Europe (Belgium and Germany) will be used for secondary analyses. ToyBox will move beyond energy equilibrium (energy intake vs. energy expenditure), and will include in these analyses the most important energy balance related behaviours with regard to physical activity, sedentary activities and food intake. Behaviours may be gender-, age- or country-specific or may be related to family demographics and SES level. Thus the ToyBox will run these analyses ensuring that all country-, cultural- and SES-specific differences are addressed (**WP2**).

1b. Explore the determinants of obesity in young childhood by conducting new behavioural research. Due to young children's developmental immaturity and since cognitive decision making has not yet been developed their habits are predominantly determined by their close social (parents/guardians, school staff) and physical environment (home and school setting). Thus, within ToyBox, focus groups interviews will be executed in the selected countries (Belgium, Bulgaria, Germany, Greece, Poland and Spain) with parents/ guardians and school teachers ensuring that identified risk groups will also be included (**WP3**).

1c. However pooling experience at a local level regarding the behaviours related to early childhood obesity as well as their determinants is not enough in designing more effective programmes to combat obesity. Identifying the roots of the problem may be the first step but ToyBox will move further by also trying to understand how it could be tackled in the most efficient way. By extensive reviewing and critically appraising literature, ToyBox will identify effective behavioural models and educational strategies to promote the formation and adoption of desired energy balance related behaviours in this young age and their families (**WP4**).

1d. The ToyBox intervention will be applied at school setting since it provides access to large cohorts of children and their families without requiring additional resources on infrastructure or personnel. However schools function under a given contextual framework and legislations which varies at a local and national level. A report by the European Network of Health Promoting Schools have identified similar barriers potentially explaining the insufficiency of such initiatives in primary schools such as lack of: curriculum time, efficiency, reliable process evaluation, skills and know-how, material and training and monitoring system. Last but not least as reported in this report the lack of the fast and visible changes and health benefits has led to the limited interest from the Ministries of Education and Health [139]. ToyBox understanding the importance of these factors for the success of the intervention, will explore the most important contextual factors and stakeholders affecting the success of school-based intervention programmes to ensure alliances and synergies and make recommendations for public policy makers (**WP5**).

<u>The **ToyBox** will achieve the **2nd Expected Impact** in the following ways:</u>

Based on insights gained at local level (1a-1c) but also examining the contextual framework at schools (1d), a new "behavioural intervention" aiming to contribute in combating childhood obesity at a European scale will be developed. The ToyBox project will develop a multi component school based and family involved intervention, aiming to facilitate local needs within a European scale approach. The content of the intervention will be developed based on the findings obtained at local level integrated with the most suitable behavioural models and educational strategies. For the identification of the behaviours as well as the development of the intervention material, scientists from different fields will contribute with their expertise aiming to tackle in the most effective way the multifactorial problem of childhood obesity. Public health experts, psychologists and behavioural scientists, medical doctors, dieticians and nutritionists, pedagogists specialized in early education, physical education and sports sciences specialists and exercise physiologists, health economists and art designers and an SME with previous experience in developing such material (Relax Kids, LegaKids, PowerKids and TigerKids) will contribute in the development, implementation and evaluation of process, impact, outcome and cost-effectiveness. The ToyBox will consist of a Teachers' General Guide, a Classroom Activities Guide and a Parental Activities Guide.

2a. The ToyBox Teachers' General Guide will provide teachers with some basic knowledge on food groups, dietary recommendations, physical activity recommendations, etc. Furthermore this guide will improve teachers' skills and self-efficacy regarding these matters. It will also provide some information on the scope of the project as well as a brief friendly- to-read report on the identified behaviours and sub-behaviours primarily related to obesity (as identified in WP2). Based on the report by the European Network of Health Promoting Schools such knowledge should not be considered as granted and should be provided to teachers[139].

2b. The ToyBox Classroom Activities Guide will provide a simple to use practical guide with specific activities to be executed in the school setting related to food intake and physical activity. It will also contain a manual on how to implement these activities using the most effective and appropriate behavioural models, intervention and educational strategies. Since children of this age have not yet developed the cognitive decision making procedure, emphasis will be given in developing a school environment and activities to support this.

2c. The ToyBox Parental Activities Guide will primarily address and aim to tackle the determinants of behaviours (e.g. parental behaviours, knowledge, beliefs, attitudes) identified locally (WP3) in the six countries to be primarily related with children's obesogenic behaviours. ToyBox will include and address all cultural and sociodemographic specific determinants in the parental component of the material to ensure programme's applicability at a European scale.

The **ToyBox** will achieve the **3rd Expected Impact** in the following ways:

The ToyBox as briefly presented above is in line with the scopes of the White Paper on Nutrition, Overweight and Obesity and contributes to the activities of the EU Platform on Diet, Physical Activity and Health. Furthermore, the work undertaken in the ToyBox WPs and the results of the programme (described above) will significantly contribute to the activities of these two initiatives.

Specifically, the White Paper states: *"Any public action, including those possibly undertaken at Community level, in this field should take into account three factors. Firstly, the individual is ultimately responsible for his lifestyle, and that of his children, while recognising the importance and the influence of the environment on his behaviour. Secondly, only a well-informed consumer is able to make rational decisions. Finally, an optimal response in this field will be achieved by promoting both the complementarity and integration of the different relevant policy areas (horizontal approach), and of the different levels of action (vertical approach)."*

The EU Platform on Diet, Physical Activity and Health mentioned some fields of action to improve nutrition and health related issues, which the White Paper further particularized to outline specific actions that can be taken at EU level. The ***actions identified are***: having better informed consumers; making the healthy option available; encouraging physical activity; addressing priority groups and settings; developing the evidence base to support policy making; and developing monitoring systems. The **ToyBox** -fully aligned with the above statement and the proposed actions- addresses all the actions identified above. Specifically it recognizes the importance of the children's physical environment in developing their behaviours (diet and physical activity) and tries to develop counteractions to improve it. It aims to educate parents (consumers) in order to be able to make the best choices for them and their children. It addresses groups and settings prioritizing children and wide Public Health approaches via school setting, develops monitoring system to assess process, impact, outcome and cost-effectiveness and develop evidence base outcomes and support decision making in European Public Health Policy.

It is however important to undertake such actions by following specific planned strategies. The four fundamental aspects to the actions outlined by the White Paper to achieve this are noted below in *italics*. ToyBox addresses all of them through a detailed work plan broken down into 10 Work Packages (including consortium management and assessment of progress and results).

3a. ***Firstly***, *actions should aim to address the root causes of the health related risks. In this way, the actions set out in the strategy should contribute to reducing all risks associated with poor diet and limited physical activity including, but not limited to, that associated with excess weight.*

The ToyBox, fully aligned with this statement, aims to record the behaviours and their determinants and use this knowledge to develop intervention strategies to reduce this risk. *Priority groups and settings* are central to the ToyBox project which addresses young children from South, East and Central-North Europe; emphasis will be given to low SES groups and risk groups for high childhood obesity prevalence. ToyBox will aim to develop procedures to

promote an environment which will be supportive to children's desired eating and activity behaviours within school setting and home.

3b. ***Secondly**, the actions described are intended to work across government policy areas and at different levels of government using a range of instruments including legislation, networking, public-private approaches, and to engage the private sector and civil society.*

Fully aligned with the second aspect also, ToyBox will involve and engage relevant proximal (parents, teachers) and distal (board of education, ministries, NGOs) stakeholders with regard to the promotion of healthy weight and formation of healthy energy balance related behaviours in young children. The results of ToyBox will be translated in practical strategies and knowledge that can be used by schools, policymakers, health professionals and the general public. Thus, the ToyBox project clearly constitutes a **translational research project**.

3c. ***Thirdly**, for the sake of efficiency, the strategy will require action from a wide range of private actors, such as the food industry and civil society, and actors at local level, such as schools and community organisations.*

The ToyBox consortium consists not only of research institutes, but also includes advocacy and policy oriented organisations and SMEs. These SMEs will contribute valuable expertise to ensure translation of the research in an intervention that is suitable for the general public.
The Ludwig-Maximilian University of Munich (LMU), the Bavarian State Institute for Early Childhood Research (IFP) as well as a SME Publishing Company (AOK-Verlag) will be responsible for the development of the ToyBox intervention.
In addition, ToyBox will involve and engage schools and parent-teacher organizations to address actors at local level. The collaboration of academia, state institutes and private sector for the development of the intervention material will improve efficiency and the alliance with local actors will accentuate ToyBox as an exemplary model of intervention strategy.
Furthermore, during the dissemination phase two policy oriented organizations participating in the ToyBox consortium (IASO and NIGZ) will promote the material of the ToyBox programme and the key findings of the study in their networks, schools, Ministries, parent-teacher organizations and policy makers. Effective partnerships will be developed at a European level and local networks for action will be strengthened. ToyBox will be developed based on the input that will be obtained at a local level from civil society and stakeholders in the school setting; its success will be heavily influenced by stakeholders' involvement and engagement in this field and as such ToyBox has to be integrated and serve local needs.

3d. ***Finally**, and perhaps most importantly, monitoring will be essential over the coming years. The number of activities aimed at improving diet and physical activity is already extensive and is growing year by year. There is often little or no monitoring taking place, resulting in limited assessment of what is working well, or whether actions need to be refined or fundamentally changed.*

The ToyBox will provide a European approach which will be flexible enough to accommodate cultural differences as well as differences in local policies. *Interventions at these levels are vital to tailor design and validated general approaches to specific local contexts. These actions also need to be monitored, evaluated and discussed* (White Paper on Nutrition, Overweight and Obesity). This is another innovative aspect of ToyBox and adds to its strength. Within ToyBox, process measures will be evaluated during the intervention to identify potential barriers and weak points and inform for further corrective actions. Their friendly-to-use form will make them suitable for use if the programme is sustained at school-setting. The outcome and impact evaluation tools will also be easy to use to monitor the effectiveness of the activities while the ToyBox results will support decision making for Public Health Policy.
Finally, a network-of-networks list will be created and maintained by IASO to gather all actors across the Europe contributing to the achievement of the objectives established by the White Paper on Nutrition, Overweight and Obesity [122]

B 3.1.3 A Partnership Approach at a European level

As stated in call KBBE-2009-2-1-03-Behavioural models for prevention of obesity, with a particular focus on children, ToyBox aims at developing an intervention that can be implemented in different settings throughout Europe. In order to be able to do this, great care is taken to include partners representing all regions of Europe. This is essential to actually tailor the intervention to the regions cultural and social features, and make it more successful for wider implementation.
The ToyBox consortium consists of the top European scientists in this multidisciplinary research field and the major European and world-wide task forces that are concerned with dealing with obesity prevention, including linkage groups that will promote local implementation. The research activities undertaken will take into account and connect the current research efforts and therefore create a synergy that will be of considerable additive value.
The ToyBox consortium focuses at the development of an intervention that can be implemented in different regions across Europe but will expand dissemination of the results to the wider international research and policy communities through its involvement in the crucial scientific meetings on obesity prevention, through publications in international scientific journals and through the ToyBox website communications. This to promote large scale implementation of the results also in non-European countries in order to contribute to curbing the global obesity epidemic.

B 3.1.4 Feasibility of expected impacts

Achievement of all impacts will of course depend on external factors and rely on assumptions. Some of the main external factors will be:
* *school attendance.*
In Europe, different legislation applies regarding pre-primary education in different countries and school attendance is not compulsory for all children aged 4-6 years old. However the majority of children of this age (95-100%) attend kindergartens (www.eurydice.org). Therefore school is an ideal arena to implement an intervention promoting healthy energy balance health behaviours;
* *school curriculum*
In primary education, the overloaded curriculum often does not allow much space for health promotion activities to integrate in the daily programme. However in pre-primary education where ToyBox is targeting, curriculum is more flexible and mainly aims to supply children with skills and construction of knowledge. Health promotion activities seem to share these aims and thus can more easily become incorporated. The ToyBox intervention will for the larger part be implemented at schools and has to be incorporated in the existing school curricula and/or in the school environment. Of course, in the development procedure of the intervention, we will consider all possibilities and will connect as closely to the school curricula, and combine parental activities with other parental activities, such as parents' evenings. School staff, parents and young children will be closely involved in all phases of the intervention development and pre-testing so that local implementation possibilities will be taken into account.
* *motivation of the school teachers*
From previous experiences it is known that the implementation rate depend largely on the motivation the people responsible for the implementation, in this case the teachers and the school boards. The ToyBox consortium has ample experience in working with school teachers to develop intervention schemes that do link to their way of working and that are fitting in their work routines. Furthermore, the ToyBox project will provide training and easy to use material to increase teachers' skills and self-efficacy and motivate them towards programme's activities.
* *motivation and health illiteracy of parents*
Likewise, the involvement of the parents in the various activities largely depends not only on their motivation to prevent overweight of their child and to improve health behaviours of their child, but also on their knowledge and insights into the necessity of involvement in school-based health promotion activities, and on their time restraints linked to other, especially work-related obligations. Their motivation and ability to be involved in school-based health promotion is linked to their knowledge and health illiteracy. Since parental involvement is of key importance for promoting health behaviours in young children, parents will be addressed in the intervention scheme and will

be motivated to be actively involved in some of the ToyBox intervention activities. Potential involvement activities will include participation in parents' evenings as well as receiving fact sheets and periodical newsletters, which include illustrations and use simple language.
* *mass media campaigns*
Mass media campaigns and other initiatives that start during the implementation of the ToyBox intervention, may influence the impact of the intervention in various ways. It may affect the knowledge related to adverse health effects of overweight, recommendations for fruit and vegetable intake, fat intake, etc and therefore motivate people to change their behaviours. However, since the ToyBox intervention scheme will be validated in a randomized school-based intervention trial, such potential bias from mass media intervention activities will be equal in intervention and control groups.
* *national policy initiatives*
Local legislation might influence school policies, for instance by banning vending machines in primary schools, or banning snack bars in the close vicinity of schools, introducing school meals. These legislations might differ by country, or even by city. We know that in the several European countries (e.g. Greece, Netherlands) no meals are typically offered in kindergartens, and that, for example, in the UK school meals are offered and have to meet certain minimum standards. Such school environmental factors will affect the nutritional intake of the children, and the ToyBox intervention will leave room for local adaptations.
* *access to internet*
From previous experiences access to the internet at schools and at home may vary substantially both within countries but also across Europe. In ToyBox, an internet-based intervention component will be available only for teachers of intervention schools to avoid contamination with the control group. However after the completion of the intervention, ToyBox material will be available to general public including parents and teachers to increase dissemination. Still, since computer illiteracy in certain subgroups or regions of Europe may prohibit access to these electronic files, this material will be printable so as teachers can print them in the school setting and provide them to those not having access to it.

B 3.2 Plan for the use and dissemination of foreground

ToyBox proposes to conduct innovative state-of-the-art applied research to directly inform policy and practice regarding obesity prevention efforts among young children. Because of the direct applicability of the project's results, dissemination to the scientific community and to policy makers and health promotion professionals is equally important. To give this task the necessary attention, the ToyBox project has dedicated a specific WP to dissemination.

B 3.2.1. Dissemination to scientific community and health professionals

The ToyBox consortium members are well-embedded in the international Public Health research community in all relevant disciplines, including epidemiology, human nutrition and movement sciences, health promotion, pediatrics, psychology, sociology and health economics. The consortium will disseminate the results of the project through existing channels:

1) publication in the most relevant peer-reviewed scientific journals, in the field of obesity (e.g. International Journal of Obesity, Obesity Research, Obesity Reviews, International Journal of Pediatric Obesity), public health (Preventive Medicine, Annals of behavioral Medicine, European Journal of Public Health, Public Health Nutrition), and nutrition and physical activity (British Journal of Nutrition, Journal of Nutrition Education and Behavior, International Journal of Behavioral Nutrition and Physical Activity, Appetite). In our scientific publication policy we will include open access journals to ensure the widest dissemination of peer-reviewed published results among relevant scientists.
2) international scientific conferences, such as the annual meetings of the European Public Health Association, European and International Associations of the Study of Obesity, International Society for Behavioral Nutrition and Physical Activity. Partners in the ToyBox consortium are involved as board members or otherwise in the societies organising these meetings.

3) dedicated symposium: The ToyBox consortium will organise a specific ToyBox symposium at the European Congress on Obesity (2011 or 12).
4) research consortia and scientific organisations. Most members take part in other collaborative projects at the EU levels, such as Idefics (Contract no.: 016181-2), Helena (FOOD-CT-007034), Eurocadet (SP23-CT-2005-006528), HOPE (SP5-CT-2006-044128). The HOPE consortium is specifically dedicated to creating a network of networks of European efforts in curbing the obesity epidemic, and the ToyBox project will join the HOPE Network to fill the gap of systematic intervention development that is clearly present in the HOPE project. The ToyBox partners are also active members in international research consortia including Europe and beyond. One of ToyBox's partners, IASO, is a leading international organization for compiling and transferring scientific knowledge on obesity-related issues within the scientific community within Europe and world-wide. Furthermore, members of the ToyBox consortium are members of the boards of the leading international society in this research area, the International Society of Behavioural Nutrition and Physical Activity (ISBNPA) and the European Childhood Obesity Taskforce. They will disseminate the ToyBox results to these communities.
5) training of young researchers and health professionals. A number of partners are involved in training activities of doctors, nutritionists, movement scientists and other public health professionals. Most members are employed at Universities and have educational tasks in the curricula for medical students, nutrition students and/or students in public health research. The results of ToyBox will be incorporated in student training activities where appropriate. This provides excellent opportunity to share the latest insights with the next generation of researchers.
6) web site: one of the first tasks of WP1 responsible for the coordination of ToyBox will be to build a web environment to communicate the research methods and results within the consortium and to the larger scientific, policy makers and public health professional communities outside the consortium. For this purpose a website will be designed with a restricted area to transfer and discuss preliminary methods and results within the ToyBox consortium, and a public access area with a science community hyperlink to pages where all agreed upon methods and quality approved results will be accessible to the general scientific community.

Several ToyBox consortium members have access to these dissemination channels. For instance, Professors Johannes Brug, Carolyn Summerbell, Agneta Yngve and Ilse de Bourdeaudhuij are members of editorial boards, associate editors or editors- in-chief of key scientific journals in the field, including the International Journal of Obesity, the Journal of Human Nutrition and Dietetics, the International Journal of Paediatric Obesity, Public Health Nutrition, the Journal of Nutrition Education and Behaviour, and the International Journal of Behavioural Nutrition and Physical Activity. Professors Johannes Brug and Ilse de Bourdeaudhuij are the current president and board member of the International Society of Behavioral Nutrition and Physical Activity (ISBNPA) that organises annual conferences on these key health behaviours. Professor Luis Moreno is the representative of ESPGHAN at EU platform level and a member of the European Childhood Obesity Task Force of the European Association for the Study of Obesity, and member of the European Society for Paediatric Gastroenterology, Hepatology and Nutrition (ESPGHAN) Committee of Nutrition.

B 3.2.2. Dissemination to policy makers, caretakers, education professionals and the general public

Whether the designed interventions to induce a healthier life style among the European children will be successful depends largely on the health perceptions, attitudes and practices of the main stakeholders: European citizens, policy makers, school staff and management and health professionals. Dissemination of the results of this project to the larger general public is therefore needed, and we will provide tailored information to the different stakeholder groups via different channels, in order to positively influence their knowledge and attitudes:

1) website: the website includes a ´general public´ hyperlink to a series of pages and downloads within the open access part, where we will publish the project goals, results and implications in lay people's language in the languages of the participating countries. In the ToyBox website we

will also include an ´information for schools´ hyperlink to pages and downloads where all assessment instruments, the description of the intervention package with implementation protocol, and all materials in electronic form or description will be accessible for school staff. Finally, the website will include a 'policy makers and health professionals' hyperlink to pages and downloads with information on key recommendations based on the ToyBox results.

2) Written and broadcasting media: all partners will apply an active strategy to approach the popular press to cover results of the ToyBox project. This process will be guided by participants in WP10. During previous research projects most members of the ToyBox consortium have experience in publishing articles in local news papers
3) Meeting: to the end of the project a dissemination meeting will be organized where the ToyBox consortium will present its results and recommendations to key policy makers in school-based health promotion and obesity prevention, including representatives of the European Commission, WHO, and the Schools for Health in Europe (SHE) Network.
4) Networks: each of the partners will further use their existing contacts and networks with their national and regional policy makers to inform them about ToyBox results and recommendations. Several of the ToyBox consortium members belong to national advisory boards: Johannes Brug served on the Netherlands Health Council Committee on Obesity and on the Committee on health promotion campaigns; Lea Maes is Chair of the commission of school health promotion of the Flemish Educational Council, vice president of the Flemish Health Promotion; Stefaan De Henauw is a member of the National Health Council and of the Scientific Committee of the National Food Safety Authority in Belgium; Lieven Annemans is Past-President of the Intern. Society for PharmacoEconomics and Outcomes Research, and chairman of the Flemish Health Council; the NIGZ is a member of the International Union for Health Promotion and Education (IUHPE), member of EuroHealthNet, Coordinator of the Schools for Health in Europe Network; Agneta Yngve is the Co-ordinating Centre for Monitoring of Public Health Nutrition in Europe (EU DG SANCO) and project co-ordinator of the EUNUTNET – The European network for public health nutrition.

Within the ToyBox consortium different partners and participants have extensive experience with scientific knowledge and recommendation transfer to policy makers and practitioners on an international level. IASO, NIGZ and **HiOA**will be the main partners that ensure a timely and tailored transfer. E.g. IASO has a direct link to the European Platform on Diet, Physical Activity and Health.

B 3.2.3. Exploitation

The leader and participants in WP10 taking care of the large scale implementation and dissemination will develop implementation protocols and disseminate this to local policymakers, schools and other stakeholders. They will develop a strategy to instruct the potential users and implementers of the ToyBox interventions and provide a handbook (in all relevant languages), so that the intervention can easily be implemented across Europe. As mentioned before, several consortium members are involved in training of health professionals and can train professions in implementing the intervention.

B 3.2.4. Management of intellectual property and innovations

The present project will generate scientific knowledge that will be translated into publications in peer-reviewed journals and innovative intervention materials and instruments. Intellectual property and authorship of publications generated by the project will follow strict rules, based on the international standards outlined in the Uniform Requirements for manuscripts Submitted to Biomedical Journals (last version updated October 2008, available at www.icmje.org). This document outlines the basis procedures for authorship and intellectual property for scientific publications in the medical field. In short, authorship will be credited to participants based on 1) substantial contributions to conception and design, or acquisition of data, or analysis and interpretation of data; 2) drafting the article or revising it critically for important intellectual content; and 3) final approval of the version to be published. Authors should meet conditions 1,2 and 3.

Additionally, our agreement will take notice that a) acquisition of funding, data collection or general supervision of the research group, alone, does not justify authorship; b) all persons designated as authors should have participated sufficiently in the work to take public responsibility for appropriate portions of the content (International Committee of Medical Journal Editors, 2008). With these basic principles as basis, a specific agreement on intellectual property will be included in the consortium agreement. Innovative intervention materials and instruments might consist of teaching materials and books. The consortium will not claim authorship on these materials, but adheres great importance to make them freely available accompanied with good guidelines for effective use. WP10 will be responsible for the dissemination and exploitation of the results and the intervention materials and instruments. This WP will ensure effective use of the newly developed interventions materials (see 3.2.3)

B4. Ethical issues

This chapter will address all ethical issues that might arise about the proposed project. The project concerns mainly social science research and consists of a number of different research categories that can be divided in the following activities:
• Secondary data analyses on existing data;
• Focus group interviews with parents;
• Implementation of the intervention study and process evaluation;
• Assessing impact and outcome
Ethical issues regarding these research activities will be addressed separately in the following sections, dealing with all issues that might arise in social science research and research in young children.
The consortium certifies that all regulations/legislation at European/national level for interventions on humans will be respected and that, where applicable, agreements of local ethical committees will be obtained before the relevant work starts. Copies of these approvals will be sent to the Commission with the respective annual reports. Regulations/Legislation on data protection will be fully respected. The Harokopio University Bioethics Committee will act as an independent ethics expert for the duration of the project, to ensure that appropriate informed consent is obtained from participants.
The ethical issues in the ToyBox project will address the concepts about ethics in biomedical research that originated from the Nuremberg Code and the Declaration of Helsinki. The most recent published guidelines are the ICH, E6 and 2001/20/CE directives, which were written originally for therapeutic clinical trials (available at http://www.ich.org). Following these guidelines and those concerning the ethical conduct of medical research involving children, each centre has named a principal investigator (PI). All information collected as part of this project will be stored in accordance with the Data Protection Acts (1984, 1998). Access to the project materials and data, while the project is underway, will be restricted to members of the research team. Any notes taken and/or any paper based materials you may give us will be stored in a locked filing cabinet for the length of the project, and/or stored electronically on password protected computers.
The final English version of the information letter for parents and teachers and the consent form will be translated by the PIs into their local languages and adapted to the local rules or habits for presenting these documents but they will surely contain: freedom to withdraw from the protocol, general design and details about the tests. The protocol, along with the information letter and consent form adapted to the local language, will be submitted to the local or national IEC of each centre.
Research in young children
The proposed study addresses young children aged 4-6 years old, according the title of the call **KBBE-2009-2-1-03 - Behavioural models for prevention of obesity, with a particular focus on children.** In order to get up to date and accurate information about what unhealthy and healthy behaviours children actually engage in and why, parental involvement is necessary for this young age group.

Minimal risk and burden
The type of research we plan to conduct is social science research and will have a very minimal burden on the participating children.

Systematic reviews and secondary data analyses
The first steps in the project conducted in the WP2 and 4 and 5 will not request actual participation of children. We will use the literature and existing data sets of studies that all are approved by the relevant medical committees.

Focus group interviews
In addition to literature research and secondary data analyses, WP4 will include focus group interviews, but this will involve parents and teachers and not the children. Parents and teachers will be asked to a come to a nearby location to minimize travel costs and time and to optimize the participation rates. They will also be compensated for the costs made and the time invested.
Evaluation of the intervention
WP7 will include an evaluation study of the newly developed intervention in order to validate the new strategy as requested in the call. This study will be conducted in 6 countries and 20 schools per country will be recruited and will evaluate intervention effects for the prevention of obesity. For this evaluation study ethical clearance will be asked from the relevant ethical committees prior to the start of the relevant work. We will further ask informed consent from the parents of the participating children, as described in WP7. However, assent from the children will also be obtained; even if parents have given their consent, children not wishing to participate to part or all of the measurements, will be excluded from these measurements. Furthermore, the intervention itself will be addressed to all children and not to specific groups. Therefore the intervention will not promote stigmatisation of overweight children.

Obtain consent from the legal representative
WP7, which will collect original data among children will fully inform the parents about the contents and the aims of the study and will ask for written consent for the participation of their child and themselves in the ToyBox project. If no consent is available for a child, the child will not participate in the impact and outcome evaluation study. The information provided will depend on the research activity.

Evaluation of the intervention
In WP7, the newly developed intervention will be evaluated. Schools will be recruited first by telephone, and they will be informed about the design of the study in writing, i.e. that after the baseline measurement schools can be assigned to either the intervention or the control condition. They will then be informed about the content of the intervention, and how this might interfere with the regular curriculum. They will as well be informed about the measurements. All verbally transferred information will also be sent to the schools in a letter and a brochure of the intervention. The letter will also explain that all information gathered will be treated anonymously and that information specific for their school will not be available for other schools. Local authorities and/or school boards will decide upon participation in the study evaluating the intervention, and thus decide whether or not the intervention can be implemented in their schools. All children in the intervention condition will be exposed to the intervention materials and activities. Regarding the measurements, i.e. the completion of the questionnaires, parents will receive a letter explaining the purpose of the survey. It will be clarified that all data will be treated anonymously, that they can withdraw any moment, that they are not obliged to participate and that no home addresses will be entered in the data base. The research assistants instructing parents on how to complete the questionnaires will inform them that it is important to give honest answers, that they will not be judged, that all their answers will be treated anonymously, that the teachers and class mates of their child will not be informed about their answers and that, as mentioned above, they can withdraw any moment during the study. In addition, parents will be informed about the content of

the intervention in a separate letter and a brochure. The brochure will show pictures of the intervention materials and explain the content of intervention activities.

Demonstrate awareness of sensitivities when conducting social research
Researchers involved in the ToyBox project are all experienced in the field of obesity research and the promotion of health behaviour, and are therefore all very aware of the sensitive issues related to these topics. We will prevent stigmatisation of obese children by not addressing subgroups of children, but address all children in the class/school. Furthermore, privacy during the completion of the questionnaires (WP7) and the assessment of height and weight (WP7) will be guaranteed. Furthermore, the newly developed intervention to be implemented in the schools will be tailored to the cultural and religious background of the schools and the region, and therefore take into account potential sensitive issues related to this.

Participant response
The research activities to be conducted in the proposed project are not very likely to result in aggressive responses of the child. However, we learned from previous experiences in school based studies that parents might feel that the study interferes with their parenting and relationship with the child. We are aware of the fact that parents not always appreciate this involvement of the project, and might react aggressively. We will anticipate on this and respect all personal opinions and not oblige parents to participate.

Approval for the research protocol
As mentioned earlier, for all studies to be conducted in the proposed project, ethical approval from the ethical committees in participating countries as well as all necessary approvals to enter the school sites will be obtained before the relevant work starts. Before the beginning of the relevant work packages all the necessary approvals (on privacy and data protection) will be submitted to the European Commission.

Intentional deception over the aims of the study
All researchers will be honest about the aims of the study, and parents, schools and children will be fully informed about the content and the aims of the study, as stated earlier. However, to prevent parents and teachers to respond with social desirable answers during the intervention study, it will not be specifically stressed that the intervention aims at increased physical activity, decreased sedentariness and improved diets. In fact, we will stress that it is very important to give honest answers.

If information is withheld
No information will be withheld from the children, parents or teachers.

Any negative effects and misconceptions should be monitored and addressed
As mentioned before, potential negative effect might be stigmatisation of overweight children. We will prevent this by addressing all children and no specific subgroups, in addition we will not stress that this is a study on overweight, but rather on healthy behaviour. Furthermore, privacy during completion of the questionnaires and measurements of height and weight will be guaranteed.

Focus group interviews
The focus group interviews will address the topic of parenting and teaching practices, which might be a sensitive issue and parents / teachers might feel to be judged on their parenting/teaching qualities. To prevent this, all researchers conducting the focus group interviews will be trained to respect all personal opinions, and do their very best to convince the participating parents and teachers that these qualities are not judged by the researchers.

Evaluation of the intervention
During the evaluation of the intervention substantial attention will be given to the process, impact and outcome evaluation of the implemented intervention, this will also include potential adverse

outcomes. Again, we will do our very best to prevent adverse outcomes by preventing stigmatisation and respecting all personal opinions. Furthermore, the ethical committees will also examine the protocols for potential adverse side effects. If any issues arise we will respond adequately or withdraw a specific part of the intervention.

Right to withdraw
We will state in the information sent to parents and schools that participants in the study have the right to withdraw at any moment during the study or withdraw from specific parts of the study (e.g. measurements of height and weight)

Discussing results with parents
Results of the study, e.g. regarding the child's health behaviour or overweight status, will not be discussed with the parents or the teachers during the course of the study. We therefore avoid that evaluation statements carry unintended weight. The official outcomes of the study will be made publicly available according to a dissemination protocol, but these results will not contain personal information.

Observational research

Evaluation of the intervention
As part of the process evaluation carried out in WP7, class observations will be conducted. However, these observations will focus on the teachers, and on how they implement the intervention materials.

Minimum of personal biographical data should be taken
In all survey activities to be conducted in the ToyBox project, we will only ask for relevant biographical information.

Focus group interviews
Participants in the focus group interviews do not need to provide specific personal biographical data. Groups will be homogeneous for socio-economic position, thus groups rather that individuals will be 'labelled' regarding their socio-economic position. No names or addresses will be stored in the files, only gender.

Evaluation of the intervention
The evaluation of the intervention will include a baseline and a follow-up measurement. The questionnaires used in the survey will not ask about family names and home addresses. A unique id number will be assigned to all children and parents. A child-parent couple will receive the same id number; the number will consist of four parts: country number; school number, class number, participant number. Children and parents will be recruited through the schools. A file will be kept containing the combination of the child's name and the unique id number. This separate file will be stored at the national research centres during the course of the study and will not appear in other data bases containing all other data. This list will enable to merge the follow-up data with the baseline data and will be used by the research assistant only. During follow-up the questionnaires and measurements obtained for each child will be assigned with the unique id number assigned to the child during the baseline measurements. Sensitive information will be destroyed after the end of the ToyBox project.

Specific issues related to the use of existing data

Research activities to be conducted in WP2 include secondary data analyses. We will only include data from studies that have ethical clearance from the relevant ethical committees. Furthermore, all data will be anonymous and cannot be directed to personal information. For the studies included for the secondary data analyses (see Table 1, page 26) ethical approval has been obtained as follows:

- Belgium: Ghent University Hospital Ethical Committee
- Bulgaria: University Hospital (UMHAT "St. Marina") Ethical Committee
- Germany: Ethical Committee of the Bavarian Board of Physicians (Bayerische Landesärztekammer)
- Greece: Harokopio University Ethical Committee
- Poland: Institute of Mother and Child and Food and Nutrition Institute
- Spain: Ethical committee of the Spanish Society of Community Nutrition.

ToyBox logo

University logo

Parent Information Sheet

ToyBox Project

What is the purpose of the project?
This project aims to provide evidence on the how well a health promotion programme called ToyBox works. The programme aims to improve eating behaviours and activity levels in children aged 4-6 years old.

Who will be taking part?
About 4800 children 4-6 years old from six European countries (Belgium, Bulgaria, Germany, Greece, Poland, Spain) will participate in the project. Children whose parents/guardians/carers do not object to them (and return the opt-out form) participating in the project will be included.

When will this project run and what will it include?
The project will run between September 2011 and September 2012 and the following measurements will take place:

- You will be asked to fill in a written (parental) questionnaire related to energy-balance behaviours
- We will perform measurement on your child's' height, weight and waist circumference.

All child measurements will take place during school hours and you could fill in the parent questionnaire at home.

What are the possible benefits of taking part?
The information gained from this project will help to show if this new food and activity program can help to improve eating behaviours and activity levels in 4-6 years old children.

Informed consent and confidentiality
It is up to you to decide whether or not you want your child to take part. You and your child can withdraw from the project at any time without giving a reason. If you agree that your child can take part, all the information that we collect from them will be kept strictly confidential, and your child will not be identified in any reports or publications, although your child's school will be named.

Safe storage of information
All information collected as part of this project will be stored in accordance with the Data Protection Acts (1984, 1998). Access to the project materials and data, while the project is underway, will be restricted to members of the research team. Any notes taken and/or any paper based materials you may give us will be stored in a locked filing cabinet for the length of the project, and/or stored electronically on password protected computers.

Contact details
For any further information you may require, please contact at working days:

Name: ______________________________

Telephone: ______________________________

E-mail: ______________________________

ToyBox logo

University logo

Certificate of consent

I have read the information form and the information supplied is clear to me.
If I do have more questions, I know whom to call.

I agree with my child's participation in the study described in the information form and I am willing to participate in the study as well.

I have been informed that my child's and my participation in this study are voluntary. I am free to withdraw and / or my child from the study any time and without need to give a reason. Choosing not to participate or withdrawing from this study will not result in any disadvantages for me or my child.

Name of child (in block letters)

____

Name of parent/ caretaker (in block letters)

____

City, date

____

Signature parent/caretaker

____

Name researcher: ________________

DRAFT

ETHICAL ISSUES TABLE

	Research on Human Embryo/ Foetus	YES	Page
	Does the proposed research involve human Embryos?		
	Does the proposed research involve human Foetal Tissues/ Cells?		
	Does the proposed research involve human Embryonic Stem Cells (hESCs)?		
	Does the proposed research on human Embryonic Stem Cells involve cells in culture?		
	Does the proposed research on Human Embryonic Stem Cells involve the derivation of cells from Embryos?		
	I CONFIRM THAT NONE OF THE ABOVE ISSUES APPLY TO MY PROJECT	X	
	**Research on Humans**	**YES**	**Page**
	Does the proposed research involve children?	X	All
	Does the proposed research involve patients?		
	Does the proposed research involve persons not able to give consent?		
	Does the proposed research involve adult healthy volunteers?	X	WP3, WP7
	Does the proposed research involve Human genetic material?		
	Does the proposed research involve Human biological samples?		
	Does the proposed research involve Human data collection?	X	WP7
	I CONFIRM THAT NONE OF THE ABOVE ISSUES APPLY TO MY PROJECT		
	**Privacy**	**YES**	**Page**
	Does the proposed research involve processing of genetic information or personal data (e.g. health, sexual lifestyle, ethnicity, political opinion, religious or philosophical conviction)?	X	WP7
	Does the proposed research involve tracking the location or observation of people?		
	I CONFIRM THAT NONE OF THE ABOVE ISSUES APPLY TO MY PROJECT		
	**Research on Animals**	**YES**	**Page**
	Does the proposed research involve research on animals?		
	Are those animals transgenic small laboratory animals?		
	Are those animals transgenic farm animals?		
	Are those animals non-human primates?		
	Are those animals cloned farm animals?		
	I CONFIRM THAT NONE OF THE ABOVE ISSUES APPLY TO MY PROJECT	X	
	**Research Involving Developing Countries**	**YES**	**Page**
	Does the proposed research involve the use of local resources (genetic, animal, plant, etc)?		
	Is the proposed research of benefit to local communities (e.g. capacity building, access to healthcare, education, etc)?	X	WP5
	I CONFIRM THAT NONE OF THE ABOVE ISSUES APPLY TO MY PROJECT		
	**Dual Use**	**YES**	**Page**
	Research having direct military use		
	Research having the potential for terrorist abuse		
	I CONFIRM THAT NONE OF THE ABOVE ISSUES APPLY TO MY PROJECT	X	

B5. Gender aspects

In ToyBox we will consider gender aspects both in the project consortium as well as in our research.

B 5.1 Participation of women

In the project consortium we try to account for gender equality and equity in composition of research teams. This is being done by:

Involving as many female partners as male partners in the consortium. In the current composition of the consortium, women are already fairly represented. Of the current participants 58.5% are women. In addition to this, there are 10 vacancies. In order to maintain this equal gender balance, we will strive for 50 % of these positions to be filled by women.

Promoting the role of women in leading positions within the WPs. Women are involved in the scientific management of ToyBox. Of the ten WP leaders, 3 are women.

Enabling part-time work. This will be possible for all positions within the project. Women will also be allowed to take part-time positions for a limited period and to take up full-time positions after that if they wish. This will enable them to deal with changing family circumstances. In addition, during meetings and workshops childcare will be organised, in order to stimulate participation of mothers/fathers.

5.2 Gender aspects in research

The most recent data show that obesity among children is more prevalent in girls than boys, while boys spent more time both in physical activity as well as screen behaviours such as television viewing and playing computer and video games. Nevertheless, in our research gender bias will be avoided and males and females will be considered equally. Gender issues will be carefully addressed at all steps of the research process in the sense of awareness and taking into account differences and similarities between sexes.

Gender differences will be considered in the examination of the energy balance-related behaviours as well as the behavioural determinants. In WPs 2 and 3 gender will be one of the potential moderators of intervention effects that will be examined. The development, implementation and evaluation of the intervention programme will be performed in a gender sensitive manner taking gender issues and differences into account. E.g. both obesity and eating disorders are much more common in females than in males. Therefore, in the development of the intervention attention will also be paid to preventing the development of possible negative dietary behaviours such as dropping meals and eating disorders.

Furthermore, we expect to find differences between males and females in energy balance-related behaviours and their determinants. It may be that family processes or environments that tend to lead to overweight in boys may not play the same role for girls. This will be carefully assessed and taken into account in the development of the intervention. If necessary, possible gender differences may result in the development of intervention components specifically for either males or females. In the end, the designed intervention should be able to offer both sexes an equal opportunity to prevent obesity and obtain a healthy life style.
